# A Survey of Plant Iron Content—A Semi-Systematic Review

**DOI:** 10.3390/nu7125535

**Published:** 2015-12-10

**Authors:** Robert Ancuceanu, Mihaela Dinu, Marilena Viorica Hovaneţ, Adriana Iuliana Anghel, Carmen Violeta Popescu, Simona Negreş

**Affiliations:** 1 Faculty of Pharmacy, Department of Pharmaceutical Botany and Cell Biology, Carol Davila University of Medicine and Pharmacy, Bucharest 20956, Romania; robert.ancuceanu@umf.ro; (R.A.); marilena.hovanet@umf.ro (M.V.H.); adriana.anghel@umf.ro (A.I.A.); 2 Pharmacy and Dental Medicine, Faculty of Medicine, Department of Microbiology, Virology and Parasitology, “Vasile Goldis” Western University, Arad; S.C. Hofigal S.A, Bucharest 042124, Romania; carmen_popescu@hofigal.eu; 3 Faculty of Pharmacy, Department of Pharmacology, Carol Davila University of Medicine and Pharmacy, Bucharest 20956, Romania; simona_negres@yahoo.com

**Keywords:** iron, herbal organs, taxonomic groups, life-forms, food supplements, anemia

## Abstract

Iron is an essential mineral nutrient for all living organisms, involved in a plurality of biological processes. Its deficit is the cause of the most common form of anemia in the world: iron deficiency anemia (IDA). This paper reviews iron content in various parts of 1228 plant species and its absorption from herbal products, based on data collected from the literature in a semi-systematic manner. Five hundred genera randomly selected from the Angiosperms group, 215 genera from the Pteridophytes groups and all 95 Gymnosperm genera as listed in the Plant List version 1.1 were used as keywords together with the word “iron” in computerized searches. Iron data about additional genera returned by those searches were extracted and included in the analysis. In total, iron content values for a number of 1228 species, 5 subspecies, and 5 varieties were collected. Descriptive and inferential statistics were used to compare iron contents in various plant parts (whole plant, roots, stems, shoots, leaves, aerial parts, flowers, fruits, seeds, wood, bark, other parts) and exploratory analyses by taxonomic groups and life-forms were carried out. The absorption and potential relevance of herbal iron for iron supplementation are discussed.

## 1. Introduction

Iron is a vital nutrient for the human body, playing an essential role in a variety of cellular activities [1]. It functions as a cofactor in numerous enzymes involved in the biosynthesis of certain amino acids, hormones, neurotransmitters and collagen [2]. The iron content in the body is tightly regulated, as both deficit and excess may have harmful consequences [2]. Estimates of the iron needs in humans vary according to various authorities, depending on bibliographic source, methodology and assumptions (for instance, reference nutrient intakes, RNI, are higher in the USA than in the UK) [3]. It is accepted that men and older women (over 51 years of age) need about 8 mg iron a day in their diet (8.7 mg officially in the UK, 8 mg in the USA). Women of childbearing age need considerably higher amounts, to compensate for the menstrual blood loss, about 18 mg per day (officially, 14.8 mg in the UK); during pregnancy iron need is still higher, 27 mg per day [3,4]. In children, iron needs vary according to age, being higher in the first two years of life, then lower and almost doubling in adolescence [5]. 

Anemia is defined as “hemoglobin concentration below established cutoff levels” [6]. It is not a disease, but a state reflecting a nutritional deficit or—sometimes—an underlying disorder [7]. As for the cutoff levels, most studies carried out so far have used the values suggested by a WHO expert committee at the end of the 1960s: 13 g/dL for men, 12 g/dL for women [8]. However, uncritical, extended use of these values, driven by the authority lent by “the imprimatur of the WHO” has been challenged with apparently good reason, as the WHO document recommending those cutoff thresholds was based on a very limited number of data generated with inadequate methods [9,10]. It has been estimated that, at worldwide level, anemia affects about 20%–30% of the population [7,11], with a high prevalence in developing countries [7]. Iron deficiency anemia (IDA) is the most common form of anemia in the world [12] and the most frequent form of anemia in pregnant women [13]. 

The multiple negative effects of anemia on health and the quality of life justify interventions designed to prevent and control anemia, one of which is the use of iron-containing food supplements. Although the priority in controlling anemia is recognized for pregnant and postpartum women, as well as for children of 6–24 months of age [14], other subpopulations may also need iron supplementation to improve their hemoglobin (Hb) level.

Whereas most iron-containing food supplements are based on inorganic or organic derivatives of iron obtained by chemical synthesis, and while iron of herbal origin is not as easily absorbed, a certain interest exists for food supplements containing iron of herbal origin, especially for a segment of the public charmed by the idea of “returning to nature”. To be able to scientifically formulate such a food supplement, knowledge of the iron level in various plant species and the factors that influence iron contents in the plant world is necessary.

How much of the plant world has been investigated in terms of iron contents? Which plant organs have been investigated most from this standpoint and which have been studied least? Are there any detectable patterns? (e.g., are herbs richer in iron than trees, shrubs or vines? Are there any differences among Angiosperms, Gymnosperms, and Pteridophytes or between Monocots and Eudicots? Are certain plant parts more abundant in iron than others?) How well is iron from plant sources absorbed by the human body? This paper is a semi-systematic review aiming to answer these questions based on a sample of iron values derived from 1228 species (the reasons for choosing a semi-systematic review instead of a systematic one are discussed in the following section). 

## 2. Materials and Methods

### 2.1. Search Strategy

A systematic review of the literature on iron contents in plants has been beyond the reasonable possibility of the authors because of the ubiquitous character of iron and, consequentially, the non-efficient character of usual database interrogation techniques. We have therefore decided for a semi-systematic approach, based on a long list of plant genera randomly sampled in a stratified manner from the Plant List version 1.1 [15]). We have downloaded all genera available for Angiosperms, Gymnosperms, and Pteridophytes as three distinct lists and randomly selected 500 genera for Angiosperms and 215 genera for Pteridophytes to be used as keywords (together with “iron”); all 95 Gymnosperm genera have been used as keywords (the genera names used for each taxonomic group are provided as electronic supplementary material). True random numbers have been generated using the R package, “random” (2013) [16]. Each genus keyword plus the word “iron” have been used in computerized searches in Pubmed, Proquest Central, and Google Scholar. Similar searches have also been carried out in the “Plants for the future” database [17]; for this database we have additionally performed a generic interrogation with the keyword “iron”. Not only data about the specific keyword have been retained for analysis, but all data about iron contents in plant species returned for that specific keyword; if a result contained iron data on different genera than that looked for (e.g., because the genus aimed for was cited in a reference within the paper), that publication has also been used for data extraction. We have classified the species for which iron contents data were found according to their life-form and according to the respective taxonomic classification; for the latter, the APG III classification [18] has been in principle followed, but, in order to have groups of a meaningful size, we have aggregated Nymphaeales together with Magnoliids, Commelinids with Monocots and treated all other taxonomic groups as Dicots. 

Sample size calculations have been based on the number of keywords (sampling from the total number of genera names) and not on the herbal species analyzed. The possibility has been considered to detect a difference in the proportions between the genera investigated for iron content for at least one plant part among the three main taxa (Angiosperms, Gymnosperms and Pteridophytes) with a power of 80%, at a 0.05 level of statistical significance and for a medium effect size (0.3 according to Cohen) by the chi-square test (*df* = (3 − 1) × (2 − 1)): a sample size of minimum 108 would have been necessary. The assumption has also been made that, because papers often report on iron contents in more than one species and genus, the number of negative results of the interrogation for many plant genera will be compensated by the multiple reports included in single papers and thus we were expecting to retrieve information on about 800 genera and 1000 species. Sample size calculations were carried out using the R package “pwr” [19].

In addition to plant data, we have used “herbal iron absorption” and “plant iron absorption” as MeSH terms in Pubmed to screen for all publications available in this database on non-heme iron absorption. Searches for both iron contents and iron absorption have been carried out in English, but publications in other languages (e.g., French, Spanish, German, Chinese) for which at least an abstract in English was available, have also been included.

### 2.2. Study Eligibility and Data Extraction

Inclusion in the study has been conditioned on reporting on iron contents in lycophytes, pteridophytes, gymnosperms, angiosperms and iron absorption in humans or animals; papers reporting *in vitro* availability of iron have also been included, but different degrees of confidence in the results have been applied (clinical data > animal data > *in vitro* data). Titles and abstracts returned by the searches have been appraised by one evaluator and in the case of doubt by two additional evaluators; publications found to be obviously irrelevant according to the information contained in the title and/or abstract have been excluded. Systematic reviews were mainly used to identify other potentially pertinent publications. Studies not reporting the reasonable identification of at least one plant species and organ for which iron content was assessed have been excluded; when the same study reported on iron values in several plants, only values for which a clear identity was available have been retained for review. For instance, in certain publications, authors have considered genera names (e.g., *Tilia* ssp. [20], *Pinus* sp. [21], *Pyrrosia*, *Epimedium* [22], *etc.*) sufficient for herbal product description; the data for these genera have not been included in our review, but data for species completely identified in the same papers have been retained. Some publications only provided the plant name, with no details on the herbal parts used [23]. For instance, a study reported on iron content in “linden” (further described in the text as “*Tilia vulgaris*”, which is not a species, but an illegitimate name for a hybrid), but it was not clear whether the product included the inflorescence bracts or was limited to flowers only; in this same study, “senna tea (*Cassia anqustifolia*)” (*Cassia angustifolia* Vahl, a synonym for *Senna alexandrina* Mill.) was also reported, but although it may consist of leaves or pods, it was not clear from the paper to which the results refer [24]. Minor nomenclature errors (such as the above “anquestifolia” instead of “angustifolia”) were relatively frequent, subsequently corrected in the extraction process. For each species, the currently accepted name in The Plant List v. 1.1. has been checked and the reported name has been replaced with the current one, where relevant. Studies reporting iron content on a fresh basis were excluded if water content was not simultaneously reported (if reported, results have been converted by us on a dry basis). When a single point estimate was reported, this has been tabulated. When more than one result was available in a paper for a defined species, the minimum and maximum values have been tabulated, so as to provide a complete picture of the range of values. When several papers reported on iron contents in a certain species (and herbal part), the point estimate or the minimum and maximum values, as appropriate, have been collected from each paper.

Leaves have been the parts most widely collected and analyzed for iron contents and, therefore, we used them as a reference to compare iron contents from other parts. In addition to the global comparison, to control for confounding from other variables we defined subsets of data consisting of values reported by the same publication for two different variables (e.g., leaf and root, leaf and stem, young leaf and mature leaf, *etc.*) and compared iron concentrations in these paired variables. 

### 2.3. Statistical Analysis

All statistical analyses have been performed with the R computing and programming environment [25] and several R packages, as detailed below. Normality has been assessed by visual examination of the data (histograms, boxplots, q-q plots) and for an additional objective evaluation the d’Agostino-Pearson omnibus test has been applied for *n* > 20 and Shapiro-Wilk test, for *n* < 20, with the “fBasics” R package [26]. Homoscedasticity has been evaluated using a modified robust Brown-Forsythe version of the Levene-type test, based on substituting the mean with the median, as implemented in the R “lawstat” package [27]. Due to the nonnormality of most data sets, the median was used as the most relevant central tendency measure and 95% confidence intervals have been computed by bootstrapping with the bias corrected and accelerated method (BCa), using the “simpleboot” R package [28] and 10,000 replicates. Outliers have been identified visually on histograms, but, for the purpose of a more objective evaluation, the R package “extremevalues” has also been applied [29]. Although not normally distributed, data were often homoscedastic and, therefore, the Mann-Whitney (Wilcoxon rank sum test) and Kruskal-Wallis tests have been used to compare iron concentrations between different two and multiple continuous variables, respectively; for paired values, the Wilcoxon signed rank test has been employed. Nonparametric relative contrast effects based on global rankings have been computed with a Tukey-type test, based on the Fisher transformation function as the asymptotic approximation method, in the implementation of the npcarcomp R package (function “mctp”) [30]. For sensitivity analysis purposes, nonparametric relative contrast effects have also been computed with different asymptotic approximation methods (and the results were equivalent). In the few cases where data were both nonnormal and heteroscedastic, to compare two groups the Welch’s *t* test has been applied on the ranked data, and to compare more than two groups Welch’s ANOVA has been used on the ranked values, as these have been shown to have the best control on the type I error, with little impact on power [31]; for ANOVA, in this case, ranked-based, nonparametric multiple contrast tests have also been used, as proposed by F. Konietschke, L.A. Hothorn and E. Brunner (2012) [32] and implemented in the nparcomp package [30]. In addition to the relative effect size as computed by “nparcomp” (where relevant), Hedge’s g unbiased estimator computed by the “effsize” [33], R package has been used for two group comparisons; for Kruskal-Wallis, eta [34] and epsilon squared [35,36] computed manually in R have been used as effect size measures. Chi-square test (R package) has been employed to compare frequencies, without applying the Yate’s correction (its use is controversial and rather “out of fashion” and of little relevance even for small sample sizes with expected frequencies lower than 5 or 10 [37,38]). Segmented regression of dialyzable iron from *Amaranthus* leaves has been performed with the R “segmented” package [39,40] a Davies test (k = 50) has been applied to evaluate the significance of the slope change. Natural cubic spline modeling of the same dataset has been conducted with the “ns” function of the R package “spline” (of the core R). Graphics have been built with “ggplot2” [41] and “lattice” [42] R packages.

## 3. Results

### 3.1. Extent of Iron Content Investigation in Plants

Searches in Pubmed, Proquest Central and Google Scholar using the set of keywords indicated above (including references from papers thus returned) have identified 200 publications reporting iron content in various plant species and organs. The additional interrogation of the “Plant for a future” database has returned 595 potentially relevant results, of which only the first 100 have been accessible and of these, 72 results have been relevant. In total, iron contents values for a number of 1228 species, 5 subspecies and 5 varieties have been collected (Table 1). Although we have not included keywords for bryophytes, data for a few species from this taxonomic group have also been obtained in our survey of the literature (which have been included in the 1228 species). The complete plant list, including the relevant references, taxonomic information and growth habit information are included in the Electronic supplementary material, Table S1).

**Table 1:** Synthetic overview of the data collected in our review including iron concentration variation among different plant parts.

Plant Part	Number of Species ^a^	Number of Families	Minimum Iron Conc. (mg/kg, dwb ^b^)	Maximum Iron Conc. (mg/kg, dwb ^b^)	Median Iron Conc. (95% CI) (mg/kg, dwb ^b^)	Mean Iron Conc. (95% CI) (mg/kg, dwb ^b^)
Root	66	33	1.9	111,200.0	502.4 (259.3–691.0)	5706.0 (2750, 11,560)
Stem	60	34	7.3	25,650.0	171.0 (69.2–313.4)	1431.0 (829, 2696)
Shoot	32	22	20.2	9418.0	91.0 (72.7–101.5)	513.5 (227.1–1113.8)
Bark	41 ^c^	19	3.6	1585.0	45.0 (35.0–57.0)	106.8 (74.3–188.8)
Leaf	632 ^d^	155	0.1	24,070.0	167.0 (155.2–186.6)	489.4 (401.8–618.4)
Aerial parts	295	89	0.0	27,100.0	240.1 (216.5–263.3)	596.9 (468.4–900.8)
Flower	28	15	15.7	5139.0	159.9 (91.2–194.1)	426.1 (187.5–1008.1)
Fruit	200 ^e^	62	0.0	8424.0	72.6 (61.0, 87.7)	257.9 (195.2–393.3)
Seed	104	42	0.0	11,610.0	70.2 (53.8–90.0)	522.6 (333.0–894.4)
Whole plant	41	25	11.4	70,480.0	156.0 (89–747)	2785.0 (1072–9184)
Wood	35	15	0.0	35.0	0.0 (N/A)	3.4 (1.9–6.5)
Other parts ^f^	30	28	0.7	3730.0	141.0 (80.0–215.0)	293.1 (179.3–657.2)

^a^ Given that, for some species, iron values were available for several plant parts, whereas in the case of others iron values were available only for one or two parts, the total in this column adds up to 1562 and not 1228. The same reason explains the apparent discrepancy regarding the number of subspecies and varieties (one organ was reported for the species, while a different organ for a subspecies or variety of the same species); ^b^ dwb = on a dry weight basis; ^c^ + 1 subspecies; ^d^ + 2 subspecies + 3 varieties; ^e^ + 1 subspecies; ^f^ aril, bud, bulb, calyx, false fruit, leaf pulp *etc.* (see Figure S22).

Of the 500 angiosperm genera used as keywords, we have found iron content values reported in the scientific literature for only 35 (7.00%; 95% CI 4.99%–9.69%). In the case of Pteridophytes, of 215 genera used as keywords, iron contents were reported in publications for 13 genera (6.05%; 95% CI 3.39%–10.35%). Gymnosperms are the smallest taxonomic group of the three analyzed in our study, with only 95 genera of which 27 have been found to have been investigated for their iron contents, giving the highest rates of positive results in terms of genera (28.12%). For five angiosperm genera, six potentially relevant papers (*i.e.*, potentially containing information on iron contents) were not accessible, two being in Chinese, and four being relatively old); were they all relevant (which they seemed, judging from the abstract information), the proportion would increase to 8.2% for this taxonomic group (95% CI 6.02%–11.05%). For six Gymnosperm genera, a handful of papers have also been found potentially available, three only being in Chinese, and the other three in English. Were they all relevant, the proportion would increase to 33.68% (95%; CI 24.51%–44.20%). 

**Figure 1:**
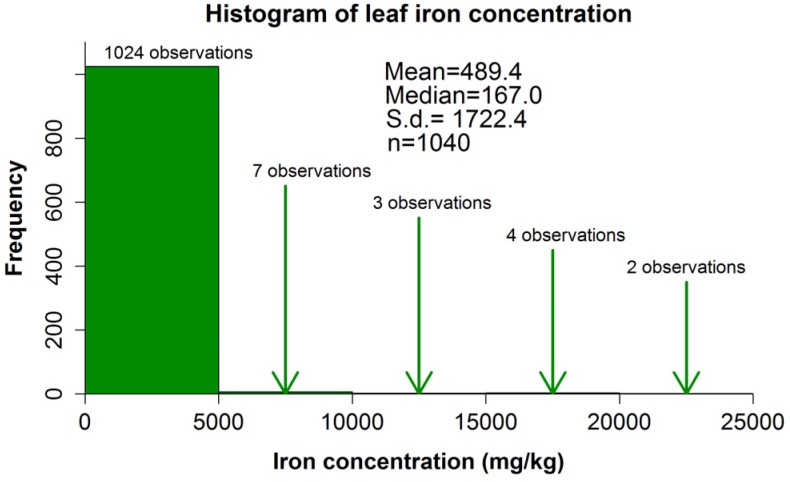
Histogram of iron concentration in the leaf. A zoomed-in histogram (without the largest outliers, covering the interval 0–5000 mg/kg) is provided as an electronic supplementary material, Figure S3).

For all plant parts analyzed, the distribution of iron concentration was positively skewed, *i.e.*, with a posterior tail (an illustrative histogram is provided in Figure 1 and histograms for all part parts are provided as supplementary material, Figures S1–S11), because, for most species, the iron concentration was low. There are significant differences between different parts with respect to iron concentrations (*p* < 0.001); relevant contrasts will be discussed in the context of each plant part.

No family seems characterized by remarkably high or low iron concentrations and the distribution of iron contents inside a family is spread on relatively large intervals; for most families the data were limited to a small number of observations (Figure S12).

### 3.2. Iron Contents in Various Herbal Parts

The variation of iron contents in various herbal parts and distribution by taxonomic group and life-form is shown synthetically in Table 2 and Table 3.

**Figure 2:**
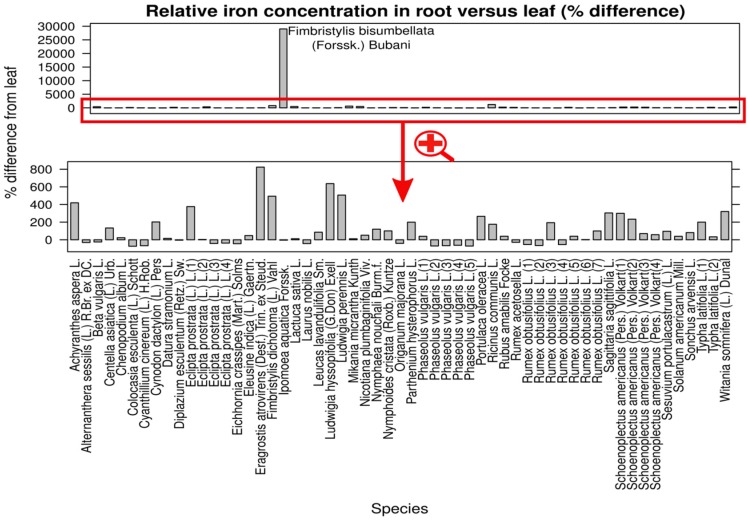
Iron concentration in root *versus* leaf. The bars show the differences between iron concentrations in the two organs, computed as percentages from the leaf concentration (bars over zero indicate higher contents in roots, bars under zero indicate higher contents in leaves). A graph with the absolute values is provided as a supplementary electronic material—Figure S13.

Of all plant parts, roots have had the highest iron contents, significantly higher than leaves (*p* = 0.003, nonparametric Tukey contrast). In the paired leaf-root subset, the frequency of observations for which root iron has been higher than leaf iron has been significantly greater for root than for leaf (67.80% (40/59); 95% CI 54.2%–79.0%; *p* = 0.09 (chi-squared) (Figure 2 and Figure S13). In this dataset, the median value of iron concentration in root has been 600 mg/kg, whereas the median in leaf has been 508.4 mg/kg. The difference is significant (*p* = 0.009), but of relatively small importance (Hedges’s g 0.314).

Stem and leaf iron levels seem to be very similar (relative effects—0.578 leaf, 0.576 stem; median rank 1197.8 in leaf, 1211.5 in stem; nonparametric Tukey contrast, *p* = 1.000). The paired dataset also showed no statistically significant difference in iron contents between the two organs (340.6 mg/kg in leaf *versus* 180.9 mg/kg in stem, *p* = 0.201) (Figure S14).

**Figure 3:**
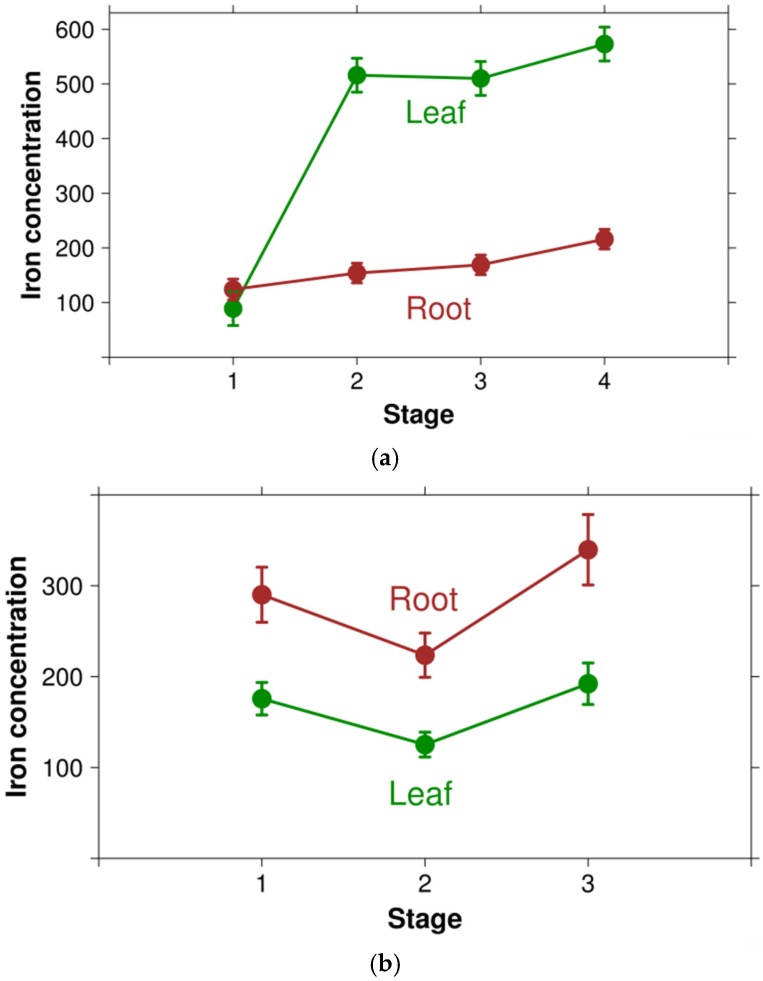

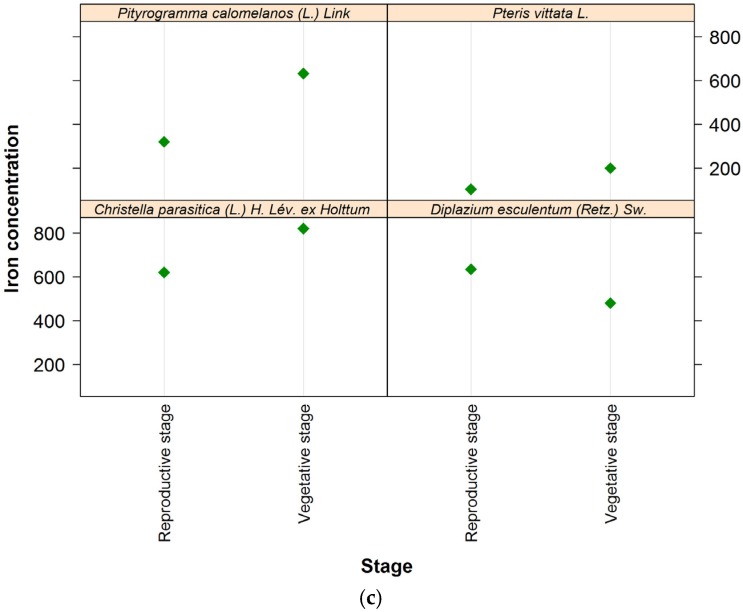
(**a**) Variation of iron concentration along four life stages in *Phaseolus vulgaris* L. (based on [45]). The error bars are used as declared by the authors, who have not stated the nature of the error measurement, however; (**b**) Variation of iron concentration along three life stages in *Lactuca sativa* L. (based on [46]. The error bars are 95% confidence intervals; (**c**) Variation of iron concentration in the leaves of four fern species depending on life stage. Based on [47].

As reported by five different bibliographic sources, the leaves of *Spinacia oleracea* L. have an iron concentration varying between 73.2 [43] and 416.5 [44] mg/kg, with a median of 156.2 mg/kg and a mean of 192.3. The corresponding median and mean ranks are 1147.0 and 1135.0, both corresponding roughly to the 51st percentile of concentration ranks. In other words, published data for iron concentration in spinach leaves indicate that they have an unimpressive middle of the range iron content, far from the (still) widely accepted high content of the popular culture imagery. In a paired dataset of 29 observations for young *versus* mature leaves, no significant difference has been found between the two types of leaves (*p* = 0.329, Wilcoxon ranked test). The two studies measuring iron concentration in more than two time points in leaves have revealed different patterns: in *Phaseolus vulgaris* L., J. Ayala-Vela *et al.* (2008) measured iron in leaves at four stages: 50% of flowering (stage I), beginning of seed filling (stage II), pod filling (stage III) and “physiological maturity” (stage IV). Between stage I and stage II, iron level increased about 5-fold, almost reaching a plateau afterwards (Figure 3a) [45]. V. Pillay and SB Jonnalagadda (2007) measured iron in leaves of *Lactuca sativa* L. at three stages: 25 days of growth (stage I), 45 days of growth (stage II) and 75 days of growth. They found a nonlinear, U-shaped pattern, with highest values at 75 days (stage III), lowest values in stage II and intermediary values at 30 days (stage I) [46] (Figure 3b). V. P. Masal and M. Meena (2010) reported that, in three fern species (*Pityrogramma calomelanos* (L.) Link, *Pteris vittata* L. and *Christella parasitica* (L.) H. Lév. ex Holttum), leaves in the reproductive stage contained lesser amounts of iron than in the vegetative stage, whereas in a fourth species (*Diplazium esculentum* (Retz.) Sw.) the reverse was true [47] (Figure 3c).

**Table 2:** Synthetic overview of iron concentration variation by taxonomic groups.

Plant Part	Pteridophytes (Median) (95% CI) (*n* ^a^)	Gymnosperms (Median) (95% CI) (*n*)	Magnoliids (Median) (95% CI) (*n*)	Dicots (Median) (95% CI) (*n*)	Monocots (Median) (95% CI) (*n*)	Relevant Statistical Comparisons
Root	296.5	NA	259.3	426.5	573.9	M ^b^ *versus* D ^c^: *p* = 0.443 (Mann-Whitney)
	NA	NA	194.0–37856.4	186.0–985.9	394.3–1100.0	
	2	0	3	44	17	
Stem	42.0	175.65	7783.7	140.8	325.0	M ^b^ *versus* D ^c^: *p* = 0.584 (Mann-Whitney)
	27–50	NA	NA	59–441	115.1–550.0	
	3	1	1	39	16	
Leaf	200.0	133.6	253.2	163.0	188.0	G ^d^ *versus* D: *p* = 0.017;
	109.5–238.6	109.0–155.0	166.6–277.5	152.0–193.0	141.0–240.0	G *versus* M: *p* = 0.038;
	33	42	34	438	82	G: *versus* Mag ^e^: *p* = 0.005; *
Shoot	119.2	NA	NA	94.4	89.0	M ^b^ *versus* D ^c^ *versus* P ^d^: *p* = 0.941
	53.4–128.0	NA	NA	52.15–134.50	72.7–92.0	
	4	0	0	20	8	
Aerial parts	156.0	353.5	487.0	225.0	305.0	M *vs.* D: *p* = 0.022 (nparcomp) (Hedges’s g 0.416)
	109.0–223.0	NA	NA	200.0–243.0	220.0–522.0	
	25	2	2	186	64	
Flower	NA	NA	2631.45	159.9	NA	NA
	NA	NA	NA	88.3–193.6	NA	
	0	0	2	26	0	
Fruit	NA	NA	96.42	69.9	67.8	Kruskal Wallis: *p* = 0.486
	NA	NA	77.85–155.00	58.00–87.70	37.60–186.20	
	0	0	9	178	13	
Seed	NA	7.2	14.5	80.5	59	M *vs.* D: *p* = 0.098 (Mann-Whitney)
	NA	1.5–41.1	0.80–264.60	60.0–99.8	4.0–70.0	
	0	5	5	83	11	
Whole plant	83.0	35.2	NA	427.0	118.0	M *vs.* D: *p*= 0.7976 (Mann-Whitney)
	45.0–106.5	NA	NA	83.2–1317.5	72.5–3041.0	
	3	1	0	26	9	
Wood	NA	1.2	1.75	0	NA	NA
	NA	0.0–3.1	NA	NA	NA	
	0	15	2	18	0	
Bark	NA	60.5	14.5	42.0	20.0	G *vs.* D: *p* = 0.089 (Welch t on ranks)
	NA	37.0–120.0	9.85–123.65	24.0–45.7	NA	
	0	15	3	23	1	
Other parts	240	35.6	2065	117.9	248.0	NA
	NA	15.40, 37.15	NA	71.1–190.8	45.0–317.0	
	1	2	1	18	8	

Bryophytes species (*n* = 19) not included in the table. ^a^ *n* = number of unique species (number of data points is in most cases larger); ^b^ M = Monocots; ^c^ D = Dicots; ^d^ G = Gymnosperms; ^e^ Mag = Magnoliids. * Kruskal-Wallis for all groups, *p* = 0.015; all other intergroup comparisons nonsignificant (*p* between 0.215 and 0.999) (nparcomp).

**Table 3:** Synthetic overview of iron concentration variation by life-form.

Plant Part	Herb (Median) (95% CI) (*n*)	Tree (Median) (95% CI) (*n*)	Shrub (Median) (95% CI) (*n*)	Subshrub (Median) (95% CI) (*n*)	Vine (Median) (95% CI) (*n*)	Relevant Statistical Comparisons
Root	506.2	226.7	NA	559.3	1520.0	NA
	288.4–893.6	125.0–1401.8	NA	137.8–15,777.0	NA	
	57	4	0	4	1	
Stem	171.0	313.3	59.0	160.85	485.6	NA
	58.0–441.0	38.0–313.4	37.0–73.0	NA	13.6–1300.0	
	46	4	4	2	4	
Leaf	200.0	149.2	162.0	263.4	397.2	H ^a^ *vs.* T ^b^: *p* < 0.0001 (Welch t) (Hedges’s g 0.216)
	161.6–218.0	134.5–160.0	120.0–210.7	133.8, 351.0	78.8, 532.0	
	273	255	73	21	7	
Shoot	91.0	49.05	108.6	6293.9	6010.0	NA
	72.7–92.8	34.50–82.55	28.5–138.4	NA	NA	
	21	4	4	2	1	
Aerial parts	240.2	220.5	259.0	266.5	NA	Kruskal Wallis: *p* = 0.963
	209.0–274.2	200.0–300.0	206.0–308.4	127.0–458.0	NA	
	195	28	52	18	0	
Flower	159.9	161.9	16.7	84.4	NA	NA
	79.7–261.8	108.5–204.6	NA	NA	NA	
	13	13	1	1	0	
Fruit	100.83	68.0	50.5	152.4	134	H *vs.* T: *p* < 0.0001 (Hedges’s g 0.411)
	67.0–240.0	56.5–81.4	40.0–129.1	NA	77.5–155.0	
	24	146	19	1	3	
Seed	76.0	53.2	151.9	2380	11	H *vs.* T: *p* = 0.244 (Welch t on ranks)
	59.5–94.9	41.1–122.2	4.3–4954.0	NA	NA	
	69	27	5	1	2	
Whole plant	172	29.9	520	NA	NA	NA
	85.0–793.0	NA	26.5, 1317.5	NA	NA	
	33	2	6	0	0	
Wood	NA	0	NA	NA	NA	NA
	NA	NA	NA	NA	NA	
	0	35	0	0	0	
Bark	NA	45.7	NA	NA	NA	NA
	NA	35.1–58.5	NA	NA	NA	
	0	41	0	0	0	
Other parts	233.5	113.1	164.8	164.1	NA	NA
	41.0–395.9	67.9–190.8	NA	NA	NA	
	13	14	2	1	0	

Pseudo-tree species (*n* = 11) not included in the table. ^a^ H = herbs; ^b^ T = trees.

In two papers [48,49], comparative data have been identified for leaf iron levels in specimens collected from unpolluted and polluted areas. One compared data on plants grown nearby an iron steel factory with those grown in a botanical garden (serving as control group), while the other compared plants grown in crude-oil contaminated soils with plants from normal (non-contaminated) soils. A third paper has been excluded dealing with a plant species grown on three different soils, which the authors qualified as more or less polluted, not based on an objective criterion (e.g., a known polluting factor), but by the mere presence of higher levels of heavy metals in the respective soils [50]. Although reporting iron contents on species grown in polluted areas, other papers could not be included in this subset because they were not comparative in nature (did not report on same species harvested more or less simultaneously from non-polluted areas in the same geographical area). By pooling the two datasets, we have found that, of 16 species, for 15 of them (93.75%) plants grown in polluted areas had a higher iron level than those from non-polluted area and in only one the reverse was true (*p* < 0.001, chi squared); the latter species was one of the four grown on crude oil-polluted soil. Data suggest that both sources of pollution tend to increase iron internalization by plant species, but while in the case of the steel factory this may be easily understood, in the case of crude oil pollution identification of the mechanism possibly leading to an increase is not as simple (but the latter is based on only four data points, of which in one case crude oil pollution seemed to have a negative effect on iron absorption) (Figure S15).

A. Rangarajan and J.F. Kelly (1998) [51] investigated the iron contents and *in vitro* iron dialysability for several species of genus *Amaranthus*, grown in open fields and in greenhouses. The authors did not compare specimens grown in the two different habitats, (probably) because of differences in treatments (e.g., fertilizers applied) and the time of harvest (plants grown in open field were collected 35 days after seeding, whereas those cultivated in the greenhouse were collected 28 days after transplanting, *i.e.*, 42 days after seeding). Plotting the results for the two habitats suggest substantial differences in iron contents between the specimens grown in different environments (Figure S16). The hypothesis of a potentially large difference between open field and greenhouse plants is worth further examination, but, because the two groups were not really comparable, no statistical testing has been performed on this subset.

B. Musa and E.O. Ogbadoyi (2012) have investigated the leaf position influence on the iron level in this vegetative organ in *Telfairia occidentalis* Hook. f. (Cucurbitaceae), using both a control soil and a nitrogen-fertilized one [52]. Their results suggest that nitrogen supply slightly increases iron absorption, but the authors conducted no statistical inference test (applying t-tests on data published we have found that the differences were not significant, however consistent across the three leaf positions, which indicates that increasing the sample size might result in statistical significance, currently this being a hypothesis only, yet to be confirmed). However, the finding would be in line with other few reports (in monocots) showing that nitrogen supply tends to increase iron uptake by roots and accumulation in organs, at least for soils with limited iron concentrations [53,54]. Significant differences have been found in iron content between basal leaves and middle and upper leaves (higher levels in the former than in the latter) (Figure S17), but no significant difference has been established between concentrations in the middle and upper ones.

K. D. Rode *et al.* (2003) [55] provided—*inter alia*—comparative data for petiole, blade or whole leaf for seven species (Figure S18). In four of five species of this dataset, the petiole was richer in iron than the whole leaf and, for one species, its iron content was inferior to the leaf; for the two species for which the blade was compared with the whole leaf (with no direct data available for the petiole, however), the latter had lower iron levels than the former (suggesting that, in these two species, the petiole might also have lower iron contents than the blade). 

Iron concentration in shoots is significantly lower than in roots (*p* < 0.001, nonparametric Tukey-type contrast), aerial parts (*p* < 0.001) and leaves (*p* = 0.026), but does not differ significantly from stems (*p* = 0.432). The median concentration was 91.0 mg/kg (almost half of that measured in leaves and about one fifth of that in roots).

Iron concentration in aerial parts does not differ significantly from roots (*p* < 0.768) or stems (*p* = 0.391), but is higher than in leaves (*p* < 0.001) and shoots (*p* < 0.001). Despite the “statistical significance”, this might be a chance finding, taking into account that the comparison of leaves and stems has found no significant difference between these two main components of the “aerial parts”. The median rank was 1439.8 (higher than the global median rank, 1118.0) and the median concentration 240.0 mg/kg (higher than the median concentration in leaves and about half of the median level in roots).

A paired subset of eight species showed that leaves in five species were richer in iron than flowers, while in the other three flowers had higher levels of iron than leaves (Figure S19) with no statistical difference between flower and leaf iron levels (*p* = 0.742).

Iron fruit concentrations seem to be lower than in root (*p* < 0.001, nonparametric Tukey-type contrast), leaf (*p* < 0.001), stem (*p* = 0.035) and aerial parts (*p* = 0.029). The median iron concentration in fruits is 72.6 mg/kg, less than half of that in the leaves and about a seventh of the amount in the root. Despite these apparent differences in iron content favoring vegetative organs over fruits, a paired subset showed that in about half of the observations (*n* = 15) iron levels were higher in leaves, while in the other half (*n* = 14) iron levels were higher in fruits (Figure S20). The ratio between iron levels in fruits and leaves is not constant, depending on other variables such as the plant development stage, as illustrated by *Phaseolus vulgaris* L.: in stage I (50% of blossoming) there is little difference between leaf and fruit iron content (89 *versus* 68 mg/kg), while in stages II and III, the discrepancy between content levels in the two organs is considerably larger (e.g., in stage II 516.0 mg/kg in leaves *versus* 51 mg/kg in fruits) [45]. In this context, even data coming from the same published reports may actually be derived from different plant individuals at different development stages and thus not fully comparable (if leaves and fruits are not collected simultaneously and from the same individual(s)). 

Iron concentration in seed seems to be lower than in root (*p* < 0.001), leaf (*p* < 0.001) and aerial parts (*p* < 0.001), but no lower than in the whole plant (0.051) or stem (*p* = 0.087), although, for these two latter categories, there is a relatively strong trend that could be confirmed with higher sample sizes. A small paired subset showed lower values in seed than in leaves for six out of seven species; two of these species (*Phaseolus vulgaris* L. and *Rumex obtusifolius* L.) had four data points (paired values) each and were all consistent in this direction. *Celtis gomphophylla* Baker (Ulmaceae) was the only species for which the seed iron concentration was higher than the leaf one: 313.7 mg/kg *versus* 152.5 mg/kg [55] (Figure S21). 

For wood, most data have come from three families: Pinaceae, Cupressaceae, and Fagaceae) (Figure S12k). In wood, iron values are very low, varying between zero (more technically, under detection limit) and 35.0 mg/kg. These very low levels differ significantly from those in any other plant parts (*p* < 0.001).

Bark is among the plant parts with lowest iron concentration, only second to wood in low levels (with significant differences against most other plant parts, except for seeds, for which *p* = 0.119, in which case, however, this lack of significance is more likely a result of low statistical power related to the small number of data points available for bark). The median iron level in bark has been 45.0 mg/kg, about a quarter of the amount in leaves, and about one eleventh of the root median.

With respect to other parts, less commonly used or analyzed (aril, bud, bulb, calyx, false fruit, leaf pulp, *etc.* —Figure S22), iron level data have been available for 64 species belonging to 39 taxonomic families.

## 4. Discussion

### 4.1. Extent of Iron Contents Investigation in Plants

Initially, authors expected to find a large number of plant genera and species examined with regard to iron content, but our semi-systematic investigation has shown that only a small part of the plant kingdom has been explored with regard to iron contents, resulting in a wide knowledge gap in this respect. In terms of genera, leaving Gymnosperms aside (28% at least partially investigated), less than 10% of all plant genera have been analytically probed for iron content (6.05% for ferns and fern allies, 7.00% for angiosperms). At the species level, the scarceness of iron data is still more striking: 4.10% for gymnosperms, 0.52% (0.89% if only species with resolved status are considered) for angiosperms and 0.22% (0.65% of the species with resolved status) for pteridophytes. Admitting the limitations of our research process and assuming the study has missed a double number of publications than collected, the volume of research in the field is still short of covering even 5% of all the species. The large intraspecies variability of iron reported so far (see below) and the fact that, for a number of species, data have been limited to one or two parts (e.g., leaf and root) indicate that, even for the species already investigated, additional data are necessary.

### 4.2. Iron Contents in Different Organs

Iron contents have not been reported with the same frequency for various plant parts in the literature, thus only allowing for speculation on the part of the researcher about the potential reasons for such discrepancies. Of the variety of herbal parts investigated for iron content, leaves are by far the part most widely sampled for analysis. This largest number is probably related to easy access (no digging or climbing ordinarily required for collection) and renewable character (collecting roots or stems may imply the destruction of a plant, while collecting leaves usually does not endanger its life). Root and stem data are more limited than leaf or aerial parts data, probably because roots and stems are less accessible; in the case of trees and shrubs, collecting roots or stems may be intimidating or impractical because of their size, while in the case of ferns or some monocots, collecting roots may be daunting because of their small size and thread-like appearance and the same taxons may be simply devoid of stems or have very reduced ones. The larger number of publications for aerial parts might to a certain extent reflect researcher passivity (not taking the pains to separate each organ), but might also translate concerns for efficient management of limited resources, prompted by investigators’ assumption that a global assay of the aerial parts were sufficient for rough appraisal of the iron level in a certain species, such an approach allowing for increased number of species or specimens evaluated. 

For most plant parts, the majority of the data has referred to dicots and monocots (to a smaller extent), while those for magnoliids, ferns, and lycophytes or gymnosperms have typically been much more limited. This is probably related to the natural proportions of the taxons (the group of Dicots is the largest, Magnoliids and Gymnosperms are relatively small groups in terms of genera), as well as to phytogeography considerations. Similar reasons probably apply—within a certain window of variability - for the life-forms. The majority of plants analyzed for iron contents have been herbs and trees (the former usually more frequent than the latter, except for fruits, where the reverse was true, while wood and bark are virtually not available for herbs).

Iron concentration varies within large limits in various plant organs and the levels are low in most cases. We have been especially interested in maximal values, labeled as outliers from a statistical perspective, but of particular interest from the biological point of view as species and herbal parts very rich in iron. In this area, leaving aside the issue of iron bioavailability, the interest lies in assessing whether a species is truly an iron hyperaccumulator or those results are mere chance findings originating from analytical errors, pollution or other unidentified factors. Publications reporting consistently high values for a certain species (from different regions) would hint to an iron hyperaccumulator, while discrepant values would indicate high variability at best. However, for the large majority of plant species, the number of independent reports is limited to one or two only, precluding such assessments. 

In various organs (root, stem, leaf) very high levels of iron were reported in macrophytes [56] or species sampled from a wetland [57], which would suggest a tendency of such species to hyperaccumulate iron, a hypothesis still in need of more supporting data. All data available for Bryophytes (limited in number) tend to indicate a hyperaccumulating feature; for genus *Hypnum*, data from three different publications (“leaves” [58], aerial parts [59] or whole plant [60]) consistently indicate high iron levels (varying between 701 and 7520 mg/kg).

Although point estimates would suggest that certain plant parts are usually richer in iron than others (e.g., roots seem to be richer than leaves in about two thirds of the cases), no definite pattern may be defined, precluding a prediction of which herbal part will be richer in iron. For instance, in *Colocasia esculenta* (L.) Schott, iron concentration in leaf has been 2056.3 mg/kg, while the same paper reports results about four times lower in roots, namely 547.8 mg/kg [57]. On the contrary, in *Portulaca oleracea* L., the higher concentration of iron was described for the root (121.5 mg/kg), and about four times lower in leaves (33.2 mg/kg) [61]. One definite exception is wood, for which all data indicate very low iron levels; a second possible exception would be bark, for which most data point to low iron amounts. 

Factors responsible for differences in iron amounts distributed in different parts of the plant are only partially understood and a detailed discussion would exceed the scope of this paper. Soil minerals accompanying iron may have an impact on iron distribution within plants, as shown by Co in *Vigna radiata* (L.) R. Wilczek (mung bean) or by Cd in *Brassica napus* L. (rapeseed). Co did not inhibit iron uptake into the roots, but did decrease iron concentration in leaves by about 80% [62]. Similarly, Cd does not affect iron accumulation in roots for rapeseed, but it causes a decline in iron concentration in leaves (as well as in phloem and xylem) [63]. Expression of proteins involved in iron uptake (enzymes involved in iron reduction for nongraminaceous species, proteins involved in phytosiderophore secretion and regulation in grasses, chelators or chaperons involved in long-distance transport of Fe) may also vary in different conditions of environmental stress [64]. The developmental stage of the plant should also affect the distribution of iron among different organs; iron is known to be transported in the developing seed not only from the root but also from leaves or fruits, which is likely to lead to a gradual decrease in leaves or fruits with the increase of concentration in seeds [64,65]. Mutations may affect iron homeostasis in plants as well and it has been shown that, in soils with normal iron contents, plants may develop Fe deficiency or toxicity, depending on mutation targets [66].

### 4.3. Variability of Iron Contents in the Plant World

The heritability of iron concentration in various species seems high (around 60%–70% in three species for which published data have been found [67,68,69]), a relatively large number of genes being apparently involved in iron level regulation [67]. Despite the high heritability suggested by the limited data currently available, a large variability characterizes the distribution of iron in the plant world, among both different species for the same organ and different organs for the same species. Intraspecific variations may be substantial (presumably resulting from genetic, ontogenetic or environmental factors). In the case of *Trifolium subterraneum* L. (Fabaceae) for instance, iron content in the aerial parts has varied in a study of 179 populations from less than 50 mg/kg to more than 450 mg/kg, with a mean value of 171.1 (with a somewhat lower median, about 154.5 mg/kg) [70]. In *Cuminum cyminum* (Apiaceae), seed iron content varied in 20 samples from 190 mg/kg to 1690 mg/kg, with a median of only around 310 mg/kg (and mean around 500 mg/kg, because of skewed distribution) [71]. In other papers, iron contents as low as 129/mg/kg [72] and even 27 mg/kg [73] were reported for the seeds of *Cuminum cyminum*. Concentrations varying between 90 and 510 mg/kg have been reported for the aerial parts of *Centaurium erythraea* Rafn (Gentianaceae) collected from 30 different sites [74]. In spinach leaves, an analysis of various genotypes has shown variations between 156.2 and 235.7 mg/kg [75]. 

### 4.4. Limitations

Our estimations of iron contents in various plant parts and different subsets have been affected by a number of limitations. Firstly, the whole data set comprises iron concentration data for 1228 plant species, representing about 0.35% of the 350,699 species with “accepted” status included in The Plant List and 0.21% of the total number (593,411) of species with “accepted” or “unresolved” status. We could not get access to a small number of papers containing potentially relevant data (five angiosperm and six gymnosperm genera). Despite the randomization applied in the selection of the papers used for data extraction, several sources of bias might distort the representativity and generalizability of estimations for the true global population. As discussed above, for the genus keywords used to define the data set only a minority returned results and most of the species were included in the data set by co-occurrence with other genera used as a keyword or as false positive results for a specific genus. The large intraspecies variability, the influence of various external factors (such as pollution, soil, climate, *etc.*) may also have affected some of the results included in our computations. Moreover, in the case of papers analyzing larger number of samples/accessions, we have limited the extraction process to the minimum and maximum values from each paper, except where the influence of different variables on iron uptake was investigated as part of an experiment. For certain species, several papers with different values have been published, while for others a single paper has been made available, a fact introducing a degree of heterogeneity within the data set. For sensitivity analysis purposes a subset has been defined where, for each species, the data have been limited to the minimum and maximum value (irrespective of both the number of publications available for that species and the experimental factors) statistical parameters changing little as compared with the global dataset, suggesting that the results are somewhat robust in their main findings.

Although our investigation was started with statistical power calculations, these were based on certain assumptions (as described in the Materials and methods section) and related to data available for at least one plant part. To ensure meaningfulness of comparisons among different taxonomic groups or different life-forms the same parts (organs) have been compared; however, whereas for leaves, aerial parts or fruits, the number of species (and of data points) was 200 or larger, for other plant parts, the sample size (in terms of number of species) was smaller, many of the comparisons, although prespecified in our protocol, thus remaining exploratory and affected by limited statistical power. One should always bear in mind that large effects may be detected even with small(er) sample sizes, while small effects need very large sample sizes, which helps to put various findings into context; however, small to modest effects seem to be more widespread than large ones and may be important [38], pointing to the need for similar analyses on larger samples in the future. 

Finally, the manner of appraising the papers included in our review may have been biased by the views of the first evaluator, but our initial assessment has indicated a very small number of papers possibly affected by this bias.

### 4.5. Absorption of Iron of Plant Origin in Humans

The review we have undertaken of iron contents in a variety of plant species has shown large variations, with examples of both very low and very large iron amounts. Although many species included in our review are used to a limited extent (or not at all) for human nutrition purposes, there is a growing tendency of using a variety of plants as food supplements or as new foods within a “return to nature” fashion. The potential usefulness of iron from these herbal sources is dictated by its availability: a large amount of iron, if not available for the needs of the organism (e.g., because of a form inappropriate for absorption) would only be of a theoretical interest for scientists and of no interest for the general patient. Measuring iron bioavailability in humans is fraught with methodological difficulties [76], which may explain the limited number of high-quality studies in this field and the issues still unresolved in understanding iron absorption and bioavailability, but a number of aspects have been clarified.

Our Pubmed search for papers on the absorption of non-heme iron from plants returned 1392 papers in total, of which, reviews left aside, 382 were found to be relevant (even if marginally so). Of these, 91 reported on *in vitro* experiments, 100 on nonclinical investigations and 185 reported on iron absorption in humans from interventional or observational studies; six additional papers reported on iron absorption from transgenic plants designed to increase iron uptake from soil and have lower contents in phytochemical inhibitors of iron bioavailability (e.g., phytate). For editorial space constraints, the discussion here will be limited to a summary only, the authors planning on a full review in a separate paper.

The literature under scrutiny has shown that it is classical knowledge that heme-iron is better absorbed than non-heme-iron (such as iron of herbal origin) [77,78,79]. Although this notion was mainly established by long past studies (carried out mostly between the 1960s and 1980s), most of those investigations used radiolabelled heme or hemoglobin (accurate methods) and were largely consistent in showing better absorption of heme over non-heme iron [80,81,82] (although initially the contrary was believed to be true and one study did report better absorption of inorganic iron over hemoglobin [83]) and considerably less inhibitory effects of other food components [81]. In this context, using plants as a source of iron would seem not the best option. However, commercially available iron-containing food supplements also contain non-hem iron. In the case of non-heme iron, although lower, absorption is not necessarily exceedingly low but rather variable, influenced by a large variety of factors, with either favorable or unfavorable effects on iron absorption (most of which have already been known for several decades) [84]. In a nonclinical, parallel group study comparing absorption of iron from several genotypes of maize with that of ferrous sulfate, no significant differences were found in terms in biological effects on Hb between maize and ferrous sulfate [85]. In an experiment involving 2-week supplementation in rats, no significant difference was seen in several hemoglobin parameters (hemoglobin concentration, mean corpuscular volume, mean corpuscular hemoglobin, and mean corpuscular hemoglobin concentration) between soybean sprouts enriched in ferritin (by germination in a FeSO4 solution), ferritin isolate, control ferrous sulfate and a control group of healthy animals [86]. A nonsignificant difference in hematologic indices was reported in an *in vivo*, parallel group experiment comparing a leaf extract of *Telfairia occidentalis,* ferrous sulfate and a control group fed on an iron-deficient diet with no treatment [87] (careful examination of the data, however, suggest that this was mainly the results of insufficient statistical power, the effect of the extract being about half of that seen with the inorganic salt). Nevertheless, this experiment does show that increase of the extract dosage allows for acceptable levels of Hb and that iron administered from herbal sources may be sufficiently bioavailable). In a Caco-2 cell model, it has been shown that addition of cassava to a cereal homemade recipe may significantly increase ferritin formation, from 36.74 to 67.58 ng/mg [88]. Dietary sulfur amino-acids, as provided by a diet rich in shallot (*Allium ascalonicum*) and leek (*Allium tuberosum*) were shown *in vitro* to increase the iron availability of cereals and pulses with 10%–67% and 10%–38%, respectively [89]. A mathematical modeling based on data on iron absorption from a rice-based meal in Indian female subjects with iron deficiency and iron deficiency anemia has predicted that iron intakes of 20–55 mg per day are sufficient in (plant based) low-bioavailability diets to ensure stable, non-anemic levels of Hb in women [90].

On the other hand, experimental results as the above have to be interpreted with much caution, given that they are not derived from clinical settings but rather from nonclinical experiments and should be considered only as hypothesis-generating. The Caco-2 cell model has been able to predict human iron absorption from maize, but not from beans, and the predictions were accurate only from a qualitative point of view (*i.e.*, indicating direction of differences) [91]. From many herbal sources, iron seems to be absorbed only to a very limited extent. For instance, it has been reported that only about 2% of the total iron was absorbed in women from maize or bean meals [91]. Depending on the phytochemical matrix accompanying the iron in various plant sources, iron uptake by the human body may be more or less effective. The number of variables related to the food matrix influencing non-heme iron absorption is impressive and our study provides a synthetic overview for the most important ones (Table 4). In this overview, subject-related factors such as iron and nutritional status, infection, inflammation, genetic disorders, *etc.,* have been left aside. [78].

**Table 4:** Food-matrix related factors affecting absorption of iron of plant origin.

Factor	Nature of Evidence	Comments
Phytic acid, phytates (from the plant source itself or from other foods, e.g., cereal bran)	*In vitro*, nonclinical, clinical (interventional)	It is the most studied factor influencing non-heme iron uptake, with very robust supporting evidence (e.g., [92,93,94,95,96,97,98,99,100].
Polyphenols (from the plant source itself or from other food).	*In vitro*, nonclinical, clinical (interventional)	There is convincing evidence that polyphenols may interfere with human iron uptake from food, but not all polyphenols “are created equal” and as yet there is no complete picture of their effects, especially in the presence of other phytochemicals. In the case of cowpea (*Vigna unguiculata* (L.) Walp.) flour fortified with iron, there was no difference in iron absorption between a variety with low polyphenol content and one with high polyphenol content. The authors speculated that the reason might be that both had a similar phytate:iron ratio, which might be much more relevant for iron uptake than the concentration of polyphenols [96].
Tannins (from the plant source itself or from other food).	*In vitro*, nonclinical, clinical (interventional)	Animal data (rat) and *in vitro* studies support an inhibitory role of tannins on iron uptake. 500 mg tannic acid added to a broccoli meal significantly decreased iron uptake (geometric mean 0.015 *versus* 0.297) [101]. It is also speculated that the inhibitory effect of green tea is related to its tannin and polyphenol contents [102]. But the matrix remains essential: although brown rice has significantly higher levels of tannic acid and phytate than milled rice, in a study in healthy adults no significant difference was identified in the amount of iron absorbed from the two types of rice [103].
Green tea	Nonclinical (rat), Clinical (interventional)	A clinical trial [104] and several rat studies suggest that tea (through its polyphenols and aluminum) dose-dependently decrease iron uptake. An observational study in humans found no effect of black, green or herbal tea on serum ferritin or the iron depletion risk [105] (limitations of observational studies have to be taken into account, however).
Chilli	Clinical (observational, interventional)	An interventional study carried out in women [106] and an observational study in subjects of both genders found an inhibitory effect in female, but not in male chili consumers [107]. Unlike for humans, data in rats suggested that capsaicin (similarly to piperine or ginger) increases iron absorption [108].
Iron mineral competitors	Nonclinical, clinical	Fe, Zn and Ca may interact with each other, reciprocally decreasing their bioavailability [109]. The effect of Zn is perceptible only when present in high levels (from 90 mg/kg upwards) [110]. Initially contradictory results have been published con calcium, some suggesting that calcium supplementation would negatively influence iron absorption [111,112], while more recent several long-term intervention studies have shown that long-term use of calcium supplements by post-menopausal women does not affect iron status. It has been suggested that iron absorption might be perturbed only on short-term, while on long term adaptive responses of the body might reestablish the iron balance [113].
Certain fruits (orange, guava, kiwi)	Clinical (interventional)	The increase in uptake might be related to the ascorbic acid and possibly beta-carotene contents [114].
Ascorbic acid	*In vitro*, nonclinical, clinical (interventional)	There is convincing evidence that ascorbic acid facilitates iron uptake, it may partially offset the negative effects of phytates [115] and of small amounts of polyphenols [116], but not the inhibitory effects of tannins [115] or of high amounts of polyphenols (as indicated by *in vitro* data) [116]. It seems more effective than EDTA [117] but has little effect in the presence of the former [93].
Other organic acids (citric, erythorbic, malic, tartaric, succinic, fumaric, aminoacids, especially cysteine)	*In vitro*, nonclinical, clinical	The evidence is scarcer and less robust than for ascorbic acid and these organic acids seem substantially less effective than vitamin C [118]. Contradictory evidence exists for lactic and oxalic acids (no effect in some studies [119,120,121], positive effect in others [122,123,124], a slightly inhibitory effect in a study for oxalic acid [101]).
EDTA	Clinical (interventional)	EDTA facilitates iron absorption. Its effects seem inferior to those of the ascorbic acid [117], but the body of research is less extensive for EDTA.
Iron amount	Nonclinical, clinical	Although the relationship is nonlinear, there is relatively robust evidence that higher iron intake leads to higher (but not proportionally so) absorption [125].
Meat protein (beef, fish and chicken)	*In vitro*, nonclinical, clinical	There is a relatively large body of evidence that animal protein (the so-called “meat factor”) favors iron uptake [126]. In a study, egg protein had a moderate inhibitory effect [127]. Several decades ago it was estimated that 30 grams of meat, poultry or fish are roughly equivalent to 25 mg ascorbic acid [128].There are quantitative differences among different meat sources (animal species) [129,130]. There are also differences in the effect of proteins on absorption in humans and rodents [131]. Plant-derived proteins have either no effect [132] or variable effects (most often inhibitory [133], rarely facilitatory [134]) depending on source and properties. *In vitro* data indicate that high molecular proteins have a better influence on iron absorption than low molecular weight proteins, irrespective of the source (animal, plant) [135], but the relevance of *in vitro* data for the clinical context is limited.
Plant species or variety	Nonclinical, *in vitro*	Different plant species (of the same genus even [136]) and different varieties of the same species [137] have different effects on iron uptake, probably depending on their phytochemical matrices.
Prebiotics	*In vitro*, nonclinical, clinical	Different prebiotics seem to have different effects on iron metabolism and uptake [138,139]. There are also discrepancies between the *in vitro* and *in vivo* data (the latter are more relevant to the clinical context) [140,141].
Probiotics	*In vitro*, clinical (interventional)	Although theoretical mechanisms have been proposed by which various bacterial species might increase iron absorption, limited *in vitro* data show that this is not always the case [100,142], the bacterial species being also relevant. Certain lactic fermentation bacteria have been shown to facilitate iron absorption [119,140,143] and it seems that the effect is not due to lactic acid [119].
Fructose	Nonclinical, clinical	In one clinical trial fructose (but not high fructose corn syrup) was reported to favor iron excretion and diminish the iron balance, probably by the induced diarrhea and consecutive lower absorption [144]. Nonclinical data (*in vitro* and in rats) claimed both increased [145] and limited [146] iron absorption.
Fructo-oligosaccharides	Nonclinical, clinical	Negative [147], neutral [148] and positive effects [149,150] have been recorded in various nonclinical experiments with fructooligosaccharides. In a clinical trial, no significant difference was observed *versus* the control group [151].
l-alpha-glycerophosphocholine	Clinical	Identified in one study as the so-called “meat factor” and claimed to have improved iron absorption [126], in a different study it did not seem to influence iron uptake [93].
Iron source (salt, complex)	*In vitro*, nonclinical, clinical	A number of studies have shown that iron is absorbed differently from various salts (e.g., NaFeEDTA is better absorbed from fortified soy sauce than FeSO_4_ [152]). Ion chelates with aminoacids (e.g., glycine) seem to be subject to less influence from inhibitors and enhancers than ferrous sulfate [153].
Food cooking/processing (boiling, roasting, decortication, germination, fermentation, *etc.*).	*In vitro*	Various processing methods have different impacts on iron uptake (increase, decrease or no influence on availability), depending on the nature of treatment and of the food [154,155,156,157,158,159,160,161].
Vitamin A, beta-carotene	Clinical, nonclinical	In a clinical experiment, vitamin A increased iron absorption from rice up to twofold, 0.8-fold from wheat (*i.e.*, it caused a slight decrease in absorption) and 1.4-fold from corn. Beta-carotene increased iron absorption more than 3 times for rice and 1.8-fold for wheat and corn [162]. In a rat study, carotene was claimed to hinder iron absorption [163]. Clinical data should be considered more relevant and thus it is likely that carotene rather increases absorption.

The negative effects of some of the inhibitor factors are more or less offset by the positive ones of those favoring absorption; for instance, the effects of polyphenols [164] or phytic acid [165] seem to be counterbalanced by the positive effects of ascorbic acid. But the array of factors influencing iron absorption is considerably larger, as illustrated by the fact that in a study in humans, three main variables (animal tissue, phytic acid, and vitamin C) could only explain about 16% of the variability seen in absorption (in a multiple regression statistical model) [166]. Similarly to what has been reported for long-term use of calcium, multimeal studies containing a plurality of absorption inhibitors and enhancers seem to show a more modest effect for the inhibitors (which is to be expected, considering the large number of variables with conflicting effects) [78,167,168].

A few years ago the statement was made that “there are already publications dealing with total element concentrations in medicinal plants, but only a few investigations deal with more detailed information on the forms of these elements, for example, availability of metals by using different extractants, or correlation of extracted metals with total amounts of organic substances” [169]. While separate data usually exist for a certain herbal product regarding its iron and polyphenolic or tannin contents, they in most cases come from different studies; therefore, little is known about the potential correlations between the two and it is difficult to appreciate the real levels of the two in the same sample, as they rarely have been assessed in the same samples (to allow for firm conclusions). For instance, the iron chelating capacity of polyphenols from *Cuminum cyminum* L. seeds has been experimentally tested, but polyphenols were not assayed simultaneously with the iron in the seeds [170]. The iron contents of these seeds may vary substantially and it would have been interesting to know to what extent a higher content in iron correlates with a higher or lower content in polyphenols. The situation is similar for *Betula pendula* (Betulaceae) leaves [171], fruits of *Crataegus pentagyna* subsp. *Elburensis* (Rosaceae) [172], roots, stems and leaves of *Raphanus sativus* L. (Brassicaceae) [173,174], rice bran [175] and other herbal products [176,177,178]. In one of the few studies performed in this sense, it has been reported that calyces of *Hibiscus sabdariffa* L. (accompanying the fruits) have a relatively high iron content (800–833 mg/kg) and only traces of tannins and phytic acid [179]. A few other studies have tested specifically the effects of polyphenols or other phytochemicals from algae [180], beans [98,156,161,181], cereals [182,183,184,185], nuts [186], a few herbal teas [187] or other plant products [188] on iron bioavailability, but have not simultaneously measured the amounts of iron and polyphenols (and possible correlations thereof). A few such studies where iron and other trace elements were measured simultaneously with polyphenols in a few plant species have been published so far, but they were not specifically focused on investigating the relationship between the two chemical entities (and have not tested correlations between them) [189,190,191,192,193,194]. It should also be born in mind that not all polyphenols have the same iron-chelation properties, only a weak correlation (*r* = 0.40) was found in an experimental study between phenolic and flavonoids contents and iron chelating activity [195]. Kaempferol was indicated as a strong inhibitor of iron uptake by Caco-2 cells in 2008 [161], but no study since then seems to have investigated its content in relationship with iron.

Similarly to iron content, the heritability of iron bioavailability also seems high (10 quantitative trait loci have been identified in maize, explaining about half of the variance recorded for samples from a single time period and location) [67]. A study has investigated 15 rice genotypes and found that lower iron bioavailability ones tended to be darker in color [183].

**Figure 4:**
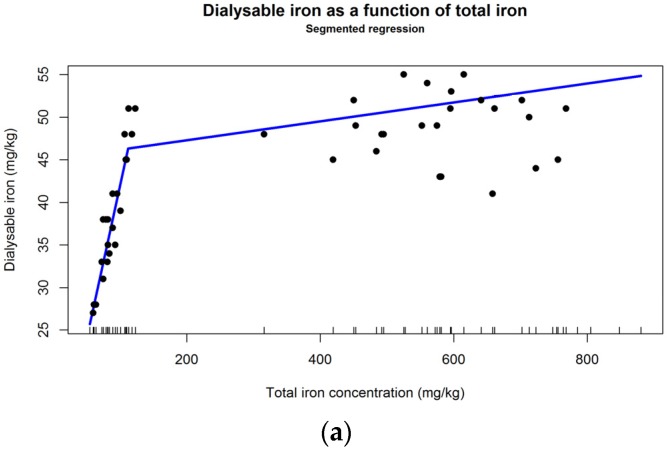

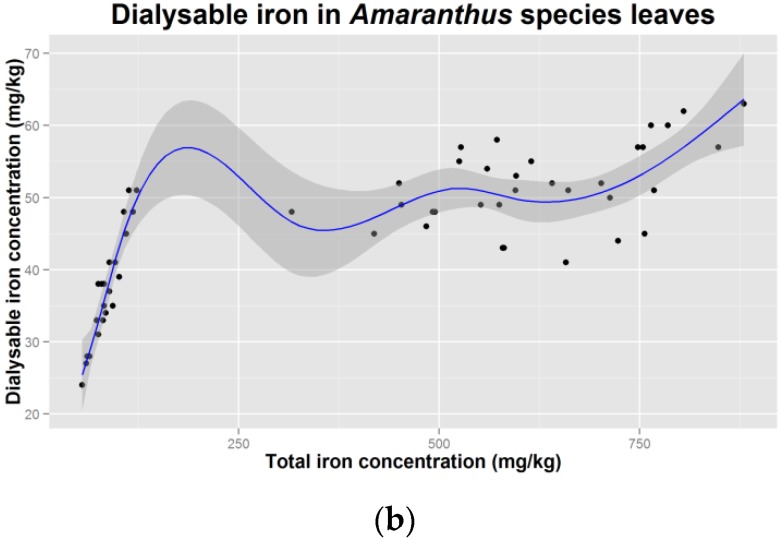
(**a**) Segmented regression modeling of dialyzable iron in *Amaranthus* species leaves as a function of total iron in the herbal product; (**b**) natural cubic spline modeling of the same data. Based on [71].

Little interest has been shown so far for the chemical forms in which iron is present in plant tissues and the influence these forms might have on iron absorption. Rangarajan and JF Kelly (1999) [51] have investigated the relationship between total iron and dialyzable iron in a set of 12 *Amaranthus* species, grown in both open field and in greenhouse, finding that despite a considerable increase in total concentration, the incremental concentration of dialyzable iron was very modest. We applied a segmented regression to these data, modeling the dialyzable iron concentration as a function of total iron. Two segments have been identified, with a breakpoint around 112 mg/kg (total iron concentration) and obviously different slopes: the slope for the second segment is about 32 times smaller than for the first one (lower concentrations) (Figure 4A). In other words, in this study for total iron concentrations higher than 112 mg/kg the gain in dialyzable iron was very minor, compared with total iron levels lower than 112 mg/kg, for which the slope was considerably steeper. This would suggest that total iron levels higher than 110–125 mg/kg have little contribution to improving iron absorption. Nonlinear modeling of the same data using natural cubic splines (with seven degrees of freedom) were broadly in line with the segmented model (Figure 4B). It is difficult to estimate the relevance of these data for other herbal sources though, not only because different sources have dissimilar phytochemical matrices with potentially different influence on iron absorption, but also because of potential confounders in the modeled data, the two segments corresponding to values derived from plants grown in different conditions: the plants with low total iron levels were grown in a greenhouse environment, while high total iron ones were grown in open field; in addition, there were small differences in the growth conditions and timing of harvesting between the two sets. Moreover, as the dialysability was assessed *in vitro*, the *in vivo* understanding of the significance of this finding is complicated by additional uncertainty. Thirty years ago, a good correlation between the *in vitro* and *in vivo* availability data was claimed [196], only to be shown later that, with respect to factors affecting iron absorption and especially inhibitors in the meal, such methods may be less accurate than initially expected [197]. 

The same group of researchers used a comparative approach to the bioavailability of iron from three different *Amaranthus* species in anemic rats and reported that when the concentration of Fe in the *Amaranthus* diet was the same, iron in *A. hypochondriacus* was more bioavailable than that of *A. tricolor* [198]; the latter contains more iron than the former, but the authors limited their research to comparable iron levels. It is not clear, therefore, whether the increased iron contents of *A. tricolor* finally leads to better absorption. More research is therefore needed on comparative iron bioavailability from various species, as the iron content *per se* is not conclusive in this direction.

Our focus has left out efforts spent on biofortification, development of foods with increased iron content and availability, such as maize [67], beans [199], bananas, *etc.* [200].

In theory plants with high contents of iron (the richest parts, more specifically) would be a solution for the prevention of iron-deficiency anemia, either to be used as such in human nutrition or after processing and incorporation in appropriate form as food supplements. Assessment of the usefulness of such an approach is complicated by the complex matrix of variables surrounding iron absorption from plant sources. Increasing iron contents may associate with an improvement in absorption up to a certain threshold, but as suggested by dialyzable iron in *Amaranthus* leaves, very high levels may not translate into any additional benefit. Limited clinical data also support this notion, as it has been reported that, in young women, a higher proportion of iron-rich leafy vegetables did not lead to increased iron absorption, presumably because the larger amount of leafy vegetables also contributed larger amounts of inhibitory polyphenols [201]. It is recognized that the most important determinant of iron bioavailability is the subject’s iron status (and not the iron amount in the food), but ensuring sufficient amounts of iron in foods of herbal origin should probably be preferable to providing limited amounts, especially in vegetarians (for whom iron bioavailability factors have been estimated to be 5%–12% [78]).

### 4.6. Could Iron of Herbal Origin Be a Meaningful Supplementation Option?

Iron supplementation remains a necessary measure in preventing iron-deficiency anemia and, in contemporary society, there is an increasing tendency to use products of “natural” origin and herbal food supplements, while rejecting “synthetic” medicines and food supplements. Most iron-containing food supplements on various national markets use iron salts (of chemical origin) and not ”biological“ iron. Conventional oral iron-containing food supplements may (and often do) cause unpleasant gastro-intestinal side-effects, mainly constipation or diarrhea, heartburn, nausea and abdominal cramps. They often lead to changes in stool color, which may make users worry, but this is not a real adverse effect [202]. It is not impossible that these effects might be related to the nature of iron used in these supplements—most often pure inorganic or organic iron salts. One might speculate that natural extracts rich in iron might be exempt from such effects. This assumption would be supported by the fact that normal diet with appropriate iron intakes does not lead to such effects and also by the well-accepted finding that taking iron supplements with food (and not on an empty stomach), considerably diminishes the likelihood of their occurrence [202]. This remains a pure conjecture, however, and evidence is needed to confirm or reject such a hypothesis.

## 5. Conclusions

The examination of iron amounts in different organs or parts of over 1000 plant species has shown very large inter- and intra-species variations**,** with few detectable patterns, if any. Iron content seems to be highest in roots, lower in green organs (leaves, stems, aerial parts), still lower in fruits and seeds and lowest in bark and wood. Nevertheless, except for bark and wood (with negligible levels for all practical purposes), no *a priori* determination of the part with the highest iron level is possible for a particular species. No particular life-form (herb, tree, shrub, subshrub, vine) seems particularly associated with higher amounts of iron.

Heme may be disallowed by certain persons as a source of iron for religious, personal, or food safety considerations [203] and thus there remains an interest for food supplements containing iron of herbal origin. Some manufacturers have formulated herbal food supplements intended for iron supplementation. Because the available data suggest that iron of herbal origin tends to be less bioavailable (although theoretically, speculatively speaking, it might have better safety or at least better acceptance for some consumers), such formulations have to be based on judicious selection of herbal ingredients, so as to be relatively high in iron content, low in the content of natural absorption inhibitors (such as polyphenols, tannins or phytic acid) and high in the content of phytochemicals favoring iron absorption (such as ascorbic and other carboxylic acids, vitamin A or beta-carotene). This needs to be backed-up by high-quality research simultaneously investigating the respective contents in at least several of these phytochemicals, but such research still primarily remains a task for the future.

## Supplementary Materials

The following are available online at http://www.mdpi.com/2072-6643/7/12/5535/s1.
