# Supplementary material for: A Survey of Plant Iron Content—A Semi-Systematic Review
Source: Nutrients. 2015 Dec 10;7(12):10320–51. doi: 10.3390/nu7125535 (PMC4690087; doi:10.3390/nu7125535)
Supplement: Supplementary file 1 [file nutrients-07-05535-s001.docx]

Supplementary Materials: A Survey of Plant Iron Content—A Semi-Systematic Review

Robert Ancuceanu, Mihaela Dinu, Marilena Viorica Hovaneţ, Adriana Iuliana Anghel,
Carmen Violeta Popescu and Simona Negreş

| 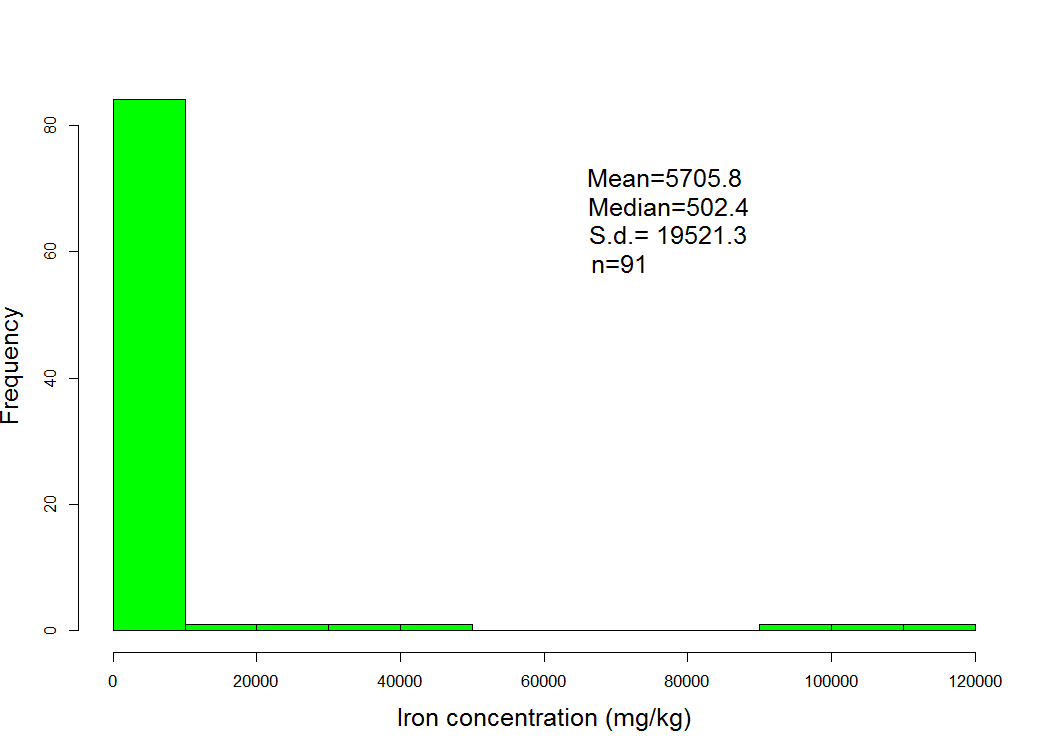 |
| --- |
| (**a**) |
| 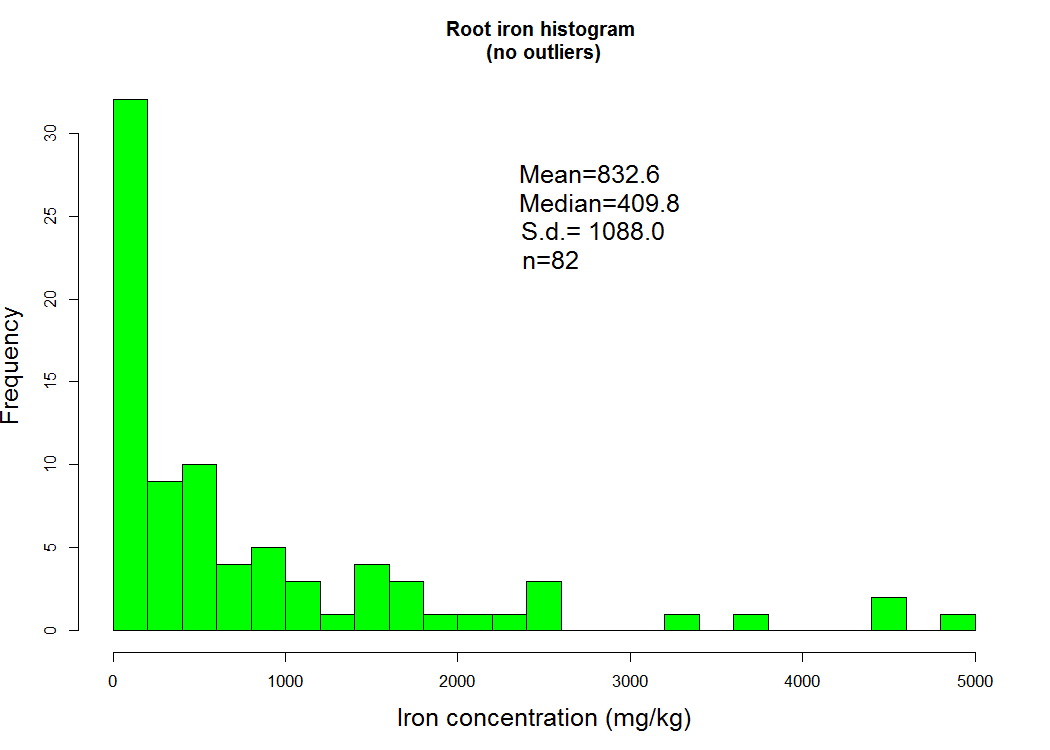 |
| (**b**) |

**Figure S1.** Histograms of root iron concentrations. (**a**) with all outliers; (**b**) with the exclusion of values larger than 5000 mg/kg.

| 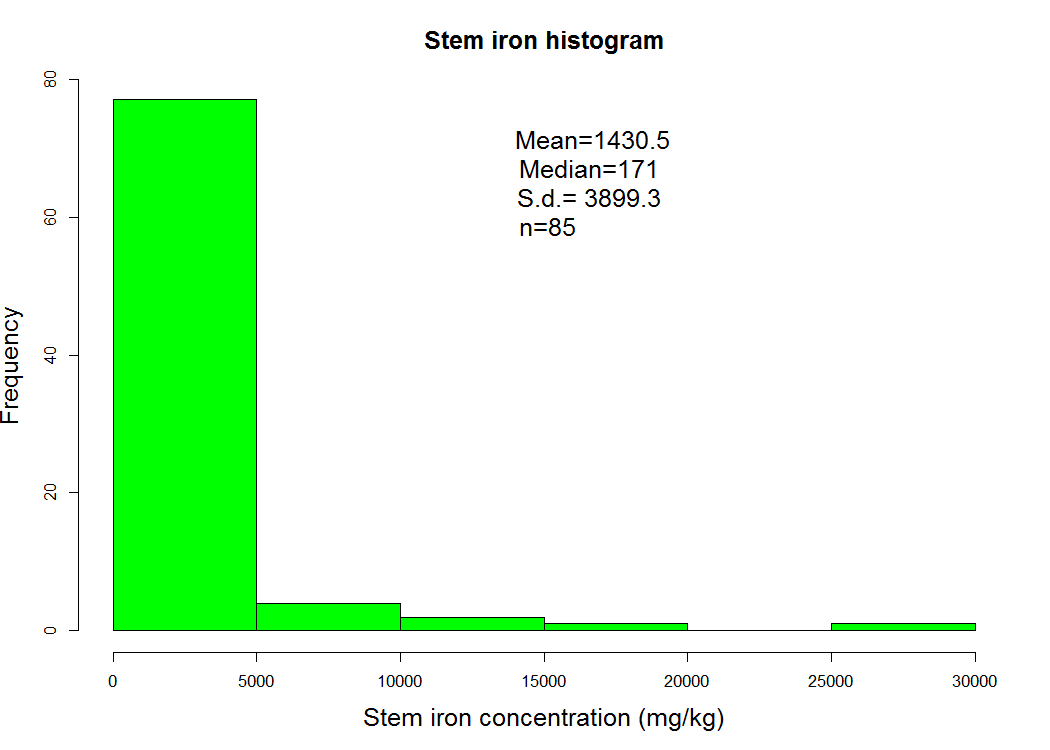 |
| --- |
| (**a**) |
| 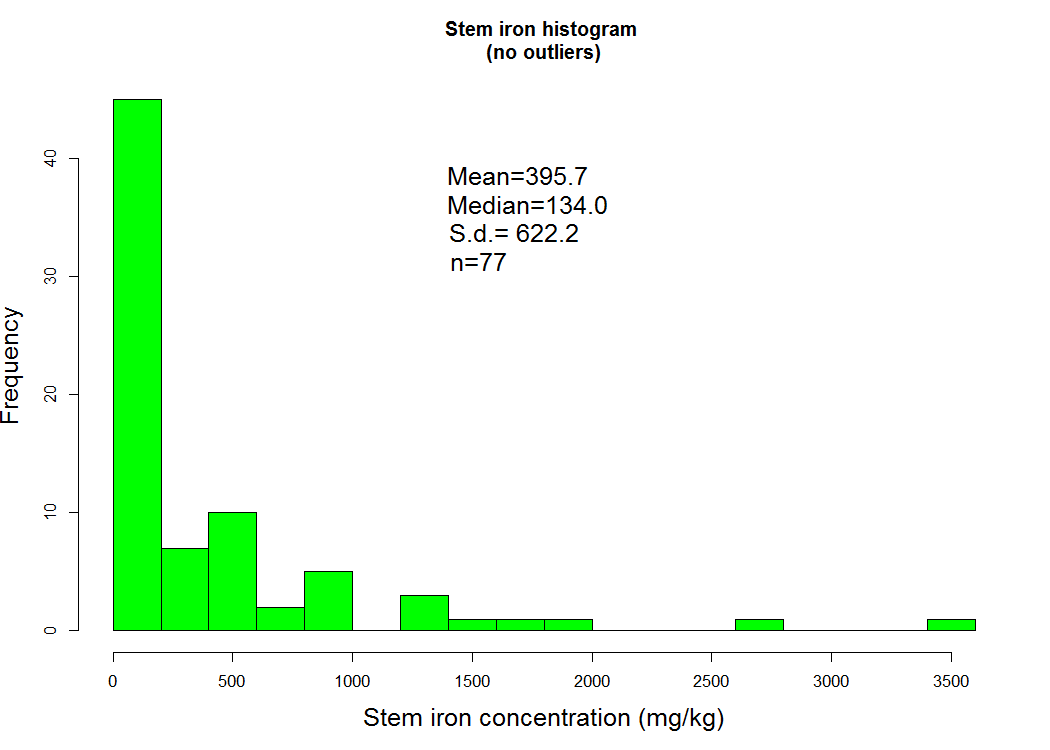 |
| (**b**) |

**Figure S2.** Histograms of stem iron concentrations. (**a**) with all outliers; (**b**) with the exclusion of values larger than 5000 mg/kg.


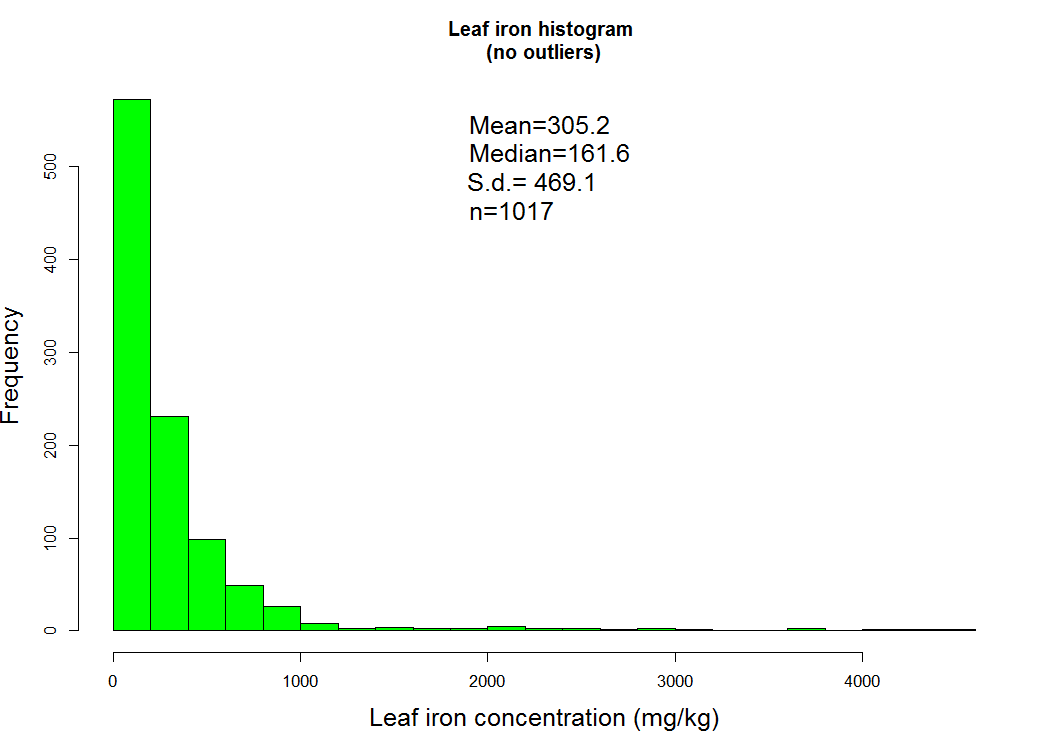


**Figure S3.** Histogram of leaf iron concentrations (with the exclusion of values larger than
5000 mg/kg).

| 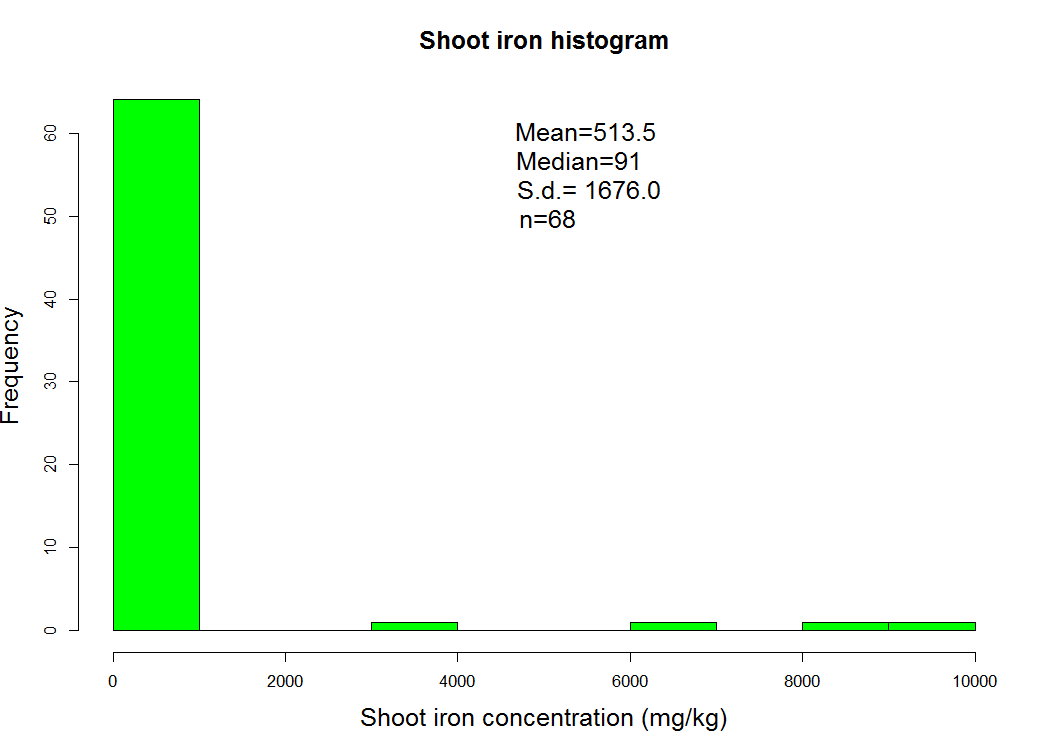 |
| --- |
| (**a**) |
| 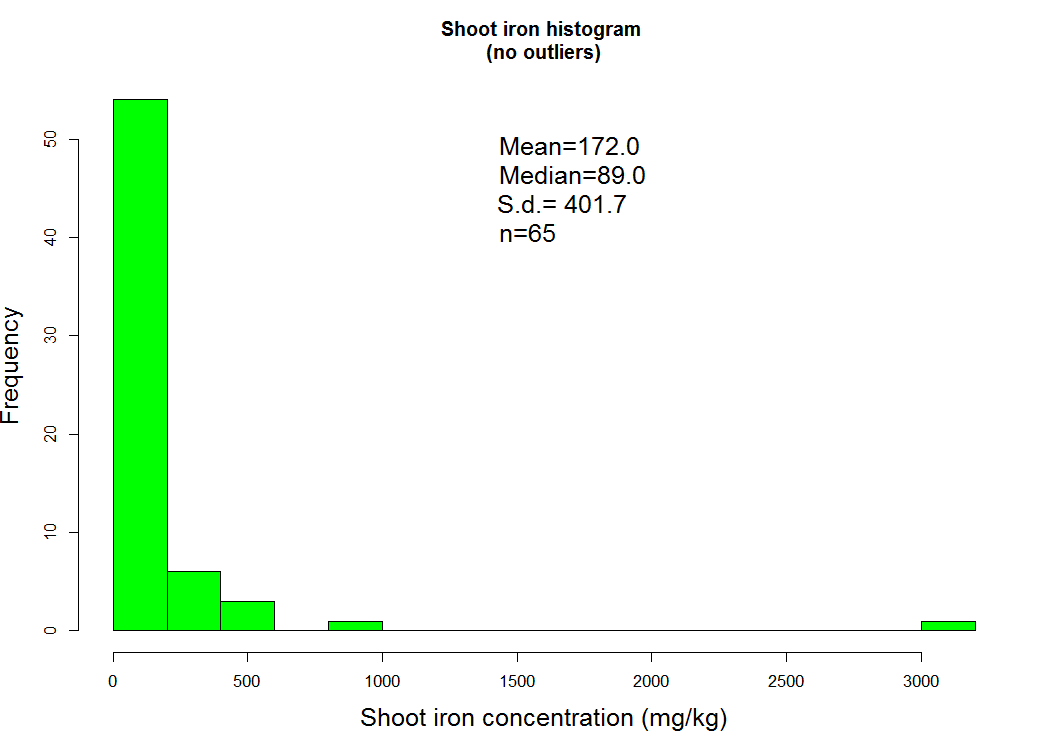 |
| (**b**) |

**Figure S4.** Histograms of shoot iron concentrations. (**a**) with all outliers; (**b**) with the exclusion of values larger than 5000 mg/kg.

| 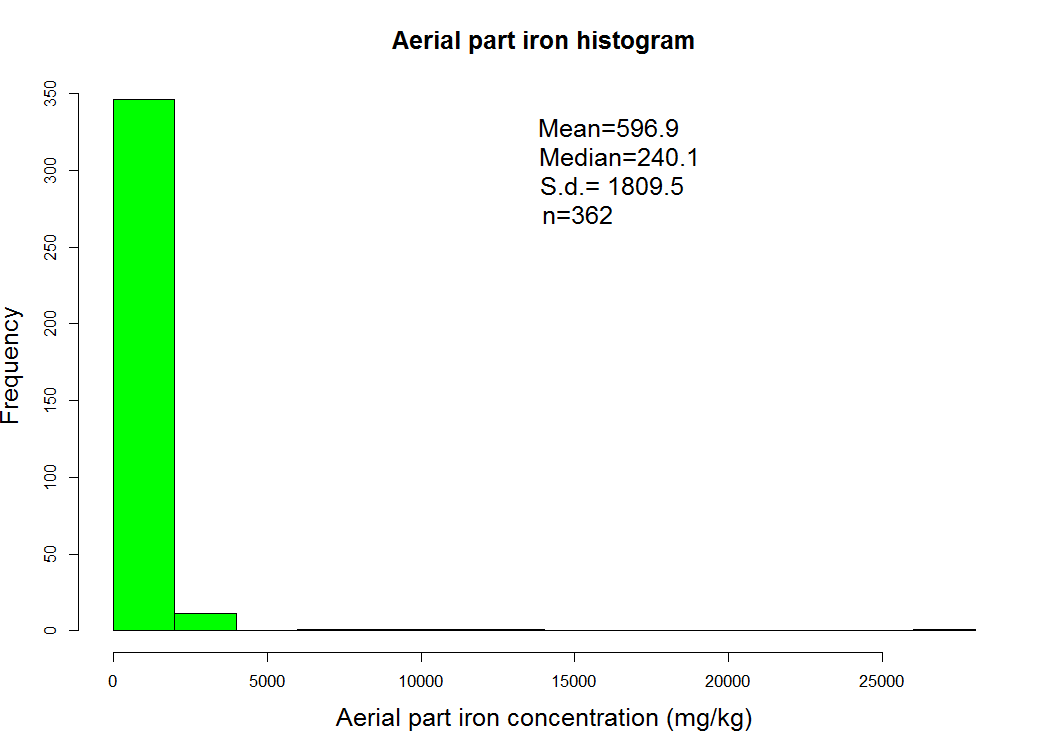 |
| --- |
| (**a**) |
| 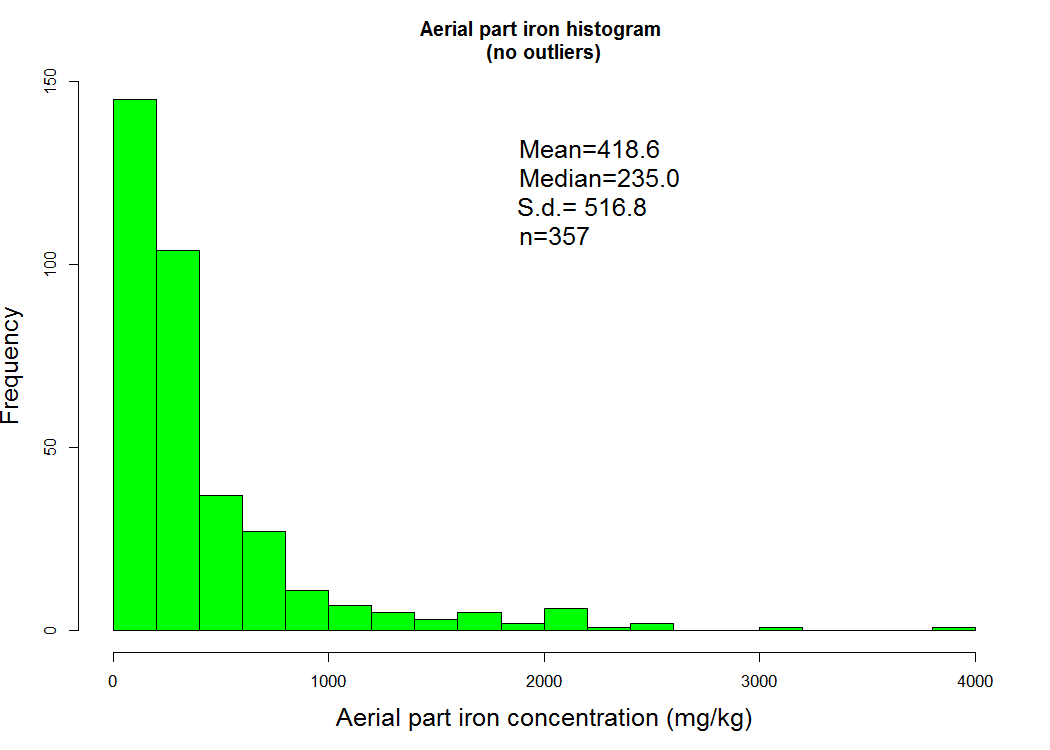 |
| (**b**) |

**Figure S5.** Histograms of aerial part iron concentrations. (**a**) with all outliers; (**b**) with the exclusion of values larger than 5000 mg/kg.

| 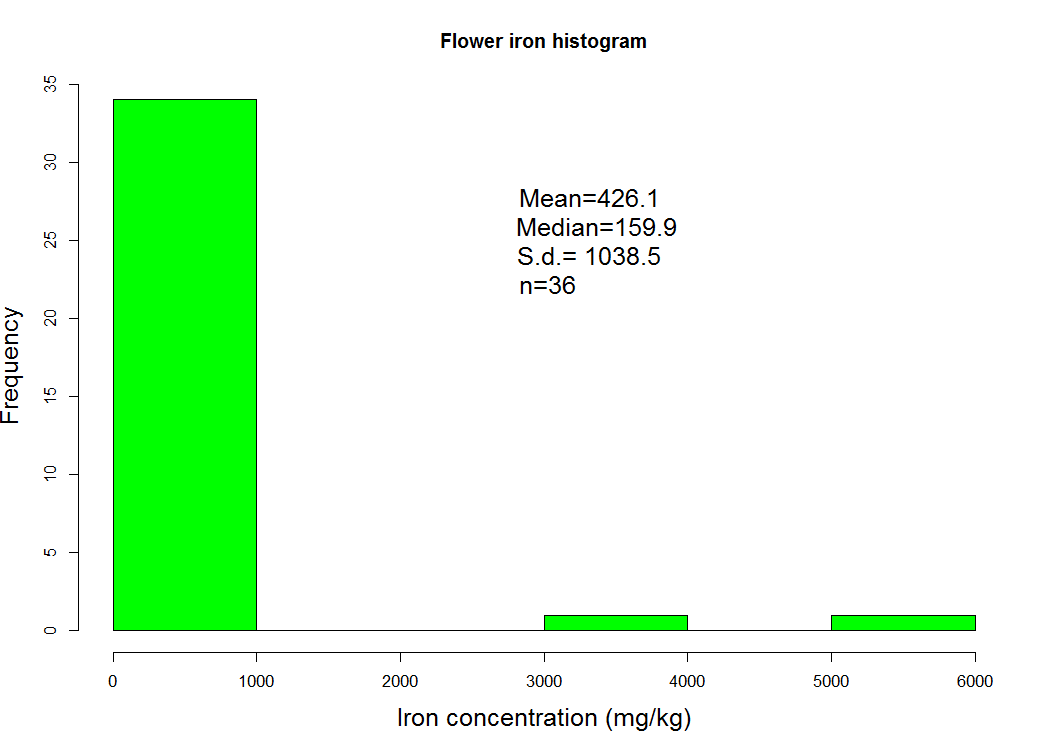 |
| --- |
| (**a**) |
| 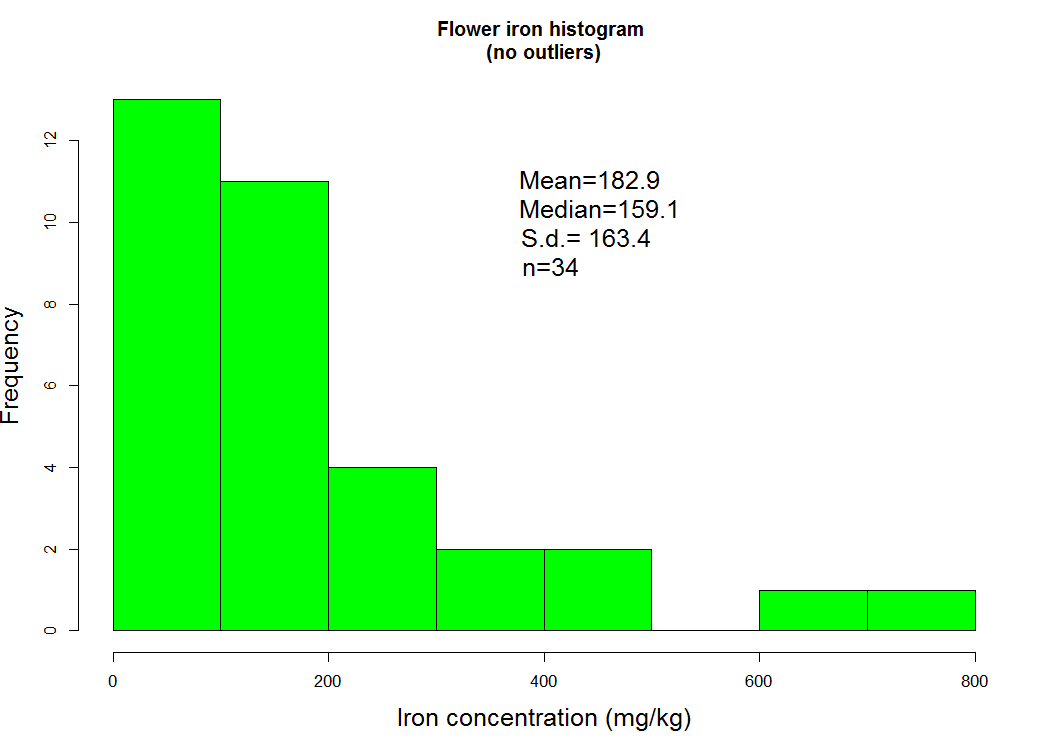 |
| (**b**) |

**Figure S6.** Histograms of flower iron concentrations. (**a**) with all outliers; (**b**) with the exclusion of values larger than 5000 mg/kg.

| 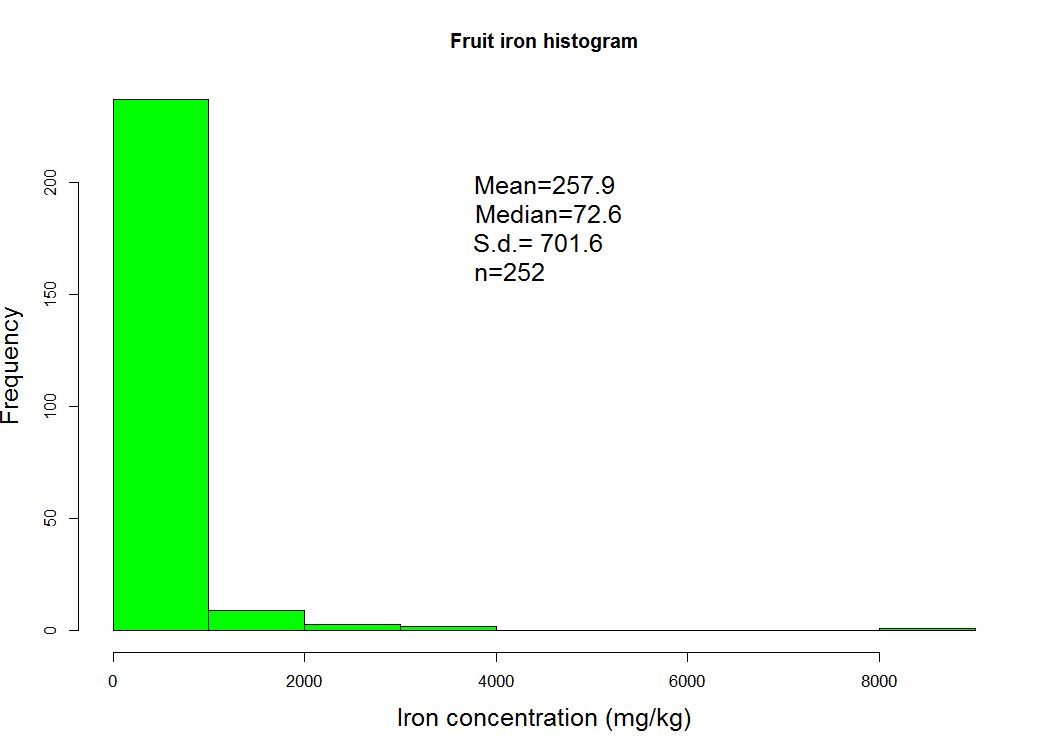 |
| --- |
| (**a**) |
| 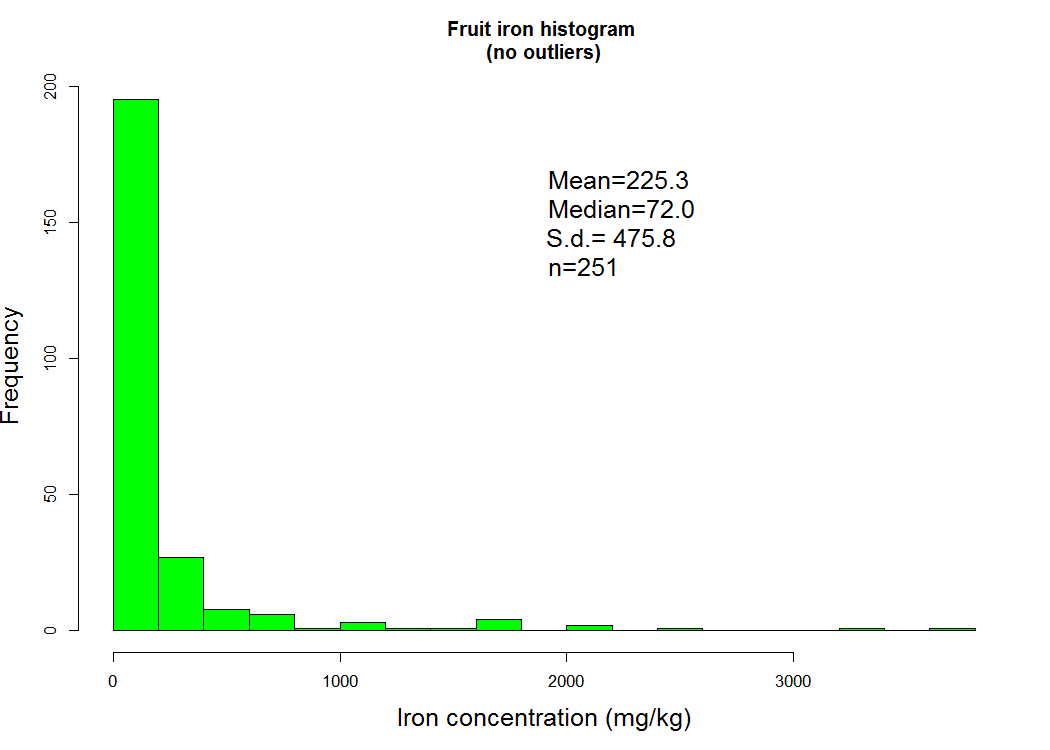 |
| (**b**) |

**Figure S7.** Histograms of fruit iron concentrations. (**a**) with all outliers; (**b**) with the exclusion of values larger than 5000 mg/kg.

| 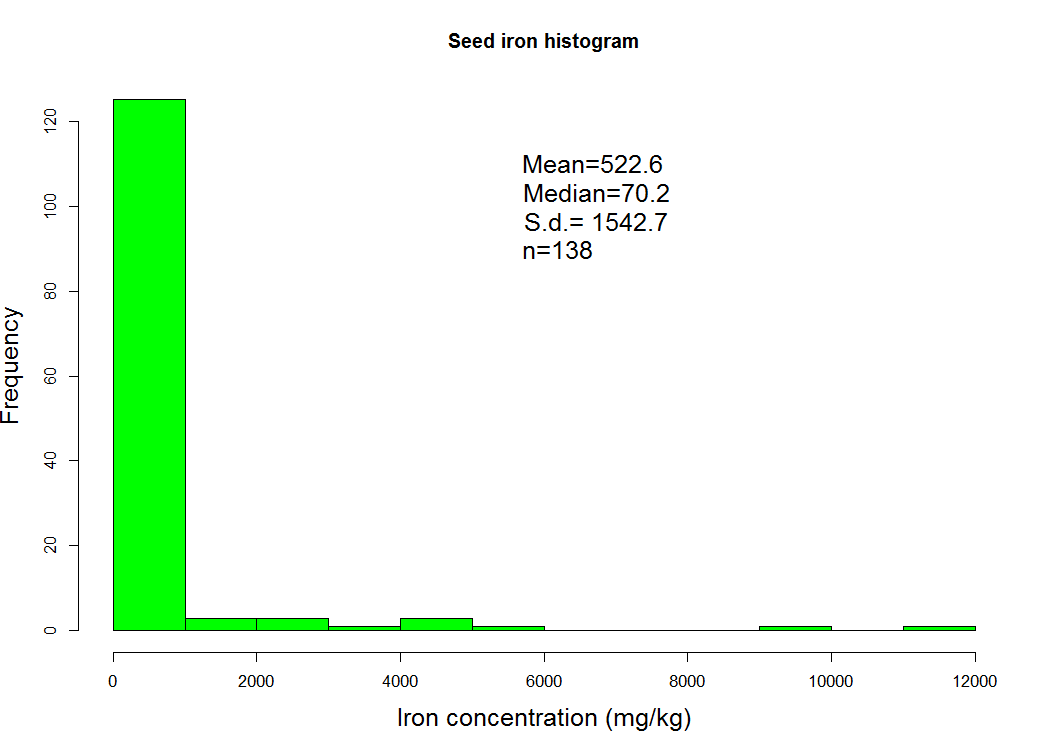 |
| --- |
| (**a**) |
| 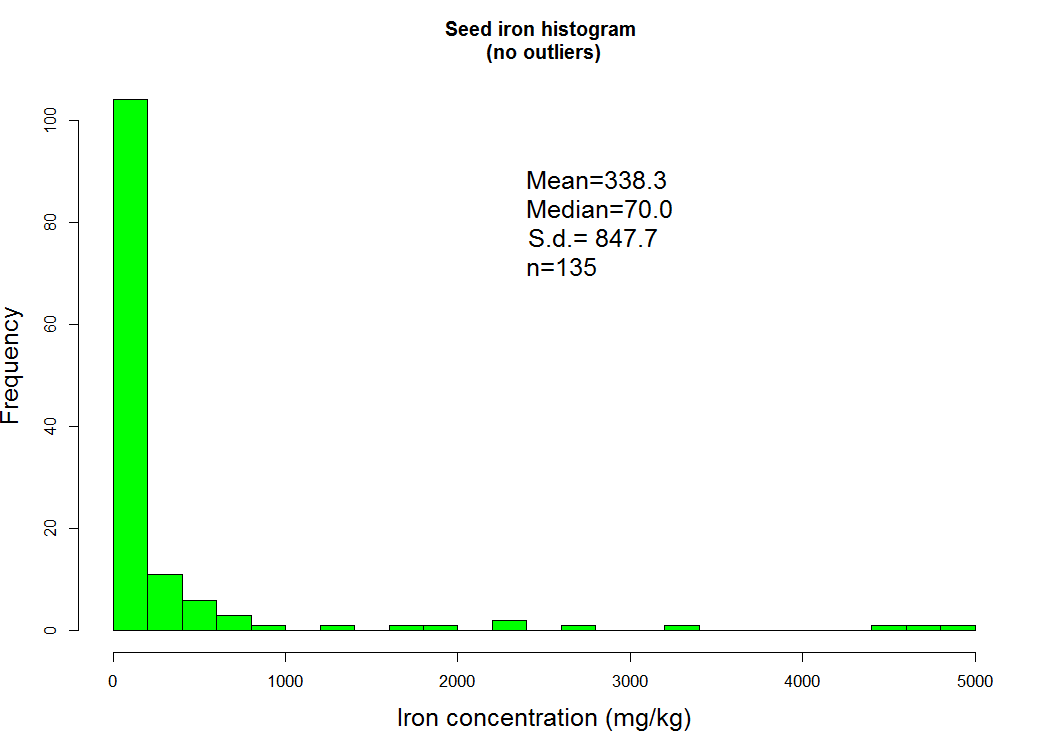 |
| (**b**) |

**Figure S8.** Histograms of seed iron concentrations. (**a**) with all outliers; (**b**) with the exclusion of values larger than 5000 mg/kg.


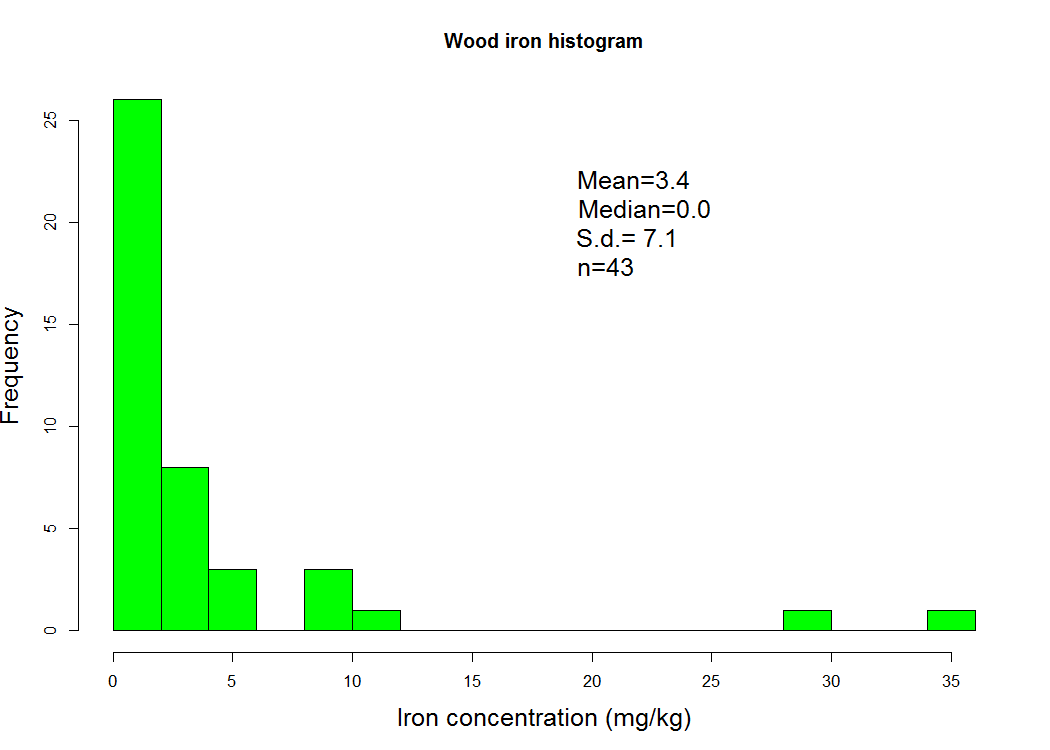


**Figure S9.** Histogram of wood iron concentrations.


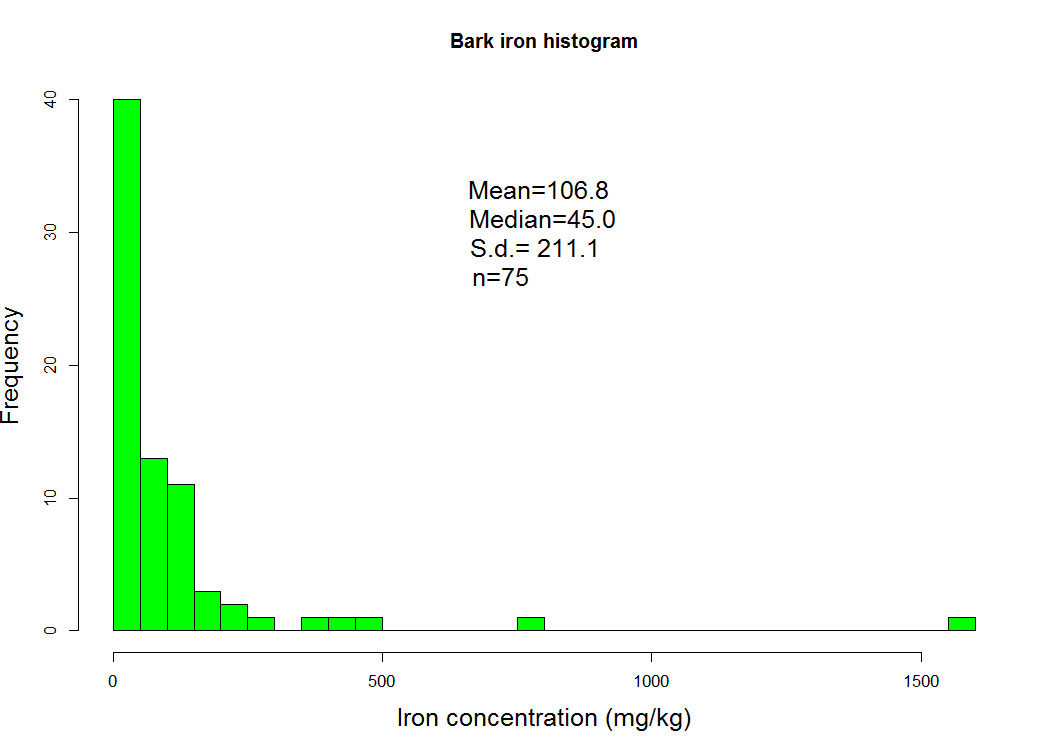


**Figure S10.** Histogram of bark iron concentrations.


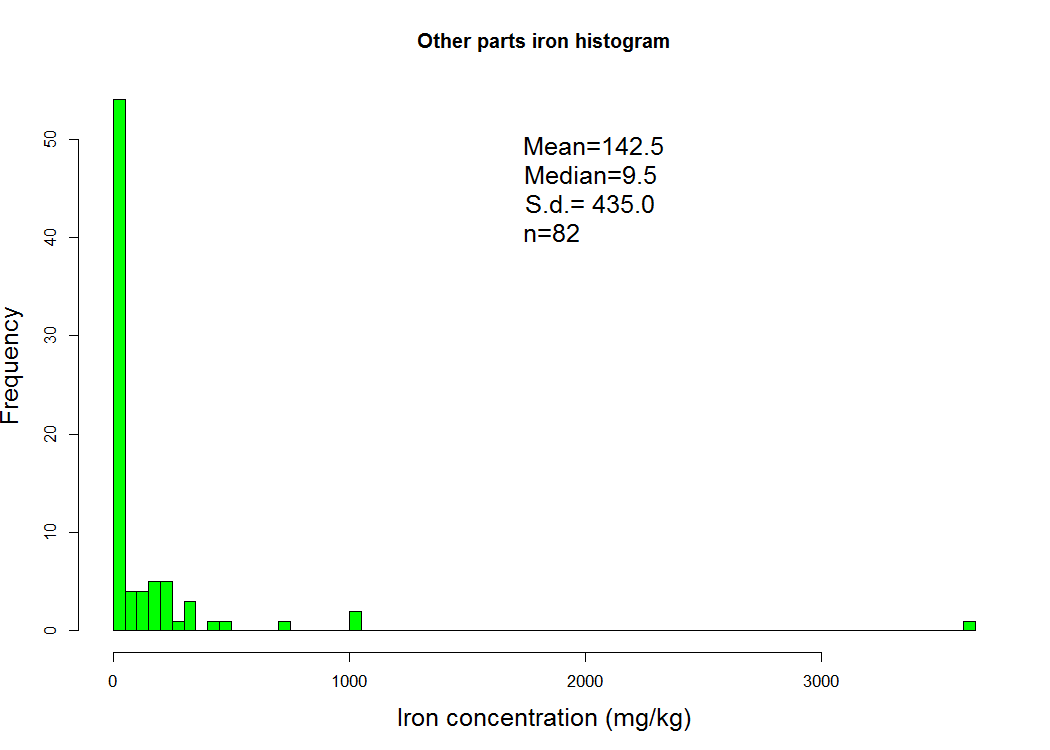


**Figure S11.** Histograms of iron concentrations in other herbal parts.

| 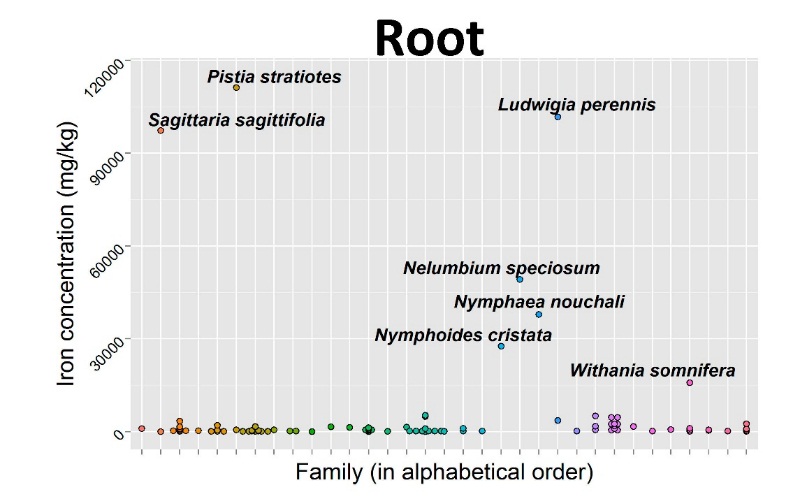 |
| --- |
| (**a**) |
| 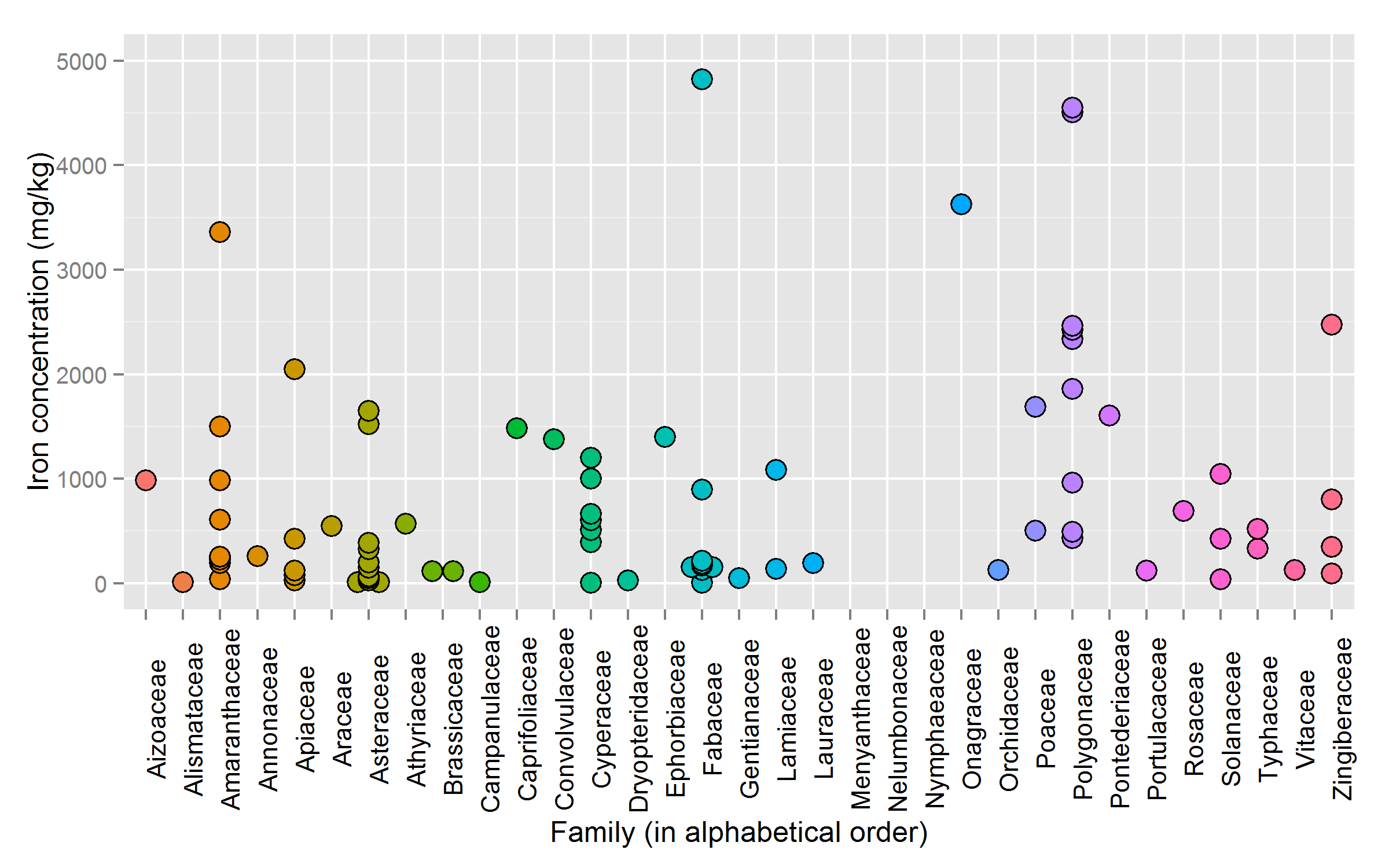 |
| (**b**) |

**Figure S12.** *Cont.*

| 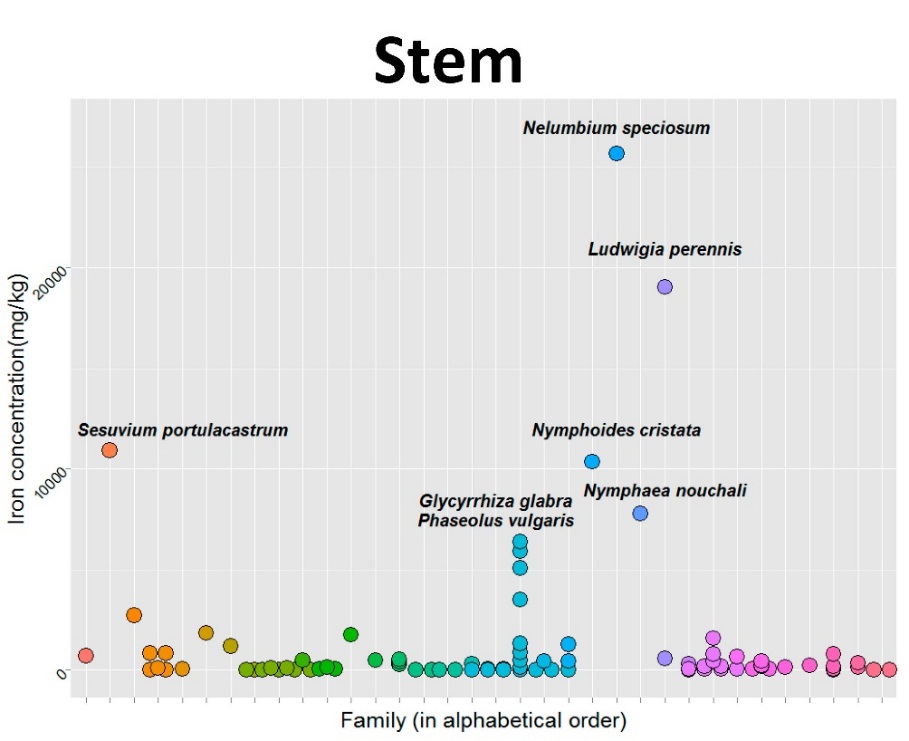 |
| --- |
| (**c**) |
| 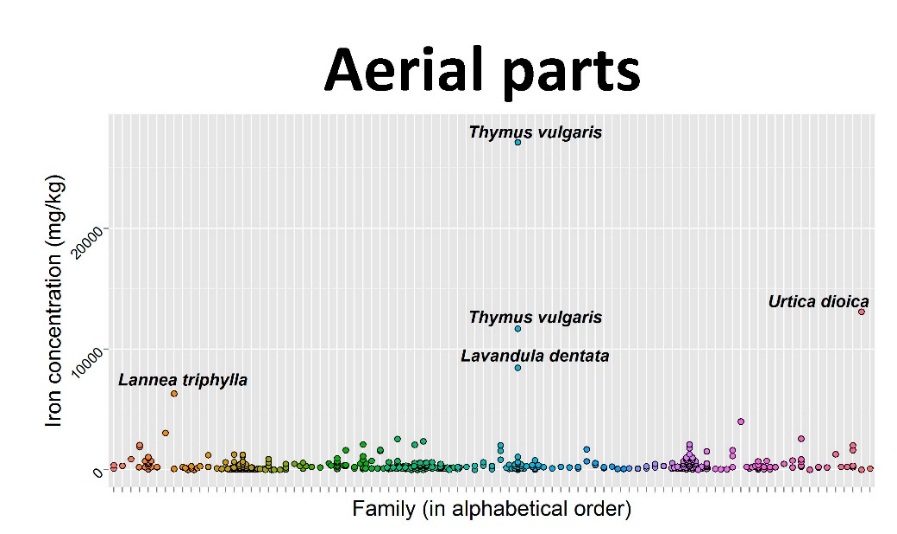 |
| (**d**) |
| 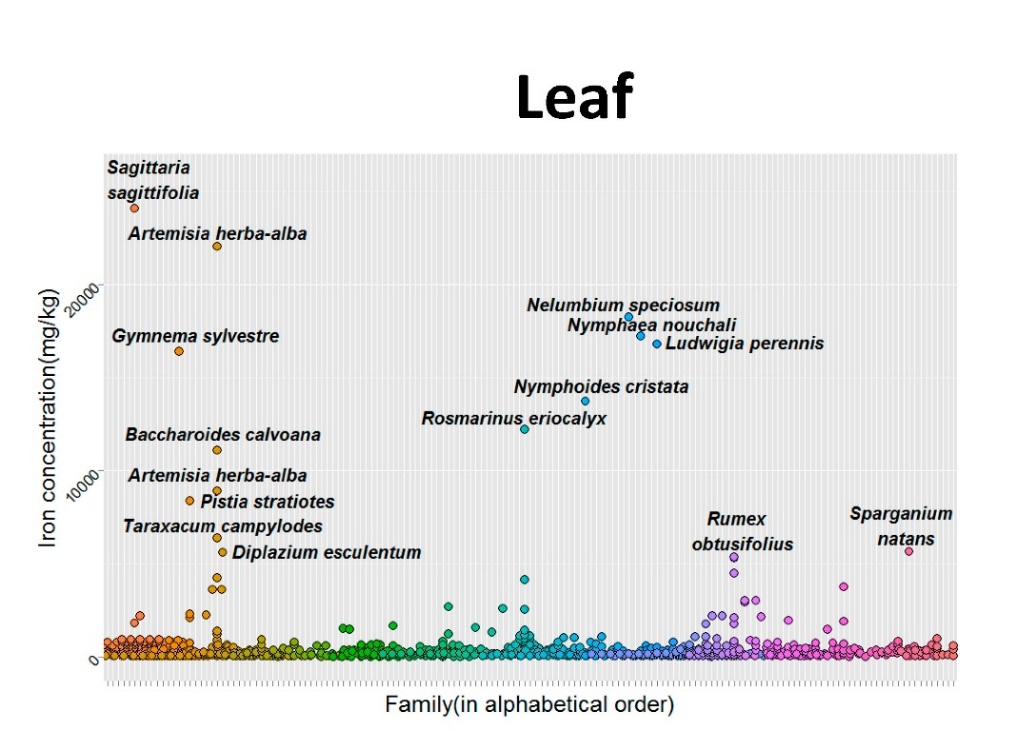 |
| (**e**) |

**Figure S12.** *Cont.*

| 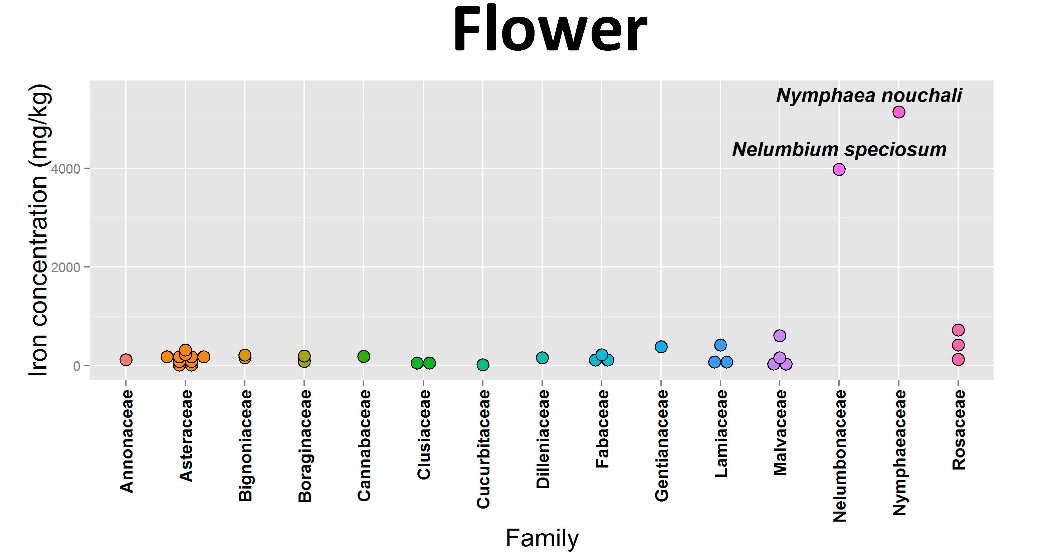 |
| --- |
| (**f**) |
| 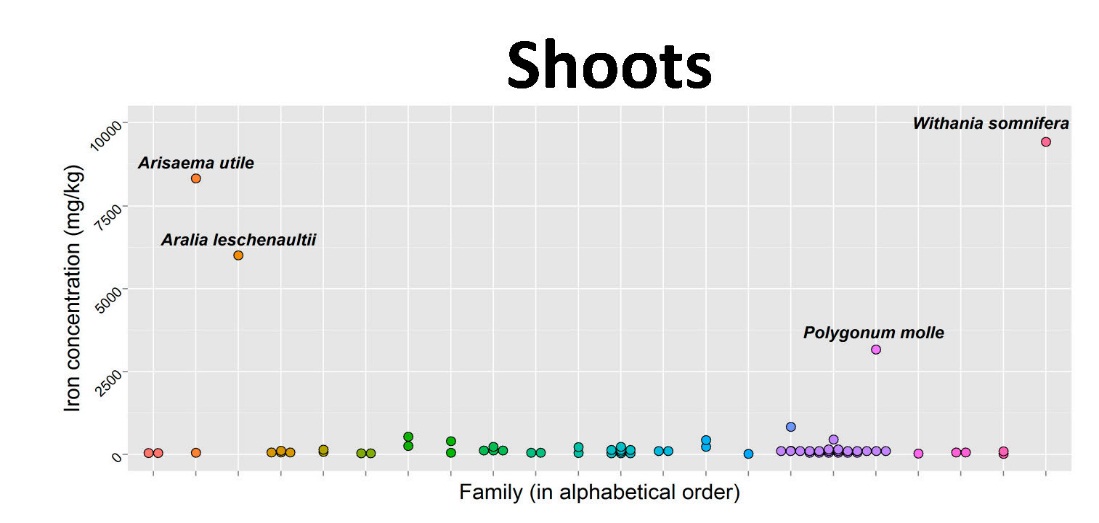 |
| (**g**) |
| 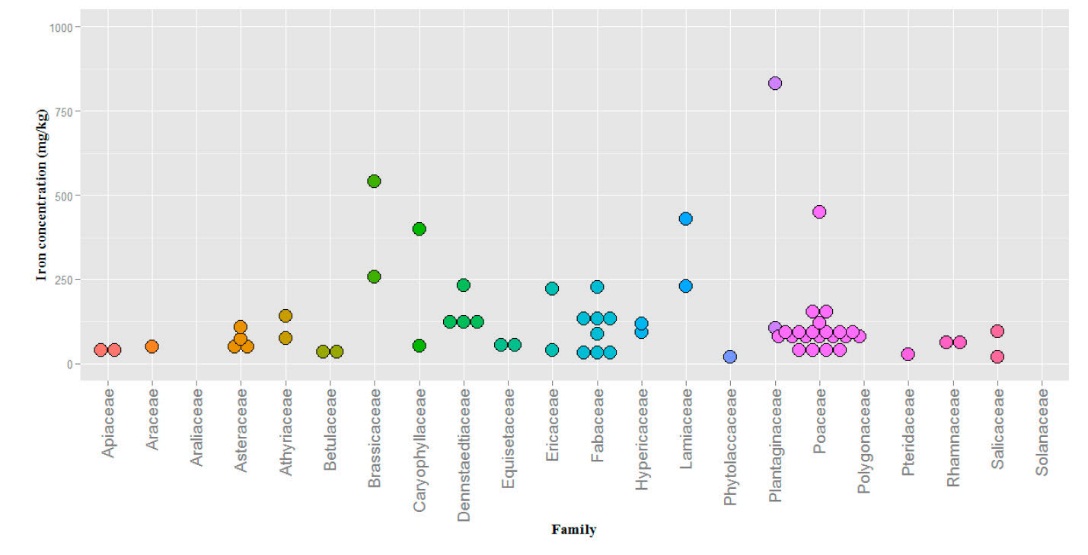 |
| (**h**) |

**Figure S12.** *Cont.*

| 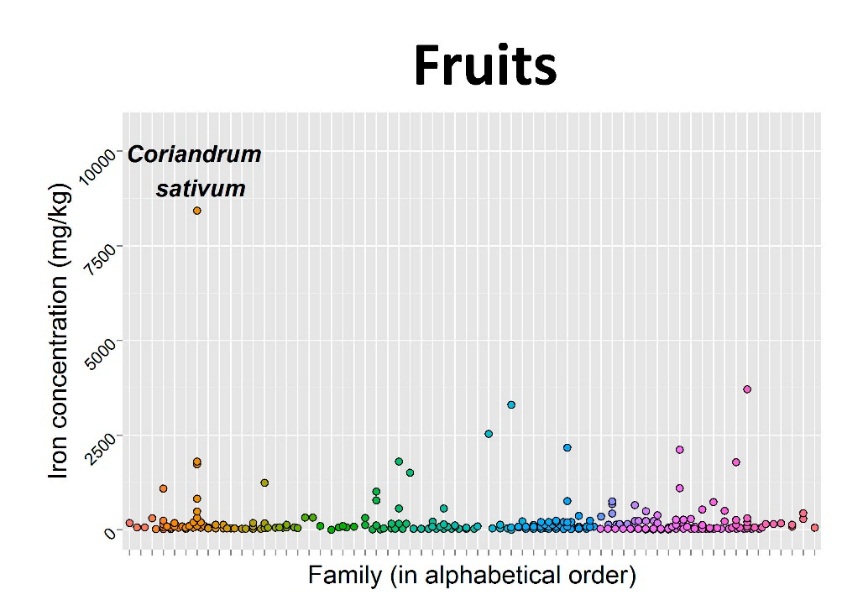 |
| --- |
| (**i**) |
| 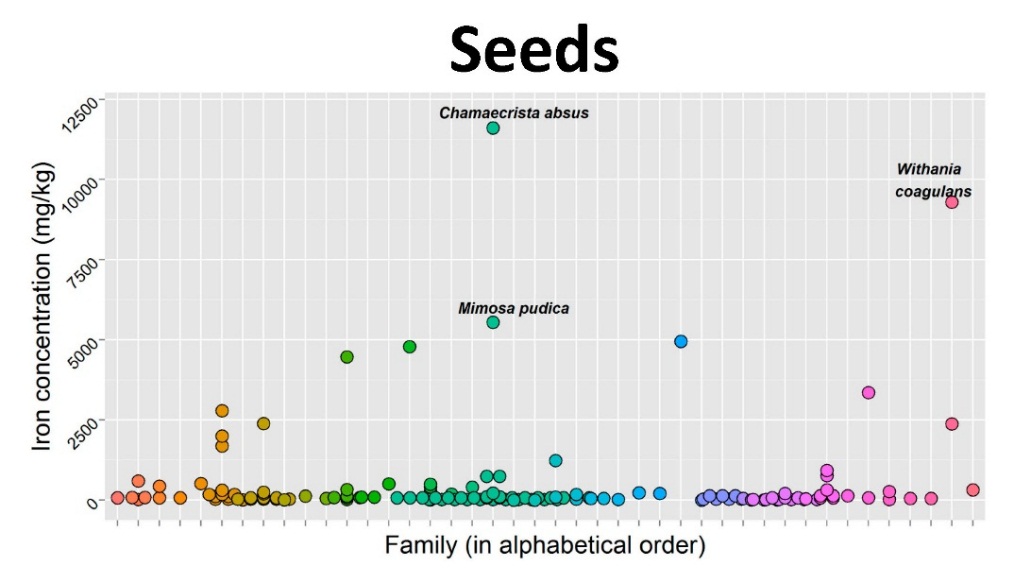 |
| (**j**) |
| 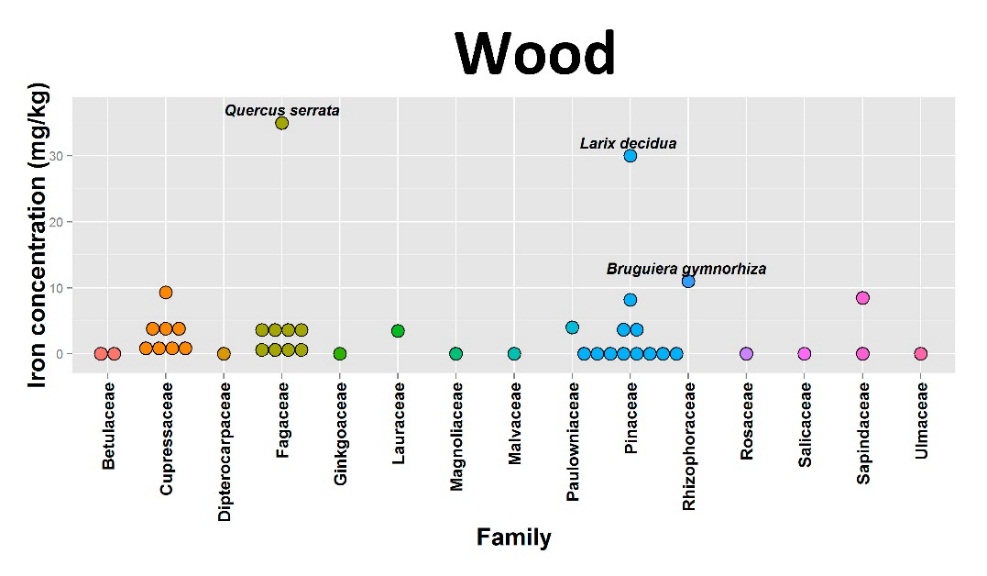 |
| (k) |

**Figure S12.** *Cont.*

| 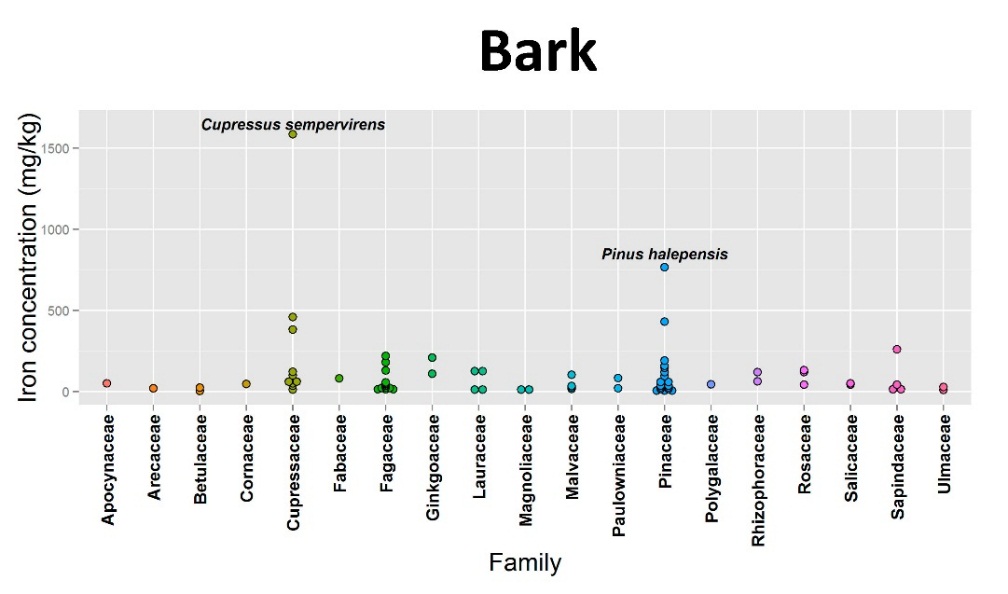 |
| --- |
| (**l**) |

**Figure S12.** Dotplot iron concentrations by family, with the largest values labeled. (**a**) In root (**b**) In root, excluding outliers larger than 5000 mg/kg; (**c**) In stem; (**d**) In aerial parts; (**e**) In leaf; f; In flower (**g**) In shoots; (**h**) In shoots, excluding outliers larger than 1000 mg/kg; (**i**) In fruit; (**j**) In seed; (**k**) In wood; (**l**) In bark.


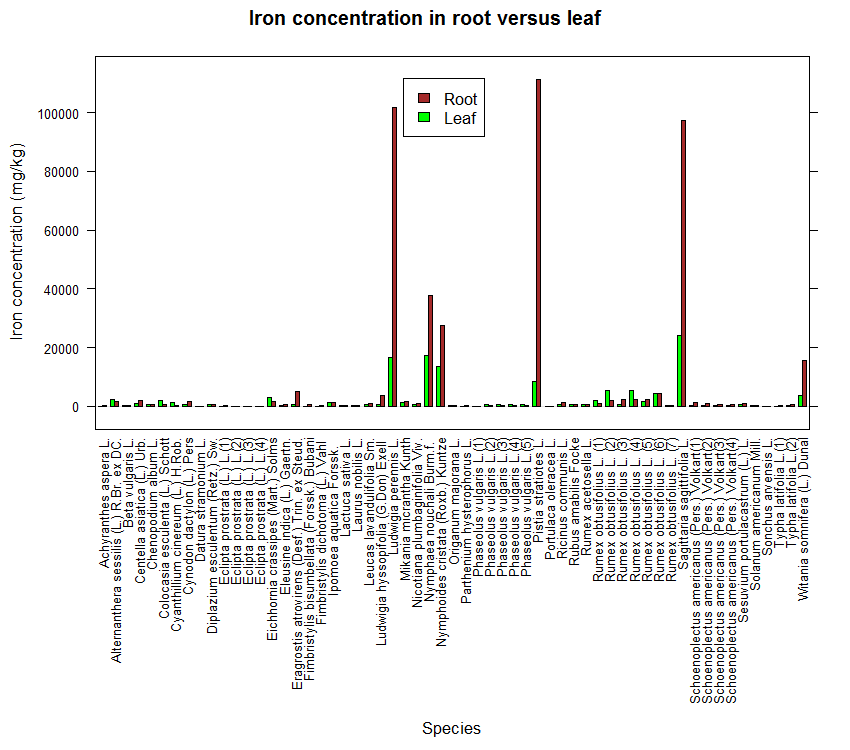


**Figure S13.** Barplots of iron concentration in root and leaf from the same species, reported by the
same publications.

| 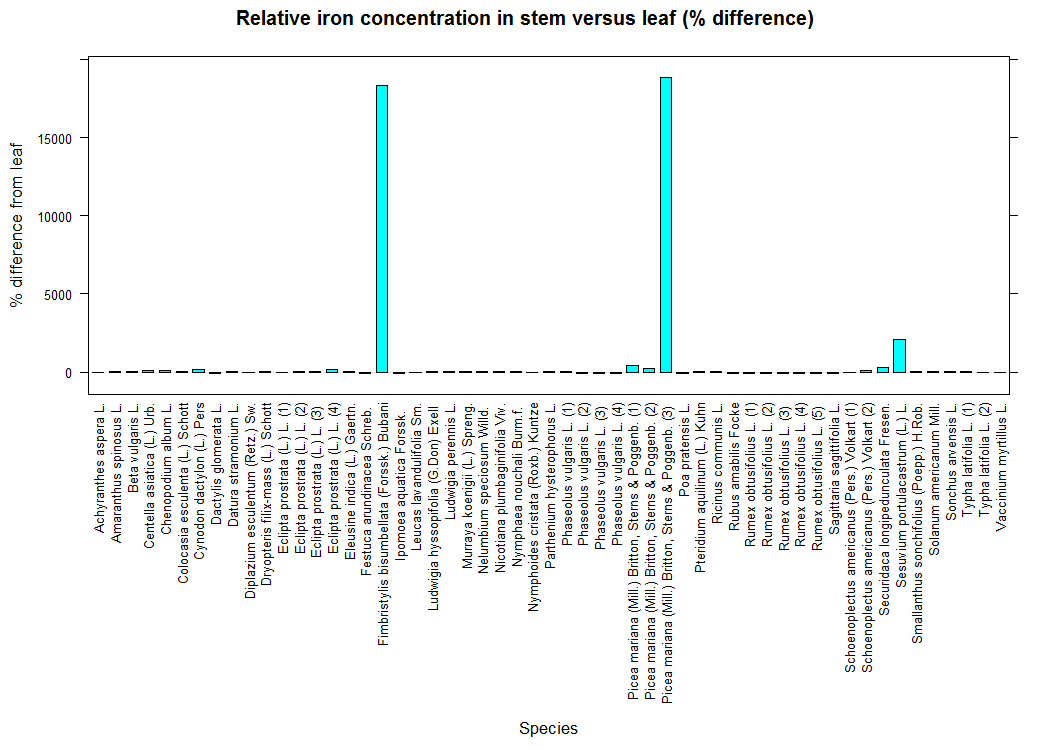 |
| --- |
| (**a**) |
| 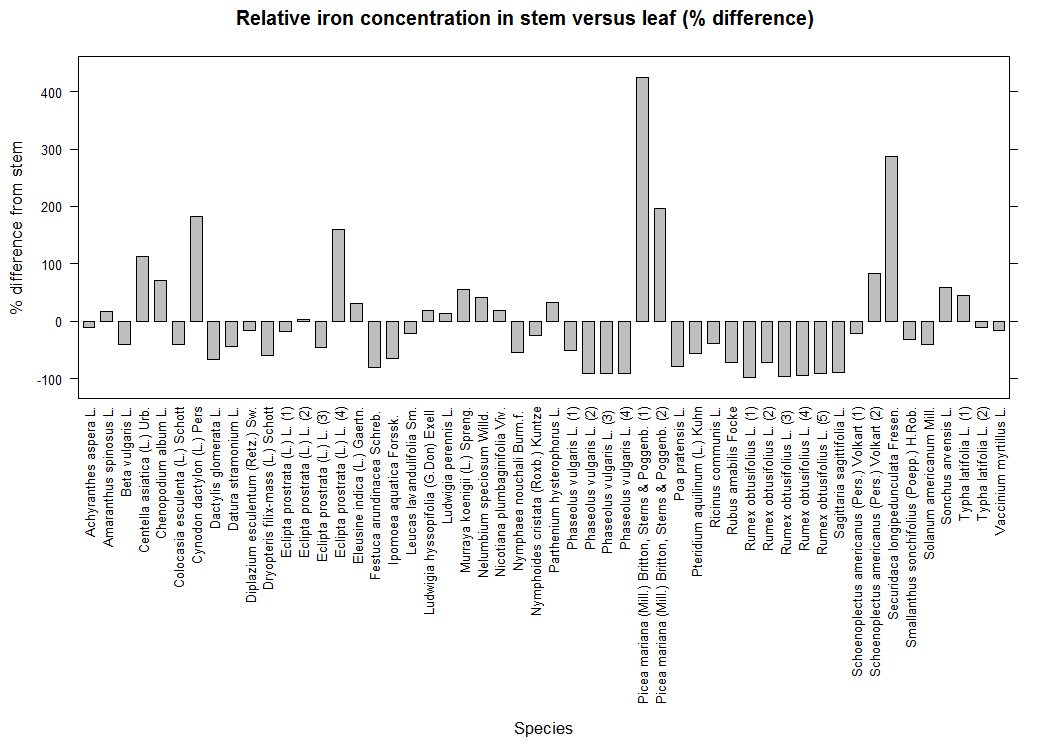 |
| (**b**) |

**Figure S14.** *Cont.*

| 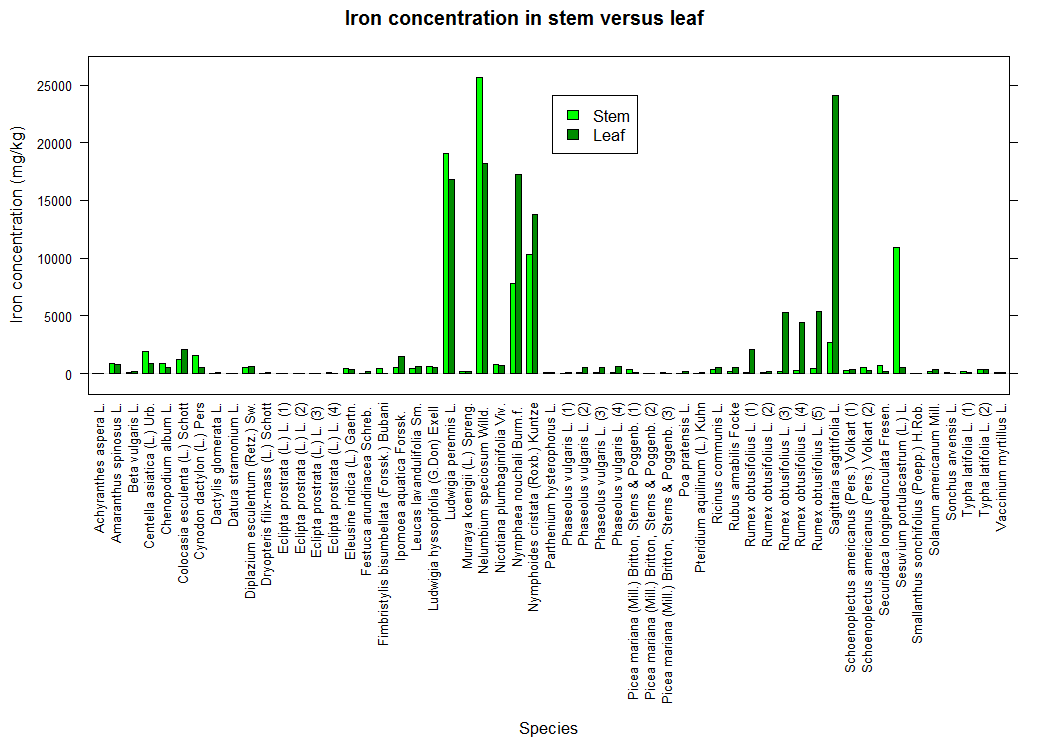 |
| --- |
| (**c**) |

**Figure S14.** Barplots depicting in a comparative manner iron concentration in stem versus leaf.
(**a**) relative iron concentration calculated as percentage against the leaf level (no outlier excluded);
(**b**) relative iron concentration calculated as percentage against the leaf level (no outlier excluded);
(**c**) absolute concentrations (mg/kg).

| 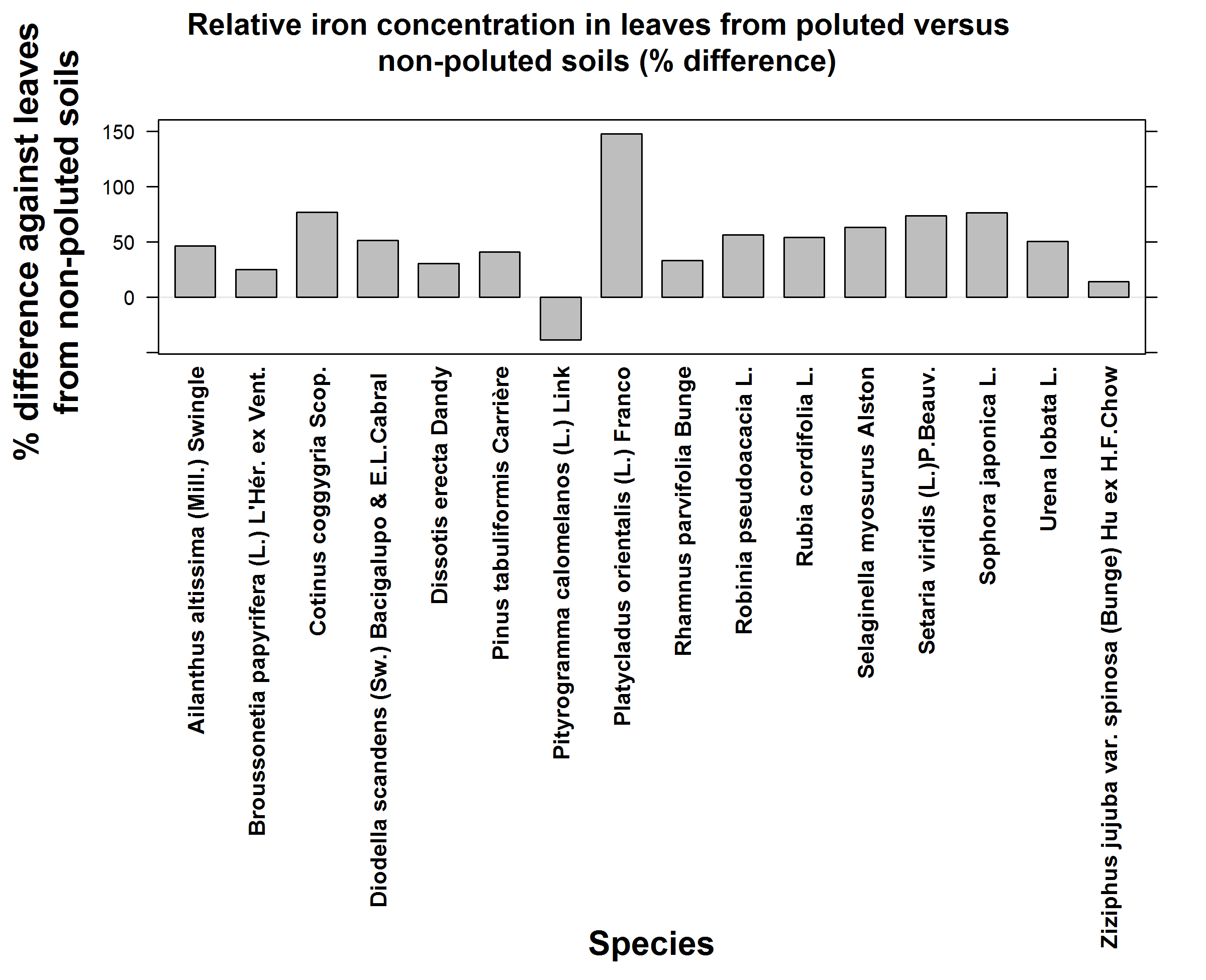 |
| --- |
| (**a**) |

**Figure S15.** *Cont.*

| 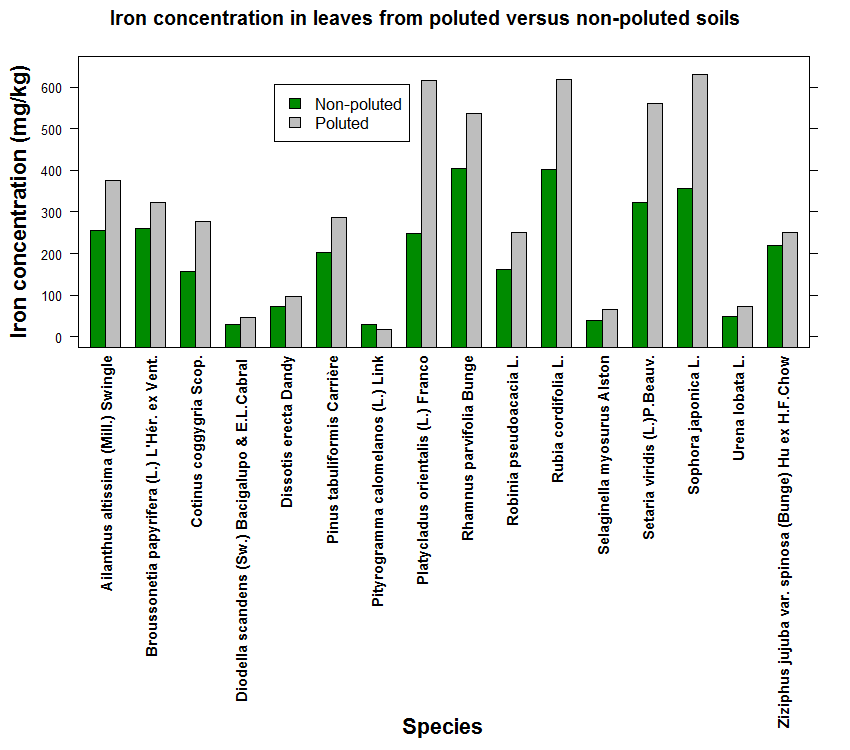 |
| --- |
| (**b**) |

**Figure S15.** Comparative barplots of iron concentrations in leaves from polluted versus non-polluted soils (absolute values). (**a**) relative concentrations—percentages from the level in leaves from non-polluted soils (bars over zero indicate higher contents in leaves from polluted areas); (**b**) absolute values. Based on [68,69].


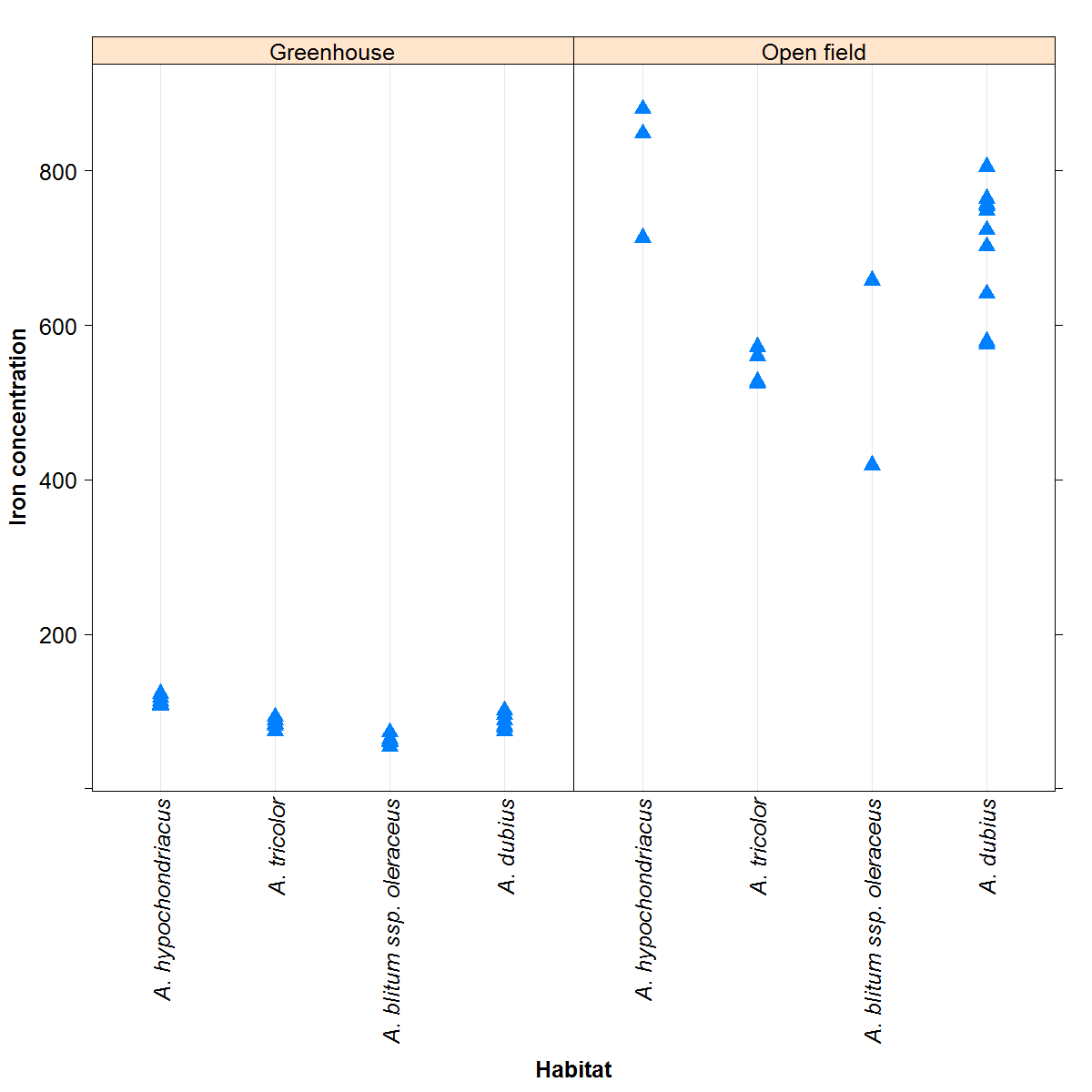


**Figure S16.** Difference in leaf iron concentration between Amaranthus species grown in open field and in greenhouse. Based on [51].


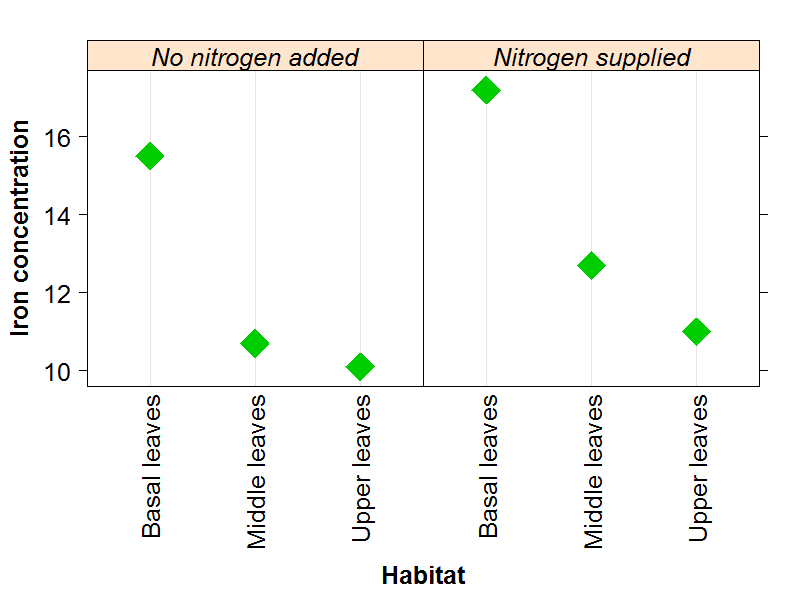


**Figure S17.** Influence of the leaf position on the iron level in this vegetative organ in *Telfairia occidentalis* Hook. f. (Cucurbitaceae) grown in a control and a nitrogen-fertilized soil. Based on [72].


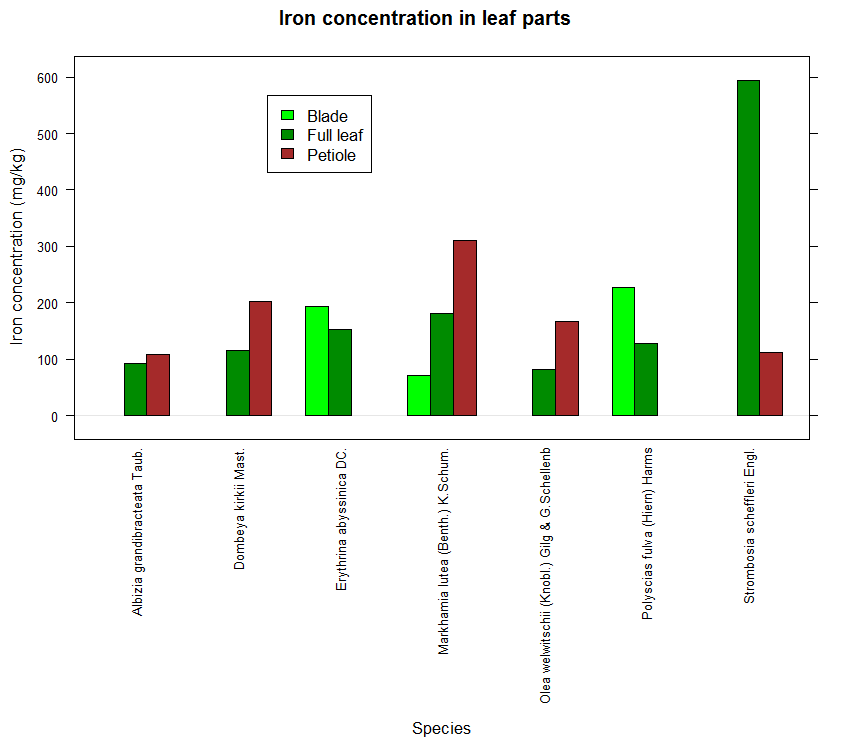


**Figure S18.** Comparative iron concentration in blade, full leaf and petiole of the same species. Based on data extracted from [75].

| 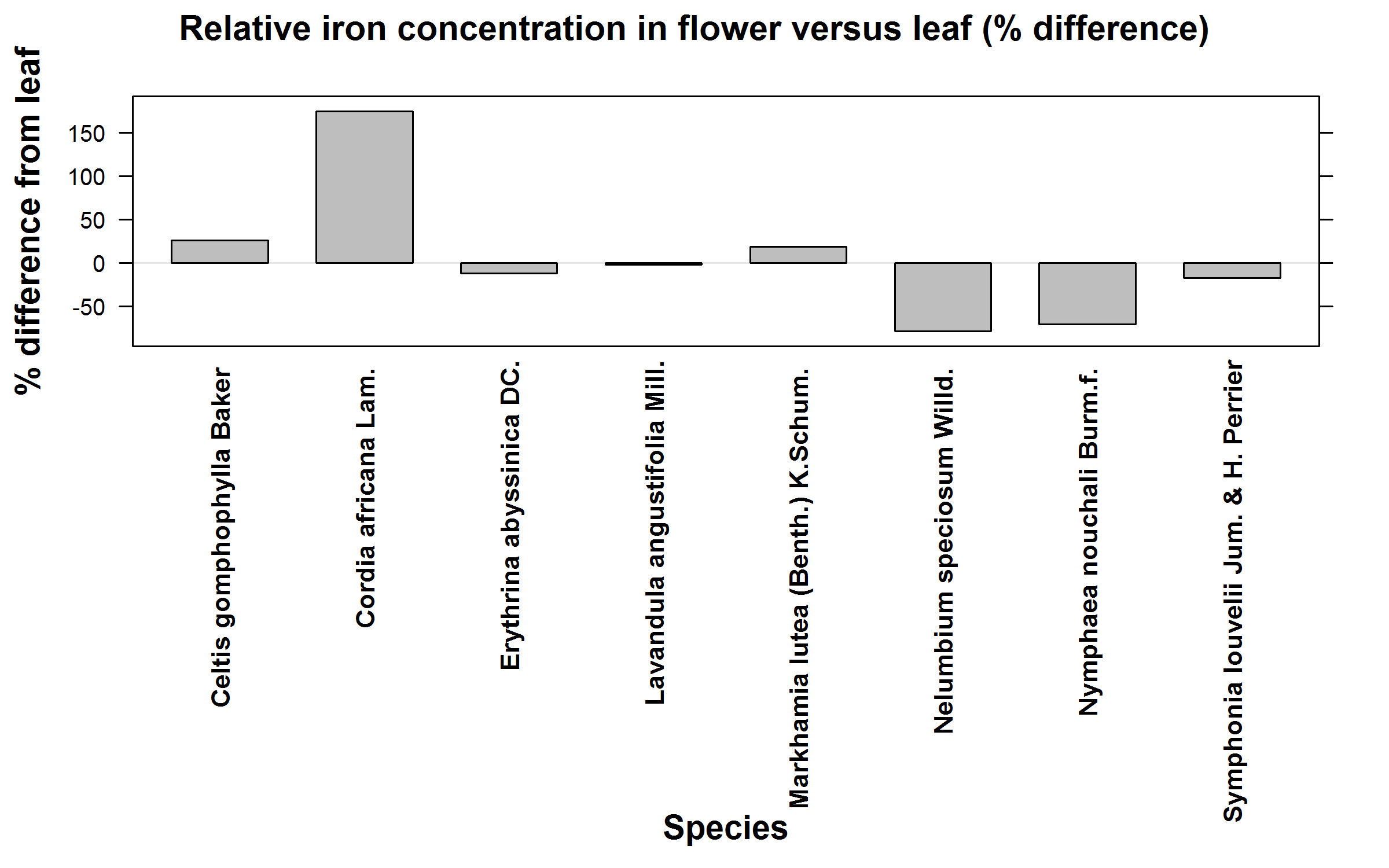 |
| --- |
| (**a**) |
| 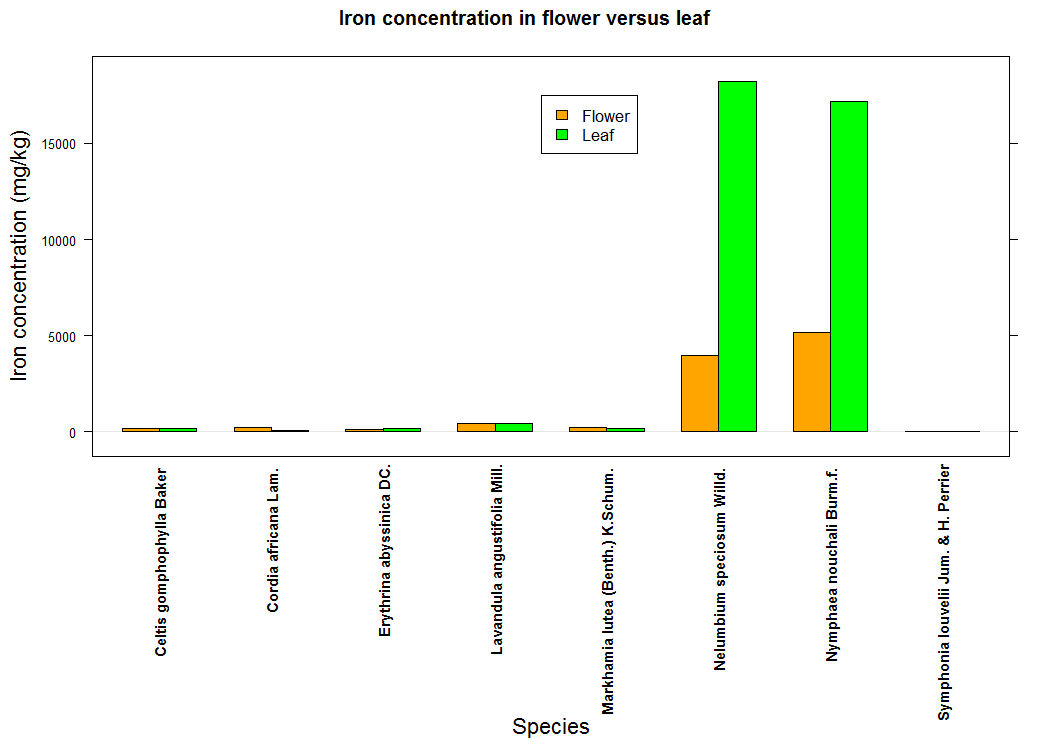 |
| (**b**) |

**Figure S19.** Comparative iron concentration in flower versus leaf: (**a**) relative values (percentages against the level in leaves—bars over zero indicate higher contents in flowers, those under zero higher contents in leaf); (**b**) absolute values.

| 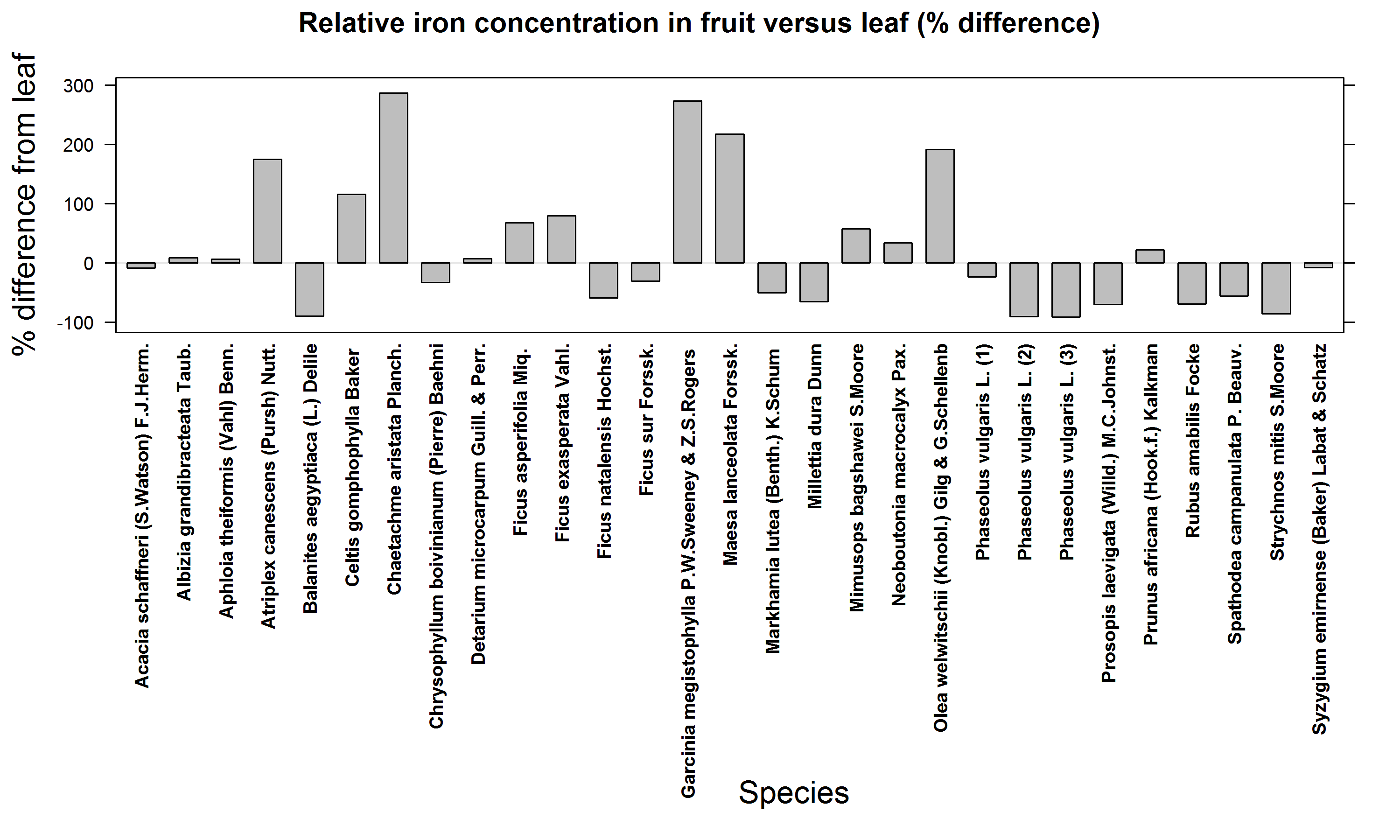 |
| --- |
| (**a**) |
| 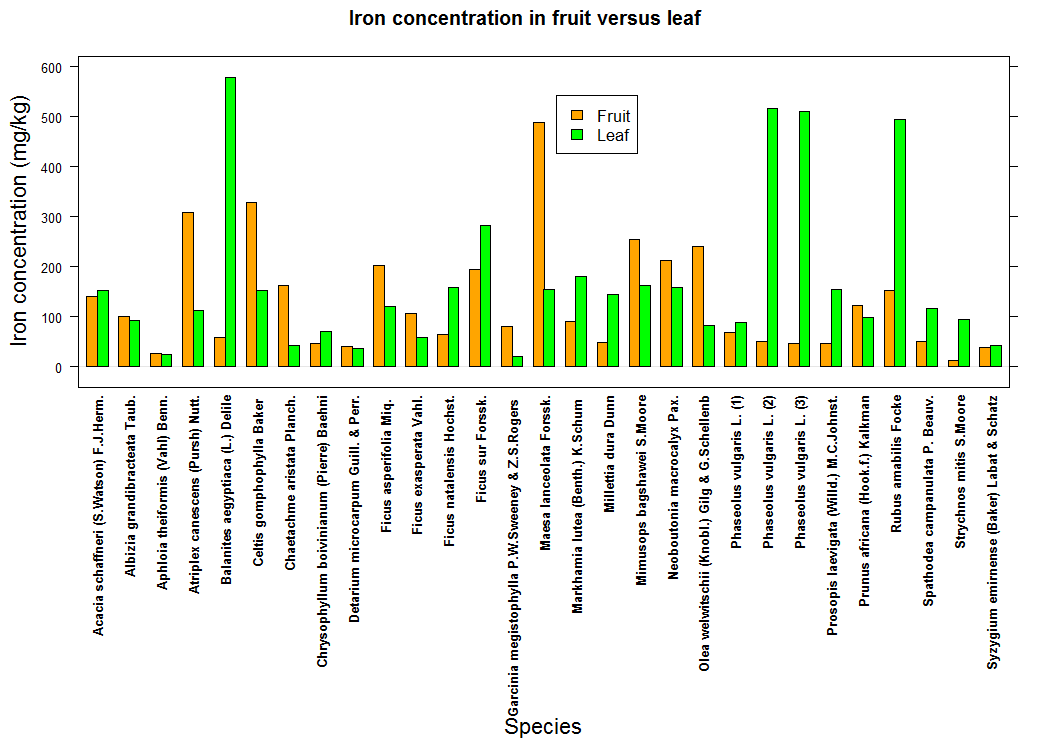 |
| (**b**) |

**Figure S20.** Comparative iron concentration in fruit versus leaf: (**a**) relative values—(percentages against the level in leaves—bars over zero indicate higher contents in fruits, those under zero higher contents in leaf); (**b**) absolute values.

| 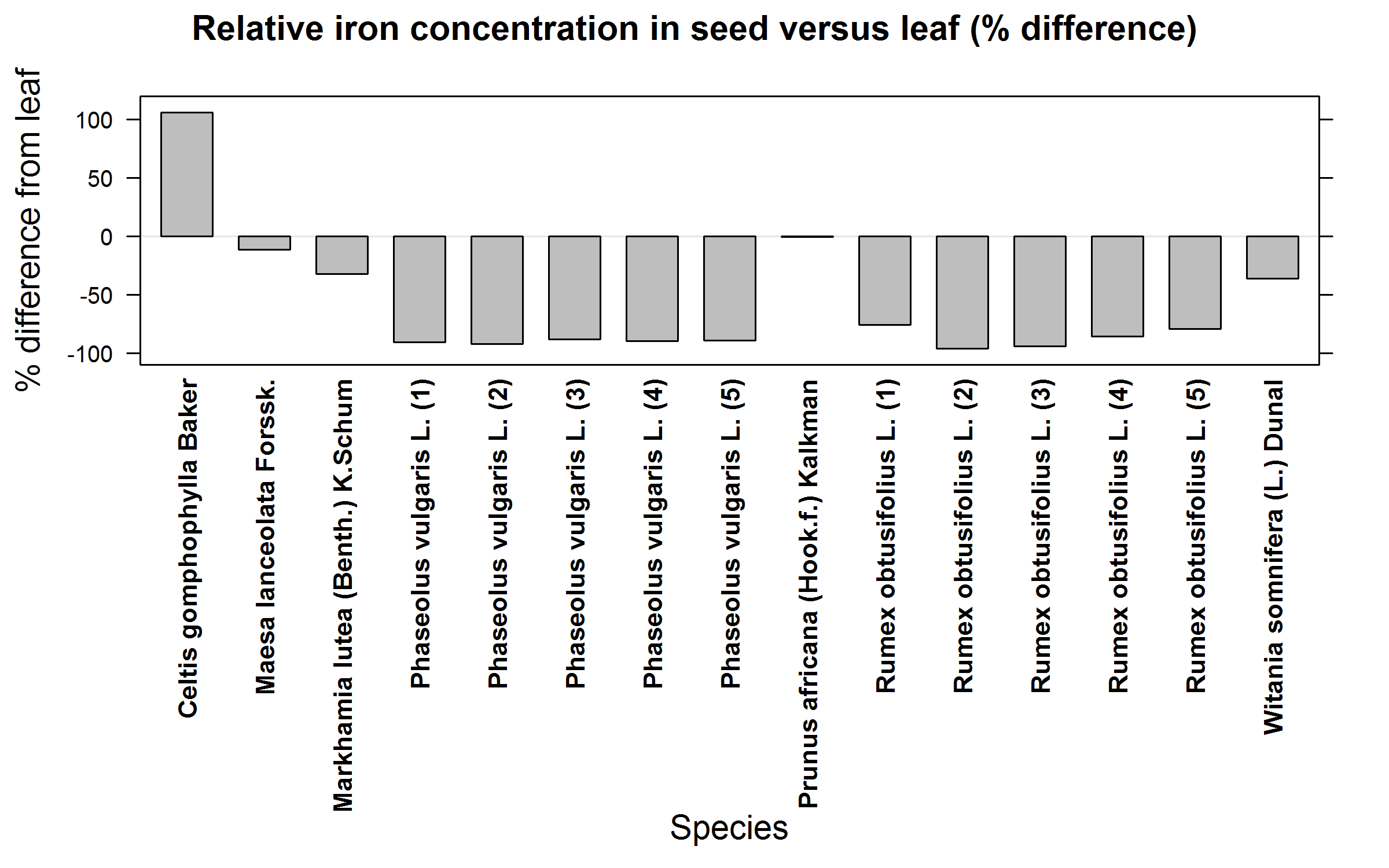 |
| --- |
| (**a**) |
| 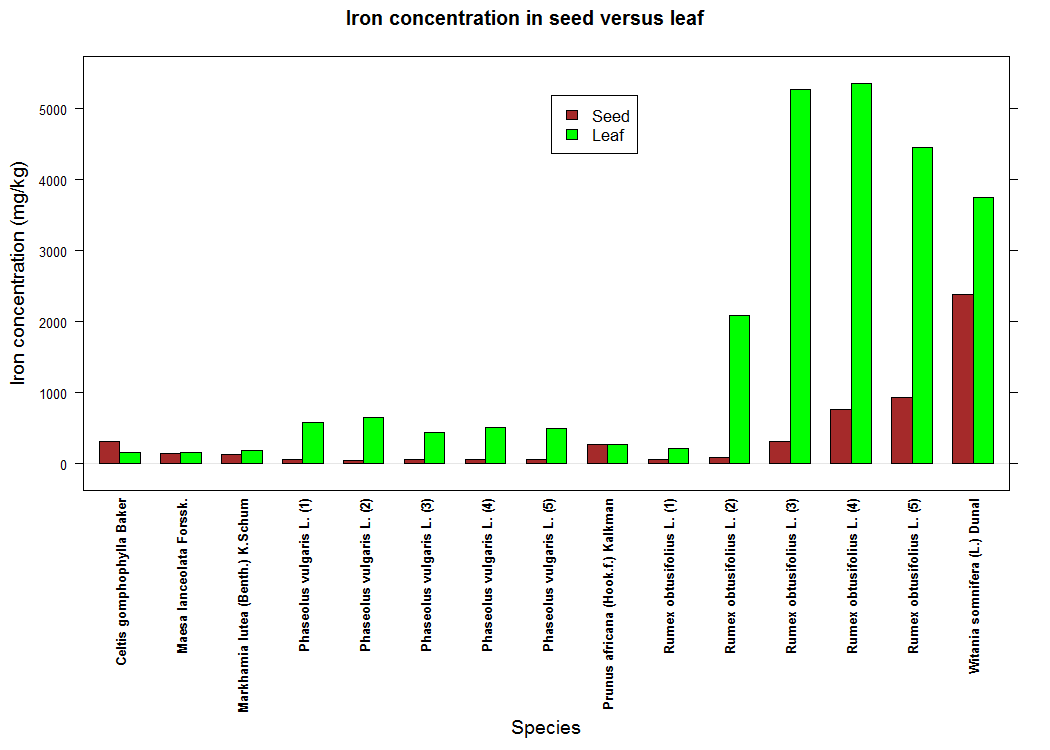 |
| (**b**) |

**Figure S21.** Comparative iron concentration in seed versus leaf: (**a**) relative values (percentages against the level in leaves (bars over zero indicate higher contents in seed, those under zero higher contents in leaf);
(**b**) absolute values.


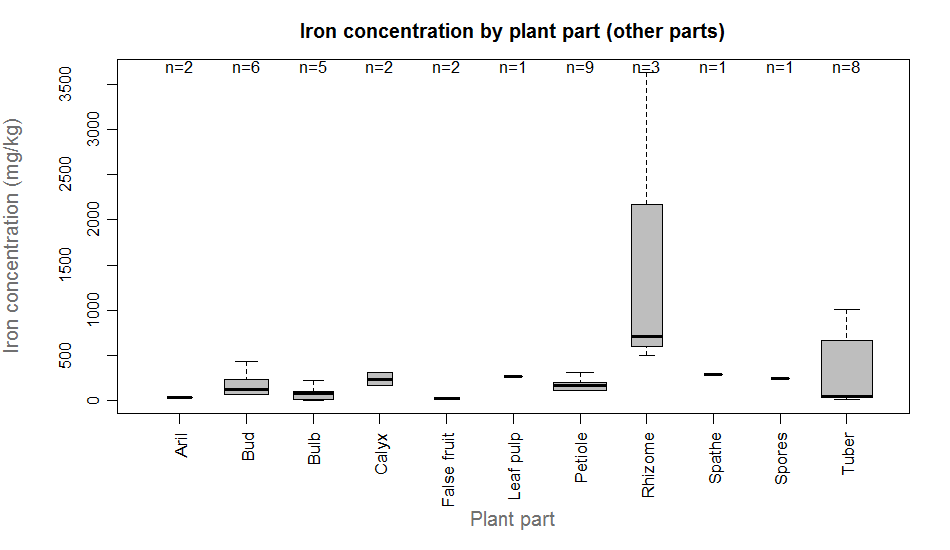


**Figure S22.** Iron concentration by plant part in other parts than the main ones (as discussed within
the article).

**Table S1.** The complete plant list we used for iron concentration variation among different plant parts.

| **Species** | **Iron Concentration  (ppm)** | **Plant Part** | **Comments** | **Family** | **Plant Group  (M, D, G, P, B)** | **Life Form  (H, V, S, Su, T)** |
| --- | --- | --- | --- | --- | --- | --- |
| Abelmoschus esculentus (L.) Moench | 426.6 [1] | Aerial parts | Destalked. | Malvaceae | D | H |
| *Abelmoschus* *ficulneus* (L.) Wight & Arn. | 54 [2] | Leaf | Reported as "leaves (or shoots)”. Saline soil (pH 3.9); Reported as  *Hibiscus ficulneus* L. | Malvaceae | D | T |
| *Abies alba* Mill. | 109.1 [3] | Leaf | Needles age class 3 | Pinaceae | G | T |
| *Abies alba* Mill. | 119.0 [4] | Leaf | Needles age class 5 | Pinaceae | G | T |
| *Abies alba* Mill. | 119.1 [5] | Leaf | Needles age class 3 | Pinaceae | G | T |
| *Abies alba* Mill. | 124.7 [6] | Leaf | Needles age class 1 | Pinaceae | G | T |
| *Abies alba* Mill. | 133.2 [7] | Leaf | Needles age class 2 | Pinaceae | G | T |
| *Abies alba* Mill. | 134.0 [8] | Leaf | Needles age class 5 | Pinaceae | G | T |
| *Abies alba* Mill. | 140.5 [9] | Leaf | Needles age class 4 | Pinaceae | G | T |
| *Abies alba* Mill. | 85.4 [10] | Leaf | Needles age class 0 | Pinaceae | G | T |
| *Abies alba* Mill. | 88.2 [11] | Leaf | Needles age class 1 | Pinaceae | G | T |
| *Abies alba* Mill. | 89.3 [12] | Leaf | Needles age class 2 | Pinaceae | G | T |
| *Abies alba* Mill. | 90.8 [13] | Leaf | Needles age class 0 | Pinaceae | G | T |
| *Abies alba* Mill. | 91.8 [14] | Leaf | Needles age class 4 | Pinaceae | G | T |
| *Abies firma* Siebold & Zucc. | 5.3 [15] | Bark |  | Pinaceae | G | T |
| *Abies firma* Siebold & Zucc. | 7.9 [16] | Bark |  | Pinaceae | G | T |
| *Abies firma* Siebold & Zucc. | 80 [17] | Leaf |  | Pinaceae | G | T |
| *Abies firma* Siebold & Zucc. | 0 [18] | Wood | Under LOQ. | Pinaceae | G | T |
| *Abies nordmanniana* subsp. *equi-trojani* (Asch. & Sint. ex Boiss.) Coode & Cullen | 145.5 [19] | Bark | Reported as *Abies bornmülleriana*  (*Abies bornmuelleriana* Mattf.) | Pinaceae | G | T |
| *Abies sachalinensis* (F.Schmidt) Mast. | 16 [20] | Bark |  | Pinaceae | G | T |
| *Abies sachalinensis* (F.Schmidt) Mast. | 95 [21] | Bark |  | Pinaceae | G | T |
| *Abies sachalinensis* (F.Schmidt) Mast. | 0 [22] | Wood | Under LOQ. | Pinaceae | G | T |
| *Acacia bussei* Sjostedt | 200 [23] | Aerial parts | These are approximations, as the authors expressed the values in grams/kg (including the iron in the group of “macro elements”) instead of the usual mg/kg or ppm. | Fabaceae | D | T |
| *Acacia constricta* A.Gray | 129 [24] | Leaf |  | Fabaceae | D | S |
| *Acacia schaffneri* (S.Watson) F.J.Herm. | 140 [25] | Fruit |  | Fabaceae | D | T |
| *Acacia schaffneri* (S.Watson) F.J.Herm. | 153 [26] | Leaf |  | Fabaceae | D | T |
| *Acacia tortilis* (Forssk.) Hayne | 200 [27] | Aerial parts | These are approximations, as the authors expressed the values in grams/kg (including the iron in the group of “macro elements”) instead of the usual mg/kg or ppm. | Fabaceae | D | T |
| *Acanthophyllum mucronatum* C.A.Mey. | 520 [28] | Whole plant | Reported as *Acanthophyllum microcephalum* Boiss. From a copper and iron mine area. | Caryophyllaceae | D | S |
| *Acanthus pubescens* (Oliv.) Engl. | 104.0 [29] | Leaf | Young leaf. | Acanthaceae | D | S |
| *Acanthus pubescens* (Oliv.)Engl. | 132.1 [30] | Leaf |  | Achantaceae | D | T |
| *Acer micranthum* Siebold & Zucc. | 146 [31] | Leaf |  | Sapindaceae | D | T |
| *Acer miyabei subsp. miaotaiense* (P.C.Tsoong) A.E.Murray | 424.4 [32] | Leaf | Reported as *Acer yangjuechi* W.P.Fang & P.L.Chiu | Sapindaceae | D | T |
| *Acer palmatum* Thunb. | 93 [33] | Leaf | Reported as *Acer palmatum* var. *amoenum* (Carrière) Ohwi | Sapindaceae | D | T |
| *Acer pehpeiense* W.P.Fang & H.Y.Su | 221.9 [34] | Leaf |  | Sapindaceae | D | T |
| *Acer pictum* Thunb. | 15 [35] | Bark | Reported as *Acer mono* Maxim. | Sapindaceae | D | T |
| *Acer pictum* Thunb. | 260 [36] | Bark | Reported as *Acer mono* Maxim. | Sapindaceae | D | T |
| *Acer pictum* Thunb. | 155 [37] | Leaf | Reported as *Acer mono* Maxim; var. *connivens* is not reported in The Plant List | Sapindaceae | D | T |
| *Acer pictum* Thunb. | 97 [38] | Leaf | Reported as *Kalopanax pictus* (Thunb.) Nakai. | Sapindaceae | D | T |
| *Acer pictum* Thunb. | 0 [39] | Wood | Reported as *Acer mono* Maxim.Under LOQ. | Sapindaceae | D | T |
| *Acer rufinerve* Siebold & Zucc. | 139 [40] | Leaf |  | Sapindaceae | D | T |
| *Acer sieboldianum* Miq. | 134 [41] | Leaf |  | Sapindaceae | D | T |
| *Achillea millefolium* L. | 158 [42] | Aerial parts |  | Asteraceae | D | H |
| *Achillea millefolium* L. | 163 [43] | Aerial parts |  | Asteraceae | D | H |
| *Achillea* *millefolium* L. | 57.6 [44] | Aerial parts |  | Asteraceae | D | H |
| *Achillea millefolium* L. | 59 [45] | Aerial parts | Razick, S.; *et al.*, 2008.  152 mg/kg reported by Stef DS *et al.* 2010, but herbal parts not stated. | Asteraceae | D | H |
| *Achillea* *millefolium* L. | 75.8 [46] | Aerial parts |  | Asteraceae | D | H |
| *Achillea millefolium* L. | 80 [47] | Aerial parts |  | Asteraceae | D | H |
| *Achillea millefolium* L. | 80 [48] | Aerial parts |  | Asteraceae | D | H |
| *Achillea millefolium* L. | 316.7 [49] | Flower |  | Asteraceae | D | H |
| *Achillea millefolium* L. | 46.8 [50] | Root |  | Asteraceae | D | H |
| *Achillea nobilis* L*.* subsp. *sipylea* (O. Schwarz) Baessler | 5.7 [51] | Aerial parts | 1000 m altitude. | Asteraceae | D | H |
| *Achillea nobilis* L*.* subsp. *sipylea* (O. Schwarz) Baessler | 8.5 [52] | Aerial parts | 1600 m altitude. | Asteraceae | D | H |
| *Achyranthes aspera* L. | 37.5 [53] | Leaf |  | Amaranthaceae | D | H |
| *Achyranthes aspera* L. | 374 [54] | Leaf |  | Amaranthaceae | D | H |
| *Achyranthes aspera* L. | 194.7 [55] | Root |  | Amaranthaceae | D | H |
| *Achyranthes aspera* L. | 33.5 [56] | Stem |  | Amaranthaceae | D | H |
| *Acorus calamus* L. | 706.52 [57] | Rhizom |  | Acoraceae | M | H |
| *Acorus calamus* L. | 700 [58] | Stem |  | Acoraceae | M | H |
| *Acorus calamus* L*.* | 16.1 [59] | Whole plant |  | Acoraceae | M | H |
| *Acrostichum aureum* L. | 75 [60] | Leaf | Reported as „leaves (or shoots)”. Saline soil (pH 3.9) | Pteridaceae | P | H |
| *Actinidia chrysantha* C.F.Liang | 264.4 [61] | Leaf |  | Actinidiaceae | D | S |
| *Adansonia digitata* L. | 42.3 [62] | Fruit |  | Malvaceae | D | T |
| *Adansonia digitata* L. | 58 [63] | Fruit |  | Malvaceae | D | T |
| *Adansonia digitata* L. | 383.9 [64] | Leaf |  | Malvaceae | D | T |
| *Adansonia digitata* L. | 43.5 [65] | Leaf |  | Malvaceae | D | T |
| *Adansonia digitata* L. | 43.5 [66] | Seed |  | Malvaceae | D | T |
| *Adenocarpus complicatus* (L.) Gay | 136.8 [67] | Shoot | From an area around an abandoned mine. | Fabaceae | D | S |
| *Adenocarpus complicatus* (L.) Gay | 88.2 [68] | Shoot | From an area around an abandoned mine. | Fabaceae | D | S |
| Adenocarpus telonensis  (Loisel) DC. | 117 [69] | Aerial parts |  | Fabaceae | D | S |
| *Adiantum reniforme* L. | 175.7 [70] | Leaf | Reported as *Adiantum reniforme* var. *sinense*. | Pteridaceae | P | H |
| *Aegle marmelos* (L.) Corrêa | 42 [71] | Fruit |  | Rutaceae | D | T |
| *Aesculus chinensis* var. *wilsonii* (Rehder) Turland & N.H.Xia | 281.1 [72] | Leaf | Reported as *Aesculus wilsonii* Rehder. | Sapindaceae | D | T |
| *Aesculus turbinata* Blume | 14 [73] | Bark |  | Sapindaceae | D | T |
| *Aesculus turbinata* Blume | 42 [74] | Bark |  | Sapindaceae | D | T |
| *Aesculus turbinata* Blume | 211 [75] | Leaf |  | Sapindaceae | D | T |
| *Aesculus turbinata* Blume | 8.5 [76] | Wood |  | Sapindaceae | D | T |
| *Aesculus wangii* Hu | 465.7 [77] | Leaf |  | Sapindaceae | D | T |
| *Afraegle paniculata* (Schumach. & Thonn.) Engl. | 128.0 [78] | Fruit |  | Rutaceae | D | T |
| *Ageratum conyzoides* (L.) L. | 161 [79] | Leaf |  | Asteraceae | D | H |
| *Ageratum conyzoides* (L.) L. | 245.9 [80] | Leaf |  | Asteraceae | D | H |
| *Ageratum conyzoides* (L.) L. | 67.7 [81] | Leaf |  | Asteraceae | D | H |
| *Ageratum conyzoides* (L.) L. | 1650 [82] | Root |  | Asteraceae | D | H |
| Agrostis castellana Boiss. & Reuter | 215 [83] | Aerial parts |  | Poaceae | M | H |
| Agrostis stolonifera L. | 264 | Aerial parts |  | Poaceae | M | H |
| *Ailanthus altissima* (Mill.) Swingle | 256.5 [84] | Leaf | From Beijing Botanica Garden (control). | Simaroubaceae | D | T |
| *Ailanthus altissima* (Mill.) Swingle | 375.1 [85] | Leaf | From hills near the Beijing Steel Factory. | Simaroubaceae | D | T |
| *Aira caryophyllea* L. | 120.7 [86] | Shoot | From an area around an abandoned mine. | Poaceae | M | H |
| *Aira caryophyllea* L. | 161.2 [87] | Shoot | From an area around an abandoned mine. | Poaceae | M | H |
| *Alangium chinense* (Lour.) Harms | 46.4 [88] | Bark |  | Cornaceae | D | T |
| *Albizia grandibracteata* Taub. | 81.8 [89] | Bark |  | Fabaceae | D | T |
| *Albizia grandibracteata* Taub. | 100.1 [90] | Fruit |  | Fabaceae | D | T |
| *Albizia grandibracteata* Taub. | 92.3 [91] | Leaf |  | Fabaceae | D | T |
| *Albizia grandibracteata* Taub. | 109.3 [92] | Petiole |  | Fabaceae | D | T |
| *Albizia grandibracteata* Taub. | 140.5 [93] | Young leaf |  | Fabaceae | D | T |
| *Alchemilla xanthochlora* Rothm. | 141 [94] | Aerial parts | Reported as  *Alchemilla vulgaris* | Rosaceae | D | H |
| *Alchemilla xanthochlora* Rothm. | 213 [95] | Aerial parts | Reported as  *Alchemilla vulgaris* | Rosaceae | D | H |
| *Alchemilla xanthochlora* Rothm. | 240.2 [96] | Aerial parts | Reported as  *Alchemilla vulgaris* | Rosaceae | D | H |
| *Alhagi maurorum* Medik. | 400 [97] | Aerial parts | Reported as *Alhagi camelorum.* | Fabaceae | D | S |
| *Allium schoenoprasum* L. | 227 [98] | Bulb |  | Amaryllidaceae | M | H |
| Allium angulosum L. | 118.5 [99] | Leaf |  | Amaryllidaceae | M | H |
| *Allium cepa* L. | 265.0 [100] | Leaf |  | Amaryllidaceae | M | H |
| *Allium cepa* L. | 430 [101] | Seed |  | Amaryllidaceae | M | H |
| *Allium cepa* L. | 66 [102] | Seed | Reported as *Allium cepa* var. *tropeana*. Variety not included in The Plant List. | Amaryllidaceae | M | H |
| *Allium cepa* L. | 50.5 [103] | Stem | Described as "onion” | Amaryllidaceae | D | H |
| *Allium fistulosum* L. | 18.6 [104] | Leaf |  | Amaryllidaceae | M | H |
| *Allium flavescens* Besser | 209.6 [105] | Leaf |  | Amaryllidaceae | M | H |
| Allium nutans L. | 118.5 [106] | Leaf | Narrow-leaved. | Amaryllidaceae | M | H |
| Allium nutans L. | 152.6 [107] | Leaf | Broad-leaved. | Amaryllidaceae | M | H |
| *Allium odorum* L. | 119.9 [108] | Leaf | Narrow-leaved | Amaryllidaceae | M | H |
| *Allium odorum* L. | 59.9 [109] | Leaf | Broad-leaved | Amaryllidaceae | M | H |
| *Allium schoenoprasum* L. | 128.5 [110] | Leaf | Broad-leaved | Amaryllidaceae | M | H |
| *Allium schoenoprasum* L. | 15 [111] | Leaf |  | Amaryllidaceae | M | H |
| *Allium schoenoprasum* L. | 99.8 [112] | Leaf | Narrow-leaved | Amaryllidaceae | M | H |
| *Allium sibthorpianum* Schult. & Schult.f. | 112.6 [113] | Leaf | Reported as *Allium montanum* Schmidt. [Nom. illeg.] | Amaryllidaceae | M | H |
| *Alnus rubra* Bong. | 0 [114] | Wood | Under LOQ. | Betulaceae | D | T |
| *Alnus* *glutinosa* (L.) Gaertn. | 240 [115] | Leaf |  | Betulaceae | D | T |
| *Aloe vera* (L.) Burm.f. | 79.5 [116] | Leaf | Succulent. | Xanthorrhoeaceae | M | H |
| *Aloe vera* (L.) Burm.f. | 269 [117] | Leaf pulp | Reported as *Aloe barbandensis* Mill*.* | Xanthorrhoeaceae | M | H |
| *Alstonia boonei* De Wild. | 51.3 [118] | Bark |  | Apocynaceae | D | T |
| *Alternanthera sessilis* (L.) R.Br. ex DC | 25.42 [119] | Leaf | Aquatic. | Amaranthaceae | D | H |
| *Alternanthera sessilis* (L.) R.Br. ex DC. | 2176.0 [120] | Leaf | Aquatic. From a paper mill contaminated area. | Amaranthaceae | D | H |
| *Alternanthera sessilis* (L.) R.Br. ex DC. | 1500 [121] | Root | Aquatic. From a paper mill contaminated area. | Amaranthaceae | D | H |
| *Althaea officinalis* L. | 53.0 [122] | Flower |  | Malvaceae | D | H |
| *Althaea officinalis* L. | 606.6 [123] | Flower |  | Malvaceae | D | H |
| *Alyssum fulvescens* Sm. | 5.9 [124] | Aerial parts | 1000 and 1600 m altitude, respectively. Reported as *Alyssum fulvescens* Sibth. & Sm. subsp. *fulvescens* | Brassicaceae | D | H |
| *Alyssum fulvescens* Sm. | 6.0 [125] | Aerial parts | 1000 and 1600 m altitude, respectively. Reported as *Alyssum fulvescens* Sibth. & Sm. subsp. *fulvescens* | Brassicaceae | D | H |
| *Amaranthus blitum* subsp. *oleraceus* (L.) Costea | 107 [126] | Leaf | Reported as *Amaranthus lividus.* Greenhouse. 48 dialysable iron | Amaranthaceae | D | H |
| *Amaranthus blitum* subsp. *oleraceus* (L.) Costea | 109 [127] | Leaf | Reported as *Amaranthus lividus.* Greenhouse.  45 dialysable iron | Amaranthaceae | D | H |
| *Amaranthus blitum* subsp. *oleraceus* (L.) Costea | 110 [128] | Leaf | Reported as *Amaranthus lividus.* Greenhouse.  45 dialysable iron | Amaranthaceae | D | H |
| *Amaranthus blitum* subsp. *oleraceus* (L.) Costea | 113 [129] | Leaf | Reported as *Amaranthus lividus.* Greenhouse.  51 dialysable iron | Amaranthaceae | D | H |
| *Amaranthus blitum* subsp. *oleraceus* (L.) Costea | 118 [130] | Leaf | Reported as *Amaranthus lividus.* Greenhouse.  48 dialysable iron | Amaranthaceae | D | H |
| *Amaranthus blitum* subsp. *oleraceus* (L.) Costea | 123 [131] | Leaf | Reported as *Amaranthus lividus.* Greenhouse.  51 dialysable iron | Amaranthaceae | D | H |
| *Amaranthus blitum* subsp. *oleraceus* (L.) Costea | 713 [132] | Leaf | Reported as *Amaranthus lividus*. 50 dialysable iron | Amaranthaceae | D | H |
| *Amaranthus blitum* subsp. *oleraceus* (L.) Costea | 848 [133] | Leaf | Reported as *Amaranthus lividus*. 57 dialysable iron | Amaranthaceae | D | H |
| *Amaranthus blitum* subsp. *oleraceus* (L.) Costea | 880 [134] | Leaf | Reported as *Amaranthus lividus*. 63 dialysable iron | Amaranthaceae | D | H |
| *Amaranthus caudatus* L. | 316 [135] | Leaf | 48 dialysable iron | Amaranthaceae | D | H |
| *Amaranthus caudatus* L. | 492 [136] | Leaf | 48 dialysable iron | Amaranthaceae | D | H |
| *Amaranthus caudatus* L. | 615 [137] | Leaf | Cultivar ‘Chua”. 55 dialysable iron | Amaranthaceae | D | H |
| *Amaranthus cruentus* L. | 484 [138] | Leaf | 46 dialysable iron | Amaranthaceae | D | H |
| *Amaranthus cruentus* L. | 495 [139] | Leaf | 48 dialysable iron | Amaranthaceae | D | H |
| *Amaranthus cruentus* L. | 530.5 [140] | Leaf | From a paper mill contaminated area. | Amaranthaceae | D | H |
| *Amaranthus cruentus* L. | 552 [141] | Leaf | 49 dialysable iron | Amaranthaceae | D | H |
| *Amaranthus cruentus* L. | 595 [142] | Leaf | 51 dialysable iron | Amaranthaceae | D | H |
| *Amaranthus cruentus* L. | 985.9 [143] | Root | From a paper mill contaminated area. | Amaranthaceae | D | H |
| *Amaranthus dubius* Mart. ex Thell. | 353.9 [144] | Leaf |  | Amaranthaceae | D | H |
| *Amaranthus dubius* Mart. ex Thell. | 525 [145] | Leaf | Cultivar *“Imbondwe”.* 55 dialysable iron | Amaranthaceae | D | H |
| *Amaranthus dubius* Mart. ex Thell. | 527 [146] | Leaf | 57 dialysable iron | Amaranthaceae | D | H |
| *Amaranthus dubius* Mart. ex Thell. | 560 [147] | Leaf | Cultivar *“Stubby”.* 54 dialysable iron | Amaranthaceae | D | H |
| *Amaranthus dubius* Mart. ex Thell. | 572 [148] | Leaf | 58 dialysable iron | Amaranthaceae | D | H |
| *Amaranthus dubius* Mart. ex Thell. | 75 [149] | Leaf | Greenhouse.  31 dialysable iron | Amaranthaceae | D | H |
| *Amaranthus dubius* Mart. ex Thell. | 81 [150] | Leaf | Greenhouse.  33 dialysable iron | Amaranthaceae | D | H |
| *Amaranthus dubius* Mart. ex Thell. | 82 [151] | Leaf | Greenhouse.  35 dialysable iron | Amaranthaceae | D | H |
| *Amaranthus dubius* Mart. ex Thell. | 84 [152] | Leaf | Cultivar *“Imbondwe”.* Greenhouse.34 dialysable iron | Amaranthaceae | D | H |
| *Amaranthus dubius* Mart. ex Thell. | 89 [153] | Leaf | Cultivar *“Noudom*”*.* Greenhouse.  37 dialysable iron | Amaranthaceae | D | H |
| *Amaranthus dubius* Mart. ex Thell. | 93 [154] | Leaf | Cultivar *“Stubby”.* Greenhouse.  35 dialysable iron | Amaranthaceae | D | H |
| *Amaranthus hybridus* L. | 135.8 [155] | Leaf |  | Amaranthaceae | D | H |
| *Amaranthus hybridus* L. | 450 [156] | Leaf | 52 dialysable iron | Amaranthaceae | D | H |
| *Amaranthus hybridus* L. | 596 [157] | Leaf | 53 dialysable iron | Amaranthaceae | D | H |
| *Amaranthus hybridus* L. | 661 [158] | Leaf | 51 dialysable iron | Amaranthaceae | D | H |
| *Amaranthus hybridus* L. | 87.5 [159] | Leaf |  | Amaranthaceae | D | H |
| *Amaranthus hypochondriacus* L. | 419 [160] | Leaf | 45 dialysable iron | Amaranthaceae | D | H |
| *Amaranthus hypochondriacus* L. | 55 [161] | Leaf | Greenhouse. 24 dialysable iron | Amaranthaceae | D | H |
| *Amaranthus hypochondriacus* L. | 60 [162] | Leaf | Greenhouse. 27 dialysable iron | Amaranthaceae | D | H |
| *Amaranthus hypochondriacus* L. | 61 [163] | Leaf | Greenhouse. 28 dialysable iron | Amaranthaceae | D | H |
| *Amaranthus hypochondriacus* L. | 61 [164] | Leaf | Cultivar *„Julma”.* Greenhouse. 28 dialysable iron | Amaranthaceae | D | H |
| *Amaranthus hypochondriacus* L. | 64 [165] | Leaf | Greenhouse. 28 dialysable iron | Amaranthaceae | D | H |
| *Amaranthus hypochondriacus* L. | 658 [166] | Leaf | 41 dialysable iron | Amaranthaceae | D | H |
| *Amaranthus hypochondriacus* L. | 73 [167] | Leaf | Greenhouse. 33  dialysable iron | Amaranthaceae | D | H |
| *Amaranthus palmeri* Swatson | 453 [168] | Leaf | 49 dialysable iron | Amaranthaceae | D | H |
| *Amaranthus spinosus* L. | 0.1 [169] | Leaf | Reported on a fresh matter basis. Converted by us on dry matter basis. | Amaranthaceae | D | H |
| *Amaranthus spinosus* L. | 13.5 [170] | Leaf |  | Amaranthaceae | D | H |
| *Amaranthus spinosus* L. | 152.7 [171] | Leaf |  | Amaranthaceae | D | H |
| *Amaranthus spinosus* L. | 24.61 [172] | Leaf |  | Amaranthaceae | D | H |
| *Amaranthus spinosus* L. | 764.4 [173] | Leaf | From a paper mill contaminated area. | Amaranthaceae | D | H |
| *Amaranthus spinosus* L. | 768 [174] | Leaf | 51 dialysable iron | Amaranthaceae | D | H |
| *Amaranthus spinosus* L. | 78.6 [175] | Leaf | From a waste dump area. | Amaranthaceae | D | H |
| *Amaranthus spinosus* L. | 785 [176] | Leaf | 60 dialysable iron | Amaranthaceae | D | H |
| *Amaranthus spinosus* L. | 3356.9 [177] | Root | From a paper mill contaminated area. | Amaranthaceae | D | H |
| *Amaranthus spinosus* L. | 37.4 [178] | Root | From a waste dump area. | Amaranthaceae | D | H |
| *Amaranthus spinosus* L. | 32.2 [179] | Stem | From a waste dump area. | Amaranthaceae | D | H |
| *Amaranthus spinosus* L. | 889.8 [180] | Stem | From a paper mill contaminated area. | Amaranthaceae | D | H |
| *Amaranthus tricolor* L. | 101 [181] | Leaf | Cultivar "*Red leaf”.* Greenhouse.  39 dialysable iron | Amaranthaceae | D | H |
| *Amaranthus tricolor* L. | 16.2 [182] | Leaf |  | Amaranthaceae | D | H |
| *Amaranthus tricolor* L. | 370.5 [183] | Leaf | Reported as *Amaranthus gangeticus* L. | Amaranthaceae | D | H |
| *Amaranthus tricolor* L. | 51 [184] | Leaf |  | Amaranthaceae | D | H |
| *Amaranthus tricolor* L. | 551.1 [185] | Leaf |  | Amaranthaceae | D | H |
| *Amaranthus tricolor* L. | 575 [186] | Leaf | Cultivar “*Red leaf*”.49 dialysable iron | Amaranthaceae | D | H |
| *Amaranthus tricolor* L. | 579 [187] | Leaf | Cultivar “*Red leaf”.* 43 dialysable iron | Amaranthaceae | D | H |
| *Amaranthus tricolor* L. | 641 [188] | Leaf | Cultivar “*Red leaf”.* 52 dialysable iron | Amaranthaceae | D | H |
| *Amaranthus tricolor* L. | 702 [189] | Leaf | Cultivar “*White leaf”.* 52 dialysable iron | Amaranthaceae | D | H |
| *Amaranthus tricolor* L. | 723 [190] | Leaf | 44 dialysable iron | Amaranthaceae | D | H |
| *Amaranthus tricolor* L. | 748 [191] | Leaf | Cultivar “*White leaf”.* 57 dialysable iron | Amaranthaceae | D | H |
| *Amaranthus tricolor* L. | 75 [192] | Leaf | Cultivar“*Chulai”.* Greenhouse. 38 dialysable iron | Amaranthaceae | D | H |
| *Amaranthus tricolor* L. | 754 [193] | Leaf | Cultivar “*Aupamalip”.* 57 dialysable iron | Amaranthaceae | D | H |
| *Amaranthus tricolor* L. | 756 [194] | Leaf | Cultivar “*Duradera”.* 45 dialysable iron | Amaranthaceae | D | H |
| *Amaranthus tricolor* L. | 764 [195] | Leaf | Cultivar “*Tiger leaf”.* 60 dialysable iron | Amaranthaceae | D | H |
| *Amaranthus tricolor* L. | 79 [196] | Leaf | Greenhouse. 38 dialysable iron | Amaranthaceae | D | H |
| *Amaranthus tricolor* L. | 805 [197] | Leaf | Cultivar “*Chulai”.* 62 dialysable iron | Amaranthaceae | D | H |
| *Amaranthus tricolor* L. | 82 [198] | Leaf | Cultivar “*Tiger leaf”.* Greenhouse. 38 dialysable iron | Amaranthaceae | D | H |
| *Amaranthus tricolor* L. | 89 [199] | Leaf | Cultivar “*Red leaf”.* Greenhouse. 41 dialysable iron | Amaranthaceae | D | H |
| *Amaranthus tricolor* L. | 96 [200] | Leaf | Cultivar “*Tiger leaf”.* Greenhouse. 41 dialysable iron | Amaranthaceae | D | H |
| *Amaranthus tuberculatus* (Moq.) Sauer | 511 [201] | Leaf | Reported as *Amaranthus rudis* J.D.Sauer. 52 dialysable iron | Amaranthaceae | D | H |
| *Amaranthus viridis* L. | 20.26 [202] | Leaf |  | Amaranthaceae | D | H |
| *Amaranthus viridis* L. | 27.0 [203] | Leaf |  | Amaranthaceae | D | H |
| *Amaranthus viridis* L. | 311 [204] | Leaf |  | Amaranthaceae | D | H |
| *Amaranthus viridis* L. | 319.9 [205] | Leaf |  | Amaranthaceae | D | H |
| *Amaranthus viridis* L. | 500.0 [206] | Leaf |  | Amaranthaceae | D | H |
| *Amaranthus viridis* L. | 581 [207] | Leaf | 43 dialysable iron | Amaranthaceae | D | H |
| *Amomum subulatum* Roxb. | 285 [208] | Fruit |  | Zingiberaceae | M | H |
| *Amorphophallus konjac* K.Koch | 11.5 [209] | Leaf | Reported as *Amorphophallus rivierei* Durand ex Carrière. | Araceae | M | H |
| *Amorphophallus paeoniifolius* (Dennst.) Nicolson | 20.9 [210] | Leaf | Reported as *Amorphophallus canmpanulatus* Decne. | Araceae | M | H |
| *Ampelopsis delavayana* var. *glabra* (Diels & Gilg) C.L.Li | 187.9 [211] | Leaf | Reported as *Ampelopsis aconitifolia* var. *glabra* Diels & Gilg. From Beijing Botanica Garden (control). | Vitaceae | D | V |
| *Ampelopsis delavayana* var. *glabra* (Diels & Gilg) C.L.Li | 404.4 [212] | Leaf | Reported as *Ampelopsis aconitifolia* var. *glabra* Diels & Gilg. From hills near the Beijing Steel Factory. | Vitaceae | D | V |
| *Anacardium* *occidentale* L. | 43 [213] | Leaf | Reported as “leaves (or shoots)”. Sandy podzolic soil (pH 5.0) | Anacardiaceae | D | T |
| *Anacyclus radiatus* Loisel. | 190 [214] | Seed |  | Asteraceae | D | H |
| *Anchusa azurea* Mill. | 50 [215] | Seed |  | Boraginaceae | D | H |
| *Anemia villosa* Humb. & Bonpl. ex Willd. | 205.7 [216] | Aerial parts |  | Anemiaceae | P | H |
| *Anethum graveolens* L. | 52.59 [217] | Leaf | 220 reported in another publication, but the part is not specified. | Apiaceae | D | H |
| *Anethum graveolens* L. | 163 [219] | Seed |  | Apiaceae | D | H |
| *Angiopteris evecta* (G. Forst.) Hoffm. | 1000.0 [220] | Leaf |  | Marattiaceae | P | H |
| *Annona senegalensis* Pers. | 84 [221] | Fruit |  | Annonaceae | Mag | T |
| *Annona senegalensis* Pers. | 259.3 [222] | Root |  | Annonaceae | Mag | T |
| Antirrhinum graniticum Rothm. | 147 [223] | Aerial parts | Reported as Antirrhinum graniticum subsp. onubensis (Fern, Casas) Valdés | Plantaginaceae | D | H |
| *Aphloia theiformis* (Vahl) Benn. | 27.1 [224] | Fruit |  | Aphloiaceae | D | T |
| *Aphloia theiformis* (Vahl) Benn. | 25.5 [225] | Leaf |  | Aphloiaceae | D | T |
| Apium graveolens L. | 126.8 [226] | Leaf |  | Apiaceae | D | H |
| Apium graveolens L. | 182.6 [227] | Leaf |  | Apiaceae | D | H |
| Apocynum venetum L. | 200 [228] | Leaf |  | Apocynaceae | D | H |
| *Arachis hypogaea* L. | 1345.2 [229] | Stem |  | Fabaceae | D | H |
| *Aralia* *leschenaultii* (DC.) J.Wen | 6010 [230] | New shoot | Reported as *Pentapanax leschenaultii* (DC)Seem | Araliaceae | D | V |
| *Araucaria angustifolia* (Bertol.)Kuntze | 7.2 [231] | Seed |  | Araucariaceae | G | T |
| Arbutus unedo L. | 204 [232] | Aerial parts |  | Ericaceae | D | T |
| *Ardisia macrocarpa* Wall*.* | 210 [233] | Fruit |  | Primulaceae | D | S |
| *Arisaema utile* Hook.f. ex Schott | 8310 [234] | New shoot | Reported as *Arisema utile* Hook. f. | Araceae | M | H |
| *Aristida adscensionis* L. | 423.2 [235] | Aerial parts |  | Poaceae | M | H |
| *Aristida adscensionis* L. | 454.0 [236] | Aerial parts |  | Poaceae | M | H |
| *Aristolochia clematitis* L. | 2210 [237] | Leaf |  | Aristolochiaceae | Mag | H |
| Arrhenatherum album (Vahl) Clayton | 29.5 [238] | Aerial parts |  | Poaceae | M | H |
| *Artemisia absinthium* L. | 80 [239] | Aerial parts |  | Asteraceae | D | Su |
| *Artemisia absinthium* L. | 80 [240] | Aerial parts | Razick S *et al.*, 2008. 629 mg/kg reported by Stef DS *et al.* 2010, but herbal parts are not stated. | Asteraceae | D | Su |
| *Artemisia annua* L. | 83.2 [241] | Whole plant |  | Asteraceae | D | H |
| *Artemisia dracunculus* L. | 201 [242] | Aerial parts |  | Asteraceae | D | H |
| *Artemisia dracunculus* L. | 243 [243] | Aerial parts |  | Asteraceae | D | H |
| *Artemisia frigida* Willd. | 168 [244] | Aerial parts |  | Asteraceae | D | Su |
| *Artemisia frigida* Willd. | 462 [245] | Aerial parts |  | Asteraceae | D | Su |
| *Artemisia herba-alba* Asso | 22053 [246] | Leaf |  | Asteraceae | D | S |
| *Artemisia herba-alba* Asso | 3605 [247] | Leaf |  | Asteraceae | D | S |
| *Artemisia herba-alba* Asso | 3617 [248] | Leaf |  | Asteraceae | D | S |
| *Artemisia herba-alba* Asso | 4208 [249] | Leaf |  | Asteraceae | D | S |
| *Artemisia herba-alba* Asso | 8882 [250] | Leaf |  | Asteraceae | D | S |
| *Artemisia sieberi* Besser | 200 [251] | Aerial parts |  | Asteraceae | D | S |
| *Artemisia gmelinii* Weber ex Stechm. | 409.2 [252] | Leaf | From hills near the Beijing Steel Factory. | Asteraceae | D | Su |
| *Arthrocnemum macrostachyum* (Moric.) K.Koch | 1067 [253] | Aerial parts |  | Amaranthaceae | D | S |
| Artocarpus altilis (Parkinson ex F.A.Zorn) Fosberg | 48.1 [254] | Fruit |  | Moraceae | D | T |
| Artocarpus heterophyllus Lam. | 14.6 [255] | Seed |  | Moraceae | D | T |
| *Asparagus officinalis* L. | 12.0 [256] | Stem |  | Asparagaceae | M | H |
| *Asplenium × ticinense* D. E. Mayer | 126 [257] | Aerial parts | This hybrid is not included in The Plant List. | Aspleniaceae | P | H |
| *Asplenium adiantum nigrum* L. | 278 [258] | Aerial parts | Reported as *Asplenium adiantum-nigrum* L. ssp. *adiantum nigrum* | Aspleniaceae | P | H |
| *Asplenium cuneifolium* Viv. | 1297 [259] | Aerial parts | Reported as *Asplenium cuneifolium* Viv. ssp. *cuneifolium* | Aspleniaceae | P | H |
| *Asplenium cuneifolium* Viv. | 679 [260] | Aerial parts | Reported as *Asplenium cuneifolium* Viv. ssp. *cuneifolium* | Aspleniaceae | P | H |
| *Asplenium nidus* L. | 269.2 [261] | Leaf | Reported as *Neottopteris nidus* (L.) J. Sm. | Aspleniaceae | P | H |
| *Asplenium onopteris* L. | 98 [262] | Aerial parts |  | Aspleniaceae | P | H |
| *Asplenium trichomanes* L. ssp *quadrivalens* D.E. Meyer | 156 [263] | Aerial parts | This ssp.is not included in The Plant List. | Aspleniaceae | P | H |
| *Asplenium* *scolopendrium* L. | 200 [264] | Leaf | Reported as *Phyllitis scolopendrium* (L.) Newman*.* | Aspleniaceae | P | H |
| *Astragalus boeticus* L. | 50 [265] | Seed |  | Fabaceae | D | H |
| *Astragalus verus* Olivier | 2117 [266] | Whole plant | From a copper and iron mine area. | Fabaceae | D | S |
| *Athyrium filix-femina* (L.) Roth | 130 [267] | Aerial parts |  | Athyriaceae | P | H |
| *Athyrium filix-femina* (L.) Roth | 198 [268] | Aerial parts |  | Athyriaceae | P | H |
| *Athyrium filix-femina* (L.) Roth | 141.8 [269] | Shoot | From an area around an abandoned mine. | Athyriaceae | P | H |
| *Athyrium filix-femina* (L.) Roth | 76.5 [270] | Shoot | From an area around an abandoned mine. | Athyriaceae | P | H |
| *Athyrium filix-femina* (L.) Roth | 470 [271] | Leaf |  | Athyriaceae | P | H |
| *Atriplex canescens* (Pursh) Nutt. | 308 [272] | Fruit |  | Amaranthaceae | D | S |
| *Atriplex canescens* (Pursh) Nutt. | 112 [273] | Leaf |  | Amaranthaceae | D | S |
| *Atriplex lentiformis* (Torr.) S.Watson | 50 [274] | Aerial parts |  | Amaranthaceae | D | S |
| *Atriplex portulacoides* L. | 632 [275] | Aerial parts | Reported as *Halimione portulacoides* (L.) Aellen*.* | Amaranthaceae | D | S |
| *Attalea maripa* (Aubl.) Mart. | 20 [276] | Bark | It may be argued that palm trees (including Arecaceae species) are not strictly trees (not having secondary wood), but rather large grasses, although in another view “tree” is not a technical term, merely designating a certain size. | Arecaceae | M | T* |
| *Avena sativa* L. | 155.0 [277] | Aerial parts |  | Poaceae | M | H |
| *Avena sativa* L. | 175 [278] | Stem |  | Poaceae | M | H |
| *Avena sterilis* L. | 40 [279] | Seed |  | Poaceae | M | H |
| *Azadirachta indica* A. Juss. | 333.6 [280] | Leaf |  | Meliaceae | D | T |
| *Azadirachta indica* A. Juss. | 475 [281] | Leaf |  | Meliaceae | D | T |
| *Azanza garckeana* (F.Hoffm.) Exell & Hillc. | 84 [282] | Fruit |  | Malvaceae | D | T |
| *Baccaurea ramiflora* Lour*.* | 750 [283] | Fruit | Reported as *Baccaurea sapida* (Roxb.) Müll.Arg.. | Phyllanthaceae | D | T |
| *Baccharis trimera* (Less.) DC. | 172.5 [284] | Leaf |  | Asteraceae | D | H |
| *Baccharis trimera* (Less.) DC. | 230.4 [285] | Leaf |  | Asteraceae | D | H |
| *Baccharoides calvoana* (Hook.f.) "Isawumi, El-Ghazaly & B.Nord." | 11100.0 [286] | Leaf | Stated as 1.1%. Reported as *Vernonia calvoana* (Hook.f.) Hook.f. | Asteraceae | D | T |
| *Baeckea* *frutescens* L. | 47 [287] | Leaf | Reported as “leaves (or shoots)”. Sandy podzolic soil (pH 5.0) | Myrtaceae | D | T |
| *Balanites aegyptiaca* (L.) Delile | 58.0 [288] | Fruit |  | Zygophyllaceae | D | T |
| *Balanites aegyptiaca* (L.) Delile | 579.3 [289] | Leaf |  | Zygophyllaceae | D | T |
| *Basella alba* L. | 445.7 [290] | Leaf | Reorted as *Basella rubra* L. | Basellaceae | D | H |
| *Basella alba* L*.* | 545 [291] | Leaf | Reportes as *Basella rubra* L. | Basellaceae | D | V |
| *Bauhinia forficata* Link | 151.0 [292] | Leaf |  | Fabaceae | D | T |
| *Bauhinia forficata* Link | 155.3 [293] | Leaf |  | Fabaceae | D | T |
| *Bauhinia purpurea* L. | 147 [294] | Leaf |  | Fabaceae | D | T |
| *Bauhinia purpurea* L. | 168.0 [295] | Leaf |  | Fabaceae | D | T |
| Bellucia grossularioides Triana | 449 [296] | Leaf | From an area degraded by anthropic activities. | Melastomataceae | D | T |
| *Benincasa hispida* (Thunb.) Cogn. | 10.3 [297] | Fruit |  | Cucurbitaceae | D | H |
| *Benincasa hispida (Thunb.) Cogn.* | 421.0 [298] | Leaf |  | Cucurbitaceae | D | H |
| *Benkara malabarica* (Lam.) Tirveng. | 235.2 [299] | Aerial parts |  | Rubiaceae | D | T |
| *Berberis nervosa* Pursh. | 30 [300] | Fruit |  | Berberidaceae | D | S |
| *Beta macrocarpa* Guss. | 70 [301] | Seed |  | Amaranthaceae | D | H |
| *Beta vulgaris* L. | 169.4 [302] | Leaf |  | Amaranthaceae | D | H |
| *Beta vulgaris* L. | 238 [303] | Leaf |  | Amaranthaceae | D | H |
| *Beta vulgaris* L. | 335.6 [304] | Leaf | Reported as *Beta vulgaris* var. *cicla* L. | Amaranthaceae | D | H |
| *Beta vulgaris* L. | 251.9 [305] | Root | Reported as *Beta vulgaris* var. *cicla* L. | Amaranthaceae | D | H |
| *Beta vulgaris* L. | 101.4 [306] | Stem |  | Amaranthaceae | D | H |
| *Betula pendula* Roth | 34.5 [307] | Shoot | From an area around an abandoned mine. | Betulaceae | D | T |
| *Betula pendula* Roth | 36.5 [308] | Shoot | From an area around an abandoned mine. | Betulaceae | D | T |
| *Betula platyphylla* Sukaczev | 24 [309] | Bark | Reported as *Betula platyphylla* var. *japonica* (Miq.) Hara. | Betulaceae | D | T |
| *Betula platyphylla* Sukaczev | 4 [310] | Bark | Reported as *Betula platyphylla* var. *japonica* (Miq.) Hara. | Betulaceae | D | T |
| *Betula platyphylla* Sukaczev | 0 [311] | Wood | Reported as *Betula platyphylla* var. *japonica* (Miq.) Hara. Under LOQ. | Betulaceae | D | T |
| *Betula pubescens* Ehrh. | 106.3 [312] | Leaf | Reported as *Betula alba* L. | Betulaceae | D | T |
| *Betula pubescens* Ehrh. | 130 [313] | Leaf |  | Betulaceae | D | T |
| *Betula pubescens* Ehrh. | 140 [314] | Leaf |  | Betulaceae | D | T |
| *Betula pubescens* Ehrh. | 180 [315] | Leaf | Median=70 in this publication. | Betulaceae | D | T |
| *Betula pubescens* Ehrh. | 40 [316] | Leaf | Median=70 in this publication. | Betulaceae | D | T |
| Blackstonia perfoliata (L.) Huds. | 258 [317] | Aerial parts |  | Gentianaceae | D | H |
| *Blechnum nipponicum* Makino | 139 [318] | Leaf | *Reportes as Struthiopteris niponica* (Kunze) Nakai | Blechnaceae | P | H |
| *Blechnum occidentale* L. | 82.4 [319] | Aerial parts |  | Blechnaceae | P | H |
| *Blechnum* *serrulatum* Rich. | 18 [320] | Leaf | Reported as “leaves (or shoots)”. Peat soil (pH 3.6). | Blechnaceae | P | H |
| *Blechnum spicant* (L.) Sm*.* | 160 [321] | Leaf |  | Blechnaceae | P | H |
| *Blepharispermum pubescens* S.Moore | 30 [322] | Aerial parts | These are approximations, as the authors expressed the values in grams/kg (including the iron in the group of “macro elements”) instead of the usual mg/kg or ppm. | Asteraceae | D | S |
| *Blighia sapida* K.D.Koenig | 500 [323] | Fruit |  | Sapindaceae | D | T |
| *Blighia unijugata* Baker | 120.0 [324] | Young leaf |  | Sapindaceae | D | T |
| *Blumea lanceolaria* (Roxb.) Druce | 16.0 [325] | Leaf |  | Asteraceae | D | S |
| *Boerhavia diffusa* L. | 427 [326] | Whole plant |  | Nyctaginaceae | D | H |
| *Bolboschoenus maritimus* (L.) Palla | 1003 [327] | Tuber | Reported as *Scirpus maritimus* L. | Cyperaceae | M | H |
| Bombacopsis macrocalyx (Ducke) A.Robyns | 350 [328] | Leaf | Reported as Bombacopsis macrocalyx (Ducke) Robyns. From an area degraded by anthropic activities. | Bombacaceae | D | T |
| *Borago officinalis* L. | 85.7 [329] | Flower |  | Boraginaceae | D | H |
| *Borassus aethiopum* Mart. | 56.6 [330] | Fruit | See comment to *Dypsis lastelliana.* | Arecaceae | D | T* |
| *Boscia coriacea* Graells | 100 [331] | Aerial parts | These are approximations, as the authors expressed the values in grams/kg (including the iron in the group of “macro elements”) instead of the usual mg/kg or ppm. | Capparaceae | D | T |
| *Boscia minimifolia* Chiov. | 300 [332] | Aerial parts | These are approximations, as the authors expressed the values in grams/kg (including the iron in the group of “macro elements”) instead of the usual mg/kg or ppm. | Capparaceae | D | T |
| *Boswellia hildebrandtii* Engl. | 200 [333] | Aerial parts | These are approximations, as the authors expressed the values in grams/kg (including the iron in the group of “macro elements”) instead of the usual mg/kg or ppm. | Burseraceae | D | T |
| *Boswellia microphylla* Chiov. | 30 [334] | Aerial parts | These are approximations, as the authors expressed the values in grams/kg (including the iron in the group of “macro elements”) instead of the usual mg/kg or ppm. | Burseraceae | D | T |
| *Bothriochloa pertusa* (L.) A.Camus | 947.6 [335] | Aerial parts |  | Poaceae | M | H |
| *Brachiaria ramosa* (L.) Stapf | 1017.0 [336] | Aerial parts |  | Poaceae | M | H |
| *Brachiaria ramosa* (L.) Stapf | 1017.0 [337] | Aerial parts |  | Poaceae | M | H |
| *Brachiaria repens* Gardn. et Hubb. | 526.2 [338] | Aerial parts | The Plant List does not include *Brachiaria repens* but only *Brachiaria reptans* (L.) C.A.Gardner & C.E.Hubb | Poaceae | M | H |
| *Brachypodium* *sylvaticum* (Huds.) P.Beauv. | 180 [339] | Aerial parts | Reported as *Festuca sylvatica* Huds. | Poaceae | M | H |
| *Brachypodium* *sylvaticum* (Huds.) P.Beauv. | 430 [340] | Aerial parts |  | Poaceae | M | H |
| *Brachypodium* *sylvaticum* (Huds.) P.Beauv. | 80 [341] | Aerial parts |  | Poaceae | M | H |
| *Brachystegia eurycoma* Harms | 0.11 [342] | Seed |  | Fabaceae | D | T |
| *Brasenia schreberi* J.F.Gmel. | 27 [343] | Leaf | Aquatic. | Cabombaceae | D | H |
| *Brassica cretica* Lam. | 193.9 [344] | Leaf | Reported as *B. oleracea* L. var. *botrytis.* | Brassicaeae | D | H |
| *Brassica napus* L. | 215.3 [345] | Leaf |  | Brassicaceae | D | H |
| *Brassica nigra* (L.)K.Koch | 254 [346] | Leaf |  | Brassicaceae | D | H |
| *Brassica oleracea* L. | 10.5 [347] | Leaf | Reported as *Brassica oleracea* var. *gongylodes* L. | Brassicaceae | D | H |
| *Brassica oleracea* L. | 10.5 [348] | Leaf |  | Brassicaceae | D | H |
| *Brassica oleracea* L. | 124.2 [349] | Leaf | Described as „cabbage”. | Brassicaceae | D | H |
| *Brassica oleracea* L. | 18.4 [350] | Leaf |  | Brassicaceae | D | H |
| *Brassica oleracea* L. | 89.4 [351] | Leaf |  | Brassicaceae | D | H |
| *Brassica rapa* L. | 118 [352] | Leaf |  | Brassicaceae | D | H |
| *Brassica rapa* L. | 157.9 [353] | Leaf | Reported as *Brassica rapa* var. *rapa.* | Brassicaceae | D | H |
| *Brassica rapa* L. | 17.0 [354] | Leaf |  | Brassicaceae | D | H |
| *Brassica rapa* L. | 185 [355] | Leaf |  | Brassicaceae | D | H |
| *Brassica rapa* L. | 110.3 [356] | Root | Reported as “turnip”. | Brassicaceae | D | H |
| *Brassica rapa* L. | 118.0 [357] | Root |  | Brassicaceae | D | H |
| *Breonadia salicina* (Vahl) Hepper & J.R.I.Wood | 47.4 [358] | Seed | Reported as *Adina microcephala* (Delile) Hiern. | Rubiaceae | D | T |
| *Bretschneidera sinensis* Hemsl. | 226.5 [359] | Leaf |  | Akaniaceae | D | T |
| *Bridelia ferruginea* Benth. | 143.1 [360] | Fruit |  | Phyllanthaceae | D | T |
| *Bridelia ferruginea* Benth. | 193 [361] | Leaf |  | Phyllanthaceae | D | T |
| *Bridelia micrantha* (Hochst.) Baill. | 133.2 [362] | Fruit |  | Phyllanthaceae | D | T |
| *Bridelia micrantha* (Hochst.) Baill. | 114.5 [363] | Petiole |  | Phyllanthaceae | D | T |
| Briza minor L. | 223 [364] | Aerial parts |  | Poaceae | M | H |
| *Bromus hordeaceus* L. | 170.0 [365] | Aerial parts |  | Poaceae | M | H |
| *Bromus hordeaceus* L. | 210.0 [366] | Aerial parts |  | Poaceae | M | H |
| *Bromus rigidus* Roth. | 70 [367] | Seed |  | Poaceae | M | H |
| *Broussonetia papyrifera* (L.) L'Hér. ex Vent. | 259.4 [368] | Leaf | From Beijing Botanica Garden (control). | Moraceae | D | T |
| *Broussonetia papyrifera* (L.) L'Hér. ex Vent. | 323.7 [369] | Leaf | From hills near the Beijing Steel Factory. | Moraceae | D | T |
| *Bruguiera gymnorhiza* (L.) Lam. | 120 [370] | Bark |  | Rhizophoraceae | D | T |
| *Bruguiera gymnorhiza* (L.) Lam. | 62 [371] | Bark |  | Rhizophoraceae | D | T |
| *Bruguiera gymnorhiza* (L.) Lam. | 11 [372] | Wood | Heartwood. | Rhizophoraceae | D | T |
| *Bryophyllum pinnatum* (Lam.) Oken | 140 [373] | Whole plant | Succulent | Crassulaceae | D | H |
| *Buglossoides arvensis* (L.) I.M.Johnst. | 8.0 [374] | Aerial parts | 1600 m altitude | Boraginaceae | D | H |
| *Bupleurum scorzonerifolium* Willd. | 171 [375] | Aerial parts |  | Apiaceae | D | H |
| *Bupleurum scorzonerifolium* Willd. | 290 [376] | Aerial parts |  | Apiaceae | D | H |
| *Bupleurum trichopodum* Boiss. & Spruner | 0.7 [377] | Aerial parts | 1000 m altitude | Apiaceae | D | H |
| *Cadaba glandulosa* Forssk. | 300 [378] | Aerial parts | These are approximations, as the authors expressed the values in grams/kg (including the iron in the group of “macro elements”) instead of the usual mg/kg or ppm. | Capparaceae | D | S |
| *Cadaba glandulosa* Forssk. | 600 [379] | Aerial parts | These are approximations, as the authors expressed the values in grams/kg (including the iron in the group of “macro elements”) instead of the usual mg/kg or ppm. | Capparaceae | D | S |
| *Caesalpinia pulcherrima* (L.) Sw. | 210.0 [380] | Seed | Reported as *Caesalpinia pulcherina* L. | Fabaceae | D | S |
| *Calamagrostis* *canescens* (Weber) Roth | 160 [381] | Leaf | Reported as *Calamagrostis lanceolata* Roth | Poaceaea | M | H |
| *Calendula arvensis* M.Bieb. | 80 [382] | Seed | Reported as *Calendula arvensis* L. | Asteraceae | D | H |
| *Calendula* *officinalis* L. | 206.8 [383] | Flower |  | Asteraceae | D | H |
| *Calendula* *officinalis* L. | 79.7 [384] | Flower |  | Asteraceae | D | H |
| *Calendula officinalis* L. | 202.9 [385] | Leaf |  | Asteraceae | D | H |
| *Calendula officinalis* L. | 238.9 [386] | Leaf |  | Asteraceae | D | H |
| *Calla palustris* L. | 1231 [387] | Aerial parts | Aquatic | Araceae | M | H |
| *Calliandra eriophylla* Benth. | 162 [388] | Leaf |  | Fabaceae | D | S |
| *Callicarpa japonica* Thunb. | 152 [389] | Leaf |  | Lamiaceae | D | S |
| *Calliergon* *cordifolium* (Hedw.) Kindb. | 3070 [390] | Aerial parts | Reported as *Hypnum cordifolium* Hedw. | Amblystegiaceae | B | H |
| *Calliergonella* *cuspidate* (Hedw.) Loeske | 1600 [391] | Aerial parts | Reported as *Hypnum cuspidatum* Hedw. | Hypnaceae | B | H |
| *Calluna vulgaris* (L.) Hull | 310 [392] | Leaf |  | Ericaceae | D | S |
| *Calycanthus chinensis* (W.C.Cheng & S.Y.Chang) P.T.Li | 269.6 [393] | Leaf |  | Calycanthaceae | Mag | S |
| Camelina sativa (L.) Crantz | 125 [394] | Seed |  | Brassicaceae | D | H |
| Camelina sativa (L.) Crantz | 329 [395] | Seed |  | Brassicaceae | D | H |
| Camelina sativa (L.) Crantz | 4470 [396] | Seed |  | Brassicaceae | D | H |
| *Camellia grijsii* Hance | 191.0 [397] | Leaf |  | Theaceae | D | S |
| *Camellia nitidissima* C.W.Chi | 215.2 [398] | Leaf |  | Theaceae | D | S |
| *Camellia sinensis* (L.) Kuntze | 326 [399] | Leaf |  | Theaceae | D | T |
| *Camellia sinensis* (L.) Kuntze | 540 [400] | Leaf |  | Theaceae | D | T |
| *Camptotheca acuminata* Decne. | 326.1 [401] | Leaf |  | Cornaceae | D | T |
| *Canarium madagascariense* Engl. | 57.8 [402] | Fruit |  | Burseraceae | D | T |
| *Canarium odontophyllum* Miq. | 42.4 [403] | Fruit |  | Burseraceae | D | T |
| *Canarium odontophyllum* Miq. | 64.2 [404] | Fruit |  | Burseraceae | D | T |
| *Canavalia cathartica* Thouars | 721 [405] | Seed | Reported as *Canavalia virosa* (Roxb.) Wight &Arn. | Fabaceae | D | H |
| *Capparis spinosa* L. | 164.08 [406] | Bud | Reported as *Capparis ovata* Desf. | Capparaceae | D | Su |
| *Capsella bursa-pastoris* (L.) Medik. | 40.7 [407] | Leaf |  | Brassicaceae | D | H |
| *Capsicum annuum* L. | 299.5 [408] | Fruit |  | Solanaceae | D | H |
| *Capsicum annuum* L. | 3708 [409] | Fruit | Reported as *Capsicum* *frutescens* L. | Solanaceae | D | H |
| *Capsicum annuum* L. | 92.66 [410] | Fruit | Reported as *Capsicum frutescens* L. | Solanaceae | D | H |
| *Capsicum anuum* L. | 203 [411] | Leaf |  | Solanaceae | D | H |
| *Capsicum anuum* L. | 84 [412] | Leaf |  | Solanaceae | D | H |
| *Carapa grandiflora* Sprague | 117.9 [413] | Petiole |  | Meliaceae | D | T |
| *Cardamine hirsuta* L. | 435.2 [414] | Leaf | Reported in mmol/kg, converted by us in mg/kg. | Brassicaceae | D | H |
| *Carex vesicaria* L. | 190 [415] | Leaf |  | Cyperaceae | M | H |
| *Carex canescens* L. | 650 [416] | Leaf |  | Cyperaceae | M | H |
| Carex divulsa Stokes | 77 [417] | Aerial parts |  | Cyperaceae | M | H |
| *Carex lacustris* Willd. | 522 [418] | Aerial parts | Aquatic | Cyperaceae | M | H |
| *Carex lasiocarpa* Ehrh. | 180 [419] | Leaf |  | Cyperaceae | M | H |
| *Carex stricta* Lam. | 861 [420] | Aerial parts | Aquatic (marshes, near waters). | Cyperaceae | M | H |
| *Carex* *inflata* Huds. | 180 [421] | Leaf | Reported as *Carex rostrata* Stokes | Cyperaceae | M | H |
| *Carex* *paniculata* L. | 260 [422] | Leaf |  | Cyperaceae | M | H |
| *Carica papaya* L. | 5 [423] | Fruit | Similarly to the case of palm trees, this species is strictly technically a herbaceous one, as it does not have a woody trunk. On the other hand, books dealing with trees usually include such species as “trees”, based on size and not on anatomical structure. | Caricaceae | D | T |
| *Carica papaya* L. | 306 [424] | Leaf | Similarly to the case of palm trees, this species is strictly technically a herbaceous one, as it does not have a woody trunk. On the other hand, books dealing with trees usually include such species as “trees”, based on size and not on anatomical structure. | Caricaceae | D | T* |
| *Carpinus cordata* Blume | 128 [425] | Leaf |  | Betulaceae | D | T |
| *Carpinus japonica* Blume | 159 [426] | Leaf |  | Betulaceae | D | T |
| *Carpinus laxiflora* (Siebold & Zucc.) Blume | 152 [427] | Leaf |  | Betulaceae | D | T |
| *Carpinus putoensis* W.C.Cheng | 463.3 [428] | Leaf |  | Betulaceae | D | T |
| *Carpinus tschonoskii* Maxim. | 163 [429] | Leaf |  | Betulaceae | D | T |
| *Carthamus tinctorius* L. | 294 [430] | Leaf |  | Asteraceae | D | H |
| *Carum carvi* L. | 138 [431] | Aerial parts |  | Apiaceae | D | H |
| *Carum* *carvi* L. | 33.5 [432] | Fruits |  | Apiaceae | D | H |
| *Carum* *carvi* L. | 91.5 [433] | Fruits |  | Apiaceae | D | H |
| *Carum carvi* L. | 52.6 [434] | Leaf |  | Apiaceae | D | H |
| *Carum carvi L.* | 162 [435] | Seed |  | Apiaceae | D | H |
| *Casearia nigrescens* var. *lucida* (Tul.) Sleumer | 40.0 [436] | Fruit | Reported as *Casearia lucida* Tul*.* | Salicaceae | D | T |
| *Cassia fistula* L. | 559 [437] | Fruit |  | Fabaceae | D | T |
| *Cassia* *occidentalis* L. | 23.77 [438] | Leaf | Reported as *Senna occidentalis* (L.) Link | Fabaceae | D | S |
| *Cassipourea congensis* R.Br. ex DC. | 67.0 [439] | Fruit |  | Rhizophoraceae | D | H |
| *Cassipourea ruwensorensis* (Engl.) Alston | 55.4 [440] | Leaf | Young leaf | Rhizophoraceae | D | T |
| *Cassipourea ruwensorensis* (Engl.) Alston | 93.4 [441] | Leaf |  | Rhizophoraceae | D | T |
| *Castanea crenata* Siebold & Zucc. | 12 [442] | Bark |  | Fagaceae | D | T |
| *Castanea crenata* Siebold & Zucc. | 44 [443] | Bark |  | Fagaceae | D | T |
| *Castanea crenata* Siebold & Zucc. | 107 [444] | Leaf |  | Fagaceae | D | T |
| *Castanea crenata* Siebold & Zucc. | 1. 2 [445] | Wood | Sapwood | Fagaceae | D | T |
| *Castanea sativa* Mill. | 220 [446] | Bark |  | Fagaceae | D | T |
| *Catharinea undulata* (Hedw.) F. Weber & D. Mohr | 50 [447] | Aerial parts |  | Polytrichaceae | B | H |
| *Catharinea undulata* (Hedw.) F. Weber & D. Mohr | 69 [448] | Aerial parts |  | Polytrichaceae | B | H |
| *Catunaregam* *spinose* (Thunb.) Tirveng. | 14.5 [449] | Fruit | Results apparently reported on a fresh matter basis (no dry matter mentioned, and 82.8% moisture reported). We therefore made the calculations on a dry matter basis. Reported as *Randia dumetorum* (Retz.) Lam. | Rubiaceae | D | T |
| *Catunaregam* *tomentosa* (Blume ex DC.) Tirveng. | 72 [450] | Leaf | Reported as “leaves (or shoots)”. Sandy podzolic soil (pH 5.0); Reported as *Randia dasycarpa* (Kurz) Bakh.f. | Rubiaceae | D | T |
| *Cayratia trifolia* (L.) Domin | 21.6 [451] | Leaf |  | Vitaceae | D | V |
| Cecropia ficifolia Warb. ex Snethl. | 319 [452] | Leaf | Reported as Cecropia ficifolia Smeth. From an area degraded by anthropic activities. | Urticaceae | D | T |
| Cecropia sciadophylla Mart. | 247 [453] | Leaf | From an area degraded by anthropic activities. | Urticaceae | D | T |
| *Cedrus deodara* (Roxb. ex D.Don) G.Don | 390 [454] | Leaf | From an urban area (Madrid). | Pinaceae | G | T |
| *Cedrus libani* A. Rich. | 1.2 [455] | Leaf | 1000 m altitude. | Pinaceae | G | T |
| *Cedrus libani* A. Rich. | 613 [456] | Leaf |  | Pinaceae | G | T |
| Ceiba pentandra (L.) Gaertn. | 800.0 [457] | Aerial parts | Reported as Ceiba patendra. Destalked. | Malvaceae | D | T |
| *Celastrus paniculatus* Willd. | 1750 [458] | Stem |  | Celastraceae | D | V |
| *Celosia argentea* L. | 1000.0 [459] | Aerial parts | Destalked. | Amaranthaceae | D | H |
| *Celosia argentea* L. | 200.0 [460] | Leaf |  | Amaranthaceae | D | H |
| *Celtis africana* Burm.f | 68.8 [461] | Leaf |  | Cannabaceae | D | T |
| *Celtis africana* Burm.f. | 133.9 [462] | Young leaf |  | Cannabaceae | D | T |
| *Celtis australis* L. | 4.0 [463] | Leaf | 1600 m altitude | Cannabaceae | D | T |
| *Celtis gomphophylla* Baker | 432.5 [464] | Buds (leaf) | Reported as *Celtis durandii* Engl. | Ulmaceae | D | T |
| *Celtis gomphophylla* Baker | 192.5 [465] | Flower | Reported as *Celtis durandii* Engl. | Cannabaceae | D | T |
| *Celtis gomphophylla* Baker | 329.4 [466] | Fruit | Reported as *Celtis durandii* Engl. | Cannabaceae | D | T |
| *Celtis gomphophylla* Baker | 152.5 [467] | Leaf | Reported as *Celtis durandii* Engl. | Ulmaceae | D | T |
| *Celtis gomphophylla* Baker | 313.7 [468] | Seed | Reported as *Celtis durandii* Engl. | Ulmaceae | D | T |
| *Celtis gomphophylla* Baker | 139.9 [469] | Unripe fruit | Reported as *Celtis durandii* Engl. | Ulmaceae | D | T |
| *Celtis gomphophylla* Baker | 207.5 [470] | Young leaf | Reported as *Celtis durandii* Engl. | Ulmaceae | D | T |
| *Celtis pallida* Torr. | 312 [471] | Leaf |  | Cannabaceae | D | T |
| *Centaurea eriophora* L. | 40 [472] | Seed |  | Asteraceae | D | H |
| Centaurea exarata Boiss. ex Coss. | 82.6 [473] | Aerial parts |  | Asteraceae | D | H |
| *Centaurea nigra* L. | 109.6 [474] | Shoot | From an area around an abandoned mine. | Asteraceae | D | H |
| *Centaurea nigra* L. | 72.7 [475] | Shoot | From an area around an abandoned mine. | Asteraceae | D | H |
| *Centaurea virgata* Lam. | 3470 [476] | Whole plant | From a copper and iron mine area. | Asteraceae | D | H |
| *Centaurium erythraea* Rafn (Gentianaceae) | 510 [477] | Aerial parts |  | Gentianaceae | D | H |
| *Centaurium erythraea* Rafn (Gentianaceae) | 90 [478] | Aerial parts |  | Gentianaceae | D | H |
| *Centaurium erythraea* Rafn (Gentianaceae) | 386.1 [479] | Flower |  | Gentianaceae | D | H |
| *Centaurium erythraea* Rafn (Gentianaceae) | 49.9 [480] | Root |  | Gentianaceae | D | H |
| *Centella* *asiatica* (L.) Urb | 19.56 [481] | Leaf |  | Apiaceae |  |  |
| *Centella asiatica* (L.) Urb. | 838 [482] | Leaf |  | Apiaceae | D | H |
| *Centella asiatica* (L.) Urb. | 876.9 [483] | Leaf | From a paper mill contaminated area. | Apiaceae | D | H |
| *Centella asiatica* (L.) Urb. | 2048.5 [484] | Root | From a paper mill contaminated area. | Apiaceae | D | H |
| *Centella asiatica* (L.) Urb. | 1868 [485] | Stem | From a paper mill contaminated area. | Apiaceae | D | H |
| *Centella asiatica* (L.)Urb. | 357.4 [486] | Leaf | Reported as *Centella asiatica* L. | Apiaceae | D | H |
| *Cephalotaxus harringtonii var. nana* (Nakai) Rehder | 84 [487] | Leaf |  | Taxaceae | G | T |
| *Cephalotaxus oliveri* Mast. | 160.3 [488] | Leaf |  | Taxaceae | G | T |
| *Cephalotus follicularis* Labill. | 51.3 [489] | Leaf | Carnivorous. Trap leaves. | Cephalotaceae | D | H |
| *Cerastium semidecandrum* L. | 786.8 [490] | Leaf | Reported in mmol/kg, converted by us in mg/kg. | Caryophyllaceae | D | H |
| *Cerasus jamasakura* (Siebold ex Koidz.) H.Ohba | 80 [491] | Leaf | Reported as *Prunus jamasakura* Siebold. | Rosaceae | D | T |
| Ceratonia siliqua L. | 193 [492] | Aerial parts |  | Fabaceae | D | T |
| *Ceratophyllum demersum* L. | 546 [493] | Aerial parts | Aquatic | Ceratophyllaceae | D | H |
| *Cercidiphyllum japonicum* Siebold & Zucc. ex J.J.Hoffm. & J.H.Schult.bis | 210.8 [494] | Leaf |  | Cercidiphyllaceae | D | T |
| *Chaetachme aristata* Planch. | 162.0 [495] | Fruit |  | Ulmaceae | D | T |
| *Chaetachme aristata* Planch. | 20.7 [496] | Leaf | Young leaf | Ulmaceae | D | T |
| *Chaetachme aristata* Planch. | 41.9 [497] | Leaf |  | Ulmaceae | D | T |
| Chaetopogon fasciculatus (Link.) Hayek | 305 [498] | Aerial parts |  | Poaceae | M | H |
| *Chamaecrista absus* (L.) H.S.Irwin & Barneby | 11613 [499] | Seed |  | Fabaceae | D | H |
| *Chamaecyparis formosensis* Matsum. | 445.5 [500] | Leaf |  | Cupressaceae | G | T |
| *Chamaecyparis obtusa* (Siebold & Zucc.) Endl. | 50 [501] | Bark |  | Cupressaceae | G | T |
| *Chamaecyparis obtusa* (Siebold & Zucc.) Endl. | 58 [502] | Bark |  | Cupressaceae | G | T |
| *Chamaecyparis obtusa* (Siebold & Zucc.) Endl. | 0 [503] | Wood | Under LOQ. | Cupressaceae | G | T |
| *Chamaemelum nobile* (L.) All. | 159.1 [504] | Flower | Reported as *Anthemis nobilis* L. | Asteraceae | D | H |
| Chamaerops humilis L. | 118 [505] | Aerial parts | See comment to *Dypsis lastelliana.* | Arecaceae | M | T* |
| *Changiostyrax dolichocarpus* (C.J.Qi) Tao Chen | 298.2 [506] | Leaf | Reported as *Sinojackia dolichocarpa* C.J.Qi. | Styracaceae | D | T |
| *Chengiopanax sciadophylloides* (Franch. & Sav.) C.B.Shang & J.Y.Huang | 105 [507] | Leaf | Reported as *Acanthopanax sciadophylloides* Franch. & Sav.. | Araliaceae | D | T |
| *Chenopodium album* L. | 117.8 [508] | Leaf |  | Amaranthaceae | D | H |
| *Chenopodium* *album* L. | 18.76 [509] | Leaf |  | Amaranthaceae | D | H |
| *Chenopodium album* L. | 25 [510] | Leaf |  | Amaranthaceae | D | H |
| *Chenopodium album* L. | 288 [511] | Leaf |  | Amaranthaceae | D | H |
| *Chenopodium album* L. | 491.8 [512] | Leaf |  | Amaranthaceae | D | H |
| *Chenopodium album* L. | 78.17 [513] | Leaf |  | Amaranthaceae | D | H |
| Chenopodium album L. | 781.7 [514] | Leaf | Minimum from a set of 13 values. | Amaranthaceae | D | H |
| *Chenopodium album* L. | 87.80 [515] | Leaf |  | Amaranthaceae | D | H |
| Chenopodium album L. | 878 [516] | Leaf | Maximum from a set of 13 values. | Amaranthaceae | D | H |
| *Chenopodium album* L. | 608.24 [517] | Root | From a paper mill contaminated area. | Amaranthaceae | D | H |
| *Chenopodium album* L. | 844.2 [518] | Stem | From a paper mill contaminated area. | Amaranthaceae | D | H |
| *Chenopodium berlandieri* Moq. | 91.33 [519] | Leaf | Reported as Chenopodium berlandieri ssp. nuttalliae (Saff.) H.D.Wilson & Heiser. | Amaranthaceae | D | H |
| Chenopodium berlandieri Moq. | 913.3 [520] | Leaf | Reported as Chenopodium berlandieri ssp. nuttalliae (Saff.) H.D.Wilson & Heiser. | Amaranthaceae | D | H |
| *Chenopodium berlandieri* Moq. | 92.33 [521] | Leaf | Reported as Chenopodium berlandieri ssp. nuttalliae (Saff.) H.D.Wilson & Heiser. | Amaranthaceae | D | H |
| Chenopodium berlandieri Moq. | 923.3 [522] | Leaf | Reported as Chenopodium berlandieri ssp. nuttalliae (Saff.) H.D.Wilson & Heiser. | Amaranthaceae | D | H |
| *Chenopodium berlandieri* var. *bushianum* (Aellen) Cronquist | 84.03 [523] | Leaf | Reported as *Chenopodium bushianum* Aellen*.* | Amaranthaceae | D | H |
| *Chenopodium berlandieri* var. *bushianum* (Aellen) Cronquist | 840.3 [524] | Leaf | Reported as *Chenopodium bushianum* Aellen*.* | Amaranthaceae | D | H |
| *Chenopodium foliosum* Asch. | 13.0 [525] | Whole plant |  | Amaranthaceae | D | H |
| *Chenopodium giganteum* D.Don | 80.2 [526] | Leaf |  | Amaranthaceae | D | H |
| Chenopodium giganteum D.Don | 802.3 [527] | Leaf | Minimum from a set of 4 values | Amaranthaceae | D | H |
| *Chenopodium giganteum* D.Don | 81 [528] | Leaf |  | Amaranthaceae | D | H |
| Chenopodium giganteum D.Don | 820.7 [529] | Leaf | Maximum from a set of 4 values. | Amaranthaceae | D | H |
| *Chenopodium murale* L. | 75.60 [530] | Leaf |  | Amaranthaceae | D | H |
| Chenopodium murale L. | 756.0 [531] | Leaf |  | Amaranthaceae | D | H |
| *Chenopodium murale* L. | 81.53 [532] | Leaf |  | Amaranthaceae | D | H |
| *Chenopodium murale* L. | 590 [533] | Seed |  | Amaranthaceae | D | H |
| Chenopodium opulifolium Schrad. ex W.D.J.Koch & Ziz | 815.3 [534] | Leaf | Reported as Chenopodium ugandae (Aellen) Aellen. | Amaranthaceae | D | H |
| Chenopodium quinoa Willd. | 792.0 [535] | Leaf | Minimum from a set of 18 values. | Amaranthaceae | D | H |
| Chenopodium quinoa Willd. | 894.0 [536] | Leaf | Maximum from a set of 18 values. | Amaranthaceae | D | H |
| *Chenopodium quinoa* Willd. | 79.2 [537] | Leaf |  | Amaranthaceae | D | H |
| *Chenopodium quinoa* Willd. | 89.4 [538] | Leaf |  | Amaranthaceae | D | H |
| Chenopodium quinoa Willd. | 20 [539] | Seed |  | Amaranthaceae | D | H |
| Chenopodium quinoa Willd. | 81 [540] | Seed |  | Amaranthaceae | D | H |
| *Chionanthus africanus* (Knobl.) Stearn | 75.5 [541] | Fruit | Reported as *Linociera johnsonii* Baker | Oleaceae | D | T |
| *Choerospondias* *axillaris* (Roxb.) B.L.Burtt & A.W.Hill | 1090 [542] | Fruit | Reported as *Spondias axillaris* Roxb. | Anacardiaceae | D | T |
| *Chondrilla juncea* L. | 240 [543] | Whole plant | From a copper and iron mine area. | Asteraceae | D | H |
| *Christella parasitica* (L.) H. Lév. *ex* Holttum | 620 [544] | Leaf | Reproductive stage | Thelypteridaceae | P | H |
| *Christella parasitica* (L.) H. Lév. *ex* Holttum | 820 [545] | Leaf | Vegetative stage | Thelypteridaceae | P | H |
| *Christella dentata* (Forssk.) Brownsey & Jermy | 179.89 [546] | Aerial parts | Reported as *Thelypteris dentata* (Forssk.) E.P. St. John. | Thelypteridaceae | P | H |
| *Chrysobalanus icaco* subsp. *atacorensis* (A.Chev.) *F.White* | 504.6 [547] | Seed | Reported as *Chrysobalanus atacorensis* A.Chev. | Chrysobalanaceae | D | T |
| *Chrysophyllum boivinianum* (Pierre) Baehni | 47.0 [548] | Fruit |  | Sapotaceae | D | T |
| *Chrysophyllum boivinianum* (Pierre) Baehni | 70.3 [549] | Leaf |  | Sapotaceae | D | T |
| Chrysophyllum sanguinolentum (Pierre) Baehni | 725 [550] | Leaf | From an area degraded by anthropic activities. | Sapotaceae | D | T |
| *Chrysopogon* *aciculatus* (Retz.) Trin. | 31 [551] | Leaf | Reported as “leaves (or shoots)”. Sandy podzolic soil (pH 5.0) | Poaceae | M | H |
| Cicer arietinum L. | 41.7 [552] | Seed |  | Fabaceae | D | H |
| *Cichorium intybus* L. | 24.6 [553] | Leaf |  | Asteraceae | D | H |
| *Cichorium intybus* L. | 2390 [554] | Seed |  | Asteraceae | D | H |
| *Cinnamomum camphora* (L.) J.Presl | 14 [555] | Bark |  | Lauraceae | Mag | T |
| *Cinnamomum camphora* (L.) J.Presl | 8.7 [556] | Bark |  | Lauraceae | Mag | T |
| *Cinnamomum camphora* (L.) J.Presl | 3.5 [557] | Wood | Heartwood | Lauraceae | Mag | T |
| *Cinnamomum japonicum* Siebold | 195.5 [558] | Leaf | This is reported by The Plant List as unresolved and illegitimate. | Lauraceae | Mag | T |
| *Cinnamomum platyphyllum* (Diels) C.K.Allen | 303.5 [559] | Leaf |  | Lauraceae | Mag | T |
| *Cinnamomum verum* J.Presl. | 123.65 [560] | Bark | Reported as *Cinnamomum zeylanicum.* | Lauraceae | Mag | T |
| *Cinnamomum verum* J.Presl. | 129 [561] | Bark | Reported as *Cinnamomum zeylanicum.* | Lauraceae | Mag | T |
| *Cirsium leucocephalum* subsp. *penicillatum* (K.Koch) Greuter | 275 [562] | Whole plant | Reported as *Cirsium lappaceum.* From a copper and iron mine area. | Asteraceae | D | H |
| *Cirsium oleraceum* (L.) Scop. | 116 [563] | Aerial parts |  | Asteraceae | D | H |
| *Cirsium oleraceum* (L.) Scop. | 137 [564] | Aerial parts |  | Asteraceae | D | H |
| *Cirsium sorocephalum* Fisch. & C.A.Mey. | 2900 [565] | Whole plant | Reported as *Cirsium congestum* Fisch. & C.A.Mey. ex DC. From a copper and iron mine area. | Asteraceae | D | H |
| *Cissus cornifolia* (Baker) Planch. | 125.0 [566] | Root |  | Vitaceae | D | T |
| *Cissus populnea* Guill. & Perr. | 10.5 [567] | Stem |  | Vitaceae | D | V |
| *Cissus populnea* Guill. & Perr. | 16.7 [568] | Stem |  | Vitaceae | D | V |
| *Cissus quadrangularis* L. | 532 [569] | Leaf |  | Vitaceae | D | H |
| *Cissus quadrangularis* L. | 967.5 [570] | Leaf |  | Vitaceae | D | V |
| Cistus albidus L. | 390 [571] | Aerial parts |  | Cistaceae | D | S |
| *Cistus creticus* L. | 3.8 [572] | Leaf | 1000 m altitude | Cistaceae | D | H |
| Cistus crispus L. | 979 [573] | Aerial parts |  | Cistaceae | D | S |
| Cistus ladanifer L. | 724 [574] | Aerial parts |  | Cistaceae | D | S |
| Cistus monspeliensis L. | 564 [575] | Aerial parts |  | Cistaceae | D | S |
| Cistus populifolius L. | 341 [576] | Aerial parts |  | Cistaceae | D | S |
| Cistus salviifolius L. | 266 [577] | Aerial parts |  | Cistaceae | D | S |
| *Citrullus colocynthis* (L.) Schrad. | 779 [578] | Fruit |  | Cucurbitaceae | D | H |
| *Citrullus ecirrhosus* Cogn. | 494.0 [579] | Seed |  | Cucurbitaceae | D | H |
| *Citrullus lanatus* (Thunb.) Matsum.&Nakai. | 4.7 [580] | Fruit |  | Cucurbitaceae | D | H |
| *Citrus aurantiifolia* (Christm.) Swingle | 35.1 [581] | Leaf | Reported as *Citrus × macrophylla* Wester*.* | Rutaceae | D | T |
| *Citrus aurantiifolia* (Christm.) Swingle | 40.2 [582] | Leaf | Reported as *Citrus × macrophylla* Wester*.* | Rutaceae | D | T |
| *Citrus japonica* Thunb. | 5.5 [583] | Fruit | Reported as *Fortunella margarita* (Lour.) Swingle. | Rutaceae | D | T |
| *Citrus limon* (L.) Osbeck | 5 [584] | Fruit | Reported as *Citrus limon* (L.)Burm.f. | Rutaceae | D | T |
| *Citrus volkameriana* Pasq. | 32.7 [585] | Leaf |  | Rutaceae | D | T |
| *Cladium* *mariscus* (L.) Pohl | 120 [586] | Leaf |  | Cyperaceae | M | H |
| *Clausena anisata* (Willd.)Hook.f. ex Benth | 127.3 [587] | Leaf |  | Rutaceae | D | T |
| *Clausena anisata* (Willd.) Hook.f. ex Benth. | 85.6 [588] | Leaf | Young leaf. | Rutaceae | D | T |
| *Cleistogenes squarrosa* (Trin. ex Ledeb.)Keng | 338.3 [589] | Leaf | From hills near the Beijing Steel Factory. | Poaceae | M | H |
| *Cleome gynandra* L. | 14.3 [590] | Leaf |  | Cleomaceae | D | H |
| *Clerodendrum infortunatum* L. | 1100 [591] | Leaf |  | Lamiaceae | D | S |
| *Clethra barbinervis* Siebold & Zucc. | 135 [592] | Leaf |  | Clethraceae | D | T |
| *Cocos nucifera* L. | 21.4 [593] | Fruit | See comment to *Dypsis lastelliana*. Pod (husk). | Arecaceae | M | T* |
| *Coix lacryma-jobi* L. | 5.6 [594] | Seed |  | Poaceae | M | H |
| *Coldenia greggii* (Torr. & A.Gray) A.Gray | 257 [595] | Leaf | Although in the source paper the table states *Cassia greggii*, the abstract and the body of the text mention *Coldenia greggii* (and *Cassia wislizeni*); it seems therefore that *Coldenia* is the correct genus. | Boraginaceae | D | S |
| *Colocasia* *esculenta* (L.) Schott | 18.22 [596] | Leaf |  | Araceae | M | H |
| *Colocasia esculenta* (L.) Schott | 2056.3 [597] | Leaf | From a paper mill contaminated area. | Araceae | M | H |
| *Colocasia esculenta* (L.) Schott | 2275 [598] | Leaf | Reported as *Colocasia esculenta* L. | Araceae | M | H |
| *Colocasia esculenta* (L.) Schott | 316 [599] | Leaf | Reported as *Colocasia esculenta* L. | Araceae | M | H |
| *Colocasia esculenta* (L.) Schott | 700.0 [600] | Leaf | Reported as *Colocasia esculenta* L. | Araceae | M | H |
| *Colocasia esculenta* (L.) Schott | 547.8 [601] | Root |  | Araceae | M | H |
| Colocasia esculenta (L.) Schott | 49.2 [602] | Shoots |  | Araceae | M | H |
| *Colocasia esculenta* (L.) Schott | 1216.8 [603] | Stem |  | Araceae | M | H |
| *Colocasia esculenta* (L.) Schott. | 142.5 [604] | Leaf |  | Araceae | M | H |
| *Comarum palustre* L. | 350 [605] | Aerial parts | Semi-aquatic. | Rosaceae | D |  |
| *Commelina africana* L. | 1500 [606] | Leaf |  | Commelinaceae | M | H |
| *Commelina benghalensis* L. | 230 [607] | Leaf |  | Commelinaceae | M | H |
| *Commiphora erythraea* (Ehrenb.) Engl. | 500 [608] | Aerial parts | These are approximations, as the authors expressed the values in grams/kg (including the iron in the group of “macro elements”) instead of the usual mg/kg or ppm. | Burseraceae | D | T |
| *Condalia lycioides* (A.Gray) Weberb. | 193 [609] | Leaf |  | Rhamnaceae | D | S |
| *Convallaria majalis* L. | 41 [610] | Whole plant | Mean = 57 in this publication. Pollution-free area. | Asparagaceae | M | H |
| *Convallaria majalis* L. | 76 [611] | Whole plant | Mean = 57 in this publication. Pollution-free area. | Asparagaceae | M | H |
| *Corchorus capsularis* L. | 580.3 [612] | Leaf |  | Malvaceae | D | H |
| Corchorus olitorius L. | 274.2 [613] | Aerial parts | Destalked. | Malvaceae | D | H |
| *Corchorus tridens* L*.* | 6.3 [614] | Leaf |  | Malvaceae | D | H |
| *Corchorus trilocularis* L. | 752 [615] | Leaf |  | Malvaceae | D | H |
| *Cordia africana* Lam. | 194.6 [616] | Flower | Reported as *Cordia abyssinica.* | Boraginaceae | D | T |
| *Cordia africana* Lam. | 1235.7 [617] | Fruit | Reported as *Cordia abyssinica.* | Boraginaceae | D | T |
| *Cordia africana* Lam. | 70.9 [618] | Leaf | Reported as *Cordia abyssinica*. Young leaf. | Boraginaceae | D | T |
| *Cordia millenii* Baker | 42.00 [619] | Fruit |  | Boraginaceae | D | T |
| *Cordia parvifolia* A.DC. | 285 [620] | Leaf |  | Boraginaceae | D | S |
| *Cordia sinensis* Lam. | 300 [621] | Aerial parts | These are approximations, as the authors expressed the values in grams/kg (including the iron in the group of “macro elements”) instead of the usual mg/kg or ppm, and thus the number of significant digits was reduced. | Boraginaceae | D | T |
| *Cordia sinensis* Lam. | 600 [622] | Aerial parts | These are approximations, as the authors expressed the values in grams/kg (including the iron in the group of “macro elements”) instead of the usual mg/kg or ppm, and thus the number of significant digits was reduced. | Boraginaceae | D | T |
| *Cordia sinensis* Lam. | 900 [623] | Aerial parts | These are approximations, as the authors expressed the values in grams/kg (including the iron in the group of “macro elements”) instead of the usual mg/kg or ppm, and thus the number of significant digits was reduced. | Boraginaceae | D | T |
| *Coriandrum* *sativum* L. | 38.5 [624] | Fruit |  | Apiaceae | D | H |
| *Coriandrum sativum* L. | 83.8 [625] | Fruit |  | Apiaceae | D | H |
| *Coriandrum sativum* L. | 8424 [626] | Fruit |  | Apiaceae | D | H |
| *Coriandrum sativum* L. | 411.8 [627] | Leaf |  | Apiaceae | D | H |
| *Cornus capitata* Wall. | 274 [628] | Leaf | Reported as *Dendrobenthamia capitata* var. *emeiensi*. | Cornaceae | D | T |
| *Corrigiola litoralis* subsp. *telephiifolia* (Pourr.) Briq. | 235 [629] | Aerial parts | Reported as *Corrigiola telephiifolia* Pourr. | Molluginaceae | D | H |
| *Corylus avellana* L. | 140 [630] | Leaf |  | Betulaceae | D | S |
| *Corylus avellana* L. | 4.0 [631] | Seed |  | Betulaceae | D | T |
| *Corylus chinensis* Franch. | 286.6 [632] | Leaf |  | Betulaceae | D | T |
| *Corymbia citriodora* (Hook.) K.D.Hill & L.A.S.Johnson | 501 [633] | Leaf | Reported as *Eucalyptus citriodora* Hook. | Myrtaceae | D | T |
| *Cotinus coggygria* Scop*.* | 156.9 [634] | Leaf | From Beijing Botanica Garden (control). | Anacardiaceae | D | T |
| *Cotinus coggygria* Scop*.* | 277.7 [635] | Leaf | From hills near the Beijing Steel Factory. | Anacardiaceae | D | T |
| *Cousinia bijarensis* Rech.f. | 907.5 [636] | Whole plant | From a copper and iron mine area. | Asteraceae | D | H |
| *Crassocephalum crepidioides* (Benth.) S.Moore | 1.9 [637] | Aerial parts | Stems and leaves. | Asteraceae | D | H |
| *Crataegus monogyna* Jacq. | 721.6 [638] | Flower | 255 mg/kg reported by Stef DS *et al.* 2010, but herbal parts not stated. Inflorescence. | Rosaceae | D | T |
| *Crataegus pinnatifida* Bunge. | 8.0 [640] | Fruit |  | Rosaceae | D | T |
| *Cryptocarya thouvenotii* (Danguy) Kosterm. | 33.5 [641] | Leaf |  | Lauraceae | Mag | T |
| *Cryptomeria japonica* (Thunb. ex L.f.) D.Don | 37 [642] | Bark |  | Cupressaceae | G | T |
| *Cryptomeria japonica* (Thunb. ex L.f.) D.Don | 460 [643] | Bark |  | Cupressaceae | G | T |
| *Cryptomeria japonica* (Thunb. ex L.f.) D.Don | 52 [644] | Leaf |  | Cupresaceae | G | T |
| *Cryptomeria japonica* (Thunb. ex L.f.) D.Don | 3.7 [645] | Wood | Sapwood. | Cupressaceae | G | T |
| *Cryptomeria japonica* (Thunb. ex L.f.) D.Don | 9.3 [646] | Wood | Heartwood. | Cupressaceae | G | T |
| *Cryptomeria* *japonica* (Thunb. ex L.f.) D.Don | 1.2 [647] | Wood | Sapwood. Values expressed in mmol/kg, converted to mg/kg.  Reported as *Cryptomeria japonica* D. Don | Cupressaceae | G | T |
| *Cryptomeria* *japonica* (Thunb. ex L.f.) D.Don | 1.6 [648] | Wood | Sapwood. Values expressed in mmol/kg, converted to mg/kg. Reported as *Cryptomeria japonica* D. Don | Cupressaceae | G | T |
| *Cryptomeria* *japonica* (Thunb. ex L.f.) D.Don | 1.7 [649] | Wood | Heartwood. Values expressed in mmol/kg, converted to mg/kg. Reported as *Cryptomeria japonica* D. Don | Cupressaceae | G | T |
| *Cryptomeria* *japonica* (Thunb. ex L.f.) D.Don | 4.4 [650] | Wood | Heartwood. Values expressed in mmol/kg, converted to mg/kg. Reported as *Cryptomeria japonica* D. Don | Cupressaceae | G | T |
| *Cucumis melo* Linn*.* | 1010 | Fruit |  | Cucurbitaceae | D | H |
| *Cucumis sativus* L. | 112.8 [652] | Fruit | Described as „cucumber”. It is a “herbaceous vine”, therefore we considered it as herbaceous and not a (woody) vine. | Cucurbitaceae | D | H |
| *Cucurbita maxima* Duch*.* | 360.5 [653] | Leaf |  | Cucurbitaceae | D | H |
| *Cucurbita maxima* Duchesne | 15.9 [654] | Leaf |  | Cucurbitaceae | D | H |
| *Cucurbita maxima* Duchesne | 269 [655] | Seed |  | Cucurbitaceae | D | H |
| *Cucurbita maxima* Duchesne | 407 [656] | Seed |  | Cucurbitaceae | D | H |
| *Cucurbita pepo* L. | 19.2 [657] | Flower |  | Cucurbitaceae | D | H |
| *Cucurbita pepo* L. | 65.2 [658] | Leaf |  | Cucurbitaceae | D | H |
| *Cuminum cyminum* L. | 482 [659] | Fruit |  | Apiaceae | D | H |
| *Cuminum cyminum* L. | 824.72 [660] | Fruit |  | Apiaceae | D | H |
| *Cuminum cyminum* L. | 129 [661] | Seed |  | Apiaceae | D | H |
| *Cuminum cyminum* L. | 1690 [662] | Seed | Median 310 in this study basede on 20 samples. | Apiaceae | D | H |
| *Cuminum cyminum* L. | 190 [663] | Seed | Median 310 in this study basede on 20 samples. | Apiaceae | D | H |
| *Cuminum cyminum* L. | 2000 [664] | Seed |  | Apiaceae | D | H |
| *Cuminum cyminum* L. | 27 [665] | Seed |  | Apiaceae | D | H |
| *Cuminum cyminum* L. | 310 [666] | Seed | Median value of 20 different samples. | Apiaceae | D | H |
| *Cuminum nigrum* L. | 1726 [667] | Fruit | Not included in The Plant List. | Apiaceae | D | H |
| *Cunninghamia lanceolata* (Lamb.) Hook. | 185.1 [668] | Leaf | Reported as *Cunninghamia unicanaliculata* D.Y.Wang & H.L.Liu. | Cupressaceae | G | T |
| *Cupressus arizonica* Greene | 35.2 [669] | Whole plant |  | Cupressaceae | G | T |
| *Cupressus chengiana* S.Y.Hu | 486.6 [670] | Leaf |  | Cupressaceae | G | T |
| *Cupressus lusitanica* Mill. | 260 [671] | Leaf | Reported as *Cupressus lindleyi* Klotzsch ex Endl. From a mine contaminated area. | Cupresssaceae | G | T |
| *Cupressus lusitanica* Mill. | 95 [672] | Leaf | Reported as *Cupressus lindleyi* Klotzsch ex Endl. From a mine contaminated area. | Cupresssaceae | G | T |
| *Cupressus sempervirens* L. | 122 [673] | Bark | Non-polluted area. Mean: 250 | Cupressaceae | G | T |
| *Cupressus sempervirens* L. | 1585 [674] | Bark | Polluted (urban) area. | Cupressaceae | G | T |
| *Cupressus sempervirens* L. | 382 [675] | Bark | Non-polluted area. Mean: 250 | Cupressaceae | G | T |
| *Cupressus sempervirens* L. | 62 [676] | Bark | Polluted (urban) area. | Cupressaceae | G | T |
| *Cupressus sempervirens* L. | 170 [677] | Leaf |  | Cupressaceae | G | T |
| *Cupressus sempervirens* L. | 194 [678] | Leaf | Kinetin (20 ppm) and Fe (40 ppm) applied experimentally to the plants (foliar spray). | Cupressaceae | G | T |
| *Cupressus sempervirens* L. | 204 [679] | Leaf | Fe applied experimentally to the plants (20 ppm, ferrous sulphate as foliar spray). | Cupressaceae | G | T |
| *Cupressus sempervirens* L. | 246 [680] | Leaf | Fe applied experimentally to the plants (40 ppm, ferrous sulphate as foliar spray). | Cupressaceae | G | T |
| *Cupressus sempervirens* L. | 290 [681] | Leaf | Kinetin (40 ppm) and Fe (20 ppm) applied experimentally to the plants (foliar spray). | Cupressaceae | G | T |
| *Cupressus sempervirens* L. | 392 [682] | Leaf | Kinetin (20 ppm) and Fe (20 ppm) applied experimentally to the plants (foliar spray). | Cupressaceae | G | T |
| *Cupressus sempervirens* L. | 410 [683] | Leaf | From an urban area (Madrid). | Cupressaceae | G | T |
| *Cupressus sempervirens* L. | 486 [684] | Leaf | Kinetin (40 ppm) and Fe (40 ppm) applied experimentally to the plants (foliar spray). | Cupressaceae | G | T |
| *Cupressus sempervirens* L. | 548 [685] | Leaf | Kinetin applied experimentally to the plants (20 ppm, foliar spray). | Cupressaceae | G | T |
| *Cupressus sempervirens* L. | 622 [686] | Leaf | Kinetin applied experimentally to the plants (40 ppm, foliar spray). | Cupressaceae | G | T |
| *Curcuma longa* L. | 800 [687] | Root |  | Zingiberaceae | M | H |
| *Cyanthillium cinereum* (L.) H.Rob. | 1184.2 [688] | Leaf |  | Asteraceae | D | H |
| *Cyanthillium cinereum* (L.) H.Rob. | 387.79 [689] | Root |  | Asteraceae | D | H |
| [*Cyanus segetum* Hill](http://www.theplantlist.org/tpl1.1/record/gcc-28753). | 181.4 [690] | Flower | Reported as *Centaurea cyanus* L. | Asteraceae | D | H |
| *Cyathea gibbosa* (Klotzsch) Domin | 247.8 [691] | Aerial parts | Adult plants. The „treeness” of tree ferns may be as debatable as the „treeness” of palm trees, but considering the size and common language they may be regarded as trees. | Cyatheaceae | P | T* |
| *Cyathea gibbosa* (Klotzsch) Domin | 107.2 [692] | Aerial parts | Juvenile plants. The “treeness” of tree ferns may be as debatable as the “treeness” of palm trees, but considering the size and common language they may be regarded as trees. | Cyatheaceae | P | T* |
| *Cydonia oblonga* Mill. | 4.3 [693] | Fruit |  | Rosaceae | D | T |
| *Cylindropuntia imbricata* (Haw.) F.M.Knuth | 131 [694] | Fruit | Reported as *Opuntia imbricata* (Haw.) DC*.* Succulent. | Cactaceae | D | S |
| *Cylindropuntia imbricata* (Haw.) F.M.Knuth | 41 [695] | Stem | Reported as *Opuntia imbricata* (Haw.) DC*.* Succulent. | Cactaceae | D | S |
| *Cylindropuntia leptocaulis* (DC.) F.M.Knuth | 88 [696] | Fruit | Reported as *Opuntia leptocaulis* DC*.* Succulent. | Cactaceae | D | S |
| *Cylindropuntia leptocaulis* (DC.) F.M.Knuth | 59 [697] | Stem | Reported as *Opuntia leptocaulis* DC*.*Succulent. | Cactaceae | D | S |
| Cymbopogon citratus (DC.) Stapf | 105.84 [698] | Leaf |  | Poaceae | M | H |
| Cymbopogon citratus (DC.) Stapf | 72.1 [699] | Leaf |  | Poaceae | M | H |
| Cymbopogon citratus (DC.) Stapf | 940 [700] | Leaf |  | Poaceae | M | H |
| *Cynara cardunculus* L. | 23.6 [701] | Leaf | Reported as *Cynara cardunculus* L. var. *altilis* DC. 30 mmol L^−1^ NaCl. Cultivar Bianco Avorio. | Asteraceae | D | H |
| *Cynara cardunculus* L. | 24.9 [702] | Leaf | Reported as *Cynara cardunculus* L. var. *altilis* DC. Reported as *Cynara cardunculus* L. var. *altilis* DC. 30 mmol·L^−1^ NaCl. Cultivar Gigante di Romagna. | Asteraceae | D | H |
| *Cynara cardunculus* L. | 26.4 [703] | Leaf | Reported as *Cynara cardunculus* L. var. *altilis* DC. Reported as *Cynara cardunculus* L. var. *altilis* DC. 30 mmol·L^−1^ NaCl. Cultivar Bianco Gigante Inerme. | Asteraceae | D | H |
| *Cynara cardunculus* L. | 36.1 [704] | Leaf | Reported as *Cynara cardunculus* L. var. *altilis* DC. 1 mmol·L^−1^ NaCl. Cultivar Bianco Avorio. | Asteraceae | D | H |
| *Cynara cardunculus* L. | 39.6 [705] | Leaf | Reported as *Cynara cardunculus* L. var. *altilis* DC. Reported as *Cynara cardunculus* L. var. *altilis* DC. 1 mmol·L^−1^ NaCl. Cultivar Gigante di Romagna. | Asteraceae | D | H |
| *Cynara cardunculus* L. | 40.2 [706] | Leaf | Reported as *Cynara cardunculus* L. var. *altilis* DC. Reported as *Cynara cardunculus* L. var. *altilis* DC. 1 mmol L^−1^ NaCl. Cultivar Bianco Gigante Inerme. | Asteraceae | D | H |
| *Cynara cardunculus* subsp. *flavescens* Wiklund | 21.6 [707] | Leaf | Reported as *Cynara cardunculus* L.subsp. *scolymus* (L.) Hegi. 30 mmol·L^−1^ NaCl. Cultivar Violetto di Provenza. | Asteraceae | D | H |
| *Cynara cardunculus* subsp. *flavescens* Wiklund | 22.0 [708] | Leaf | Reported as *Cynara cardunculus* L.subsp. *scolymus* (L.) Hegi. 30 mmol L^−1^ NaCl. Cultivar Violetto di Romagna. | Asteraceae | D | H |
| *Cynara cardunculus* subsp. *flavescens* Wiklund | 22.2 [709] | Leaf | Reported as *Cynara cardunculus* L.subsp. *scolymus* (L.) Hegi. 30 mmol·L^−1^ NaCl. Cultivar Romolo. | Asteraceae | D | H |
| *Cynara cardunculus* subsp. *flavescens* Wiklund | 31.6 [710] | Leaf | Reported as *Cynara cardunculus* L.subsp. *scolymus* (L.) Hegi. 1 mmol L^−1^ NaCl. Cultivar Romolo. | Asteraceae | D | H |
| *Cynara cardunculus* subsp. *flavescens* Wiklund | 35 [711] | Leaf | Reported as *Cynara cardunculus* L.subsp. *scolymus* (L.) Hegi. 1 mmol·L^−1^ NaCl. Cultivar Violetto di Romagna. | Asteraceae | D | H |
| *Cynara cardunculus* subsp. *flavescens* Wiklund | 37.8 [712] | Leaf | Reported as *Cynara cardunculus* L.subsp. *scolymus* (L.) Hegi. 1 mmol·L^−1^ NaCl. Cultivar Violetto di Provenza. | Asteraceae | D | H |
| *Cynara scolimus* L. | 123.7 [713] | Leaf | 705 mg/kg reported by Stef DS *et al.* 2010, but herbal parts not stated. | Asteraceae | D | H |
| *Cynodon dactylon* (L.) Pers | 558.8 [714] | Leaf | From a paper mill contaminated area. | Poaceae | M | H |
| *Cynodon dactylon* (L.) Pers | 1688.2 [715] | Root | From a paper mill contaminated area. | Poaceae | M | H |
| *Cynodon dactylon* (L.) Pers | 1576.5 [716] | Stem | From a paper mill contaminated area. | Poaceae | M | H |
| *Cynodon dactylon* (L.) Pers. | 1305.9 [717] | Aerial parts |  | Poaceae | M | H |
| *Cynodon dactylon* (L.) Pers. | 290 [718] | Aerial parts |  | Poaceae | M | H |
| Cynodon dactylon (L.) Pers. | 731 [719] | Aerial parts |  | Poaceae | M | H |
| *Cynometra louvellii* | 61.2 [720] | Leaf | Species not included in The Plant List. Possibly erroneous rendition of *Cynometra lyallii* Baker. | Fabaceae | D | T |
| *Cyperus compressus* L. | 1142.4 [721] | Aerial parts |  | Cyperaceae | M | H |
| *Cyperus compressus* L. | 2122.5 [722] | Aerial parts |  | Cyperaceae | M | H |
| *Cyperus involucratus* Rottb. | 144.2 | Leaf | From copper- and cobalt-rich Tailings of the Zambian Copperbelt. 257,600.0 mg/kg iron in associated reddish-brown tailings deposited in the Copperbelt impoundments. | Cyperaceae | M | H |
| *Cyperus involucratus* Rottb. | 253.1 [724] | Leaf | 15,967.2 mg/kg iron in associated grey-green tailings deposited in the Copperbelt impoundments. | Cyperaceae | M | H |
| *Cyperus* *haspan* L. | 18 [725] | Leaf | Reported as “leaves (or shoots)”. Peat soil (pH 3.6). | Cyperaceae | M | H |
| *Cyperus* *haspan* L. | 241 [726] | Leaf | Reported as “leaves (or shoots)”. Acid sulfate soil  (pH 3.4–3.6). | Cyperaceae | M | H |
| *Cytisus oromediterraneus* (G. López & C.E. Jarvis) Rivas Mart. & al | 226.6 [727] | Shoot | From an area around an abandoned mine. | Fabaceae | D | S |
| *Cytisus oromediterraneus* (G. López & C.E. Jarvis) Rivas Mart. & al | 24.5 [728] | Shoot | From an area around an abandoned mine. | Fabaceae | D | S |
| *Cytisus scoparius* (L.) Link | 140.1 [729] | Shoot | From an area around an abandoned mine. | Fabaceae | D | S |
| *Cytisus scoparius* (L.) Link | 28.5 [730] | Shoot | From an area around an abandoned mine. | Fabaceae | D | S |
| Cytisus striatus subsp. Eriocarpus  (Boiss. & Reut.) Rivas Mart. | 260 [731] | Aerial parts | Taxon not included in The Plant List (only *Cytisus striatus* (Hill) Rothm. is included in the List). | Fabaceae | D | S |
| *Dacrydium cupressinum* Sol. ex G.Forst. | 37.4 [732] | False fruit |  | Podocarpaceae | G | T |
| *Dacrydium cupressinum* Sol. ex G.Forst. | 36.9 [733] | Green aril |  | Podocarpaceae | G | T |
| *Dacrydium cupressinum* Sol. ex G.Forst. | 34.3 [734] | Red aril |  | Podocarpaceae | G | T |
| *Dacrydium cupressinum* Sol. ex G.Forst. | 41.1 [735] | Seed |  | Podocarpaceae | G | T |
| *Dactylis glomerata* L. | 114.0 [736] | Leaf | From a high-rainfall area. | Poaceae | M | H |
| *Dactylis glomerata* L. | 38.6 [737] | Stem | From a high- rainfall area. | Poaceae | M | H |
| *Dactylis glomerata* L. | 104.0 [738] | Whole plant | From a high-rainfall area. | Poaceae | M | H |
| *Dactyloctenium aegyptium* (L.) Willd. | 814.4 [739] | Aerial parts | Reported as *Dactyloctenium aegyptiacum* Willd. | Poaceae | M | H |
| *Dalbergia commiphoroides* Baker f. | 300 [740] | Aerial parts | These are approximations, as the authors expressed the values in grams/kg (including the iron in the group of “macro elements”) instead of the usual mg/kg or ppm. | Fabaceae | D | T |
| *Dalea bicolor* Willd. | 356^[741]^ | Leaf |  | Fabaceae | D | H |
| Daphne gnidium L. | 228^[742]^ | Aerial parts |  | Thymeleaceae | D | S |
| *Dasylepis eggelingii* J.B.Gillett | 182.2^[743]^ | Fruit |  | Achariaceae | D | T |
| *Dasylepis eggelingii* J.B.Gillett | 70.5^[744]^ | Seed |  | Achariaceae | D | T |
| *Datura stramonium* L. | 1910^[745]^ | Leaf |  | Solanaceae | D | H |
| *Datura stramonium* L. | 30.65 [746] | Leaf | In a waste dump area. | Solanaceae | D | H |
| *Datura stramonium* L. | 35.51 [747] | Root | In a waste dump area. | Solanaceae | D | H |
| *Datura stramonium* L. | 17.35 [748] | Stem | In a waste dump area. | Solanaceae | D | H |
| *Daucus carota* L. | 120.0 [749] | Root |  | Apiaceae | D | H |
| *Daucus carota* L. | 77.9 [750] | Root | Described as “carrot” | Apiaceae | D | H |
| *Daucus carota* L. | 39.4 [751] | Shoot | From an area around an abandoned mine. | Apiaceae | D | H |
| *Daucus carota* L. | 39.7 [752] | Shoot | From an area around an abandoned mine. | Apiaceae | D | H |
| *Davidia involucrata* Baill. | 365 [753] | Leaf |  | Cornaceae | D | T |
| *Deschampsia* *cespitosa* (L.) P.Beauv. | 250 [754] | Aerial parts | Reported as *Deschampsia caespitosa* (L.) P.Beauv. | Poaceae | M | H |
| *Deschampsia* *flexuosa* (L.) Trin. | 180 [755] | Aerial parts | From raised-bog peats. | Poaceaeae | M | H |
| *Deschampsia* *flexuosa* (L.) Trin. | 670 [756] | Aerial parts |  | Poaceae | M | H |
| *Deschampsia* *flexuosa* (L.) Trin. | 70 [757] | Aerial parts |  | Poaceae | M | H |
| *Detarium microcarpum* Guill. & Perr. | 25.3 [758] | Fruit | Reported (erroneously) as *Dereium microcarpum* [759]. | Fabaceae | D | T |
| *Detarium microcarpum* Guill. & Perr. | 38.9 [760] | Seed | Reported (erroneously) as *Dererium microcarpum.* | Fabaceae | D | T |
| *Dialium unifoliolatum* Capuron | 40.4 [761] | Fruit |  | Fabaceae | D | T |
| *Dialium unifoliolatum* Capuron | 37.8 [762] | Leaf |  | Fabaceae | D | T |
| *Dialyceras parvifolium* Capuron | 79.4 [763] | Fruit | Reported as *Dyalyceras parvifolia.* | Sphaerosepalaceae | D | T |
| *Dianthus chinensis* L. | 183 [764] | Aerial parts | Reported as *Dianthus versicolor* Fisch. ex. Link. | Caryophyllaceae | D | H |
| *Dicranopteris flexuosa* (Schrad.) Underw. | 80.3 [765] | Aerial parts |  | Gleicheniaceae | P | H |
| *Dicranum majus* Turner | 130 [766] | Aerial parts | From its typical habitat. | Dicranaceae | B | H |
| *Dicranum majus* Turner | 1600 [767] | Aerial parts | From its typical habitat. | Dicranaceae | B | H |
| *Dicranum majus* Turner | 1660 [768] | Aerial parts | “From an atypical habitat”. | Dicranaceae | B | H |
| *Digera muricata* (L.) Mart. | 20.75 [769] | Leaf |  | Amaranthaceae | D | H |
| Digitalis purpurea L. | 570 [770] | Aerial parts |  | Plantaginaceae | D | H |
| *Digitalis purpurea* L. | 220 [771] | Leaf |  | Plantaginaceae | D | H |
| *Digitalis purpurea* L. | 680 [772] | Leaf |  | Plantaginaceae | D | H |
| *Digitalis thapsi* L. | 107.1 [773] | Shoot | From an area around an abandoned mine. | Plantaginaceae | D | H |
| *Digitalis thapsi* L. | 831.6 ^[774]^ | Shoot | From an area around an abandoned mine. | Plantaginaceae | D | H |
| *Dillenia pentagyna* Roxb*.* | 162.0 [775] | Flower |  | Dilleniaceae | D | T |
| *Diodella* *scandens* (Sw.) Bacigalupo & E.L.Cabral | 30.37 [776] | Leaf | From unpolluted soil. Reported as *Diodia scandens* Sw. | Rubiaceae | D | H |
| *Diodella* *scandens* (Sw.) Bacigalupo & E.L.Cabral | 45.96 [777] | Leaf | From crude oil polluted soil. Reported as *Diodia scandens* Sw. | Rubiaceae | D | H |
| *Dioscorea bulbifera* L. | 575 [778] | Leaf |  | Dioscoreaceae | M | H |
| *Dioscorea bulbifera* L. | 317 [779] | Tuber |  | Dioscoreaceae | M | H |
| *Dioscorea pentaphylla* L. | 1662 [780] | Leaf |  | Dioscoreaceae | M | H |
| *Diospyros abyssinica* (Hiern) F.White | 56.5 [781] | Fruit |  | Ebenaceae | D | T |
| *Diospyros abyssinica* (Hiern) F.White | 106.4 [782] | Leaf | Young leaf | Ebenaceae | D | T |
| *Diospyros abyssinica* (Hiern) F.White | 64.0 [783] | Leaf |  | Ebenaceae | D | T |
| *Diospyros kaki* L.f. | 8.0 [784] | Fruit | Reported as *Diospyros kaki* Thunb. | Ebenaceae | D | T |
| *Diplazium esculentum* (Retz.) Sw. | 247.1 [785] | Leaf |  | Athyriaceae | P | H |
| *Diplazium esculentum* (Retz.) Sw. | 257 [786] | Leaf |  | Athyriaceae | P | H |
| Diplazium esculentum (Retz). SW. | 44.6 [787] | Leaf |  | Athyriaceae | P | H |
| *Diplazium esculentum* (Retz.) Sw. | 5600 [788] | Fronds | Reported as *Diplazium esculentum* (Retz) | Athyriaceae | P | H |
| *Diplazium esculentum* (Retz.) Sw. | 135.0 [789] | Leaf | Young, curled fronds. | Anthyriaceae | P | H |
| *Diplazium esculentum* (Retz.) Sw. | 47.3 [790] | Leaf | Mature fronds. | Anthyriaceae | P | H |
| *Diplazium esculentum* (Retz.) Sw. | 480 [791] | Leaf | Vegetative stage | Athyriaceae | P | H |
| *Diplazium esculentum* (Retz.) Sw. | 54.0 [792] | Leaf | Young, curled fronds. | Anthyriaceae | P | H |
| *Diplazium esculentum* (Retz.) Sw. | 595.6 [793] | Leaf |  | Athyriaceae | P | H |
| *Diplazium esculentum* (Retz.) Sw. | 634 [794] | Leaf | Reproductive stage | Athyriaceae | P | H |
| *Diplazium esculentum* (Retz.) Sw. | 94.7 [795] | Leaf | Mature fronds. | Anthyriaceae | P | H |
| *Diplazium esculentum* (Retz.) Sw. | 567.6 [796] | Root |  | Athyriaceae | P | H |
| *Diplazium esculentum* (Retz.) Sw. | 501 [797] | Stem |  | Athyriaceae | P | H |
| *Diplazium sammatii* (Kuhn.)C. Chr. | 43 [798] | Leaf | Young | Anthyriaceae | P | H |
| *Diplazium sammatii* (Kuhn.)C. Chr. | 67 [799] | Leaf | Mature | Anthyriaceae | P | H |
| *Diploknema butyracea* (Roxb.) H.J.Lam | 1780 [800] | Fruit | Reported as *Diploknema butyracea* Roxb | Sapotaceae | D | T |
| *Diplotaxis erucoides* (L.) DC. | 256.4 [801] | Shoot | From an area around an abandoned mine. | Brassicaceae | D | H |
| *Diplotaxis erucoides* (L.) DC. | 540.1 [802] | Shoot | From an area around an abandoned mine. | Brassicaceae | D | H |
| *Disanthus cercidifolius* Maxim. | 385.3 [803] | Leaf |  | Hamamelidaceae | D | S |
| *Dissotis erecta* Dandy | 73.41 [804] | Leaf | From unpolluted soil. | Melastomataceae | D | S |
| *Dissotis erecta* Dandy | 95.84 [805] | Leaf | From polluted (crude-oil contaminated) soil. | Melastomataceae | D | S |
| *Dombeya kirkii* Mast | 151.0 [806] | Leaf | Reported as *Dombeya mukole* Sprague. Young leaf. | Malvaceae | D | T |
| *Dombeya kirkii* Mast. | 35.1 [807] | Bark | Reported as *Dombeya mukole* Sprague | Malvaceae | D | T |
| *Dombeya kirkii* Mast. | 116.5 [808] | Leaf | Reported as *Dombeya mukole* Sprague | Malvaceae | D | T |
| *Dombeya kirkii* Mast. | 203.0 [809] | Petiole | Reported as *Dombeya mukole* Sprague | Malvaceae | D | T |
| *Dracaena reﬂexa* Lam. | 37.6 [810] | Fruit |  | Asparagaceae | M | T |
| *Drymaria cordata* (L.) Willd. ex Schult. | 22.1 [811] | Leaf |  | Caryophyllaceae | D | H |
| *Dryopteris spinulosa* (O.F. Müll.) Watt | 210 [812] | Leaf |  | Dryopteridaceae | P | H |
| *Dryopteris affinis* Fraser—Jenk ssp. *borreri* (Newman) Fraser-Jenk. | 80 [813] | Aerial parts |  | Dryopteridaceae | P | H |
| *Dryopteris filix-mas* (L.) Schott | 151 [814] | Aerial parts |  | Dryopteridaceae | P | H |
| *Dryopteris filix-mas* (L.) Schott | 230 [815] | Leaf |  | Dryopteridaceae | P | H |
| *Dryopteris filix-mas* (L.) Schott | 390[816] | Leaf |  | Dryopteridaceae | P | H |
| *Dryopteris* *filix*-*mass* (L.) Schott | 104 [817] | Leaf | Mean = 80 for leaf. Pollution-free area. | Dryopteridaceae | P | H |
| *Dryopteris* *filix*-*mass* (L.) Schott | 58 [818] | Leaf | Mean = 80 for leaf. Pollution-free area. | Dryopteridaceae | P | H |
| *Dryopteris* *filix*-*mass* (L.) Schott | 27 [819] | Stem | Mean = 33 for stem. Pollution-free area. | Dryopteridaceae | P | H |
| *Dryopteris* *filix*-*mass* (L.) Schott | 50 [820] | Stem | Mean = 33 for stem. Pollution-free area. | Dryopteridaceae | P | H |
| *Dryopteris* *filix*-*mass* (L.) Schott | 106 [821] | Whole plant | Mean = 78 whole plant. Pollution-free area. | Dryopteridaceae | P | H |
| *Dryopteris* *filix*-*mass* (L.) Schott | 40 [822] | Whole plant | Mean = 78 whole plant. Pollution-free area. | Dryopteridaceae | P | H |
| *Dryopteris* *carthusiana* (Vill.) H.P. Fuchs | 260 [823] | Leaf | Reported as *Dryopteris spinulosa* (O.F. Müll.) Watt | Dryopteridaceae | P | H |
| *Dryopteris expansa (C. Presl)* Fraser-Jenk. & Jermy | 25.3 [824] | Root | Results expressed by authors on a moisture basis, converted to dry basis by us. | Dryopteridaceae | P | H |
| *Duosperma longicalyx* (Deflers) Vollesen | 100 [825] | Aerial parts | Reported as *Duosperma eremophilum* (Milne-Redh.) Brummitt. These are approximations, as the authors expressed the values in grams/kg (including the iron in the group of “macro elements”) instead of the usual mg/kg or ppm. | Acanthaceae | D | S |
| *Dypsis lastelliana* (Baill.) Beentje & J.Dransf. | 39.4 [826] | Fruit | It may be argued that palm trees (including *Arecaceae* species) are not strictly trees (not having secondary wood), but rather large grasses, although in another view “tree” is not a technical  term, merely designating a certain size. | Arecaceae | M | T* |
| *Dypsis nodifera* Mart. | 51.5 [827] | Fruit | See comment to *Dypsis lastelliana.* | Arecaceae | M | T* |
| *Dysphania botrys* (L.) Mosyakin & Clemants | 4145 [828] | Whole plant | Reported as *Chenopodium botrys* L. From a copper and iron mine area. | Amaranthaceae | D | H |
| *Echinacea purpurea* (L.) Moench | 460 [829] | Aerial parts |  | Asteraceae | D | H |
| *Echinodorus grandiflorus* (Cham. & Schltdl.) Micheli | 1801.6 [830] | Leaf | Aquatic | Alismataceae | M | H |
| *Echinodorus grandiflorus* (Cham. & Schltdl.) Micheli | 367.8 [831] | Leaf | Aquatic | Alismataceae | M | H |
| *Echinophora tenuifolia* L. | 370.52 [832] | Leaf |  | Apiaceae | D | H |
| *Echinops ritrodes* Bunge | 1455 [833] | Whole plant | From a copper and iron mine area. | Asteraceae | D | H |
| *Eclipta prostrata* (L.) L. | 16.5 [834] | Leaf | Reported as *Eclipta alba* L. From non-polluted areas. Pollution defined based on heavy metal levels, not based on extern polluting factors. | Asteraceae | D | H |
| *Eclipta prostrata* (L.) L. | 19.1 [835] | Leaf | Reported as *Eclipta alba* L. From less polluted areas. Pollution defined based on heavy metal levels, not based on extern polluting factors. | Asteraceae | D | H |
| *Eclipta prostrata* (L.) L. | 23.9 [836] | Leaf | Reported as *Eclipta alba* L. From polluted areas. Pollution defined based on heavy metal levels, not based on extern polluting factors. | Asteraceae | D | H |
| *Eclipta* *prostrata* (L.) L. | 24.86 [837] | Leaf |  | Asteraceae | D | H |
| *Eclipta prostrata* (L.) L. | 31.53 [838] | Leaf | From a paper mill contaminated area. | Asteraceae | D | H |
| *Eclipta prostrata* (L.) L. | 150.2 [839] | Root | From a paper mill contaminated area. | Asteraceae | D | H |
| *Eclipta prostrata* (L.) L. | 11.9 [840] | Roots | Reported as *Eclipta alba* L. From less polluted areas. Pollution defined based on heavy metal levels, not based on extern polluting factors. | Asteraceae | D | H |
| *Eclipta prostrata* (L.) L. | 24.7 [841] | Roots | Reported as *Eclipta alba* L. From polluted areas. Pollution defined based on heavy metal levels, not based on extern polluting factors. | Asteraceae | D | H |
| *Eclipta prostrata* (L.) L. | 9.56 [842] | Roots | Reported as *Eclipta alba* L. From non-polluted areas. Pollution defined based on heavy metal levels, not based on extern polluting factors. | Asteraceae | D | H |
| *Eclipta prostrata* (L.) L. | 15.6 [843] | Stem | Reported as *Eclipta alba* L. From less polluted areas. Pollution defined based on heavy metal levels, not based on extern polluting factors. | Asteraceae | D | H |
| *Eclipta prostrata* (L.) L. | 24.6 [844] | Stem | Reported as *Eclipta alba* L. From polluted areas. Pollution defined based on heavy metal levels, not based on extern polluting factors. | Asteraceae | D | H |
| *Eclipta prostrata* (L.) L. | 8.95 [845] | Stem | Reported as *Eclipta alba* L. From non-polluted areas. Pollution defined based on heavy metal levels, not based on extern polluting factors. | Asteraceae | D | H |
| *Eclipta prostrata* (L.) L. | 81.9 [846] | Stem | From a paper mill contaminated area. | Asteraceae | D | H |
| *Ehretia cymosa* Thonn. | 167.1 [847] | Fruit | From hills near the Beijing Steel Factory. | Boraginaceae | D | T |
| *Ehretia cymosa* Thonn. | 94.8 [848] | Leaf | Young leaf. | Boraginaceae | D | T |
| *Eichhornia crassipes* (Mart.) Solms | 2889.23 [849] | Leaf | Aquatic. From a paper mill contaminated area. | Pontederiaceae | M | H |
| *Eichhornia crassipes* (Mart.) Solms | 1606.97 [850] | Root | Aquatic. From a paper mill contaminated area. | Pontederiaceae | M | H |
| *Elaeagnus angustifolia* L. | 10.3 [851] | Fruit |  | Elaeagnaceae | D | T |
| *Elaeagnus angustifolia* L. | 0.28 [852] | Leaf |  | Elaeagnaceae | D | T |
| *Elaeagnus latifolia* L. | 1800 [853] | Fruit |  | Eleagnaceae | D | T |
| *Elaeagnus latifolia* L. | 172.0 [854] | Fruot |  | Elaeagnaceae | D | S |
| *Elaeagnus pyriformis* Hook. f. | 159.0 [855] | Fruit |  | Elaeagnaceae | D | S |
| *Elaeagnus rhamnoides* (L.) A.Nelson | 150 [856] | Fruit | Reported as *Hippophae rhamnoides* ssp. *turkestanica.* | Eleagnaceae | D | S |
| *Elaeagnus rhamnoides* (L.) A.Nelson | 40 [857] | Fruit | Reported as *Hippophae rhamnoides* ssp. *turkestanica.* | Eleagnaceae | D | S |
| *Elaeagnus* *rhamnoides* (L.) A.Nelson | 560 [858] | Fruit | 87 mg/kg reported by Stef DS *et al.* 2010, but organ not stated. Reported as *Hippophae rhamnoides* L. | Eleagnaceae | D | T |
| *Elaeocarpus* *sikkimensis* Mast. | 1510 [859] | Fruit | Reported as *Elaeocarpus sikkimensis* Roxb. | Elaeocarpaceae | D | T |
| *Elaphoglossum sporadolepis* (Kunze ex Kuhn) T. Moore | 638.2 [860] | Aerial parts |  | Dryopteridaceae | P | H |
| *Eleocharis dulcis* (Burm.f.)Trin. ex Hensch. | 3.5 [861] | Root | Aquatic. | Cyperaceae | M | H |
| *Eleocharis palustris* var. *vigens* L.H.Bailey | 534 [862] | Aerial parts | Reported as *Eleocharis smallii* Britton*.* Aquatic. | Cyperaceae | M | H |
| *Eleocharis* *palustris* (L.) Roem. & Schult. | 440 [863] | Leaf | Semiaquatic. | Cyperaceae | M | H |
| *Elettaria cardamomum* (L.) Maton | 441 [864] | Fruit |  | Zingiberaceae | M | H |
| *Eleusine coracana* (L.)Gaertn. | 12.6 [865] | Seed |  | Poaceae | M | H |
| *Eleusine indica* (L.) Gaertn. | 340.6 [866] | Leaf | From a paper mill contaminated area. | Poaceae | M | H |
| *Eleusine indica* (L.) Gaertn. | 502.4 [867] | Root | From a paper mill contaminated area. | Poaceae | M | H |
| *Eleusine indica* (L.) Gaertn. | 448.7 [868] | Stem | From a paper mill contaminated area. | Poaceae | M | H |
| *Elodea canadensis* Michx. | 10 [869] | Aerial parts | Aquatic. Reported as *Anacharis canadensis* (Michx.) Planch. | Hydrocharitaceae | M | H. |
| *Elodea* *canadensis* Michx. | 1320 [870] | Leaf | Acquatic. | Hydrocharitaceae | M | H |
| *Embelia schimperi* Vatke | 253 [871] | Fruit | Reported as *Embelia abyssinica* Baker*.* | Primulaceae | D | S |
| *Emex spinosa* (L.) Campd. | 130 [872] | Seed |  | Polygonaceae | D | H |
| *Enhydra fluctuans* Lour*.* | 490.4 [873] | Leaf | This species is not reported in The Plant List.  Aquatic | Asteraceae | D | H |
| *Ephedra major* Host | 37.8 [874] | Leaf | Reported as *Ephedra vulgaris*. | Ephedraceae | G | S |
| *Equisetum arvense* L. | 108 [875] | Aerial parts | 160 [876]–804 [877]—parts not stated | Equisetaceae | P | H |
| *Equisetum arvense* L. | 527.7 [878] | Aerial parts | 160 [879]–804 [880]—parts not stated | Equisetaceae | P | H |
| *Equisetum arvense* L. | 742 [881] | Aerial parts |  | Equisetaceae | P | H |
| *Equisetum arvense* L. | 87 [882] | Aerial parts | 160 [883]–804 [884]—parts not stated | Equisetaceae | P | H |
| Equisetum ramosissimum Desf. | 109 [885] | Aerial parts |  | Equisetaceae | P | H |
| *Equisetum ramosissimum* Desf. | 2565 [886] | Aerial parts |  | Equisetaceae | P | H |
| *Equisetum ramosissimum* Desf. | 53.4 [887] | Shoot | From an area around an abandoned mine. | Equisetaceae | P | H |
| *Equisetum ramosissimum* Desf. | 56.6 [888] | Shoot | From an area around an abandoned mine. | Equisetaceae | P | H |
| *Eragrostis atrovirens* (Desf.) Trin. ex Steud. | 548.19 [889] | Leaf | From a paper mill contaminated area. | Poaceae | M | H |
| *Eragrostis atrovirens* (Desf.) Trin. exSteud. | 5063.4 [890] | Root | From a paper mill contaminated area. | Poaceae | M | H |
| *Eragrostis unioloides* (Retz.) Nees ex Steud. | 970.3 [891] | Aerial parts | Reported as *Eragrostis unioloides* (Retz.) Nees ex Steud. | Poaceae | M | H |
| Erica arborea L. | 223 [892] | Aerial parts |  | Ericaceae | D | S |
| *Erica arborea* L. | 223.2 [893] | Shoot | From an area around an abandoned mine. | Ericaceae | D | T |
| *Erica arborea* L. | 40.9 [894] | Shoot | From an area around an abandoned mine. | Ericaceae | D | T |
| Erica australis L. | 274 [895] | Aerial parts |  | Ericaceae | D | S |
| Erica lusitanica Rudolphi | 212 [896] | Aerial parts |  | Ericaceae | D | S |
| Erica mackaiana subsp. andevalensis (Cabezudo & Rivera) D.C.McClint. & E.C.Nelson | 365 [897] | Aerial parts | Reported as Erica andevalensis Cabezudo &J. Rivera. | Ericaceae | D | S |
| Erica scoparia L. | 202 [898] | Aerial parts |  | Ericaceae | D | S |
| Erica umbellata L. | 263 [899] | Aerial parts |  | Ericaceae | D | S |
| *Eriobotrya japonica* (Thunb.) Lindl. | 4.3 [900] | Fruit |  | Rosaceae | D | T |
| *Eriolobus indica Schn.* | 1100 [901] | Fruit | This species is not incluted in The Plant List. | Rosaceae | D | T |
| *Eriophorum* *angustifolium* Honck. | 160 [902] | Leaf | From raised-bog peats. | Cyperaceae | M | H |
| *Eriophorum* *vaginatum* L. | 100 [903] | Leaf | From raised-bog peats. | Cyperacea | M | H |
| *Erodium cicutarium* (L.) L'Hér. | 563.6 [904] | Leaf | Reported as *Erodium glutinosum* Dum. ssp. *dunense* Andrea (Rothm.). Reported in mmol/kg, converted by us in mg/kg. | Geraniaceae | D | H |
| *Erophila verna* (L.) DC. | 904.0 [905] | Leaf | Reported in mmol/kg, converted by us in mg/kg. | Brassicaceae | D | H |
| *Eruca vesicaria* (L.) Cav. | 119.5 [906] | Leaf | Reported as *Eruca sativa* Mill*.* Described as “Arugula” | Brassicaceae | D | H |
| *Eryngium* *foetidum* L. | 24.26 [907] | Leaf |  | Apiaceae | D | H |
| *Erythrina abyssinica* DC. | 134.4 [908] | Flower |  | Fabaceae | D | T |
| *Erythrina abyssinica* DC. | 152.4 [909] | Leaf | With petiole. Young leaf | Fabaceae | D | T |
| *Erythrina abyssinica* DC. | 193.0 [910] | Leaf | Without petiole. Young leaf. | Fabaceae | D | T |
| *Erythroxylum nitidulum* Baker | 32.4 [911] | Leaf |  | Erythroxylaceae | D | S |
| *Euadenia eminens* Hook.f. | 97.5 [912] | Fruit |  | Capparaceae | D | T |
| Eucalyptus globulus Labill. | 231 [913] | Aerial parts |  | Myrtaceae | D | T |
| *Eucommia ulmoides* Oliv. | 254.6 [914] | Leaf |  | Eucommiaceae | D | T |
| Eugenia cumini (L.) Druce | 192 [915] | Leaf | From an area degraded by anthropic activities. | Myrtaceae | D | T |
| *Eugenia uniflora* L. | 0 [916] | Fruit |  | Myrtaceae | D | T |
| *Euonymus alatus* (Thunb.) Siebold | 156 [917] | Leaf | Reported as *Euonymus alatus* f. *ciliatodentatus* (Franch. & Sav.) Hiyama | Celastraceae | D | S |
| *Euphorbia anacampseros* Boiss. | 5.3 [918] | Aerial parts | 1600 m altitude Reported as *Euphorbia anacampseros* Boiss. subsp. *anacampseros.* | Euphorbiaceae | D | H |
| *Euphorbia helioscopia* L. | 19.4 [919] | Whole plant |  | Euphorbiaceae | D | H |
| *Euphorbia macroclada* Boiss. | 1445 [920] | Whole plant | From a copper and iron mine area. | Euphorbiaceae | D | H |
| *Euphorbia rigida* M. Bieb. | 8.8 [921] | Aerial parts | 1600 m altitude | Euphorbiaceae | D | S |
| *Euphorbia tirucalli* L. | 2100 [922] | Aerial parts | These are approximations, as the authors expressed the values in grams/kg (including the iron in the group of “macro elements”) instead of the usual mg/kg or ppm. Succulent. | Euphorbiaceae | D | S |
| *Euphrasia rostkoviana* Hayne | 268.7 [923] | Aerial parts |  | Orobanchaceae | D | H |
| *Eurya acuminata* DC. | 60 [924] | Leaf |  | Pentaphylacaceae | D | T |
| *Euryale ferox* Salisb. | 1.5 [925] | Seed | Aquatic. | Nymphaeaceae | Mag | H |
| *Eurycorymbus cavaleriei* (H.Lév.) Rehder & Hand.-Mazz. | 321.4 [926] | Leaf |  | Sapindaceae | D | T |
| *Fagaropsis angolensis* (Engl.) H.M.Gardner | 190.8 [927] | Petiole |  | Rutaceae | D | T |
| *Fagus sylvatica* L. | 180 [928] | Leaf |  | Fagaceae | D | T |
| *Fagus crenata* Blume | 27 [929] | Bark |  | Fagaceae | D | T |
| *Fagus crenata* Blume | 51 [930] | Bark |  | Fagaceae | D | T |
| *Fagus crenata* Blume | 147 [931] | Leaf |  | Fagaceae | D | T |
| *Fagus crenata* Blume | 0 [932] | Wood | Under LOQ. | Fagaceae | D | T |
| *Fagus grandifolia* Ehrh. | 3.1 [933] | Wood |  | Fagaceae | D | T |
| *Fagus grandifolia* Ehrh. | 4.2 [934] | Wood |  | Fagaceae | D | T |
| *Fagus japonica* Maxim. | 111 [935] | Leaf |  | Fagaceae | D | T |
| *Fenerivia ghesquiereana* (Cavaco & Keraudren) R.M.K. Saunders | 103.6 [936] | Fruit | Reported as *Polyalthia ghesquiereana* Cavaco & Keraudren. | Annonaceae | Mag | T |
| *Festuca arundinacea* Schreb. | 180.0 [937] | Leaf | Reported as *Schedonorus phoenix* (Scop.) Holub. From a high-rainfall area. | Poaceae | M | H |
| *Festuca arundinacea* Schreb. | 34.8 [938] | Stem | Reported as *Schedonorus phoenix* (Scop.) Holub. From a high-rainfall area. | Poaceae | M | H |
| *Festuca arundinacea* Schreb. | 172.0 [939] | Whole plant | Reported as *Schedonorus phoenix* (Scop.) Holub. From a high-rainfall area. | Poaceae | M | H |
| *Ficus ampana* C.C.Berg | 152.7 [940] | Fruit |  | Moraceae | D | T |
| *Ficus asperifolia* Miq. | 203.5 [941] | Fruit | Reported as *Ficus urceolaris* Welw. ex Hiern | Moraceae | D | T |
| *Ficus asperifolia* Miq. | 121.2 [942] | Leaf | Young leaf. | Moraceae | D | T |
| *Ficus benghalensis* L. | 232 [943] | Fruit |  | Moraceae | D | T |
| *Ficus brachylepis* Welw. ex Hiern | 67.6 [944] | Fruit |  | Moraceae | D | T |
| Ficus carica L. | 201 [945] | Aerial parts |  | Moraceae | D | T |
| *Ficus carica* L. | 100.9 [946] | Fruit |  | Moraceae | D | T |
| *Ficus carica* L. | 2.7 [947] | Fruit |  | Moraceae | D | T |
| *Ficus carica* L. | 41.9 [948] | Fruit |  | Moraceae | D | T |
| *Ficus carica* L. | 56.9 [949] | Fruit |  | Moraceae | D | T |
| *Ficus conraui* Warb. | 87.7 [950] | Fruit | Reported as *Ficus stipulifera* Hutch*.* | Moraceae | D | T |
| *Ficus exasperata* Vahl. | 107.5 [951] | Fruit |  | Moraceae | D | T |
| *Ficus exasperata* Vahl. | 144.2 [952] | Leaf | Young leaf. | Moraceae | D | T |
| *Ficus exasperata* Vahl. | 59.8 [953] | Leaf |  | Moraceae | D | T |
| *Ficus lutea* Vahl. | 185.1 [954] | Fruit |  | Moraceae | D | T |
| *Ficus megapoda* Baker | 33.6 [955] | Fruit |  | Moraceae | D | T |
| *Ficus natalensis* Hochst. | 65.3 [956] | Fruit |  | Moraceae | D | T |
| *Ficus natalensis* Hochst. | 81.4 [957] | Fruit | Unripe fruit. | Moraceae | D | T |
| *Ficus natalensis* Hochst. | 122.1 [958] | Leaf | Young leaf. | Moraceae | D | T |
| *Ficus natalensis* Hochst. | 158.4 [959] | Leaf |  | Moraceae | D | T |
| *Ficus politoria* Lam. | 59.8 [960] | Fruit | Reported as *Ficus soroceoides* Baker*.* | Moraceae | D | T |
| *Ficus rubra* Vahl. | 182.2 [961] | Fruit |  | Moraceae | D | T |
| *Ficus saussureana* DC. | 71.1 [962] | Bud | Reported as *Ficus dawei* Hutch. Foliar buds. | Moraceae | D | T |
| *Ficus sur* Forssk. | 195.6 [963] | Fruit | Reported as *Ficus capensis* Thunb*.* | Moraceae | D | T |
| *Ficus sur* Forssk. | 281.8 [964] | Leaf | Reported as *Ficus capensis* Thunb. Young leaf. | Moraceae | D | T |
| *Ficus sycomorus* L. | 243.8 [965] | Fruit |  | Moraceae | D | T |
| *Ficus thonningii* Blume | 667.3 [966] | Leaf |  | Morceae | D | T |
| *Ficus tiliifolia* Baker | 147.2 [967] | Fruit |  | Moraceae | D | T |
| *Ficus vallis-caucae* Dugand | 175.4 [968] | Leaf | Young leaf. | Moraceae | D | T |
| *Filipendula* *ulmaria* (L.) Maxim. | 230 [969] | Leaf |  | Rosaceae | D | H |
| *Fimbristylis dichotoma* (L.) Vahl | 66.4 [970] | Leaf | Aquatic. From a paper mill contaminated area. | Cyperaceae | M | H |
| *Fimbristylis dichotoma* (L.) Vahl | 394.3 [971] | Root | Aquatic. From a paper mill contaminated area. | Cyperaceae | M | H |
| *Fimbristylis bisumbellata* (Forssk.) Bubani | 2.28 [972] | Leaf | From a paper mill contaminated area. | Cyperaceae | M | H |
| *Fimbristylis bisumbellata* (Forssk.) Bubani | 663.8 [973] | Root | From a paper mill contaminated area. | Cyperaceae | M | H |
| *Fimbristylis bisumbellata* (Forssk.) Bubani | 421 [974] | Stem | From a paper mill contaminated area. | Cyperaceae | M | H |
| *Fimbristylis* *quinquangularis* (Vahl) Kunth | 50 [975] | Leaf | Reported as “leaves (or shoots)”. Saline soil (pH 3.9). Reported as *Fimbristylis miliacea* (L.) Vahl. | Cyperaceae | M | H |
| *Flacourtia indica* (Burm.f.) Merr. | 734 [976] | Fruit |  | Salicaceae | D | T |
| *Flacourtia jangomas* (Lour.) Raeusch*.* | 43 [977] | Fruits |  | Salicaceae | D | T |
| *Flourensia cernua* DC | 167 [978] | Leaf |  | Asteraceae | D | S |
| *Flueggea leucopyrus* Willd. | 131.2 [979] | Aerial parts | Reported as *Securinega leucopyrus*. | Phyllanthaceae | D | S |
| Flueggea tinctoria (L.) G.L. Webster. | 284 [980] | Aerial parts |  | Phyllanthaceae | D | S |
| *Foeniculum* *vulgare* Mill. | 109 [981] | Fruit |  | Apiaceae | D | H |
| *Foeniculum vulgare* Mill. | 316 [982] | Fruit | 185 [983], 1034stated as “seed” | Apiaceae | D | H |
| *Foeniculum* *vulgare* Mill. | 43.8 [985] | Fruit |  | Apiaceae | D | H |
| *Foeniculum vulgare* Mill. | 72.04 [986] | Fruit | Reported as *Foeniculum vulgare* subsp. *piperitum* (C.Presl) Bég. | Apiaceae | D | H |
| *Foeniculum vulgare* Mill. | 328.68 [987] | Leaf | Reported as *Foeniculum vulgare* subsp. *piperitum* (C.Presl) Bég. 135 mg/kg reported by Stef DS *et al.* 2010, but organ not stated. | Apiaceae | D | H |
| *Fokienia hodginsii* (Dunn) A.Henry & H H.Thomas | 288.4 [989] | Leaf |  | Cupressaceae | G | T |
| *Frangula alnus* Mill. | 57.2 [990] | Shoot |  | Rhamnaceae | D | T |
| *Frangula alnus* Mill. | 69.2 [991] | Shoot |  | Rhamnaceae | D | T |
| *Fraxinus excelsior* L. | 120 [992] | Leaf |  | Oleaceae | D | T |
| *Fraxinus excelsior* L. | 440 [993] | Leaf |  | Oleaceae | D | T |
| *Fraxinus hubeiensis* S.Z.Qu, C.B.Shang & P.L.Su | 318.4 [994] | Leaf |  | Oleaceae | D | T |
| *Fraxinus mandshurica* Rupr. | 336.3 [995] | Leaf |  | Oleaceae | D | T |
| *Fraxinus sieboldiana* Blume | 89 [996] | Leaf |  | Oleaceae | D | T |
| *Fritillaria thunbergii* Miq. | 0.7 [997] | Bulb |  | Liliaceae | M | H |
| *Fritillaria thunbergii* Miq. | 10.1 [998] | Bulb |  | Liliaceae | M | H |
| *Fritillaria thunbergii* Miq. | 79.9 [999] | Bulb |  | Liliaceae | M | H |
| *Fritillaria usuriensis* Maxim. | 103.9 [1000] | Bulb |  | Liliaceae | M | H |
| Fumaria reuteri Boiss. | 125 [1001] | Aerial parts |  | Papaveraceae | D | H |
| *Funtumia africana* (Benth.) Stapf | 565.6 [1002] | Leaf | Reported as *Funtumia latifolia* (Stapf) Stapf. | Apocynaceae | D | T |
| *Funtumia africana* (Benth.) Stapf. | 66.4 [1003] | Fruit | Reported as *Funtumia latifolia* (Stapf) Stapf. | Apocynaceae | D | T |
| *Funtumia africana* (Benth.) Stapf. | 158.4 [1004] | Leaf | Reported as *Funtumia latifolia* (Stapf) Stapf. Young leaf. | Apocynaceae | D | T |
| *Galium verum* L. | 225 [1005] | Aerial parts |  | Rubiaceae | D | H |
| *Galium verum* L. | 68 [1006] | Aerial parts |  | Rubiaceae | D | H |
| *Garcinia megistophylla* P.W.Sweeney & Z.S.Rogers | 80.3 [1007] | Fruit | Reported as *Rheedia megaphylla* H.Perrier*.* | Clusiaceae | D | T |
| *Garcinia megistophylla* P.W.Sweeney & Z.S.Rogers | 21.5 [1008] | Leaf | Reported as *Rheedia megaphylla* H.Perrier*.* | Clusiaceae | D | T |
| *Gardenia aqualla* Stapf & Hutch. | 33.6 [1009] | Fruit |  | Rubiaceae | D | T |
| *Gardenia volkensii* K.Schum. | 40 [1010] | Aerial parts | These are approximations, as the authors expressed the values in grams/kg (including the iron in the group of “macro elements”) instead of the usual mg/kg or ppm. | Rubiaceae | D | T |
| *Gastrodia elata* Blume | 126.2 [1011] | Root |  | Orchidaceae | M | H |
| *Gaultheria shallon* Pursh. | 40 [1012] | Fruit |  | Ericaceae | D | S |
| *Genista cinerascens* Lange | 128.9 [1013] | Shoot | From an area around an abandoned mine. | Fabaceae | D | S |
| *Genista cinerascens* Lange | 39.6 [1014] | Shoot | From an area around an abandoned mine. | Fabaceae | D | S |
| Genista hirsuta Vahl | 221 [1015] | Aerial parts |  | Fabaceae | D | S |
| Genista polyanthos Willk. | 592 [1016] | Aerial parts | Reported as Genista polyanthos R. Roem. ex Willk. | Fabaceae | D | S |
| *Gentianopsis barbata* (Froel.) Ma | 156 [1017] | Aerial parts | Reported as *Gentianopsis barbata* L. | Gentianaceae | D | H |
| *Geranium* *robertianum* L. | 460 [1018] | Leaf |  | Geraniaceae | D | H |
| *Geranium pyrenaicum* Burm.f. | 4.7 [1019] | Aerial parts | 1600 m altitude Reported as *Geranium pyrenaicum* L. | Geraniaceae | D | H |
| *Ginkgo biloba* L. | 110 [1020] | Bark |  | Ginkgoaceae | G | T |
| *Ginkgo biloba* L. | 210 [1021] | Bark |  | Ginkgoaceae | G | T |
| *Ginkgo biloba* L. | 144.7 [1022] | Leaf | Male. | Ginkgoaceae | G | T |
| *Ginkgo biloba* L. | 177.5 | Leaf | Female | Ginkgoaceae | G | T |
| *Ginkgo biloba* L. | 2.8 [1024] | Leaf |  | Ginkgoaceae | G | T |
| *Ginkgo biloba* L. | 270 [1025] | Leaf |  | Ginkgoaceae | G | T |
| *Ginkgo biloba* L. | 338.4 [1026] | Leaf |  | Ginkgoaceae | G | T |
| *Ginkgo biloba* L. | 6.9 [1027] | Leaf |  | Ginkgoaceae | G | T |
| *Ginkgo biloba* L. | 2.6 [1028] | Seed |  | Ginkgoaceae | G | T |
| *Ginkgo biloba* L. | 0 [1029] | Wood | Under LOQ. | Ginkgoaceae | G | T |
| *Glebionis coronaria* (L.) Cass. ex Spach | 38.5 [1030] | Leaf | Reported as *Chrysanthemum coronarium* L. | Asteraceae | D | H |
| *Glebionis coronaria* (L.) Cass. ex Spach | 240 [1031] | Seed | Reported as *Chrysanthemum coronarium* L. var. *concolor* Batt. | Asteraceae | D | H |
| *Glebionis coronaria* (L.) Cass. ex Spach | 90 [1032] | Seed | Reported as *Chrysanthemum coronarium* L. var. *discolor.* | Asteraceae | D | H |
| *Gleditsia japonica* var. *velutina* L.Chu Li | 251.0 [1033] | Leaf | Reported as *Gleditsia vestita* B.G.Li. | Fabaceae | D | T |
| *Glinus oppositifolius* (L.)Aug.DC. | 782.4 [1034] | Leaf |  | Mollugniaceae | D | H |
| Gliricidia sepium (Jacq.) Walp. | 207.0 [1035] | Leaf |  | Fabaceae | D | T |
| *Glyceria fluitans* (L.) R.Br. | 448.9 [1036] | Shoot | From an area around an abandoned mine. | Poaceae | M | H |
| *Glyceria fluitans* (L.) R.Br. | 46.6 [1037] | Shoot | From an area around an abandoned mine. | Poaceae | M | H |
| *Glyceria* *fluitans* (L.) R.Br. | 310 [1038] | Leaf |  | Poaceaea | M | H |
| *Glycine max* (L.) Merr. | 292 | Aerial parts |  | Fabaceae | D | H |
| *Glycine max* (L.) Merr. | 110.3 [1039] | Seed |  | Fabaceae | D | H |
| *Glycine max* (L.) Merr. | 84.5 [1040] | Seed |  | Fabaceae | D | H |
| *Glycyrrhiza glabra* L. | 4823 [1041] | Root |  | Fabaceae | D | H |
| *Glycyrrhiza glabra* L. | 893.61 [1042] | Root |  | Fabaceae | D | H |
| *Glycyrrhiza glabra* L. | 3500 [1043] | Stem |  | Fabaceae | D | H |
| *Glycyrrhiza glabra* L. | 5892 [1044] | Stem |  | Fabaceae | D | H |
| *Glycyrrhiza glabra* L. | 6400 [1045] | Stem |  | Fabaceae | D | H |
| *Glycyrrhiza glabra* L. | 893.61 [1046] | Stem |  | Fabaceae | D | H |
| *Gnetum africanum* Welw. | 390 [1047] | Leaf |  | Gnetaceae | G | V |
| *Gnetum africanum* Welw. | 58 [1048] | Leaf | F. Epko *et al.* state 4.45% iron for the leaf. If really in percentage as written, that would mean 44.5 g/kg, which is unlikely. It is more likely that the real value is 4.45 mg/100 g, but it has not been written so. The situation is similar for another paper signed by this group of authors (Epko F. *et al.* 2012 [1050]), where 3.05% is reported. | Gnetaceae | G | V |
| *Gnetum africanum* Welw. | 78.8 [1051] | Leaf |  | Gnetaceae | G | V |
| *Gnetum africanum* Welw. | 1.5 [1052] | Seed |  | Gnetaceae | G | V |
| *Gossypium barbadense* L. | 38.8 [1053] | Leaf |  | Malvaceae | D | S |
| *Grewia cuneifolia* Juss. | 55.1 [1054] | Fruit |  | Malvaceae | D | T |
| *Grewia humblotii* Baill. | 225.4 [1055] | Fruit |  | Malvaceae | D | T |
| *Grewia mollis* Juss. | 104.4 [1056] | Bark |  | Malvaceae | D | T |
| *Grewia penicillata* Chiov. | 100 [1057] | Aerial parts | These are approximations, as the authors expressed the values in grams/kg (including the iron in the group of “macro elements”) instead of the usual mg/kg or ppm. | Malvaceae | D | S |
| *Grewia penicillata* Chiov. | 20 [1058] | Aerial parts | These are approximations, as the authors expressed the values in grams/kg (including the iron in the group of “macro elements”) instead of the usual mg/kg or ppm. | Malvaceae | D | S |
| *Grewia biloba* G.Don | 411.5 [1059] | Leaf | From hills near the Beijing Steel Factory. | Malvaceae | D | T |
| *Gymnanthemum coloratum* (Willd.) H.Rob. & B.Kahn | 570.8 [1060] | Leaf | Reported (erroneously) as *Veronia colorate* [*Vernonia colorata* (Willd.) Drake)] | Asteraceae | D | T |
| *Gymnema sylvestre* (Retz.) R.Br. ex Sm. | 16373 [1061] | Leaf |  | Apocynaceae | D | H |
| *Gymnema sylvestre* (Retz.) R.Br. ex Sm. | 599.7 [1062] | Leaf |  | Apocynaceae | D | H |
| *Gymnocarpium dryopteris* (L.) Newman | 350 [1063] | Leaf |  | Cystopteridaceae | P | H |
| *Gymnocarpium robertianum* (Hoffm.) Newman | 200 [1064] | Leaf |  | Cystopteridaceae | P | H |
| *Gymnosporia variabilis* (Hemsl.) Loes. | 316.1 [1065] | Leaf |  | Celastraceae | D | S |
| *Gypsophila struthium* Loefl. | 628 [1066] | Leaf |  | Caryophyllaceae | D | H |
| *Hagenia abyssinica* (Bruce ex Steud.) J.F.Gmel. | 126 [1067] | Flowers (female) |  | Rosaceae | D | T |
| *Hagenia abyssinica* (Bruce ex Steud.) J.F.Gmel. | 419 [1068] | Flowers (female) |  | Rosaceae | D | T |
| Halimium calycinum (L.) K. Koch | 636 [1069] | Aerial parts |  | Cistaceae | D | S |
| Halimium halimifolium (L.) Willk. | 83.6 [1070] | Aerial parts |  | Cistaceae | D | S |
| Halimium ocymoides (Lam.) Willk. | 377 [1071] | Aerial parts |  | Cistaceae | D | S |
| *Haloxylon persicum* Bunge | 230 [1072] | Aerial parts |  | Amaranthaceae | D | T |
| *Haloxylon salicornicum* (Moq.) Bunge ex Boiss. | 530 [1073] | Aerial parts | Reported as *Hammada salicornica* (Moq.) Iljin. | Amaranthaceae | D | S |
| *Heliamphora nutans* Benth. | 78.9 [1074] | Leaf | Carnivorous. Trap leaves. | Sarraceniaceae | D | H |
| *Helianthemum alypoides* Losa & Rivas Goday | 572 [1075] | Leaf |  | Cistaceae | D | H |
| *Helianthemum lavandulifolium* Mill. | 181 [1076] | Leaf |  | Cistaceae | D | H |
| *Helianthemum squamatum* (L.) Dum.Cours. | 547 [1077] | Leaf |  | Cistaceae | D | H |
| *Helianthus annuus* L. | 7.5 [1078] | Seed |  | Asteraceae | D | H |
| *Helichrysum armenium* DC. | 892.5 [1079] | Whole plant | Reported as *Heliochrysum armenium.* From a copper and iron mine area. | Asteraceae | D | H |
| Helichrysum stoechas (L.) Moench | 330 [1080] | Aerial parts |  | Asteraceae | D | H |
| *Hemerocallis minor* Mill. | 88 [1081] | Aerial parts |  | Xanthorrhoeaceae | M | H |
| *Heptacodium miconioides* Rehder | 369.9 [1082] | Leaf |  | Caprifoliaceae | D | T |
| *Heracleum sphondylium* subsp. *sibiricum* (L.) Simonk. | 131 [1083] | Aerial parts | Reported as *Heracleum sibiricum* L. | Ericaceae | D | S |
| *Heracleum sphondylium* subsp. *sibiricum* (L.) Simonk. | 71.9 [1084] | Aerial parts | Reported as *Heracleum sibiricum* L. | Ericaceae | D | S |
| *Heteropogon contortus* (L.) P.Beauv. ex Roem. &Schult. | 667.8 [1085] | Aerial parts |  | Poaceae | M | H |
| *Heteropogon contortus* (L.) P.Beauv. ex Roem. & Schult. | 862.5 [1086] | Aerial parts |  | Poaceae | M | H |
| *Hibiscus cannabinus* L. | 23.0 [1087] | Leaf |  | Malvaceae | D | H |
| *Hibiscus hamabo* Siebold & Zucc. | 255.8 [1088] | Leaf |  | Malvaceae | D | S |
| *Hibiscus rosa-sinensis* L. | 16.67 [1089] | Flower |  | Malvaceae | D | S |
| *Hibiscus sabdariffa* L. | 164.78 [1090] | Calyx |  | Malvaceae | D | S |
| *Hibiscus sabdariffa* L. | 306 [1091] | Calyx |  | Malvaceae | D | S |
| *Hibiscus sabdariffa* L. | 22.5 [1092] | Leaf |  | Malvaceae | D | Su |
| *Hildebrandtia africana* Vatke | 400 [1093] | Aerial parts | These are approximations, as the authors expressed the values in grams/kg (including the iron in the group of “macro elements”) instead of the usual mg/kg or ppm. | Convolvulaceae | D | S |
| *Hodgsonia heteroclita* (Roxb.) Hook.f. & Thomson | 4 [1094] | Seed |  | Cucurbitaceae | D | H |
| Holcus lanatus L. | 764 [1095] | Aerial parts |  | Poaceae | M | H |
| *Holcus mollis* L. | 130 [1096]- | Aerial parts |  | Poaceae | M | H |
| *Holcus mollis* L. | 290 [1097] | Aerial parts |  | Poaceae | M | H |
| *Hordeum murinum* L. | 133.3 [1098] | Aerial parts |  | Poaceae | M | H |
| *Hordeum murinum* L. | 166.7 [1099] | Aerial parts |  | Poaceae | M | H |
| Hordeum vulgare L. | 50 [1100] | Caryopse |  | Poaceae | M | H |
| *Hordeum vulgare* L. | 201 [1101] | Stem |  | Poaceae | M | H |
| *Hydrangea macrophylla* (Thunb.) Ser. | 173 [1102] | Leaf | Reported as *Hydrangea macrophylla* var. *acuminata*. |  |  |  |
| *Hydrangea paniculata* Siebold | 77 [1103] | Leaf |  | Hydrangeaceae | D | S |
| *Hydrocotyl* *sibthorpiodes* Lam. | 19.24 [1104] | Leaf |  | Araliaceae | M | H |
| *Hygrophila auriculata* (Schum.) Heine. | 481.5 [1105] | Leaf |  | Acanthaceae | D | H |
| *Hypericum* *perforatum* L. | 303 [1106] | Aerial parts |  | Hypericaceae | D | Su |
| *Hypericum perforatum* L. | 57 [1107] | Aerial parts |  | Hypericaceae | D | Su |
| *Hypericum perforatum* L. | 25.5 [1108] | Leaf |  | Hypericaceae | D | Su |
| *Hypericum perforatum* L. | 4.61 [1109] | Leaf | Razick, S.; *et al.*, 2008. 155 mg/kg reported by Stef DS *et al.* 2010, but herbal parts not stated. | Hypericaceae | D | Su |
| *Hypericum perforatum* L. | 119.2 [1110] | Shoot | From an area around an abandoned mine. | Hypericaceae | D | H |
| *Hypericum perforatum* L. | 92.8 [1111] | Shoot | From an area around an abandoned mine. | Hypericaceae | D | H |
| *Hypnum cupressiforme* Hedw. | 2566.8 [1112] | Leaf | Reported in mmol/kg, converted by us in mg/kg. | Hypnaceae | B | H |
| *Hypnum cupressiforme* Hedw. | 701 [1113] | Whole plant | Mean: 3360.9; Median: 3169.4 Reported as *Hypnum cupressiforme* Hedw. From an area in the vicinity of a steel plant. | Hypnaceae | B | H |
| *Hypnum cupressiforme* Hedw. | 7520 [1114] | Whole plant | Mean: 3360.9; Median: 3169.4 Reported as *Hypnum cupressiforme* Hedw. From an area in the vicinity of a steel plant. | Hypnaceae | B | H |
| *Hypnum molluscum* Hedw*.* | 2040 [1115] | Aerial parts |  | Hypnaceae | B | H |
| *Hypnum schreberi* Willd. ex Brid*.* | 860 [1116] | Aerial parts |  | Hypnaceae | B | H |
| *Ilex crenata* Thunb. | 97 [1117] | Leaf |  | Aquifoliaceae | D | T |
| *Ilex integra* Thunb. | 250.4 [1118] | Leaf |  | Aquifoliaceae | D | T |
| *Ilex macropoda* Miq. | 134 [1119] | Leaf |  | Aquifoliaceae | D | T |
| *Ilex mitis* (L.) Radlk. | 77.2 [1120] | Leaf |  | Aquifoliaceae | D | T |
| *Ilex mitis* (L.) Radlk. | 92.5 [1121] | Leaf | Young leaf. | Aquifoliaceae | D | T |
| *Ilex pedunculosa* Miq. | 73 [1122] | Leaf |  | Aquifoliaceae | D | S |
| Imperata cylindrica (L.) Raeusch. | 1808 [1123] | Aerial parts |  | Poaceae | M | H |
| *Indigofera astragalina* DC. | 209.5 [1124] | Leaf |  | Fabaceae | D | H |
| *Indigofera linnaei* Ali | 2370.1 [1125] | Aerial parts |  | Fabaceae | D | H |
| *Indigofera spinosa* Forssk. | 100 [1126] | Aerial parts | These are approximations, as the authors expressed the values in grams/kg (including the iron in the group of “macro elements”) instead of the usual mg/kg or ppm. | Fabaceae | D | S |
| *Indigofera spinosa* Forssk. | 400 [1127] | Aerial parts | These are approximations, as the authors expressed the values in grams/kg (including the iron in the group of “macro elements”) instead of the usual mg/kg or ppm. | Fabaceae | D | S |
| Inga edulis Mart. | 355 [1128] | Leaf | From an area degraded by anthropic activities. | Fabaceae | D | T |
| *Inula britannica* L. | 1140 ^[1129]^ | Aerial parts |  | Asteraceae | D | H |
| *Inula britannica* L. | 325 [1130] | Roots |  | Asteraceae | D | H |
| *Inula orientalis* Lam. | 6.1 [1131] | Aerial parts | 1600 m altitude | Asteraceae | D | H |
| *Ipomoea aquatica* Forssk*.* | 1439.6 [1132] | Leaf | Aquatic. From a paper mill contaminated area. | Convolvulaceae | D | H |
| *Ipomoea aquatica* Forssk*.* | 215.3 [1133] | Leaf | Aquatic. | Convolvulaceae | D | H |
| *Ipomoea aquatica* Forssk*.* | 22.4 [1134] | Leaf | Aquatic. | Convolvulaceae | D | H |
| *Ipomoea aquatica* Forssk*.* | 353 [1135] | Leaf |  | Convolvulaceae | D | H |
| *Ipomoea aquatica* Forssk. | 1375.66 [1136] | Root | Aquatic. From a paper mill contaminated area. | Convolvulaceae | D | H |
| *Ipomoea aquatica* Forssk. | 507.4 [1137] | Stem | Aquatic. From a paper mill contaminated area. | Convolvulaceae | D | H |
| Ipomoea batatas (L.) Lam. | 302.5 [1138] | Aerial parts | Destalked. | Convolvulaceae | D | H |
| *Ipomoea* *batatas* (L.) Lam. | 196 [1139] | Leaf | Reported as *Ipomoea batata* L. | Convolvulaceae | D | H |
| *Ipomoea batatas* (L.) Lam. | 35.0 [1140] | Tuber | Described as “Sweet potato tubers”. | Convolvulaceae | D | H |
| *Ipomoea batatas* L. | 328.4 [1141] | Leaf |  | Convolvulaceae | D | H |
| Iryanthera macrophylla Warb. | 340 [1142] | Leaf | Reported as Iryanthera macrophyla (Benth.) Warb. From an area degraded by anthropic activities. | Myristicaceae | Mig | T |
| *Ischaemum* *aristatum* L. | 43 [1143] | Leaf | Reported as “leaves (or shoots)”. Acid sulfate soil (pH 3.4–3.6). | Poaceae | M | H |
| *Ischaemum* *barbatum* Retz. | 40 [1144] | Leaf | Reported as “leaves (or shoots)”. Acid sulfate soil (pH 3.4–3.6). | Poaceae | M | H |
| *Isoetes sinensis* Palmer | 450.4 [1145] | Leaf |  | Isoëtaceae | P | H |
| *Isoetes* *lacustris* L. | 70 [1146] | Leaf | Acquatic | Isoëtaceae | P | H |
| *Itoa orientalis* Hemsl. | 348.6 [1147] | Leaf |  | Salicaceae | D | T |
| *Jacaranda mimosifolia* D.Don | 161.7 [1148] | Flower |  | Bignoniaceae | D | T |
| *Jacaranda mimosifolia* D.Don | 223 [1149] | Leaf | Reported as *Jacaranda mimosifolia* D.Don. From a mine contaminated area. | Bignoniaceae | D | T |
| *Jacaranda mimosifolia* D.Don | 93 [1150] | Leaf | Reported as *Jacaranda mimosifolia* D.Don. From a mine contaminated area. | Bignoniaceae | D | T |
| *Jasione montana* L. | 496 [1151] | Aerial parts |  | Campanulaceae | D | H |
| *Jatropha dioica* Sessé | 293 [1152] | Leaf |  | Euphorbiaceae | D | S |
| *Jatropha gossypifolia* L. | 266 [1153] | Leaf |  | Euphorbiaceae | D | H |
| Juncus acutiflorus subsp. rugosus (Steud.) Cout. | 168 [1154] | Aerial parts | Reported as Juncus rugosus Steud. | Juncaceae | M | H |
| Juncus acutus L. | 71 [1155] | Aerial parts |  | Juncaceae | M | H |
| Juncus effusus L. | 151 [1156] | Aerial parts | Reported as Juncus effusus var. subglomeratus DC. | Juncaceae | M | H |
| Juncus effusus L. | 70 [1157] | Leaf |  | Juncaceae | M | H |
| Juncus maritimus Lam. | 431 [1158] | Aerial parts |  | Juncaceae | M | H |
| *Juniperus deppeana* Steud. | 166 [1159] | Leaf | Reported as *Juniperus deppeana* Steud. From a mine contaminated area. | Cupressaceae | G | T |
| *Juniperus deppeana* Steud. | 93 [1160] | Leaf | Reported as *Juniperus deppeana* Steud. From a mine contaminated area. | Cupressaceae | G | T |
| *Juniperus drupacea* Labill. | 15.4 [1161] | False fruit | Reported as *Juniperus drupacea* Labill. | Cupressaceae | G | T |
| *Juniperus osteosperma* (Torr.) Little | 108.0 [1162] | Leaf | Mature leaves. | Cupressaceae | G | T |
| *Juniperus osteosperma* (Torr.) Little | 167.2 [1163] | Leaf | Juvenile leaves. | Cupressaceae | G | T |
| *Juniperus osteosperma* (Torr.) Little | 634.8 [1164] | Leaf | Seedlings. | Cupressaceae | G | T |
| *Juniperus oxycedrus* L. | 188.0 | Seed |  | Cupressaceae | G | T |
| *Justicia adhatoda* L. | 462.2 [1165] | Leaf | Reported as *Adhatoda vasica* Nees. | Acanthaceae | D | S |
| *Justicia glauca* Rottler | 391.1 [1166] | Aerial parts | Reported as *Justicia glauca* Rottler. | Acanthaceae | D | H |
| *Keteleeria davidiana* (C.E.Bertrand) Beissn. | 181.5 [1167] | Leaf |  | Pinaceae | G | T |
| *Keteleeria fortunei* (A.Murray bis) Carrière | 200.3 [1168] | Leaf |  | Pinaceae | G | T |
| *Kigelia africana* (Lam.) Benth. | 4.14 [1169] | Fruit |  | Bignoniaceae |  |  |
| *Kigelia africana* subsp. *moosa* (Sprague) Bidgood & Verdc*.* | 47.5 [1170] | Fruit | Reported as *Kigelia moosa* Sprague. | Bignoniaceae | D | T |
| *Koeleria macrantha* (Ledeb.) Schult. | 50.2 [1171] | Leaf | Reported in mmol/kg, converted by us in mg/kg. | Poaceae | M | H |
| *Kolkwitzia amabilis* Graebn. | 429.9 [1172] | Leaf |  | Caprifoliaceae | D | S |
| *Lablab purpureus* (L.) Sweet. | 4.4 [1173] | Seed |  | Fabaceae | D | H |
| *Lactuca orientalis* (Boiss.) Boiss. | 1317.5 [1174] | Whole plant | Reported as *Scariola orientalis* (Boiss.) Soják. From a copper and iron mine area. | Asteraceae | D | S |
| *Lactuca sativa* L. | 192.2 [1175] | Leaf | Stage 3 (75 days of growth) | Amaranthaceae | D | H |
| *Lactuca sativa* L. | 175.7 [1176] | Leaf | Stage 1 (25 days of growth) | Amaranthaceae | D | H |
| *Lactuca sativa* L. | 125.2 [1177] | Leaf | Stage 2(45 days of growth) | Amaranthaceae | D | H |
| *Lactuca sativa* L. | 465.2 [1178] | Leaf |  | Asteraceae | D | H |
| *Lactuca sativa* L. | 54.5 [1179] | Leaf |  | Asteraceae | D | H |
| *Lactuca sativa* L. | 9.86 [1180] | Leaf |  | Asteraceae | D | H |
| *Lactuca sativa* L. | 92 [1181] | Leaf |  | Asteraceae | D | H |
| *Lactuca sativa* L. | 224 [1182] | Root |  | Amaranthaceae | D | H |
| *Laetia corymbulosa* Spruce ex Benth. | 100.4 [1183] | Leaf | Young leaf. FeEDTA  (40 microM) as iron source. Aerobic conditions in hydroponic culture. | Salicaceae | D | T |
| *Laetia corymbulosa* Spruce ex Benth. | 217.6 [1184] | Leaf | Young leaf. FeSO_4_  (50 microM) as iron source. Aerobic conditions in hydroponic culture. | Salicaceae | D | T |
| *Laetia corymbulosa* Spruce ex Benth. | 256.7 [1185] | Leaf | Old leaf. FeSO_4_ (50 microM) as iron source. Aerobic conditions in hydroponic culture. | Salicaceae | D | T |
| *Laetia corymbulosa* Spruce ex Benth. | 267.8 [1186] | Leaf | Young leaf. FeSO_4_ (250 microM) as iron source. Hypoxic conditions in hydroponic culture. | Salicaceae | D | T |
| *Laetia corymbulosa* Spruce ex Benth. | 290.1 [1187] | Leaf | Old leaf. FeSO_4_ (250 microM) as iron source. Hypoxic conditions in hydroponic culture. | Salicaceae | D | T |
| *Laetia corymbulosa* Spruce ex Benth. | 401.8 [1188] | Leaf | Young leaf. FeSO_4_ (500 microM) as iron source. Hypoxic conditions in hydroponic culture. | Salicaceae | D | T |
| *Laetia corymbulosa* Spruce ex Benth. | 524.5 [1189] | Leaf | Old leaf. FeSO_4_ (500 microM) as iron source. Hypoxic conditions in hydroponic culture. | Salicaceae | D | T |
| *Laetia corymbulosa* Spruce ex Benth. | 94.9 [1190] | Leaf | Old leaf. FeEDTA (40 microM) as iron source. Aerobic conditions in hydroponic culture. | Salicaceae | D | T |
| Laetia corymbulosa Spruce ex Benth. | 157763.3 [1191] | Root | FeSO_4_ ­(500 microM) as iron source. Hypoxic conditions in hydroponic culture. | Salicaceae | D | T |
| Laetia corymbulosa Spruce ex Benth. | 1584.7 [1192] | Root | FeEDTA (40 microM) as iron source. Aerobic conditions in hydroponic culture. | Salicaceae | D | T |
| Laetia corymbulosa Spruce ex Benth. | 37637.1 [1193] | Root | FeSO_4_ (50 microM) as iron source. Aerobic conditions in hydroponic culture. | Salicaceae | D | T |
| Laetia corymbulosa Spruce ex Benth. | 91796.6 [1194] | Root | FeSO_4_ (250 microM) as iron source. Hypoxic conditions in hydroponic culture. | Salicaceae | D | T |
| *Laetia corymbulosa* Spruce ex Benth. | 1054.6 [1195] | Shoots | FeSO_4_ (250 microM) as iron source. Hypoxic conditions in hydroponic culture. | Salicaceae | D | T |
| Laetia corymbulosa Spruce ex Benth. | 122.8 [1196] | Shoots | FeEDTA (40 microM) as iron source. Aerobic conditions in hydroponic culture. | Salicaceae | D | T |
| *Laetia corymbulosa* Spruce ex Benth. | 1891.6 [1197] | Shoots | FeSO_4_ (500 microM) as iron source. Hypoxic conditions in hydroponic culture. | Salicaceae | D | T |
| Laetia corymbulosa Spruce ex Benth. | 742.1 [1198] | Shoots | FeSO_4_ (50 microM) as iron source. Aerobic conditions in hydroponic culture. | Salicaceae | D | T |
| *Lagenaria siceraria (*Molina*)* Standl*.* | 381.1 [1199] | Leaf |  | Cucurbitaceae | D | H |
| *Lagerstroemia guilinensis* S.K. Lee & L.F. Lau | 320.5 [1200] | Leaf |  | Lythraceae | D | S |
| Lamarckia aurea (L.) Moench | 159 [1201] | Aerial parts |  | Poaceae | M | H |
| *Lannea schimperi* (Hochst. ex A.Rich.) Engl. | 145.5 [1202] | Fruit | Reported (erroneously) as *Lannea schiniperi.* | Anacardiaceae | D | T |
| *Lannea triphylla* (Hochst. ex A. Rich.) Engl. | 6300 [1203] | Aerial parts | These are approximations, as the authors expressed the values in grams/kg (including the iron in the group of “macro elements”) instead of the usual mg/kg or ppm. | Anacardiaceae | D | T |
| *Lantana camara* L. | 318 [1204] | Leaf |  | Verbenaceae | D | Su |
| *Larix decidua* Mill. | 135 [1205] | Leaf |  | Pinaceae | G | T |
| *Larix decidua* Mill. | 30 [1206] | Wood | Stemwood. | Pinaceae | G | T |
| *Larix kaempferi* (Lamb.) Carrière | 430 [1207] | Bark |  | Pinaceae | G | T |
| *Larix kaempferi* (Lamb.) Carrière | 57 [1208] | Bark |  | Pinaceae | G | T |
| *Larix kaempferi* (Lamb.) Carrière | 3.1 [1209] | Wood |  | Pinaceae | G | T |
| *Larix kaempferi* (Lamb.) Carrière | 4.2 [1210] | Wood |  | Pinaceae | G | T |
| *Larix laricina* (Du Roi) K.Koch | 280 [1211] | Leaf | Mean: 130 | Pinaceae | G | T |
| *Larix laricina* (Du Roi) K.Koch | 40 [1212] | Leaf | Mean: 130 | Pinaceae | G | T |
| *Larrea tridentata* (Sessé & Moc. ex DC.) Coville | 374 [1213] | Leaf |  | Zygophyllaceae | D | S |
| *Lasia spinosa* (L.) Thwaites | 17.1 [1214] | Leaf | Aquatic | Araceae | M | H |
| *Lasianthera africana* (P. Beav.) | 9.0 [1215] | Leaf | Reportes as *Lesianthera africana* (P.Beav.) | Stemonuraceae | D | S |
| *Lathyrus clymenum* L. | 70 [1216] | Seed | Reported as *Lathyrus articulatus* L. | Fabaceae | D | H |
| *Lathyrus pratensis* L. | 177 [1217] | Aerial parts |  | Fabaceae | D | H |
| *Lathyrus pratensis* L. | 204 [1218] | Aerial parts |  | Fabaceae | D | H |
| *Lathyrus sativus* L. | 427.6 [1219] | Leaf |  | Fabaceae | D | H |
| *Laurus nobilis* L. | 126.38 [1220] | Leaf |  | Lauraceae | Mag | T |
| *Laurus nobilis* L. | 341.6 [1221] | Leaf |  | Lauraceae | Mag | T |
| *Laurus nobilis* L. | 194 [1222] | Root |  | Lauraceae | Mag | T |
| *Lavandula angustifolia* Mill. | 419.0 [1223] | Flower |  | Lamiaceae | D | H |
| *Lavandula angustifolia* Mill. | 61.2 [1224] | Flower |  | Lamiaceae | D | H |
| *Lavandula angustifolia* Mill. | 426.0 [1225] | Leaf |  | Lamiaceae | D | H |
| *Lavandula dentata* L. | 8447 [1226] | Aerial parts |  | Lamiaceae | D | Su |
| Lavandula pedunculata subsp. sampaiana (Rozeira) Franco | 474 [1227] | Aerial parts | Reported as Lavandula sampaioana (Rozeira) Rivas Mart., T.E. Díaz & Fern. Gonz. | Lamiaceae | D | Su |
| Lavandula stoechas subsp. luisieri (Rozeira) Rozeira | 454 [1228] | Aerial parts |  | Lamiaceae | D | Su |
| Lavandula viridis L ’ Her | 643 [1229] | Aerial parts |  | Lamiaceae | D | Su |
| *Lemna minor* L. | 248 [1230] | Aerial parts | Aquatic. | Araceae | M | H |
| *Lemna trisulca* L. | 70476.8 [1231] | Whole plant | Aquatic. | Araceae | M | H |
| Lens culinaris Medik. | 53.3 [1232] | Seed |  | Fabaceae | D | H |
| Leopoldia comosa (L.) Parl. | 635 [1233] | Aerial parts | Reported as Muscari comosum (L.) Mill. | Asparagaceae | M | H |
| *Lepidium sativum* L. | 161.58 [1234] | Leaf |  | Brassicaceae | D | H |
| *Lepidium sativum* L. | 11.4 [1235] | Whole plant |  | Brassicaceae | D | H |
| *Lepidium subulatum* L. | 293 [1236] | Leaf |  | Brassicaceae | D | S |
| *Leptonychia mildbraedii* Engl. | 90.9 [1237] | Leaf |  | Malvaceae | D | S |
| *Leptonychia mildbraedii* Engl. | 71.5 [1238] | Young leaf |  | Malvaceae | D | S |
| *Lespedeza floribunda* Bunge | 142.1 [1239] | Leaf | From Beijing Botanica Garden (control). | Fabaceae | D | Su |
| *Lespedeza floribunda* Bunge | 167.1 [1240] | Leaf | From hills near the Beijing Steel Factory. | Fabaceae | D | Su |
| *Lespedeza tomentosa* (Thunb.) Maxim. | 209.1 [1241] | Leaf | From Beijing Botanica Garden (control). | Fabaceae | D | Su |
| *Lespedeza tomentosa* (Thunb.) Maxim. | 263.4 [1242] | Leaf | From hills near the Beijing Steel Factory. | Fabaceae | D | Su |
| Leucaena leucocephala (Lam.) de Wit | 239.9 [1243] | Leaf |  | Fabaceae | D | T |
| *Leucas lavandulifolia* Sm. | 582.2 [1244] | Leaf | From a paper mill contaminated area. | Lamiaceae | D | H |
| *Leucas lavandulifolia* Sm. | 1083.7 [1245] | Root | From a paper mill contaminated area. | Lamiaceae | D | H |
| *Leucas lavandulifolia* Sm. | 457.3 [1246] | Stem | From a paper mill contaminated area. | Lamiaceae | D | H |
| [*Leucas zeylanica* (L.) W.T.Aiton](http://www.theplantlist.org/tpl1.1/record/kew-111936) | 22.66 [1247] | Leaf | Reported as *Leucas indica* (L.) Sm | Lamiaceae | D | H |
| *Leucobryum glaucum* (Hedw.) Ångstr. | 110 [1248] | Aerial parts |  | Dicranaceae | B | H |
| *Levisticum* *officinale* W.D.J.Koch | 403 [1249] | Leaf |  | Apiaceae | D | H |
| *Levisticum* *officinale* W.D.J.Koch | 78 [1250] | Leaf |  | Apiaceae | D | H |
| *Ligustrum expansum* Rehder | 264.8 [1251] | Leaf |  | Oleaceae | D | T |
| *Ligustrum ovalifolium* Hassk. | 138 [1252] | Leaf | From an urban area (Madrid). | Oleaceae | D | S |
| Limonium algarvense Erben | 91.3 [1253] | Aerial parts |  | Plumbaginaceae | D | H |
| *Lindera erythrocarpa* Makino | 89 [1254] | Leaf |  | Lauraceae | Mag | T |
| *Lindera praecox* (Siebold & Zucc.) Blume | 93 [1255] | Leaf | Reported as *Parabenzoin praecox* (Siebold & Zucc.) Nakai | Lauraceae | Mag | S |
| *Lindera umbellata* Thunb. | 110 [1256] | Leaf |  | Lauraceae | Mag | S |
| *Linum usitatissimum* L. | 170.5 [1257] | Seed | 66.9 mg/kg reported by S. Tokalioglu , but herbal parts not stated. | Linaceae | D | H |
| *Linum usitatissimum* L. | 24.6 [1259] | Seed |  | Linaceae | D | H |
| *Lippia alba* (Mill.) N.E.Br. ex Britton & P.Wilson | 27.4 [1260] | Leaf |  | Verbenaceae | D | S |
| *Litsea auriculata* S.S. Chien & W.C. Cheng | 359.9 [1261] | Leaf |  | Lauraceae | Mag | T |
| *Littorella* *uniflora* (L.) Asch. | 1050 [1262] | Leaf | Acquatic. | Plantaginaceae | D | H |
| *Littorella* *uniflora* (L.) Asch. | 1750 [1263] | Leaf | Acquatic. | Plantaginaceae | D | H |
| *Lolium perenne* L. | 213.0 [1264] | Leaf | From a high-rainfall area. | Poaceae | M | H |
| *Lolium perenne* L. | 70.2 [1265] | Stem | From a high-rainfall area. | Poaceae | M | H |
| *Lolium perenne* L. | 106.0 [1266] | Whole plant | From a high-rainfall area. | Poaceae | M | H |
| *Lolium rigidum* Gaudin | 200 [1267] | Seed |  | Poaceae | M | H |
| *Lolium temulentum* L. | 0.4 [1268] | Aerial parts | 1000 m altitude | Poaceae | M | H |
| *Lonicera periclymenum* subsp. *hispanica* (Boiss. & Reut.) Nyman | 243 [1269] | Aerial parts |  | Caprifoliaceae | D | S |
| *Lotus corniculatus* L. | 160 [1270] | Aerial parts |  | Fabaceae | D | H |
| *Lotus pedunculatus* Cav. | 137 [1271] | Aerial parts | Reported as *Lotus uliginosus* Schkuhr. | Fabaceae | D | H |
| *Lotus pedunculatus* Cav. | 189 [1272] | Aerial parts | Reported as *Lotus uliginosus* Schkuhr. | Fabaceae | D | H |
| *Ludwigia hyssopifolia* (G.Don) Exell | 491.8 [1273] | Leaf | From a paper mill contaminated area. | Onagraceae | D | H |
| *Ludwigia hyssopifolia* (G.Don) Exell | 3,622.5 [1274] | Root | From a paper mill contaminated area. | Onagraceae | D | H |
| *Ludwigia hyssopifolia* (G.Don) Exell | 587.2 [1275] | Stem | From a paper mill contaminated area. | Onagraceae | D | H |
| *Ludwigia perennis* L. | 16770.1 [1276] | Leaf | Aquatic. | Onagraceae | D | H |
| *Ludwigia perennis* L. | 101706.3 [1277] | Root | Aquatic. | Onagraceae | D | H |
| *Ludwigia perennis* L. | 19054.8 [1278] | Stem | Aquatic. | Onagraceae | D | H |
| *Lupinus micranthus* Guss. | 50 [1279] | Seed |  | Fabaceae | D | H |
| *Lychnodiscus cerospermus* Radlk | 225.2 [1280] | Fruit |  | Sapindaceae | D | T |
| *Lycium europaeum* L. | 200 [1281] | Aerial parts | These are approximations, as the authors expressed the values in grams/kg (including the iron in the group of “macro elements”) instead of the usual mg/kg or ppm. | Solanaceae | D | S |
| *Lycopodium clavatum* L. | 288.3 [1282] | Aerial parts |  | Lycopodiaceae | P | H |
| *Lycopodium* *clavatum* L. | 131 [1283] | Whole plant | Mean = 107. Pollution-free area. | Lycopodiaceae | P | H |
| *Lycopodium* *clavatum* L. | 84 [1284] | Whole plant | Mean = 107. Pollution-free area. | Lycopodiaceae | P | H |
| *Lycopodium clavatum* var. *nipponicum* | 76 [1285] | Leaf | This variety is not included in The Plant List | Lycopodiaceae | P | H |
| *Lyonia ovalifolia* (Wall.) Drude | 163 [1286] | Leaf |  | Ericaceae | D | T |
| *Maackia chekiangensis* S.S.Chien | 247.9 [1287] | Leaf |  | Fabaceae | D | S |
| *Machilus* *edulis* King ex Hook.f. | 2530 [1288] | Fruit | Reported as *Machilus edulis* King. | Lauraceae | Mag | T |
| *Macrosphyra longistyla* (DC.) Hiern | 102 [1289] | Leaf |  | Rubiaceae | D | S |
| *Maesa lanceolata* Forssk. | 489.0 [1290] | Fruit |  | Primulaceae | D | T |
| *Maesa lanceolata* Forssk. | 154.0 [1291] | Leaf | Young leaf. | Primulaceae | D | T |
| *Maesa lanceolata* Forssk. | 136.3 [1292] | Seed |  | Primulaceae | D | T |
| *Magnolia amoena* W.C.Cheng | 350.3 [1293] | Leaf |  | Magnoliaceae | Mag | T |
| *Magnolia cylindrica* E.H.Wilson | 403.9 [1294] | Leaf |  | Magnoliaceae | Mag | T |
| *Magnolia ernestii* Figlar | 166.6 [1295] | Leaf | Reported as *Michelia wilsonii* Finet & Gagnep. | Magnoliaceae | Mag | T |
| *Magnolia figo* var. *crassipes* (Y.W.Law) Figlar & Noot. | 277.5 [1296] | Leaf | Reported as *Michelia crassipes* Y.W.Law. | Magnoliaceae | Mag | T |
| *Magnolia grandis* (Hu & W.C.Cheng) V.S.Kumar | 265.4 [1297] | Leaf |  |  |  |  |
| *Magnolia insignis* Wall. | 229.1 [1298] | Leaf |  | Magnoliaceae | Mag | T |
| *Magnolia maudiae* (Dunn) Figlar | 169.8 [1299] | Leaf | Reported as *Michelia maudiae* Dunn. | Magnoliaceae | Mag | T |
| *Magnolia obovata* Thunb. | 11 [1300] | Bark |  | Magnoliaceae | Mag | T |
| *Magnolia obovata* Thunb. | 15 [1301] | Bark |  | Magnoliaceae | Mag | T |
| *Magnolia obovata* Thunb. | 111 [1302] | Leaf |  | Magnoliaceae | Mag | T |
| *Magnolia obovata* Thunb. | 0 [1303] | Wood | Under LOQ. | Magnoliaceae | Mag | T |
| *Magnolia odora* (Chun) Figlar & Noot. | 321.1 [1304] | Leaf | Reported as *Tsoongiodendron odorum* Chun. | Magnoliaceae | Mag | T |
| *Magnolia officinalis* Rehder & E.H.Wilson | 283.4 [1305] | Leaf |  | Magnoliaceae | Mag | T |
| *Magnolia officinalis var. biloba* Rehder & E.H.Wilson | 190.9 [1306] | Leaf | Reported as *Magnolia officinalis* subsp. *biloba* (Rehder & E.H.Wilson) W.C.Cheng & Y.W.Law | Magnoliaceae | Mag | T |
| *Magnolia patungensis* (Hu) Noot*.* | 161.2 [1307] | Leaf |  | Magnoliaceae | Mag | T |
| *Magnolia salicifolia* (Siebold & Zucc.) Maxim. | 95 [1308] | Leaf |  | Magnoliaceae | D | T |
| *Magnolia yunnanensis* (Hu) Noot. | 148.4 [1309] | Leaf | Reported as *Parakmeria yunnanensis* Hu. | Magnoliaceae | Mag | T |
| *Magnolia zenii* W.C.Cheng | 253.2 [1310] | Leaf |  | Magnoliaceae | Mag | T |
| *Maianthemum racemosum* (L.) Link. | 30 [1311] | Fruit | Reported as *Smilacina racemosa* (L.) Desf. | Asparagaceae | M | H |
| *Malus domestica* Borkh. | 55 [1312] | Fruit |  | Rosaceae | D | T |
| *Malus domestica* Borkh. | 85 [1313] | Leaf |  | Rosaceae | D | T |
| *Malus sylvestris* (L.) Mill. | 22.3 [1314] | Fruit | Described as “Wild apple” | Rosaceae | D | T |
| *Malva parviflora* L. | 60 [1315] | Seed |  | Malvaceae | D | H |
| *Malva* *sylvestris* L. | 159.0 [1316] | Flower |  | Malvaceae | D | H |
| *Mammea bongo* (R.Vig. & Humbert) Kosterm. | 43.7 [1317] | Fruit |  | Calophyllaceae | D | T |
| *Mangifera indica* L. | 22.2 [1318] | Fruit | Declared only as “mango” | Anacardiaceae | D | T |
| Manihot esculenta Crantz. | 577.9 [1319] | Aerial parts | Destalked. | Euphorbiaceae | D | S |
| *Manihot esculenta* Crantz. | 1.6 [1320] | Leaf | Reported on a fresh matter basis. Converted by us on dry matter basis. | Euphorbiaceae | D | H |
| *Manihot esculenta* Crantz. | 41.2 [1321] | Tuber |  | Euphorbiaceae | D | S |
| *Maranthes polyandra* (Benth.) Prance | 70 [1322] | Fruit |  | Chrysobalanaceae | D | T |
| *Markhamia lutea* (Benth.) K.Schum | 90.5 [1323] | Fruit | Reported as *Markhamia platycalyx* (Baker) Sprague | Bignoniaceae | D | T |
| *Markhamia lutea* (Benth.) K.Schum | 180.9 [1324] | Leaf | Leaflets and petioles (rachis) Reported as *Markhamia platycalyx* (Baker) Sprague | Bignoniaceae | D | T |
| *Markhamia lutea* (Benth.) K.Schum | 70.5 [1325] | Leaf | Leaflets only Reported as *Markhamia platycalyx* (Baker) Sprague | Bignoniaceae | D | T |
| *Markhamia lutea* (Benth.) K.Schum | 122.2 [1326] | Seed | Reported as *Markhamia platycalyx* (Baker) Sprague | Bignoniaceae | D | T |
| *Markhamia lutea* (Benth.) K.Schum | 109.1 [1327] | Young leaf | Reported as *Markhamia platycalyx* (Baker) Sprague | Bignoniaceae | D | T |
| *Markhamia lutea* (Benth.) K.Schum. | 214.5 [1328] | Flower | Reported as *Markhamia platycalyx* (Baker) Sprague | Bignoniaceae | D | T |
| *Markhamia lutea* (Benth.) K.Schum. | 311.0 [1329] | Petiole | Reported as *Markhamia platycalyx* (Baker) Sprague | Bignoniaceae | D | T |
| *Marrubium vulgare* L. | 68.8 [1330] | Aerial parts |  | Lamiaceae | D | Su |
| *Marsilea minuta* L. | 191 [1331] | Leaf |  | Marsileaceae | P | H |
| *Matricaria chamomilla* L. | 16.8 [1332] | Flower | Inflorescence. | Asteraceae | D | H |
| *Matricaria chamomilla* L. | 160.61 [1333] | Flower | Inflorescence. Inflorescence. | Asteraceae | D | H |
| *Matricaria chamomilla* L. | 227.5 [1334] | Flower |  | Asteraceae | D | H |
| *Matricaria chamomilla* L. | 15.7 [1335] | Flower. | Razick, S., *et al.*, 2008. 244 mg/kg reported by Stef DS *et al.* 2010, and 716 mg/kg by Tokalioglu S, 2012, but herbal parts not stated. Inflorescence. | Asteraceae | D | H |
| *Matricaria chamomilla* L. | 156.8 [1336] | Leaf | Reported as *Chamomilla recutita* (L.) Rauschert. | Asteraceae | D | H |
| *Matricaria chamomilla* L. | 201.7 [1337] | Leaf | Reported as *Chamomilla recutita* (L.) Rauschert. | Asteraceae | D | H |
| [*Matricaria* *chamomilla* L.](http://www.theplantlist.org/tpl1.1/record/gcc-103038) | 91.4 [1338] | Flower | Reported as *Matricaria recutita* L. Inflorescence. | Asteraceae | D | H |
| *Medicago sativa* L. | 195 [1339] | Aerial parts |  | Fabaceae | D | H |
| *Medicago sativa* L. | 31.21 [1340] | Leaf |  | Fabaceae | D | H |
| *Melaleuca* *cajuputi* Powell | 10 [1341] | Leaf | Reported as “leaves (or shoots)”. Acid sulfate soil (pH 3.4–3.6). | Myrtaceae | D | T |
| *Melaleuca* *cajuputi* Powell | 19 [1342] | Leaf | Reported as “leaves (or shoots)”. Peat soil (pH 3.6). | Myrtaceae | D | T |
| *Melaleuca* *cajuputi* Powell | 45 [1343] | Leaf | Reported as “leaves (or shoots)”. Sandy podzolic soil (pH 5.0) | Myrtaceae | D | T |
| *Melaleuca* *cajuputi* Powell | 51 [1344] | Leaf | Reported as “leaves (or shoots)”. Saline soil (pH 3.9) | Myrtaceae | D | T |
| *Melampyrum pratense* L. | 330 [1345] | Leaf |  | Orobanchaceae | D | H |
| *Melastoma malabathricum* L. | 10 [1346] | Leaf | Reported as “leaves (or shoots)”. Acid sulfate soil (pH 3.4–3.6). | Melastomataceae | D | S |
| *Melastoma malabathricum* L. | 64 [1347] | Leaf | Reported as “leaves (or shoots)”. Peat soil (pH 3.6). | Melastomataceae | D | S |
| *Melastoma* *malabathricum* l. | 95 [1348] | Leaf | Reported as „leaves (or shoots)”. Sandy podzolic soil (pH 5.0) | Melastomataceae | D | S |
| *Melastoma malabathricum* L. | 49 [1349] | Leaf | Reported as “leaves (or shoots)”. Saline soil (pH 6.2) | Melastomataceae | D | S |
| *Melica persica* Kunth | 1277.5 [1350] | Whole plant | Reported as *Melica jacquem[ontii].* From a copper and iron mine area. | Poaceae | M | H |
| *Melica* *uniflora* Retz. | 210 [1351] | Aerial parts |  | Poaceae | M | H |
| *Melica* *uniflora* Retz. | 220 [1352] | Aerial parts |  | Poaceae | M | H |
| *Melilotus indicus* (L.) All. | 100 [1353] | Seed | Reported as *Melilotus indica* (L.) All. | Fabaceae | D | H |
| *Melilotus suaveolens* Ledeb. | 105 [1354] | Aerial parts |  | Fabaceae | D | H |
| *Melilotus suaveolens* Ledeb. | 620 [1355] | Aerial parts |  | Fabaceae | D | H |
| *Melilotus sulcatus* Desf. | 80 [1356] | Seed |  | Fabaceae | D | H |
| *Melissa officinalis* L. | 126 [1357] | Leaf |  | Lamiaceae | D | H |
| *Melissa officinalis* L. | 1398 [1358] | Leaf |  | Lamiaceae | D | H |
| *Melissa officinalis* L. | 185 [1359] | Leaf |  | Lamiaceae | D | H |
| *Melissa officinalis* L. | 236.2 [1360] | Leaf |  | Lamiaceae | D | H |
| *Melissa officinalis* L. | 31.7 [1361] | Leaf |  | Lamiaceae | D | H |
| *Melissa officinalis* L. | 38.7 [1362] | Leaf |  | Lamiaceae | D | H |
| *Melissa officinalis* L. | 907.57 [1363] | Leaf |  | Lamiaceae | D | H |
| Melochia corchorifolia L. | 199.1 [1364] | Leaf |  | Malvaceae | D | H |
| *Memecylon bakerianum* Cogn. | 129.1 [1365] | Fruit |  | Melastomataceae | D | S |
| *Mentha arvensis* L. | 4144 [1366] | Leaf |  | Lamiaceae | D | H |
| *Mentha* *piperita* L. | 102 [1367] | Leaf |  | Lamiaceae | D | H |
| *Mentha* *piperita* L. | 1154 [1368] | Leaf |  | Lamiaceae | D | H |
| Mentha pulegium L. | 127 [1369] | Aerial parts |  | Lamiaceae | D | H |
| *Mentha pulegium* L. | 306.74 [1370] | Leaf |  | Lamiaceae | D | Su |
| *Mentha* *spicata* L. | 611.2 [1371] | Leaf | Reported as *Mentha viridis* L. | Lamiaceae | D | H |
| *Mentha spicata* L. | 91.8 | Leaf |  | Lamiaceae | D | H |
| *Mentha* x *piperita* L. | 117.5 | Leaf |  | Lamiaceae | D | H |
| *Mentha* x *piperita* L. | 230.36 | Leaf |  | Lamiaceae | D | H |
| *Mentha* x *piperita* L. | 29.2 [1375] | Leaf | 147 mg/kg reported by Stef DS *et al.* 2010, but herbal parts not stated. | Lamiaceae | D | H |
| *Mentha* x *piperita* L. | 37.9 | Leaf |  | Lamiaceae | D | H |
| *Mentha* x *piperita* L. | 402.43 [1377] | Leaf |  | Lamiaceae | D | H |
| *Mentha* x *piperita* L. | 734 [1378] | Leaf |  | Lamiaceae | D | H |
| *Menyanthes trifoliata* L. | 213 [1379] | Aerial parts | Aquatic. | Menyanthaceae | D | H |
| *Menyanthes* *trifoliata* L. | 160 [1380] | Leaf | Aquatic. | Menyanthaceae | D | H |
| *Mercurialis perennis* L. | 120 [1381] | Leaf |  | Euphorbiaceae | D | H |
| *Mercurialis perennis* L. | 820 [1382] | Leaf |  | Euphorbiaceae | D | H |
| *Merremia emarginata* (Burm. f.) Hallier f. | 1650.0 [1383] | Aerial parts |  | Convolvulaceae | D | H |
| *Mesembryanthemum nodiflorum* L. | 915 [1384] | Aerial parts |  | Aizoaceae | D | H |
| Micropyrum tenellum (L.) Link | 84 [1385] | Aerial parts |  | Poaceae | M | H |
| *Mikania micrantha* Kunth | 1363.24 [1386] | Leaf | From a paper mill contaminated area. | Asteraceae | D | V |
| *Mikania micrantha* Kunth | 1520 [1387] | Root | From a paper mill contaminated area. | Asteraceae | D | V |
| *Millettia dura* Dunn | 49.8 [1388] | Fruit |  | Fabaceae | D | T |
| *Millettia dura* Dunn | 144.0 [1389] | Leaf |  | Fabaceae | D | T |
| *Millettia dura* Dunn | 144.8 [1390] | Leaf | Young leaf. | Fabaceae | D | T |
| *Mimosa aculeaticarpa* Ortega | 218 [1391] | Leaf | Reported as *Mimosa biuncifera* Benth. | Fabaceae | D | S |
| *Mimosa pudica* L. | 5547 [1392] | Seed |  | Fabaceae | D | H |
| *Mimusops bagshawei* S.Moore | 255.1 [1393] | Fruit |  | Sapotaceae | D | T |
| *Mimusops bagshawei* S.Moore | 115.8 [1394] | Leaf | Young leaf | Sapotaceae | D | T |
| *Mimusops bagshawei* S.Moore | 162.0 [1395] | Leaf |  | Sapotaceae | D | T |
| *Mnium hornum* Hedw*.* | 120 [1396] | Aerial parts |  | Mniaceae | B | H |
| *Molinia* *caerulea* (L.) Moench | 110 [1397] | Leaf |  | Poaceaea | M | H |
| *Molinia* *caerulea* (L.) Moench | 120 [1398] | Leaf | From raised-bog peats. | Poaceae | M | H |
| *Monochoria vaginalis* (Burm.f.) C.Presl | 3000.0 [1399] | Leaf | Aquatic. | Pontederiaceae | M | H |
| *Monodora myristica* (Gaertner) Dunal | 508.7 [1400] | Seed |  | Annonaceae | Mag | T |
| *Monodora myristica* (Gaertn.) Dunal | 123.5 [1401] | Flower |  | Annonaceae | Mag | T |
| *Monodora myristica* (Gaertn.) Dunal | 75.9 [1402] | Fruit |  | Annonaceae | Mag | T |
| *Moringa oleifera* Lam. | 1050 [1403] | Leaf |  | Moringaceae | D | T |
| *Moringa oleifera* Lam. | 537 [1404] | Leaf |  | Moringaceae | D | T |
| *Morus alba* L. | 144.0 [1405] | Fruit |  | Moraceae | D | T |
| *Morus alba* L. | 15.2 [1406] | Fruit |  | Moraceae | D | T |
| Morus alba L. | 24.9 [1407] | Fruit | Reported as Morus multicaulis Perr. | Moraceae | D | T |
| Morus alba L. | 26.1 [1408] | Fruit | Reported as Morus multicaulis Perr. | Moraceae | D | T |
| Morus alba L. | 62.9 [1409] | Fruit |  | Moraceae | D | T |
| *Morus alba* L. | 19 [1410] | Leaf |  | Moraceae | D | T |
| *Morus alba* L. | 35.7 [1411] | Leaf |  | Moraceae | D | T |
| Morus atropurpurea Roxb. | 32.5 [1412] | Fruit |  | Moraceae | D | T |
| Morus atropurpurea Roxb. | 42.7 [1413] | Fruit |  | Moraceae | D | T |
| Morus cathayana Hemsl. | 65.0 [1414] | Fruit |  | Moraceae | D | T |
| *Murdannia edulis* (Stokes) Faden | 4782 [1415] | Seed | Reported as *Aneilema scapiflorum*. | Commelinaceae | M | H |
| *Murraya koenigii* (L.) Spreng. | 139 [1416] | Leaf |  | Rutaceae | D | T |
| *Murraya koenigii* (L.) Spreng. | 215 [1417] | Stem |  | Rutaceae | D | T |
| *Myosotis alpestris* F.W.Schmidt | 2.4 [1418] | Aerial parts | 1600 m altitude Reported as *Myosotis alpestris* F.W. Schmidt subsp. *alpestris.* | Boraginaceae | D | H |
| *Myosotis ramosissima* Rochel | 346.0 [1419] | Leaf | Reported in mmol/kg, converted by us in mg/kg. | Boraginaceae | D | H |
| Myrianthus arboreus P.Beauv. | 0 | Aerial parts | Destalked. Under the limit of detection. | Urticaceae | D | T |
| *Myrianthus holstii* Engl. | 165.8 [1421] | Fruit |  | Urticaceae | D | T |
| *Myrianthus holstii* Engl. | 203.0 [1422] | Petiole |  | Urticaceae | D | T |
| *Myrica gale* L. | 120 [1423] | Leaf |  | Myricaceae | D | S |
| *Myrica gale* L. | 120 [1424] | Leaf | From raised-bog peats. | Myricaceae | D | S |
| *Myricaria laxiflora* (Franch.) P.Y. Zhang & Y.J. Zhang | 247.9 [1425] | Leaf |  | Tamaricaceae | D | S |
| *Myriophyllum sibiricum* Kom. | 243 [1426] | Aerial parts | Reported as *Myriophyllum exalbescens* Fernald.Aquatic | Haloragaceae | D | H |
| Myristica fragrans Houtt. | 222 [1427] | Seed |  | Myristicaceae | Mag | T |
| Myrtus communis L. | 127 [1428] | Aerial parts |  | Myrtaceae | D | S |
| *Myrtus communis* L. | 44.83 | Leaf | 348 mg/kg reported by S. Tokalioglu , but herbal parts not stated. | Myrtaceae | D | T |
| *Nageia fleuryi* (Hickel) de Laub. | 99.7 [1431] | Leaf | Reported as *Podocarpus ﬂeuryi* Hickel. | Podocarpaceae | G | T |
| *Narcissus jonquilla* L. | 240 [1432] | Aerial parts |  | Amaryllidaceae | M | H |
| *Narthecium* *ossifragum* (L.) Huds. | 260 [1433] | Leaf |  | Nartheciaceae | M | H |
| *Nasturtium officinale* R. Br. | 25.37 [1434] | Leaf | Reported as *Nasturtium officinale* W.T. Aiton | Brassicaceae | D | H |
| *Nelumbium speciosum* Willd. | 3981.1 [1435] | Flower | Reported as *Nelymbium speciosum.* The Plant List includes *Nelumbium speciosum* both as an unresolved species and as a synonym for *Nelumbo nucifera* Gaertn. Aquatic. | Nelumbonaceae | D | H |
| *Nelumbium speciosum* Willd. | 18235.8 [1436] | Leaf | Reported as *Nelymbium speciosum.* The Plant List includes *Nelumbium speciosum* both as an unresolved species and as a synonym for *Nelumbo nucifera* Gaertn. Aquatic. | Nelumbonaceae | D | H |
| *Nelumbium speciosum* Willd. | 49207.4 [1437] | Root | Reported as *Nelymbium speciosum.* The Plant List includes *Nelumbium speciosum* both as an unresolved species and as a synonym for *Nelumbo nucifera* Gaertn. Aquatic. | Nelumbonaceae | D | H |
| *Nelumbium speciosum* Willd. | 25654.5 [1438] | Stem | Reported as *Nelymbium speciosum.* The Plant List includes *Nelumbium speciosum* both as an unresolved species and as a synonym for *Nelumbo nucifera* Gaertn. Aquatic. | Nelumbonaceae | D | H |
| *Neoboutonia macrocalyx* Pax | 105.5 [1439] | Leaf | Young leaf. | Euphorbiaceae | D | T |
| *Neoboutonia macrocalyx* Pax | 158.5 [1440] | Leaf |  | Euphorbiaceae | D | T |
| *Neoboutonia macrocalyx* Pax. | 212.6 [1441] | Fruit |  | Euphorbiaceae | D | T |
| *Neolitsea sericea* (Blume) Koidz. | 596.7 [1442] | Leaf |  | Lauraceae | Mag | T |
| *Neotina isoneura* (Radlk.) Capuron | 39.4 [1443] | Leaf |  | Sapindaceae | D | T |
| *Nephrolepis cordifolia* (L.)C. Presl | 1715 [1444] | Aerial parts |  | Nephrolepidaceae | P | H |
| *Nephrolepis cordifolia* (L.)C. Presl | 724.4 [1445] | Aerial parts |  | Nephrolepidaceae | P | H |
| *Nephrolepis furcans* | 26.2 [1446] | Leaf | This species is not included in The Plant List. Probably *Nephrolepis falcata* (Cav.) C. Chr., syn. *Nephrolepis biserrata* var. *furcans* hort. ex L.H. Bailey. | Nephrolepidaceae | P | H |
| *Neptunia oleracea* Lour. | 134 [1447] | Stem | Aquatic. | Fabaceae | D | H |
| *Nerium oleander* L. | 275 [1448] | Aerial parts |  | Apocynaceae | D | T |
| *Nerium oleander* L. | 240 [1449] | Leaf | From an urban area (Madrid). | Apocynaceae | D | S |
| *Nerium oleander* L. | 26.5 [1450] | Whole plant |  | Apocynaceae | D | S |
| *Nicotiana plumbaginifolia* Viv. | 691.2 [1451] | Leaf | From a paper mill contaminated area. | Solanaceae | D | H |
| *Nicotiana plumbaginifolia* Viv. | 1,047.5 [1452] | Root | From a paper mill contaminated area. | Solanaceae | D | H |
| *Nicotiana plumbaginifolia* Viv. | 815.1 [1453] | Stem | From a paper mill contaminated area. | Solanaceae | D | H |
| *Nigella sativa* L. | 3355 [1454] | Seed |  | Ranunculaceae | D | H |
| *Nigella sativa* L. | 71.21 [1455] | Seed | 90.5 mg/kg reported by S. Takalioglu, 2012, but herbal parts not stated. | Ranunculaceae | D | H |
| *Niphotrichum canescens* (Hedw.) Bednarek-Ochyra & Ochyra | 1562.4 [1457] | Leaf | Reported as *Rhacomitrium canescens* [*Racomitrium canescens* (Hedw.) Brid.]*.* Reported in mmol/kg, converted by us in mg/kg. | Grimmiaceae | B | H |
| *Notobasis syriaca* (L.) Cass. | 40 [1458] | Seed |  | Asteraceae | D | H |
| *Nuphar lutea* (L.) Sm. | 260 [1459] | Leaf | Acquatic. | Nymphaeaceae | Mag | H |
| *Nuphar lutea* (L.) Sm. | 3630 [1460] | Rhizome | Aquatic. | Nymphaeaceae | Mag | H |
| *Nuphar lutea* (L.) Sm. | 500 [1461] | Rhizome | Aquatic. | Nymphaeaceae | Mag | H |
| *Nuphar variegata* Durand | 583 [1462] | Aerial parts | Aquatic. | Nymphaeaceae | Mag | H |
| *Nymphaea nouchali* Burm.f. | 5139.4 [1463] | Flower | Reported as *Nymphaea stellata* Willd. Aquatic. | Nymphaeaceae | Mag | H |
| *Nymphaea nouchali* Burm.f. | 17209.0 [1464] | Leaf | Reported as *Nymphaea stellata* Willd. Aquatic. | Nymphaeaceae | Mag | H |
| *Nymphaea nouchali* Burm.f. | 37856.4 [1465] | Root | Reported as *Nymphaea stellata* Willd. Aquatic. | Nymphaeaceae | Mag | H |
| *Nymphaea nouchali* Burm.f. | 7783.7 [1466] | Stem | Reported as *Nymphaea stellata* Willd. Aquatic. | Nymphaeaceae | Mag | H |
| *Nymphaea odorata* subsp. *tuberosa* (Paine) Wiersema & Hellq. | 391 [1467] | Aerial parts | Reported as *Nymphaea tuberosa.* Aquatic. | Nymphaeaceae | Mag | H |
| *Nymphoides cristata* (Roxb.) Kuntze | 13740.3 [1468] | Leaf | Aquatic. | Menyanthaceae | D | H |
| *Nymphoides cristata* (Roxb.) Kuntze | 27570.9 [1469] | Root | Aquatic. | Menyanthaceae | D | H |
| *Nymphoides cristata* (Roxb.) Kuntze | 10355.9 [1470] | Stem | Aquatic. | Menyanthaceae | D | H |
| *Nypa fruiticans* Wurmb | 62 [1471] | Leaf | Reported as “leaves (or shoots)”. Saline soil (pH 6.2) | Arecaceae | M | T* |
| *Ochthocharis dicellandroides* (Gilg) C. Hansen & Wickens | 1000.0 [1472] | Leaf | Stated as 0.1%. Reported as *Ochtocharis dicellandroides* (Gilg.). | Melastomataceae | D | S |
| *Ocimum basilicum* L. | 141.2 [1473] | Aerial parts | 769–825 reported in another publication, but part not stated. | Lamiaceae | D | H |
| *Ocimum basilicum* L. | 945.3 [1475] | Leaf |  | Lamiaceae | D | H |
| *Ocimum basilicum* L. | 1237 [1476] | Seed |  | Lamiaceae | D | H |
| *Ocimum gratissimum* L. | 2521 [1477] | Leaf |  | Lamiaceae | D | Su |
| *Ocimum minimum* L. | 250.70 [1478] | Aerial parts |  | Lamiaceae | D | H |
| *Ocimum tenuiflorum* L. | 531.2 [1479] | Aerial parts |  | Lamiaceae | D | H |
| *Ocimum tenuiflorum* L. | 799.5 [1480] | Leaf | Reported as *Ocimum sanctum* L*.* | Lamiaceae | D | Su |
| *Oenanthe javanica* (Blume) DC. | 350 [1481] | Leaf | (Semi)-aquatic. | Apiaceae | D | H |
| *Oenanthe javanica* (Blume.) DC. | 32.0 [1482] | Leaf |  | Apiaceae | D | H |
| *Olax subscorpioides* Oliv. | 234 [1483] | Leaf |  | Olacaceae | D | T |
| [*Oldenlandia lactea* (Willd.) DC.](http://www.theplantlist.org/tpl1.1/record/kew-138503) | 24.73 [1484] | Leaf | Reported as *Oldenlandia corymbosa* Aiton | Rubiaceae | D | H |
| Olea europaea L. | 218 [1485] | Aerial parts | Reported as Olea europaea subsp. sylvestris (Mill.) Rouy ex Hegi | Oleaceae | D | T |
| *Olea welwitschii* (Knobl.) Gilg & G.Schellenb | 239.9 [1486] | Fruit |  | Oleaceae | D | T |
| *Olea welwitschii* (Knobl.)Gilg & G.Schellenb | 73.0 [1487] | Leaf | Young leaf. | Oleaceae | D | T |
| *Olea welwitschii* (Knobl.) Gilg & G.Schellenb | 82.2 [1488] | Leaf |  | Oleaceae | D | T |
| *Olea welwitschii* (Knobl.) Gilg & G.Schellenb | 167.3 [1489] | Petiole |  | Oleaceae | D | T |
| *Oncoba bukobensis* (Gilg) S. Hul & Breteler | 116.8 [1490] | Fruit | Reported as *Lindackeria bukobensis* Gilg | Flacourtiaceae | D | T |
| *Oncostemum botryoides* Baker | 247.3 [1491] | Fruit |  | Primulaceae | D | T |
| *Onoclea sensibilis* L. | 240 [1492] | Spores |  | Onocleaceae | P | H |
| *Ophioglossum thermale* Kom. | 265.1 [1493] | Leaf |  | Ophioglossaceae | P | H |
| *Opuntia leucotricha* DC. | 47 [1494] | Fruit | Succulent | Cactaceae | D | S |
| *Opuntia leucotricha* DC. | 51 [1495] | Fruit | Succulent | Cactaceae | D | S |
| *Opuntia leucotricha* DC. | 145 [1496] | Stem | Succulent | Cactaceae | D | S |
| *Orania longisquama* (Jum.) J.Dransf. & N.W.Uhl | 132.5 [1497] | Fruit | See comment to *Dypsis lastelliana.* | Arecaceae | M | T* |
| *Origanum majorana* L. | 231.4 [1498] | Leaf |  | Lamiaceae | D | Su |
| *Origanum majorana* L. | 137.8 [1499] | Root |  | Lamiaceae | D | Su |
| *Origanum vulgare* L. | 88.8 [1500] | Aerial parts |  | Lamiaceae | D | Su |
| *Origanum vulgare* L. | 44 [1501] | Leaf |  | Lamiaceae | D | Su |
| *Ormosia henryi* Prain | 318.3 [1502] | Leaf |  | Fabaceae | D | T |
| *Ormosia hosiei* Hemsl. & E.H.Wilson | 224.2 [1503] | Leaf |  | Fabaceae | D | T |
| *Oroxylum esculentum* Vent*.* | 180 | Fruit | This species is not incluted in The Plant List. Most likely it should have been *Oroxylum indicum* (L.) Kurz, the only species of this monotypic genus. | Bignoniaceae | D | T |
| *Oryza sativa* L. | 655.8 [1505] | Fruit | Described as “rice”. Caryopses (Fruit+seed). | Poaceae | M | H |
| *Oryza sativa* L. | 84.1 [1506] | Fruit | Described as „rice”. Caryopses (Fruit+seed). | Poaceae | M | H |
| *Osmanthus yunnanensis* (Franch.) P.S.Green | 224.7 [1507] | Leaf |  | Oleaceae | D | T |
| *Osmunda regalis* L. | 68 [1508] | Aerial parts |  | Osmundaceae | P | H |
| *Osmunda regalis* L. | 87 [1509] | Aerial parts |  | Osmundaceae | P | H |
| *Ostrya rehderiana* Chun | 391.6 [1510] | Leaf |  | Betulaceae | D | T |
| *Oxalis acetosella* L. | 190 [1511] | Leaf |  | Oxalidaceae | D | H |
| *Oxalis corniculata* L. | 18.74 [1512] | Leaf |  | Oxalidaceae | D | H |
| *Oxalis corniculata* L. | 365 [1513] | Leaf |  | Oxalidaceae | D | H |
| *Oxalis corniculata* L. | 57.14 [1514] | Leaf |  | Oxalidaceae | D | H |
| *Oxalis latifolia* Kunth | 775 [1515] | Leaf | Reported as *Oxalis latifolia* L. | Oxalidaceae | D | H |
| Oxytenanthera abyssinica (A.Rich.) Munro | 33.7 [1516] | Shoot | Reported as Oxytenanthera abyssinica (A.Rich.) Munro. | Poaceae | M | H |
| Oxytenanthera abyssinica (A.Rich.) Munro | 45.0 [1517] | Shoot | Reported as Oxytenanthera abyssinica (A.Rich.) Munro. | Poaceae | M | H |
| Oxytenanthera abyssinica (A.Rich.) Munro | 88.0 [1518] | Shoot | Reported as Oxytenanthera abyssinica (A.Rich.) Munro. | Poaceae | M | H |
| *Pancovia turbinata* Radlk. | 28.1 [1519] | Leaf | Young leaf. | Sapindaceae | D | T |
| *Pancovia turbinata* Radlk. | 34.6 [1520] | Leaf |  | Sapindaceae | D | T |
| Panicum repens L. | 589 [1521] | Aerial parts |  | Poaceae | M | H |
| *Panicum* *repens* L. | 49 [1522] | Leaf | Reported as “leaves (or shoots)”. Acid sulfate soil (pH 3.4–3.6). | Poaceae | M | H |
| *Panzerina lanata* (L.) Sojak | 1095 [1523] | Aerial parts |  | Lamiaceae | D | Su |
| *Papaver rhoeas* L. | 150 [1524] | Seed |  | Papaveraceae | D | H |
| *Papaver somniferum* L. | 10.09 | Seed |  | Papaveraceae | D | H |
| *Papaver* *somniferum* L. | 104.1 | Seed |  | Papavaraceae | D | H |
| *Papaver somniferum* L. | 149.6 | Seed |  | Papaveraceae | D | H |
| *Papaver somniferum* L. | 29 [1527] | Seed |  | Papaveraceae | D | H |
| *Papaver somniferum* L. | 44.8 [1528] | Seed |  | Papaveraceae | D | H |
| *Papaver* *somniferum* L. | 48.8 [1529] | Seed |  | Papavaraceae | D | H |
| *Parabenzoin trilobum* Nakai | 91 [1530] | Leaf |  | Lauraceae | Mag | S |
| *Paraceterach* *marantae* (L.) R.M. Tryon | 210 [1531] | Aerial parts | Reported as *Notholaena marantae* (L.) R. Br. | Pteridaceae | P | H |
| *Paraceterach* *marantae* (L.) R.M. Tryon | 245 [1532] | Aerial parts | Reported as *Notholaena marantae* (L.) R. Br. | Pteridaceae | P | H |
| *Parinari curatellifolia* Planch. ex Benth. | 103 [1533] | Fruit |  | Chrysobalanaceae | D | T |
| *Parinari curatellifolia* Planch. ex Benth. | 50.8 [1534] | Fruit | Reported (erroneously) as *Parinari curatellifolia* Planch. ex Benth. | Chrysobalanaceae | D | T |
| *Parinari excelsa* Sabine | 110.8 [1535] | Leaf | Young leaf. | Chrysobalanaceae | D | T |
| *Parinari excelsa* Sabine | 64.0 ^[1536]^ | Leaf |  | Chrysobalanaceae | D | T |
| *Parkia biglobosa* (Jacq.) G.Don | 91 ^[1537]^ | Flower |  | Fabaceae | D | T |
| *Parkia biglobosa* (Jacq.) G.Don | 11.0 ^[1538]^ | Fruit |  | Fabaceae | D | T |
| *Parkia biglobosa* (Jacq.) G.Don | 50.7 [1539] | Seed |  | Fabaceae | D | T |
| *Parkia biglobosa* (Jacq.) G.Don | 53.2 [1540] | Seed |  | Fabaceae | D | T |
| *Parkia biglobosa* (Jacq.) G.Don | 54.2 [1541] | Seed |  | Fabaceae | D | T |
| *Parkia speciosa* Hassk. | 22 [1542] | Seed |  | Fabaceae | D | T |
| *Parkia speciosa* Hassk. | 27 [1543] | Seed |  | Fabaceae | D | T |
| *Parthenium hysterophorus* L. | 528.3 [1544] | Aerial parts |  | Asteraceae | D | H |
| *Parthenium hysterophorus* L. | 66.1 [1545] | Leaf | From a paper mill contaminated area. | Asteraceae | D | H |
| *Parthenium hysterophorus* L. | 198.1 [1546] | Root | From a paper mill contaminated area. | Asteraceae | D | H |
| *Parthenium hysterophorus* L. | 87.7 [1547] | Stem | From a paper mill contaminated area. | Asteraceae | D | H |
| *Parthenium incanum* Kunth | 121 [1548] | Leaf |  | Asteraceae | D | S |
| *Paspalum logifolium* Roxb. | 11 ^[1549]^ | Leaf | Reported as “leaves (or shoots)”. Peat soil (pH 3.6). | Poaceae | M | H |
| *Paspalum longifolium* Roxb | 102 [1550] | Leaf | Reported as “leaves (or shoots)”. Acid sulfate soil (pH 3.4–3.6). | Poaceae | M | H |
| *Passiflora indica* Linn*.* | 350 | Fruit | This species is not included in The Plant List | Passifloraceae | D | V |
| *Paulownia tomentosa* Steud. | 21 [1552] | Bark |  | Paulowniaceae | D | T |
| *Paulownia tomentosa* Steud. | 84 [1553] | Bark |  | Paulowniaceae | D | T |
| *Paulownia tomentosa* Steud. | 4.0 [1554] | Wood |  | Paulowniaceae | D | T |
| *Pavonia odorata* Willd. | 262.6 [1555] | Aerial parts |  | Malvaceae | D | H |
| *Peganum harmala* L. | 4954 [1556] | Seed |  | Nitrariaceae | D | S |
| *Pennisetum glaucum* (L.) R.Br. | 234.7 [1557] | Fruit | Caryopses (Fruit+seed). Described as “bajra”. | Poaceae | M | H |
| *Peperomia pellucida* (L.) Kunth | 1069 [1558] | Leaf |  | Piperaceae | Mag | H |
| *Periploca sepium* Bunge | 268.4 [1559] | Leaf | From hills near the Beijing Steel Factory. | Apocynaceae | D | V |
| *Persicaria hydropiper* (L.) Delarbre | 1537 | Aerial parts | Reported as *Polygonum hydropiper* L. | Polygonaceae | D | H |
| *Persicaria chinensis* (L.) H. Gross | 18.21 [1561] | Leaf | Reported as *Polygonum chinense* L. | Polygonaceae | D | H |
| *Petasites japonicus* (Siebold.&Zucc.) Maxim. | 2.8 [1562] | Leaf |  | Asteraceae | D | H |
| *Petroselinum crispum* (Mill.) Fuss | 495.9 [1563] | Leaf | Described as “parsley”. | Apiaceae | D | H |
| Phagnalon saxatile (L.) Cass. | 380 [1564] | Aerial parts |  | Asteraceae | D | H |
| *Phalaris brachystachys* Link | 80 [1565] | Seed |  | Poaceae | M | H |
| *Phalaris* *arundinacea* L. | 340 [1566] | Leaf |  | Poaceaea | M | H |
| *Phaseolus vulgaris* L. | 46 [1567] | Fruit | Pod. Stage III (filling pod). | Fabaceae | D | H |
| *Phaseolus vulgaris* L. | 51 [1568] | Fruit | Pod. Stage II (beginning of seed filling). | Fabaceae | D | H |
| *Phaseolus vulgaris* L. | 68 [1569] | Fruit | Pod. Stage I (50% of flowering). | Fabaceae | D | H |
| *Phaseolus vulgaris* L. | 44 [1570] | Fruits | Pods. Cultivar Flor de Junio Marcela. | Fabaceae | D | H |
| *Phaseolus vulgaris* L. | 61 [1571] | Fruits | Pods. Cultivar Negro Jampa. | Fabaceae | D | H |
| *Phaseolus vulgaris* L. | 2669.9 [1572] | Leaf |  | Fabaceae | D | H |
| *Phaseolus vulgaris* L. | 439 [1573] | Leaf | Cultivar Negro Jampa. | Fabaceae | D | H |
| *Phaseolus vulgaris* L. | 510 [1574] | Leaf | Stage III (filling pod). | Fabaceae | D | H |
| *Phaseolus vulgaris* L. | 516 [1575] | Leaf | Stage II (beginning of seed filling). | Fabaceae | D | H |
| *Phaseolus vulgaris* L. | 573 [1576] | Leaf | Stage IV (physiological maturity). | Fabaceae | D | H |
| *Phaseolus vulgaris* L. | 643 [1577] | Leaf | Cultivar Pinto Villa. | Fabaceae | D | H |
| *Phaseolus vulgaris* L. | 69.70 [1578] | Leaf |  | Fabaceae | D | H |
| *Phaseolus vulgaris* L. | 89 [1579] | Leaf | Stage I (50% of flowering). | Fabaceae | D | H |
| *Phaseolus vulgaris* L. | 124 [1580] | Root | Stage I (50% of flowering). | Fabaceae | D | H |
| *Phaseolus vulgaris* L. | 154 [1581] | Root | Stage II (beginning of seed filling). | Fabaceae | D | H |
| *Phaseolus vulgaris* L. | 169 [1582] | Root | Stage III (filling pod). | Fabaceae | D | H |
| *Phaseolus vulgaris* L. | 216 [1583] | Root | Stage IV (physiological maturity). | Fabaceae | D | H |
| *Phaseolus vulgaris* L. | 5202.5 [1584] | Root | From a paper mill contaminated area. | Fabaceae | D | H |
| *Phaseolus vulgaris* L. | 155 [1585] | Roots | Cultivar Azufrado Higuera. | Fabaceae | D | H |
| *Phaseolus vulgaris* L. | 186 [1586] | Roots | Cultivar Pinto Villa. | Fabaceae | D | H |
| *Phaseolus vulgaris* L. | 51 [1587] | Seed | Cultivar Pinto Villa. | Fabaceae | D | H |
| *Phaseolus vulgaris* L. | 52 [1588] | Seed | Stage IV. | Fabaceae | D | H |
| *Phaseolus vulgaris* L. | 53 [1589] | Seed | Cultivar Flor de Junio Marcela. | Fabaceae | D | H |
| *Phaseolus vulgaris* L. | 56.5 [1590] | Seed |  | Fabaceae | D | H |
| *Phaseolus vulgaris* L. | 39 [1591] | Stem | Cultivar Azufrado Higuera. | Fabaceae | D | H |
| *Phaseolus vulgaris* L. | 44 [1592] | Stem | Stage I (50% of flowering). | Fabaceae | D | H |
| *Phaseolus vulgaris* L. | 46 [1593] | Stem | Stage III (filling pod). | Fabaceae | D | H |
| *Phaseolus vulgaris* L. | 49 [1594] | Stem | Stage II (beginning of seed filling). | Fabaceae | D | H |
| *Phaseolus vulgaris* L. | 49 [1595] | Stem | Stage IV (physiological maturity). | Fabaceae | D | H |
| *Phaseolus vulgaris* L. | 5063.4 [1596] | Stem | From a paper mill contaminated area. | Fabaceae | D | H |
| *Phaseolus vulgaris* L. | 58 [1597] | Stem | Cultivar Pinto Villa. | Fabaceae | D | H |
| *Phellodendron amurense* Rupr. | 395.5 [1598] | Leaf |  | Rutaceae | D | T |
| Phillyrea angustifolia L. | 153 [1599] | Aerial parts |  | Oleaceae | D | S |
| *Phlebodium pseudoaureum* (Cav.) Lellinger | 82.2 [1600] | Aerial parts | Reported as *Polypodium pseudoaureum* Cav. | Polypodiaceae | P | H |
| *Phleum arenarium* L. | 83.7 [1601] | Leaf | Reported in mmol/kg, converted by us in mg/kg. | Poaceae | M | H |
| *Phoebe bournei* (Hemsl.) Yen C. Yang | 176.1 [1602] | Leaf |  | Lauraceae | Mag | T |
| *Phoebe chekiangensis* P.T. Li | 285.3 [1603] | Leaf |  | Lauraceae | Mag | T |
| *Phoebe sheareri* (Hemsl.) Gamble | 265.2 [1604] | Leaf |  | Lauraceae | Mag | T |
| *Phoebe zhennan* S.K. Lee & F.N. Wei | 232.9 [1605] | Leaf |  | Lauraceae | Mag | T |
| *Photinia beauverdiana* C.K.Schneid. | 128 [1606] | Leaf | Reported as Reported as *Pourthiaea villosa* E.Pritz. | Rosaceae | D | T |
| Phragmites australis (Cav.) Trin. ex Steud. | 179 [1607] | Aerial parts |  | Poaceae | M | H |
| *Phragmites* *australis* (Cav.) Trin. ex Steud. | 230 [1608] | Leaf | Reported as *Phragmites communis* Trin. | Poaceaea | M | H |
| *Phyllanthus amarus* Schumach. & Thonn. | 43 [1609] | Leaf |  | Phyllanthaceae | D | H |
| *Phyllanthus muellerianus* (Kuntze) Exell | 514 [1610] | Leaf | Reported as *Phyllanthus muerillanus.* | Phyllanthaceae | D | T |
| *Phytolacca americana* L. | 20.2 [1611] | Shoots |  | Phytolaccaceae | D | H |
| *Picea abies* (L.) H.Karst. | 110 [1612] | Leaf | Leaf age: 1 year. Annual internode number from top of tree: 3 | Pinaceae | G | T |
| *Picea abies* (L.) H.Karst. | 12 [1613] | Leaf | Needles age class 1. Mean: 19.2 | Pinaceae | G | T |
| *Picea abies* (L.) H.Karst. | 120 [1614] | Leaf | Median = 30. | Pinaceae | G | T |
| *Picea abies* (L.) H.Karst. | 120 [1615] | Leaf | Leaf age: 1 year. Annual internode number from top of tree: 10 | Pinaceae | G | T |
| *Picea abies* (L.) H.Karst. | 130 [1616] | Leaf | Leaf age: 1 year. Annual internode number from top of tree: 5 | Pinaceae | G | T |
| *Picea abies* (L.) H.Karst. | 14 [1617] | Leaf | Needles age class 2. Mean: 21.8 | Pinaceae | G | T |
| *Picea abies* (L.) H.Karst. | 15 [1618] | Leaf | Needles age class 3. Mean: 23.8 | Pinaceae | G | T |
| *Picea abies* (L.) H.Karst. | 15 [1619] | Leaf | Needles age class 4. Mean: 26.5 | Pinaceae | G | T |
| *Picea abies* (L.) H.Karst. | 150 [1620] | Leaf | Leaf age: 3 years. Annual internode number from top of tree: 10 | Pinaceae | G | T |
| *Picea abies* (L.) H.Karst. | 150 [1621] | Leaf | Leaf age: 3 years. Annual internode number from top of tree: 5 | Pinaceae | G | T |
| *Picea abies* (L.) H.Karst. | 160 [1622] | Leaf | Leaf age: 1 year. Annual internode number from top of tree: 3 | Pinaceae | G | T |
| *Picea abies* (L.) H.Karst. | 160 [1623] | Leaf | Leaf age: 5+ years. Annual internode number from top of tree: 10 | Pinaceae | G | T |
| *Picea abies* (L.) H.Karst. | 17 [1624] | Leaf | Needles age class 5. Mean: 29.1 | Pinaceae | G | T |
| *Picea abies* (L.) H.Karst. | 20 [1625] | Leaf | Median = 30. | Pinaceae | G | T |
| *Picea abies* (L.) H.Karst. | 210 [1626] | Leaf | Leaf age: 1 year. Annual internode number from top of tree: 5 | Pinaceae | G | T |
| *Picea abies* (L.) H.Karst. | 220 [1627] | Leaf | Leaf age: 3 years. Annual internode number from top of tree: 10 | Pinaceae | G | T |
| *Picea abies* (L.) H.Karst. | 240 [1628] | Leaf | Leaf age: 1 year. Annual internode number from top of tree: 10 | Pinaceae | G | T |
| *Picea abies* (L.) H.Karst. | 240 [1629] | Leaf | Leaf age: 3 years. Annual internode number from top of tree: 5 | Pinaceae | G | T |
| *Picea abies* (L.) H.Karst. | 25 [1630] | Leaf | Needles age class 1. Mean: 19.2 | Pinaceae | G | T |
| *Picea abies* (L.) H.Karst. | 35 [1631] | Leaf | Needles age class 2. Mean: 21.8 | Pinaceae | G | T |
| *Picea abies* (L.) H.Karst. | 45 [1632] | Leaf | Needles age class 3. Mean: 23.8 | Pinaceae | G | T |
| *Picea abies* (L.) H.Karst. | 510 [1633] | Leaf | Leaf age: 5+ years. Annual internode number from top of tree: 10 | Pinaceae | G | T |
| *Picea abies* (L.) H.Karst. | 55 [1634] | Leaf | Needles age class 4. Mean: 26.5 | Pinaceae | G | T |
| *Picea abies* (L.) H.Karst. | 65 [1635] | Leaf | Needles age class 5. Mean: 29.1 | Pinaceae | G | T |
| *Picea jezoensis* (Siebold & Zucc.) Carrière | 16 [1636] | Bark |  | Pinaceae | G | T |
| *Picea jezoensis* (Siebold & Zucc.) Carrière | 3.6 [1637] | Bark |  | Pinaceae | G | T |
| *Picea jezoensis* subsp. *hondoensis* (Mayr) P.A.Schmidt | 26 [1638] | Bark | Reported as *Picea jezoensis* var *hondoensis*. | Pinaceae | G | T |
| *Picea jezoensis* subsp. *hondoensis* (Mayr) P.A.Schmidt | 59 [1639] | Bark | Reported as *Picea jezoensis* var *hondoensis*. | Pinaceae | G | T |
| *Picea jezoensis* subsp. *hondoensis* (Mayr) P.A.Schmidt | 0 [1640] | Wood | Reported as *Picea jezoensis* var *hondoensis*. Under LOQ. | Pinaceae | G | T |
| *Picea mariana* (Mill.) Britton, Sterns & Poggenb. | 0.4 [1641] | Leaf | In a polluted area (80 years of mining and smelting). | Pinaceae | G | T |
| *Picea mariana* (Mill.) Britton, Sterns & Poggenb. | 12.8 [1642] | Leaf | In a polluted area (80 years of mining and smelting). | Pinaceae | G | T |
| *Picea mariana* (Mill.) Britton, Sterns & Poggenb. | 59.7 [1643] | Leaf | In a polluted area (80 years of mining and smelting). | Pinaceae | G | T |
| *Picea mariana* (Mill.) Britton, Sterns & Poggenb. | 313.3 [1644] | Stem | Not the main stem, but branches. In a polluted area (80 years of mining and smelting). | Pinaceae | G | T |
| *Picea mariana* (Mill.) Britton, Sterns & Poggenb. | 38.0 [1645] | Stem | Not the main stem, but branches. In a polluted area (80 years of mining and smelting). | Pinaceae | G | T |
| *Picea mariana* (Mill.) Britton, Sterns & Poggenb. | 75.8 [1646] | Stem | Not the main stem, but branches. In a polluted area (80 years of mining and smelting). | Pinaceae | G | T |
| *Picea rubens* Sarg. | 12 [1647] | Leaf | Mean: 31.9 | Pinaceae | G | T |
| *Picea rubens* Sarg. | 67 [1648] | Leaf | Mean: 31.9 | Pinaceae | G | T |
| *Picea engelmannii* Parry ex Engelm. | 0 [1649] | Wood | Under LOQ. | Pinaceae | G | T |
| *Pimenta dioica* (L.) Merr. | 82.5 [1650] | Fruit |  | Myraceae | D | T |
| *Pimpinella anisum* L. | 4.0 [1651] | Aerial parts | 1000 m altitude | Apiaceae | D | H |
| *Pimpinella anisum* L. | 1799.5 [1652] | Fruit |  | Apiaceae | D | H |
| *Pimpinella* *anisum* L. | 193.9 [1653] | Fruit |  | Apiaceae | D | H |
| *Pimpinella anisum* L. | 374 [1654] | Leaf | Reported as *Anisum vulgare* Gaertn. | Apiaceae | D | H |
| *Pimpinella anisum* L. | 385 [1655] | Leaf | Reported as *Anisum vulgare* Gaertn. | Apiaceae | D | H |
| *Pinus armandii var. dabeshanensis* (W.C.Cheng & Y.W.Law) Silba | 144.6 [1656] | Leaf |  | Pinaceae | G | T |
| *Pinus banksiana* Lamb. | 107 [1657] | Leaf |  | Pinaceae | G | T |
| *Pinus banksiana* Lamb. | 342 [1658] | Leaf |  | Pinaceae | G | T |
| *Pinus densiflora* Siebold & Zucc. | 190 [1659] | Bark |  | Pinaceae | G | T |
| *Pinus densiflora* Siebold & Zucc. | 35 [1660] | Bark |  | Pinaceae | G | T |
| *Pinus densiflora* Siebold & Zucc. | 179 [1661] | Leaf |  | Pinaceae | G | T |
| *Pinus densiflora* Siebold & Zucc. | 0 [1662] | Wood | Under LOQ. | Pinaceae | G | T |
| *Pinus densiflora* Siebold & Zucc*.* x *Pinus tumbergii* Parl. | 137 [1663] | Leaf | Hybrid not reported in The Plant List | Pinaceae | G | T |
| *Pinus fenzeliana* Hand.-Mazz. | 204.7 [1664] | Leaf | Reported as *Pinus kwangtungensis* Chun ex Tsiang. | Pinaceae | G | T |
| *Pinus halepensis* Mill. | 159 [1665] | Bark | Water soluble iron: 8.0. | Pinaceae | G | T |
| *Pinus halepensis* Mill. | 180 [1666] | Bark | Water soluble iron: 7.8. | Pinaceae | G | T |
| *Pinus halepensis* Mill. | 766 [1667] | Bark | Water soluble iron: 31.6. | Pinaceae | G | T |
| *Pinus halepensis* Mill. | 134 [1668] | Leaf |  | Pinaceae | G | T |
| *Pinus halepensis* Mill. | 59.6 [1669] | Leaf |  | Pinaceae | G | T |
| *Pinus nigra* Arn. subsp. *pallasiana* (Lamb) Holmboe. | 2.4 [1670] | Leaf | 1600 m altitude | Pinaceae | G | T |
| *Pinus nigra* J.F. Arnold | 77.0 [1671] | Leaf | Leaf age: 1 year. Mean: 82.0 | Pinaceae | G | T |
| *Pinus nigra* J.F. Arnold | 86.0 [1672] | Leaf | Leaf age: 1 year. Mean: 82.0 | Pinaceae | G | T |
| *Pinus nigra* J.F. Arnold. | 105.0 [1673] | Leaf | Leaf age: 2 years. Mean: 106.0. | Pinaceae | G | T |
| *Pinus nigra* J.F. Arnold. | 111.0 [1674] | Leaf | Leaf age: 2 years. Mean: 106.0. | Pinaceae | G | T |
| Pinus pinaster Aiton | 197 [1675] | Aerial parts |  | Pinaceae | G | T |
| Pinus pinea L. | 510 [1676] | Aerial parts |  | Pinaceae | G | T |
| *Pinus pinea* L. | 393 [1677] | Leaf | From an urban area (Madrid). | Pinaceae | G | T |
| *Pinus sylvestris* L. | 102.4 [1678] | Leaf | Leaf age: 1 year. Mean: 98.0 | Pinaceae | G | T |
| *Pinus sylvestris* L. | 132.1 [1679] | Leaf | Leaf age: 2 years. Mean: 134.0 | Pinaceae | G | T |
| *Pinus sylvestris* L. | 136.8 [1680] | Leaf | Leaf age: 2 years. Mean: 134.0 | Pinaceae | G | T |
| *Pinus sylvestris* L. | 27 [1681] | Leaf |  | Pinaceae | G | T |
| *Pinus sylvestris* L. | 49 [1682] | Leaf |  | Pinaceae | G | T |
| *Pinus sylvestris* L. | 96 [1683] | Leaf | Leaf age: 1 year. Mean: 98.0 | Pinaceae | G | T |
| *Pinus taeda* L. | 46 [1684] | Leaf |  | Pinaceae | G | T |
| *Pinus thunbergii* Parl. | 39[ 1685] | Bark |  | Pinaceae | G | T |
| *Pinus thunbergii* Parl. | 0 [1686] | Wood | Under LOQ. | Pinaceae | G | T |
| *Pinus tabuliformis* Carrière | 203.6 [1687] | Leaf | From Beijing Botanica Garden (control). | Pinaceae | G | T |
| *Pinus tabuliformis* Carrière | 286.8 [1688] | Leaf | From hills near the Beijing Steel Factory. | Pinaceae | G | T |
| *Piper guianense* (Klotzsch) C.DC*.* | 149.2 [1689] | Fruit |  | Piperaceae | Mag | S |
| *Piper guineense* Schumach. & Thonn. | 0.10 [1690] | Seed |  | Piperaceae | Mag |  |
| *Piper guineense* Schumach. & Thonn. | 8.5 [1691] | Seed |  | Piperaceae | Mag | H |
| *Piper nigrum* L. | 155 [1692] | Fruit | Reported as “*Pepper nigrium”* | Piperaceae | Mag | V |
| *Piper nigrum* L. | 89.24 [1693] | Fruit |  | Piperaceae | Mag | V |
| *Piper nigrum* L. | 20.5 [1694] | Seed |  | Piperaceae | Mag | V |
| Piptatherum miliaceum (L.) Coss. | 550[ 1695] | Aerial parts |  | Poaceae | M | H |
| *Pipturus albidus* A.Gray ex H.Mann | 45.6 [1696] | Leaf |  | Urticaceae | D | T |
| *Pipturus albidus* A.Gray ex H.Mann | 53.6 [1697] | Leaf |  | Urticaceae | D | T |
| *Pistacia lentiscus* L. | 72.3 [1698] | Aerial parts |  | Anacardiaceae | D | T |
| *Pistia stratiotes* L. | 8339.2 [1699] | Leaf | Aquatic. | Araceae | M | H |
| *Pistia stratiotes* L. | 111212.0 [1700] | Root | Aquatic. | Araceae | M | H |
| *Pisum sativum* L. | 330.4 [1701] | Leaf |  | Fabaceae | D | H |
| *Pisum sativum* L. | 5.1 [1702] | Seed |  | Fabaceae | D | H |
| *Pittosporum tobira* (Thunb.) W.T.Aiton | 120 [1703] | Leaf | From an urban area (Madrid). | Pittosporaceae | D | S |
| *Pityrogramma calomelanos* (L.) Link | 17.37 [1704] | Leaf | From polluted (crude-oil contaminated) soil. | Pteridaceae | P | H |
| *Pityrogramma calomelanos* (L.) Link | 28.30 [1705] | Leaf | From unpolluted soil. | Pteridaceae | P | H |
| *Pityrogramma calomelanos* (L.) Link | 320 [1706] | Leaf | Reproductive stage | Pteridaceae | P | H |
| *Pityrogramma calomelanos* (L.) Link | 632 [1707] | Leaf | Vegetative stage | Pteridaceae | P | H |
| *Plagiothecium elegans* (Brid.) Schimp*.* | 300 [1708] | Aerial parts |  | Plagiotheciaceae | B | H |
| *Plantago lanceolata* L. | 223 [1709] | Aerial parts |  | Plantaginaceae | D | H |
| *Plantago major* L. | 89 [1710] | Aerial parts |  | Plantaginaceae | D | H |
| *Platanus orientalis* L. | 2.8 [1711] | Leaf | 1600 m altitude | Platanaceae | D | T |
| *Platycerium wallichii* Hook. | 210.3 [1712] | Leaf |  | Polypodiaceae | P | H |
| *Platycladus orientalis* (L.) Franco | 248.8 [1713] | Leaf | From Beijing Botanical Garden (control). | Cupressaceae | G | T |
| *Platycladus orientalis* (L.) Franco | 616.1 [1714] | Leaf | From hills near the Beijing Steel Factory. | Cupressaceae | G | T |
| *Platycodon grandiflorus -* (Jacq.)A.DC. | 8.2 [1715] | Root |  | Campanulaceae | D | H |
| *Pleurozium* *schreberi* (Willd. ex Brid.) Mitt. | 326 [1716] | Aerial parts. | “Only the green part” was used for analysis. Mean = 485. Pollution-free area. | Hylocomiaceae | B | H |
| *Pleurozium* *schreberi* (Willd. ex Brid.) Mitt. | 650 [1717] | Aerial parts. | “Only the green part” was used for analysis. Mean = 485. Pollution-free area. | Hylocomiaceae | B | H |
| *Pluchea* *indica* (L.) Less. | 227 [1718] | Leaf | Reported as “leaves (or shoots)”. Saline soil (pH 3.9) | Asteraceae | D | S |
| *Poa pratensis* L. | 218.0 [1719] | Leaf | From a high-rainfall area. | Poaceae | M | H |
| *Poa pratensis* L. | 45.7 [1720] | Stem | From a high-rainfall area. | Poaceae | M | H |
| *Poa pratensis* L. | 130.0 [1721] | Whole plant | From a high-rainfall area. | Poaceae | M | H |
| *Polygala pruinosa* Boiss. | 5.1 [1722] | Aerial parts | 1000 m altitude . Reported as *Polygalla pruinosa* L. | Polygalaceae | D | H |
| *Polygonatum verticillatum* (L.) All. | 204 [1723] | Aerial parts |  | Asparagaceae | M | H |
| *Polygonum molle* D. Don | 3170 [1724] | Shoot | New shoot | Polygonaceae | D | Su |
| *Polypodium vulgare* L. | 400 [1725] | Leaf |  | Polypodiaceae | P | H |
| Polypogon maritimus Willd. | 109 [1726] | Aerial parts | Reported as Polypogon maritimum L. | Poaceae | M | H |
| *Polyscias fulva* (Hiern) Harms | 128.8 [1727] | Young leaf | With petiole | Araliaceae | D | T |
| *Polyscias fulva* (Hiern) Harms | 227.7 [1728] | Young leaf | Without petiole | Araliaceae | D | T |
| *Populus tremula* var. *sieboldii* (Miq.) H.Ohashi | 42 [1729] | Bark | Reported as *Populus sieboldii.* | Salicaceae | D | T |
| *Populus tremula* var. *sieboldii* (Miq.) H.Ohashi | 51 [1730] | Bark | Reported as *Populus sieboldii.* | Salicaceae | D | T |
| *Populus tremula* var. *sieboldii* (Miq.) H.Ohashi | 0 [1731] | Wood | Reported as *Populus sieboldii*. Under LOQ. | Salicaceae | D | T |
| *Portulaca oleracea* L. | 29 | Leaf | Succulent. | Portulaceae | D | H |
| *Portulaca oleracea* L. | 33.21 [1733] | Leaf | Succulent. | Portulaceae | D | H |
| *Portulaca oleracea* L. | 869 [1734] | Leaf |  | Portulacaceae | D | H |
| *Portulaca oleracea* L. | 121.5 [1735] | Root |  | Portulaceae | D | H |
| *Potamogeton natans* L. | 330 [1736] | Leaf | Acquatic | Potamogetonaceae | M | H |
| *Potamogeton perfoliatus* L. | 550 [1737] | Leaf | Acquatic | Potamogetonaceae | M | H |
| *Potamogeton amplifolius* Tuck. | 1651 [1738] | Aerial parts | Aquatic | Potamogetonaceae | M | H |
| *Potamogeton crispus* L. | 820 [1739] | Leaf | Acquatic | Potamogetonaceae | M | H |
| *Potamogeton richardsonii* (A.Benn.) Rydb. | 218 [1740] | Aerial parts | Aquatic. | Potamogetonaceae | M | H |
| *Potamogeton* *alpinus* Balb. | 3030 [1741] | Leaf | Acquatic | Potamogetonaceae | M | H |
| *Potentilla anserina* L. | 399 [1742] | Aerial parts |  | Rosaceae | D | H |
| *Potentilla anserina* L. | 712 [1743] | Aerial parts |  | Rosaceae | D | H |
| *Poupartia chapelieri* (Guillaumin) H. Perrier | 93.4 [1744] | Leaf |  | Anacardiaceae | D | T |
| *Pouteria altissima* (A.Chev.) Baehni | 130.2 [1745] | Fruit | Reported as *Aningeria altissima* (A.Chev.) Aubrév. & Pellegr. | Sapotaceae | D | T |
| *Premna herbacea* Roxb. | 18.3 [1746] | Leaf |  | Lamiaceae | D | Su |
| *Primula vulgaris* Huds | 210 [1747] | Leaf |  | Primulaceae | D | H |
| *Primula vulgaris* Huds | 470 [1748] | Leaf |  | Primulaceae | D | H |
| *Prosopis africana* (Guill. & Perr.) Taub. | 43.8 [1749] | Seed | Reported (erroneously) as *Prosopos africana.* | Fabaceae | D | T |
| *Prosopis laevigata* (Willd.) M.C.Johnst. | 47 [1750] | Fruit |  | Fabaceae | D | T |
| *Prosopis laevigata* (Willd.) M.C.Johnst. | 155 [1751] | Leaf |  | Fabaceae | D | T |
| *Prunus africana* (Hook.f.) Kalkman | 131.5 [1752] | Bark |  | Rosaceae | D | T |
| *Prunus africana* (Hook.f.) Kalkman | 122.0 [1753] | Fruit |  | Rosaceae | D | T |
| *Prunus africana* (Hook.f.) Kalkman | 269.7 [1754] | Leaf | Young leaf. | Rosaceae | D | T |
| *Prunus africana* (Hook.f.) Kalkman | 99.8 [1755] | Leaf |  | Rosaceae | D | T |
| *Prunus africana* (Hook.f.) Kalkman | 267.4 [1756] | Seed |  | Rosaceae | D | T |
| *Prunus armeniaca* L. | 11.13 [1757] | Fruit |  | Rosaceae | D | T |
| *Prunus armeniaca* L. | 19.31 [1758] | Fruit |  | Rosaceae | D | T |
| *Prunus armeniaca* L. | 47.6 [1759] | Fruit | Declared only as “apricot” | Rosaceae | D | T |
| *Prunus armeniaca* L. | 48.0 [1760] | Fruit |  | Rosaceae | D | T |
| *Prunus armeniaca* L. | 6.96 | Fruit | Declared only as “apricot” | Rosaceae | D | T |
| *Prunus armeniaca* L. | 70.0 [1762] | Fruit |  | Rosaceae | D | T |
| *Prunus armeniaca* L. | 22.0 [1763] | Seed |  | Rosaceae | D | T |
| *Prunus avium* (L.) L. | 23.2 [1764] | Fruit | Declared only as “cherry” | Rosaceae | D | T |
| *Prunus domestica* L. | 19.0 [1765] | Fruit |  | Rosaceae | D | T |
| *Prunus domestica* L. | 24.5 [1766] | Fruit | Declared only as “plum” | Rosaceae | D | T |
| *Prunus domestica* L. | 32.4 [1767] | Fruit |  | Rosaceae | D | T |
| *Prunus leveilleana* Koehne | 103 [1768] | Leaf | Reported as *Prunus verecunda* (Koidz.) Koehne. | Rosaceae | D | T |
| *Prunus mume* (Siebold.) Siebold.&Zucc. | 13.4 [1769] | Fruit |  | Rosaceae | D | T |
| *Prunus persica* (L.)Batsch. | 40.0 [1770] | Fruit | Declared only as “peach” | Rosaceae | D | T |
| *Prunus persica* (L.)Batsch. | 6.5 [1771] | Fruit |  | Rosaceae | D | T |
| *Prunus sargentii* Rehder | 120 [1772] | Bark |  | Rosaceae | D | T |
| *Prunus sargentii* Rehder | 42 [1773] | Bark |  | Rosaceae | D | T |
| *Prunus sargentii* Rehder | 0 [1774] | Wood | Under LOQ. | Rosaceae | D | T |
| *Prunus* *cerasoides* Buch.-Ham. ex D.Don | 2110 [1775,1776] | Fruit | Reported as *Prunus cerasoides* D. Don | Rosaceae | D | T |
| *Pseudolarix amabilis* (J.Nelson) Rehder | 344.6 [1777] | Leaf |  | Pinaceae | G | T |
| *Pseudoscleropodium purum* (Hedw.) M. Fleisch. | 6275 [1778] | Whole plant | Mean:3022.7; Median: 2466.2 Reported as *Scleropodium purum* (Hedw.) Limpr. From an area in the vicinity of a steel plant. | Brachytheciaceae | B | H |
| *Pseudoscleropodium purum* (Hedw.) M. Fleisch. | 793 [1779] | Whole plant | Mean:3022.7; Median: 2466.2 Reported as *Scleropodium purum* (Hedw.) Limpr. From an area in the vicinity of a steel plant. | Brachytheciaceae | B | H |
| *Pseudospondias microcarpa* (A.Rich.) Engl. | 67.0 [1780] | Seed |  | Anacardiaceae | D | T |
| *Pseudotsuga menziesii* (Mirb.) Franco | 0 [1781] | Wood | Under LOQ. | Pinaceae | G | T |
| *Psidium guajava* L. | 201.2 [1782] | Fruit |  | Myrtaceae | D | T |
| *Psidium guajava* L. | 146 [1783] | Leaf | From a mine contaminated area. | Myrtaceae | D | T |
| *Psidium guajava* L. | 391 [1784] | Leaf | From a mine contaminated area. | Myrtaceae | D | T |
| *Psilopeganum sinense* Hemsl. | 361.0 [1785] | Leaf |  | Rutaceae | D | T |
| *Psophocarpus tetragonolobus* (L.) DC. | 0 [1786] | Seed |  | Fabaceae | D | H |
| *Pteridium* *aquilinum* (L.) Kuhn | 101 [1787] | Aerial parts | “Matured bracken” | Dennstaedtiaceae | P | H |
| *Pteridium* *aquilinum* (L.) Kuhn | 126 [1788] | Aerial parts | “Matured bracken” | Dennstaedtiaceae | P | H |
| *Pteridium* *aquilinum* (L.) Kuhn | 103 | Leaf | On granite soil. Mean = 122 (plant-available Fe in soil (min.-max.; mean), 0.06–0.45; 0.24) | Dennstaedtiaceae | P | H |
| *Pteridium aquilinum* (L.) Kuhn | 115 [1790] | Leaf | Mean = 88. Pollution-free area. | Dennstaedtiaceae | P | H |
| *Pteridium* *aquilinum* (L.) Kuhn | 119 [1791] | Leaf | On serpentine soil. Mean = 98 (plant-available Fe in soil (min-max.; mean), 3.3–5.5; 4.2 ppm). | Dennstaedtiaceae | P | H |
| *Pteridium* *aquilinum* (L.) Kuhn | 160 [1792] | Leaf | On granite soil. Mean = 122 (plant-available Fe in soil (min.-max.; mean), 0.06–0.45; 0.24) | Dennstaedtiaceae | P | H |
| *Pteridium* *aquilinum* (L.) Kuhn | 19.76 [1793] | Leaf |  | Dennstaedtiaceae | P | H |
| *Pteridium* *aquilinum* (L.) Kuhn | 200 [1794] | Leaf | Median = 70. | Dennstaedtiaceae | P | H |
| *Pteridium* *aquilinum* (L.) Kuhn | 50 [1795] | Leaf | Median = 70. | Dennstaedtiaceae | P | H |
| *Pteridium aquilinum* (L.) Kuhn | 67 [1796] | Leaf | Mean = 88. Pollution-free area. | Dennstaedtiaceae | P | H |
| *Pteridium* *aquilinum* (L.) Kuhn | 85 [1797] | Leaf | On serpentine soil. Mean = 98 (plant-available Fe in soil, (min-max.; mean), 3.3-5.5; 4.2 ppm). | Dennstaedtiaceae | P | H |
| *Pteridium aquilinum* (L.) Kuhn | 119.2 [1798] | Shoot | From an area around an abandoned mine. | Dennstaedtiaceae | P | H |
| *Pteridium* *aquilinum* (L.) Kuhn | 120 [1799] | Shoot | “Shoot sporut bracken” | Dennstaedtiaceae | P | H |
| *Pteridium* *aquilinum* (L.) Kuhn | 128 [1800] | Shoot | “Shoot sporut bracken” | Dennstaedtiaceae | P | H |
| *Pteridium aquilinum* (L.) Kuhn | 232.4 [1801] | Shoot | From an area around an abandoned mine. | Dennstaedtiaceae | P | H |
| *Pteridium aquilinum* (L.) Kuhn | 50 [1802] | Whole plant | Mean = 75. Pollution-free area. | Dennstaedtiaceae | P | H |
| *Pteridium aquilinum* (L.) Kuhn | 82 [1803] | Whole plant | Mean = 75. Pollution-free area. | Dennstaedtiaceae | P | H |
| *Pteridium aquilinum* (L.) Kuhn | 33 [1804] | Stem | Mean = 39. Pollution-free area. | Dennstaedtiaceae | P | H |
| *Pteridium aquilinum* (L.) Kuhn | 42 [1805] | Stem | Mean = 39. Pollution-free area. | Dennstaedtiaceae | P | H |
| *Pteridium* *aquilinum* (L.) Kuhn | 752 [1806] | Aerial parts | Reported as *Pteridium aquilinum* (L.) Khun ssp. *aquilinum*. | Dennstaedtiaceae | P | H |
| *Pteridium aquilinum* (L.) Kuhn | 260 [1807] | Leaf |  | Dennstaedtiaceae | P | H |
| *Pteridium arachnoideum* (Kaulf.) Maxon | 128.5 [1808] | Aerial parts |  | Dennstaedtiaceae | P | H |
| *Pteris vittata* L. | 130.4 [1809] | Aerial parts |  | Pteridaceae | P | H |
| *Pteris vittata* L. | 103.4 [1810] | Leaf | Reproductive stage | Pteridaceae | P | H |
| *Pteris vittata* L. | 200 [1811] | Leaf | Vegetative stage | Pteridaceae | P | H |
| *Pteris vittata* L. | 546.2 [1812] | Leaf | 59,000.0 mg/kg iron in associated grey-green tailings deposited in the Copperbelt impoundments. | Pteridaceae | P | H |
| *Pteris vittata* L. | 663.0 [1813] | Leaf | 250,571.01 in associated reddish-brown tailings deposited in the Copperbelt impoundments. | Pteridaceae | P | H |
| *Pterocarya stenoptera* C. DC. | 270.4 [1814] | Leaf | Reported as *Pterocarya stenoptera* var.*zhijiangensis*. | Juglandaceae | M | T |
| *Pteroceltis tatarinowii* Maxim. | 297.4 [1815] | Leaf |  | Cannabaceae | D | T |
| *Pterolobium hexapetalum* (Roth) Santapau & Wagh | 104.9 [1816] | Aerial parts |  | Fabaceae | D | S |
| *Pterostyrax psilophyllus* Diels ex Perkins | 447.5 [1817] | Leaf |  | Styracaceae | D | T |
| *Pueraria montana* (Lour.) Merr. | 1.9 [1818] | Root | Reported as *Pueraria montana lobata* (Willd.) Sanjappa & Pradeep. | Fabaceae | D | H |
| *Pueraria tuberosa* (Willd.) DC | 500 [1819] | Stem |  | Fabaceae | D | V |
| Pulicaria arabica subsp. hispanica (Boiss.) Murb. | 698 [1820] | Aerial parts | Reported as Pulicaria paludosa Link. | Asteraceae | D | H |
| *Punica granatum* L. | 3.0 [1821] | Fruit |  | Lythraceae | D | T |
| *Quercus acuta* Thunb. | 130 [1822] | Bark |  | Fagaceae | D | T |
| *Quercus acuta* Thunb. | 21 [1823] | Bark |  | Fagaceae | D | T |
| *Quercus acuta* Thunb. | 0 [1824] | Wood |  | Fagaceae | D | T |
| *Quercus acutissima* Carruth. | 15 [1825] | Bark |  | Fagaceae | D | T |
| *Quercus acutissima* Carruth. | 57 [1826] | Bark |  | Fagaceae | D | T |
| *Quercus acutissima* Carruth. | 0 [1827] | Wood | Under LOQ. | Fagaceae | D | T |
| *Quercus cerris* L | 6.9 [1828] | Leaf | 1000 m altitude Reported as *Quercus cerris* L. subsp. *cerris* | Fagaceae | D | T |
| *Quercus eduardi* Trel. | 154 [1829] | Leaf |  | Fagaceae | D | T |
| *Quercus grisea* Liebm. | 208 [1830] | Leaf |  | Fagaceae | D | T |
| *Quercus mongolica* subsp. *crispula* (Blume) Menitsky | 12 [1831] | Bark | Reported as *Quercus crispula* Blume. | Fagaceae | D | T |
| *Quercus mongolica* subsp. *crispula* (Blume) Menitsky | 25 [1832] | Bark | Reported as *Quercus crispula* Blume. | Fagaceae | D | T |
| *Quercus mongolica subsp. crispula* (Blume) Menitsky | 107 [1833] | Leaf | Reported as *Quercus mongolica* var. *grosseserrata* (Blume) Rehder & E.H.Wilson | Fagaceae | D | T |
| *Quercus mongolica* subsp. *crispula* (Blume) Menitsky | 3 [1834] | Wood | Reported as *Quercus crispula* Blume.Sapwood. | Fagaceae | D | T |
| *Quercus mongolica* subsp. *crispula* (Blume) Menitsky | 3.1 [1835] | Wood | Reported as *Quercus crispula* Blume.Wood. | Fagaceae | D | T |
| Quercus rotundifolia Lam. | 227 [1836] | Aerial parts |  | Fagaceae | D | H |
| *Quercus* *serrata* Murray | 180 [1837] | Bark |  | Fagaceae | D | T |
| *Quercus* *serrata* Murray | 36 [1838] | Bark |  | Fagaceae | D | T |
| *Quercus serrata Murray* | 139 [1839] | Leaf |  | Fagaceae | D | T |
| *Quercus* *serrata* Murray | 35 [1840] | Wood | Sapwood | Fagaceae | D | T |
| Quercus suber L. | 636 [1841] | Aerial parts |  | Fagaceae | D | H |
| *Quercus* *petraea* (Matt.) Liebl. | 200 [1842] | Leaf |  | Fagaceae | D | T |
| *Ranunculus macullatus* Cockayne & Allan | 85.0 [1843] | Whole plant | Reported as Ranunculus mariculatus, most likely an erroneous rendition of *Ranunculus macullatus* or (less likely) *Ranunculus marginatus* d’Urv. | Ranunculaceae | D | H |
| *Raphanus sativus* L. | 35.7 [1844] | Leaf |  | Brassicaceae | D | H |
| *Raphanus raphanistrum* subsp*. sativus* (L.) Domin | 643.1 [1845] | Leaf | Reported as *Raphanus sativus* L. | Brassicaeae | D | H |
| *Rauvolfia serpentina* (L.) Benth. ex Kurz | 18.5 [1846] | Leaf |  | Apocynaceae | D | S |
| *Ravenea sambiranensis* Jum. & H.Perrier | 137.7 [1847] | Fruit | See comment to *Dypsis lastelliana.* | Arecaceae | M | T* |
| *Rhagadiolus stellatus* (L.) Gaertn. | 50 [1848] | Seed |  | Asteraceae | D | H |
| *Rhamnus parvifolia* Bunge | 404.4 [1849] | Leaf | From Beijing Botanica Garden (control). | Rhamnaceae | D | T |
| *Rhamnus parvifolia* Bunge | 537.4 [1850] | Leaf | From hills near the Beijing Steel Factory. | Rhamnaceae | D | T |
| *Rheum emodi* Wall. | 4507 [1851] | Root |  | Polygonaceae | D | H |
| *Rhododendron dilatatum* Miq. | 116 [1852] | Leaf |  | Ericaceae | D | T |
| *Rhododendron semibarbatum* Maxim. | 224 [1853] | Leaf |  | Ericaceae | D | S |
| *Rhopalocarpus alternifolius* (Baker) Capuron | 52.6 [1854] | Fruit |  | Sphaerosepalaceae | D | T |
| *Rhus coriaria* L. | 241.24 [1855] | Fruit |  | Anacardiaceae | D | T |
| *Rhus coriaria* L. | 4.5 [1856] | Leaf | 1000 m altitude | Anacardiaceae | D | S |
| *Rhus taratana* (Baker) H. Perrier | 75.7 [1857] | Fruit |  | Anacardiaceae | D | T |
| *Ribes lacustre* (Pers.) Poir. | 50 [1858] | Fruit |  | Grossulariaceae | D | S |
| *Ricinus communis* L. | 508.4 [1859] | Leaf | From a paper mill contaminated area. | Ephorbiaceae | D | T |
| *Ricinus communis* L. | 1401.8 [1860] | Root | From a paper mill contaminated area. | Ephorbiaceae | D | T |
| *Ricinus communis* L. | 397 [1861] | Seed |  | Euphorbiaceae | D | S |
| *Ricinus communis* L. | 313.4 [1862] | Stem | From a paper mill contaminated area. | Ephorbiaceae | D | T |
| *Robinia pseudoacacia* L. | 161.0 [1863] | Leaf | From Beijing Botanica Garden (control). | Fabaceae | D | T |
| *Robinia pseudoacacia* L. | 251.7 [1864] | Leaf | From hills near the Beijing Steel Factory. | Fabaceae | D | T |
| *Rosa abyssinica* R.Br. | 104 [1865] | Fruits |  | Rosaceae | D | S |
| *Rosa abyssinica* R.Br. | 273 [1866] | Fruits |  | Rosaceae | D | S |
| *Rosa canina* L. | 13.16 [1867] | Fruit |  | Rosaceae | D | S |
| *Rosa canina* L. | 14.8 | Fruit | Described as “dried receptacles”. | Rosaceae | D | S |
| *Rosa canina* L. | 267.2 | Fruit | Described as “dried receptacles”. | Rosaceae | D | S |
| *Rosa canina* L. | 8.85 | Fruit | Described as “dried receptacles”. 78^[1871]^-106- herbal part not stated | Rosaceae | D | S |
| *Rosmarinus eriocalyx* Jord. & Fourr. | 12200 [1873] | Leaf | Reported as *Rosmarinus tournefortii* (Noë ex Jord. & Fourr.) Jahand. & Maire. | Lamiaceae | D | Su |
| Rosmarinus officinalis L. | 331 [1874] | Aerial parts |  | Lamiaceae | D | Su |
| *Rosmarinus officinalis* L. | 32 [1875] | Leaf | 501reported by S. Tokalioglu, but herbal parts not stated. | Lamiaceae | D | Su |
| *Rosmarinus officinalis* L. | 383 [1877] | Leaf |  | Lamiaceae | D | Su |
| *Rosmarinus officinalis* L. | 398.7 [1878] | Leaf |  | Lamiaceae | D | Su |
| *Rothmannia urcelliformis* (Hiern) Bullock ex Robyns | 69.8 [1879] | Fruit |  | Rubiaceae | D | T |
| *Rothmannia urcelliformis* (Hiern) Bullock ex Robyns | 120.9 [1880] | Leaf | Young leaf | Rubiaceae | D | T |
| *Rubia cordifolia* L. | 402.3 [1881] | Leaf | From Beijing Botanica Garden (control). | Rubiaceae | D | H |
| *Rubia cordifolia* L. | 619.6 [1882] | Leaf | From hills near the Beijing Steel Factory. | Rubiaceae | D | H |
| *Rubus amabilis* Focke | 152.4 [1883] | Fruit |  | Rosaceae | D | Su |
| *Rubus amabilis* Focke | 494.2 [1884] | Leaf |  | Rosaceae | D | Su |
| *Rubus amabilis* Focke | 691.0 [1885] | Root |  | Rosaceae | D | Su |
| *Rubus amabilis* Focke | 140.8 [1886] | Stem |  | Rosaceae | D | Su |
| *Rubus idaeus* L. | 1944 [1887] | Leaf |  | Rosaceae | D | Su |
| *Rubus parviflorus* Nutt. | 30 [1888] | Fruit |  | Rosaceae | D | S |
| *Rubus spectabilis* Pursh. | 50 [1889] | Fruit |  | Rosaceae | D | S |
| *Rubus ursinus* Cham. & Schltdl. | 40 [1890] | Fruit |  | Rosaceae | D | S |
| *Rumex acetosa* L. | 67 [1891] | Aerial parts |  | Polygonaceae | D | H |
| Rumex acetosella L. | 688.2 [1892] | Leaf |  | Polygonaceae | D | H |
| Rumex acetosella L. | 491.1 [1893] | Root |  | Polygonaceae | D | H |
| *Rumex bucephalophorus* L. | 130 [1894] | Seed |  | Polygonaceae | D | H |
| Rumex bucephalophorus subsp. gallicus (Steinh.) Rchb.f. | 547 [1895] | Aerial parts | Reported as Rumex bucephalophorus subsp. gallicus (Steinh.) Rech. f. | Polygonaceae | D | H |
| *Rumex crispus* L. | 75.7 [1896] | Leaf |  | Polygonaceae | D | H |
| Rumex obtusifolius L. | 1761 [1897] | Leaf | Control soil without any additive. | Polygonaceae | D | H |
| Rumex obtusifolius L. | 2092 [1898] | Leaf | Soil with lime. | Polygonaceae | D | H |
| Rumex obtusifolius L. | 216 [1899] | Leaf | Non-contaminated control soil. | Polygonaceae | D | H |
| Rumex obtusifolius L. | 4456 [1900] | Leaf | Control soil without any additive. | Polygonaceae | D | H |
| Rumex obtusifolius L. | 5273 [1901] | Leaf | Soil with lime. | Polygonaceae | D | H |
| Rumex obtusifolius L. | 5357 [1902] | Leaf | Soil with superphosphate. | Polygonaceae | D | H |
| Rumex obtusifolius L. | 795 [1903] | Leaf | Soil with superphosphate | Polygonaceae | D | H |
| Rumex obtusifolius L. | 1857 [1904] | Root | Soil with lime.  Underground organs. | Polygonaceae | D | H |
| Rumex obtusifolius L. | 2337 [1905] | Root | Soil with superphosphate. Underground organs. | Polygonaceae | D | H |
| Rumex obtusifolius L. | 2426 [1906] | Root | Soil with superphosphate. Underground organs. | Polygonaceae | D | H |
| Rumex obtusifolius L. | 2463 [1907] | Root | Control soil without any additive. Underground organs. | Polygonaceae | D | H |
| Rumex obtusifolius L. | 432 [1908] | Root | Non-contaminated control soil. Root and belowground organs | Polygonaceae | D | H |
| Rumex obtusifolius L. | 4547.5 [1909] | Root | Control soil without any additive. Root and belowground organs | Polygonaceae | D | H |
| Rumex obtusifolius L. | 965 [1910] | Root | Soil with lime. Underground organs. | Polygonaceae | D | H |
| Rumex obtusifolius L. | 315 [1911] | Seed | Soil with lime. | Polygonaceae | D | H |
| Rumex obtusifolius L. | 52 [1912] | Seed | Non-contaminated control soil. | Polygonaceae | D | H |
| Rumex obtusifolius L. | 758 [1913] | Seed | Soil with superphosphate. | Polygonaceae | D | H |
| Rumex obtusifolius L. | 82 [1914] | Seed | Soil with lime. | Polygonaceae | D | H |
| Rumex obtusifolius L. | 926 [1915] | Seed | Control soil without any additive. | Polygonaceae | D | H |
| Rumex obtusifolius L. | 171 [1916] | Stem | Soil with lime. | Polygonaceae | D | H |
| Rumex obtusifolius L. | 251.5 [1917] | Stem | Control soil without any additive. | Polygonaceae | D | H |
| Rumex obtusifolius L. | 441 [1918] | Stem | Soil with superphosphate. | Polygonaceae | D | H |
| Rumex obtusifolius L. | 51.5 [1919] | Stem | Soil with lime | Polygonaceae | D | H |
| Rumex obtusifolius L. | 62 [1920] | Stem | Non-contaminated control soil. | Polygonaceae | D | H |
| Rumex scutatus subsp. induratus (Boiss. & Reut.) Nyman | 316 [1921] | Aerial parts | Reported as Rumex induratus Boiss. & Reut. | Polygonaceae | D | H |
| Rumex vesicarius L. | 241 [1922] | Aerial parts |  | Polygonaceae | D | H |
| Rumex vesicarius L. | 425 [1923] | Aerial parts |  | Polygonaceae | D | H |
| *Sagittaria cuneata* E.Sheld. | 1904 [1924] | Aerial parts |  | Alismataceae | M | H |
| *Sagittaria rigida* Pursh | 2083 [1925] | Aerial parts |  | Alismataceae | M | H |
| *Sagittaria sagittifolia* L. | 24069.3 [1926] | Leaf | Aquatic. | Alismataceae | M | H |
| *Sagittaria sagittifolia* L. | 97358.8 [1927] | Root | Aquatic. | Alismataceae | M | H |
| *Sagittaria sagittifolia* L. | 2719.8 [1928] | Stem | Aquatic. | Alismataceae | M | H |
| *Sagittaria trifolia* L. | 8 [1929] | Root | Aquatic. | Alismataceae | M | H |
| *Salicornia patula* Duval-Jouve | 735 [1930] | Aerial parts |  | Amaranthaceae | D | H |
| *Salix alba* L. | 24.5 [1931] | Leaf |  | Salicaceae | D | T |
| *Salix atrocinerea* Brot. | 260 [1932] | Leaf |  | Salicaceae | D | S |
| *Salix atrocinerea* Brot. | 21.0 [1933] | Shoot | From an area around an abandoned mine. | Salicaceae | D | T |
| *Salix atrocinerea* Brot. | 95.9 [1934] | Shoot | From an area around an abandoned mine. | Salicaceae | D | T |
| Salix martiana Leyb. | 206.5 [1935] | Leaf | Old leaf. FeSO_4_ (50 microM) as iron source. Aerobic conditions in hydroponic culture. | Salicaceae | D | T |
| *Salix martiana* Leyb. | 212.0 [1936] | Leaf | Young leaf. FeSO_4_  (50 microM)as iron source. Aerobic conditions in hydroponic culture. | Salicaceae | D | T |
| *Salix martiana* Leyb. | 373.9 [1937] | Leaf | Young leaf. FeSO_4_  (250 microM) as iron source. Hypoxic conditions in hydroponic culture. | Salicaceae | D | T |
| *Salix martiana* Leyb. | 457.6 [1938] | Leaf | Old leaf. FeSO_4_ (250 microM) as iron source. Hypoxic conditions in hydroponic culture. | Salicaceae | D | T |
| *Salix martiana* Leyb. | 837.0 [1939] | Leaf | Old leaf. FeSO_4_  (500 microM) as iron source. Hypoxic conditions in hydroponic culture. | Salicaceae | D | T |
| *Salix martiana* Leyb. | 848.2 [1940] | Leaf | Young leaf. FeSO_4_ (500 microM) as iron source. Hypoxic conditions in hydroponic culture. | Salicaceae | D | T |
| *Salix martiana* Leyb. | 876.1 [1941] | Leaf | Young leaf. FeEDTA (40 microM) as iron source. Aerobic conditions in hydroponic culture. | Salicaceae | D | T |
| Salix martiana Leyb. | 898.4 [1942] | Leaf | Old leaf. FeEDTA (40 microM) as iron source. Aerobic conditions in hydroponic culture. | Salicaceae | D | T |
| Salix martiana Leyb. | 1919.5 [1943] | Root | FeEDTA (40 microM) as iron source. Aerobic conditions in hydroponic culture. | Salicaceae | D | T |
| Salix martiana Leyb. | 43033 [1944] | Root | FeSO_4_ (50 microM) as iron source. Aerobic conditions in hydroponic culture. | Salicaceae | D | T |
| Salix martiana Leyb. | 67328.3 [1945] | Root | FeSO_4_ (250 microM) as iron source. Hypoxic conditions in hydroponic culture. | Salicaceae | D | T |
| Salix martiana Leyb. | 90825.7 [1946] | Root | FeSO_4_ (500 microM). Hypoxic conditions in hydroponic culture. | Salicaceae | D | T |
| Salix martiana Leyb. | 1495.4 [1947] | Shoots | FeSO_4_ (250 microM) as iron source. Hypoxic conditions in hydroponic culture. | Salicaceae | D | T |
| Salix martiana Leyb. | 2723.0 [1948] | Shoots | FeSO_4_ (500 microM) as iron source. Hypoxic conditions in hydroponic culture. | Salicaceae | D | T |
| Salix martiana Leyb. | 301.3 [1949] | Shoots | FeEDTA (40 microM) as iron source. Aerobic conditions in hydroponic culture. | Salicaceae | D | T |
| Salix martiana Leyb. | 747.7 [1950] | Shoots | FeSO_4_ (50 microM) as iron source. Aerobic conditions in hydroponic culture. | Salicaceae | D | T |
| Salix pedicellata Desf. | 212 [1951] | Aerial parts |  | Salicaceae | D | T |
| *Salix* *×* *fragilis* L. | 370 [1952] | Leaf | Reported as *Salix × fragilis* var*decipiens* W.D.J.Koch | Salicaceae | D | T |
| *Salix* *aurita* L. | 170 [1953] | Leaf |  | Salicaceae | D | S |
| *Salsola orientalis* S.G. Gmel. | 230 [1954] | Aerial parts | Reported as *Salsola rigida* Pall*.* | Amaranthaceae | D | Su |
| *Salsola tomentosa* (Moq.) Spach | 305 [1955] | Aerial parts |  | Amaranthaceae | D | Su |
| *Salvadora persica* L. | 500 [1956] | Aerial parts | These are approximations, as the authors expressed the values in grams/kg (including the iron in the group of “macro elements”) instead of the usual mg/kg or ppm. | Salvadoraceae | D | T |
| *Salvia aucheri* Benth. | 98.33 [1957] | Leaf |  | Lamiaceae | D | Su |
| *Salvia fruticosa* Mill. | 330.44 [1958] | Leaf | Reported as  “*Salvia fruticase* L.” |  |  | Su |
| *Salvia officinalis* L. | 1118.6 [1959] | Leaf |  | Lamiaceae | D | Su |
| *Salvia officinalis* L. | 133.8 [1960] | Leaf |  | Lamiaceae | D | Su |
| *Salvia* *officinalis* L. | 152 | Leaf |  | Lamiaceae | D | Su |
| *Salvia officinalis* L. | 30.9 [1962] | Leaf |  | Lamiaceae | D | Su |
| *Salvia officinalis* L. | 450 [1963] | Leaf |  | Lamiaceae | D | Su |
| *Salvia officinalis* L. | 67.1 [1964] | Leaf |  | Lamiaceae | D | Su |
| *Salvia* *officinalis* L. | 889 | Leaf |  | Lamiaceae | D | Su |
| *Sambucus cerulea* Raf. | 50 [1966] | Fruit |  | Adoxaceae | D | T |
| *Sambucus racemosa* L. | 70 [1967] | Fruit |  | Adoxaceae | D | T |
| Sanguisorba hybrida (L.) Font Quer | 225 [1968] | Aerial parts |  | Rosaceae | D | Su |
| *Sanguisorba officinalis* L. | 325 [1969] | Aerial parts |  | Rosaceae | D | H |
| *Sanguisorba officinalis* L. | 59 [1970] | Aerial parts |  | Rosaceae | D | H |
| *Santolina rosmarinifolia* L*.* | 49.6 [1971] | Shoot | From an area around an abandoned mine. | Asteraceae | D | H |
| *Santolina rosmarinifolia* L*.* | 51.7 [1972] | Shoot | From an area around an abandoned mine. | Asteraceae | D | H |
| *Saposhnikovia divaricata* (Turcz.) Schichk | 27.1 [1973] | Root |  | Apiaceae | D | H |
| *Saposhnikovia divaricata* (Turcz.) Schichk | 425.3 [1974] | Root |  | Apiaceae | D | H |
| *Sarcocephalus latifolius* (Sm.) E.A.Bruce | 18.0 [1975] | Fruit | Reported as *Nuclea latifolia* [*Nauclea latifolia* Sm.]. | Rubiaceae | D | T |
| *Sarcocornia perennis* (Mill.) A.J.Scott | 190 [1976] | Aerial parts |  | Amaranthaceae | D | S |
| *Sarracenia purpurea* L. | 42.7 [1977] | Leaf | Carnivorous. Trap leaves. | Sarraceniaceae | D | H |
| *Sasa borealis* (Hack.) Makino & Shibata | 188 [1978] | Leaf |  | Poaceae | M | H |
| *Satureja hortensis* L. | 630.20 [1979] | Leaf |  | Lamiaceae | D | H |
| *Saxifraga tridactylites* L. | 1439.6 [1980] | Leaf | Reported in mmol/kg, converted by us in mg/kg. | Saxifragaceae | D | H |
| *Scandix pecten-veneris* L. | 30 [1981] | Seed | These are approximations, as the authors expressed the values in % (wt/wt). | Apiaceae | D | H |
| *Schoenoplectus americanus* (Pers.) Volkart | 300 [1982] | Leaf | Reported as *Scirpus americanus*. Values are only approximations, as the authors provided graphs but not labels with the accurate values. From a site intensely polluted with heavy metals, including iron. Aquatic. | Cyperaceae | M | H |
| *Schoenoplectus americanus* (Pers.) Volkart | 380 [1983] | Leaf | Reported as *Scirpus americanus*. Values are only approximations, as the authors provided graphs but not labels with the accurate values. From a site intensely polluted with heavy metals, including iron. Aquatic. | Cyperaceae | M | H |
| *Schoenoplectus americanus* (Pers.) Volkart | 1000 [1984] | Root | Reported as *Scirpus americanus*. Values are only approximations, as the authors provided graphs but not labels with the accurate values. From a site intensely polluted with heavy metals, including iron. Aquatic. | Cyperaceae | M | H |
| *Schoenoplectus americanus* (Pers.) Volkart | 1200 [1985] | Root | Reported as *Scirpus americanus*. Values are only approximations, as the authors provided graphs but not labels with the accurate values. From a site intensely polluted with heavy metals, including iron. Aquatic. | Cyperaceae | M | H |
| *Schoenoplectus americanus* (Pers.) Volkart | 510 [1986] | Root | Reported as *Scirpus americanus*. Values are only approximations, as the authors provided graphs but not labels with the accurate values. From a site intensely polluted with heavy metals, including iron. Aquatic. | Cyperaceae | M | H |
| *Schoenoplectus americanus* (Pers.) Volkart | 600 [1987] | Root | Reported as *Scirpus americanus*. Values are only approximations, as the authors provided graphs but not labels with the accurate values. From a site intensely polluted with heavy metals, including iron. Aquatic. | Cyperaceae | M | H |
| *Schoenoplectus americanus* (Pers.) Volkart | 300 [1988] | Stem | Reported as *Scirpus americanus*. Values are only approximations, as the authors provided graphs but not labels with the accurate values. From a site intensely polluted with heavy metals, including iron. Aquatic. | Cyperaceae | M | H |
| *Schoenoplectus americanus* (Pers.) Volkart | 550 [1989] | Stem | Reported as *Scirpus americanus*. Values are only approximations, as the authors provided graphs but not labels with the accurate values. From a site intensely polluted with heavy metals, including iron. Aquatic. | Cyperaceae | M | H |
| Scirpoides holoschoenus (L.) Soják | 199 [1990] | Aerial parts | Reported as Scirpoides holoschoenus (L.). | Cyperaceae | M | H |
| *Scleria* *sumatrenis* Retz. | 10 [1991] | Leaf | Reported as “leaves (or shoots)”. Peat soil (pH 3.6). | Cyperaceae | M | H |
| *Secale cereale* L. | 4.0 [1992] | Seed | Most likely caryopse. | Poaceae | M | H |
| *Securidaca longipedunculata* Fresen. | 45 [1993] | Bark | Bark. | Polygalaceae | D | T |
| *Securidaca longipedunculata* Fresen. | 175 [1994] | Leaf |  | Polygalaceae | D | T |
| *Securidaca longipedunculata* Fresen. | 677 [1995] | Stem |  | Polygalaceae | D | T |
| *Sedum acre* L. | 385.0 [1996] | Leaf | Reported in mmol/kg, converted by us in mg/kg. | Crassulaceae | D | H |
| *Seidlitzia rosmarinus* Bunge ex Boiss. | 450 [1997] | Aerial parts |  | Amaranthaceae | D | S |
| *Selaginella delicatula* (Desv. ex Poir.) Alston | 400 [1998] | Leaf | Vegetative stage | Selaginellaceae | P | H |
| *Selaginella myosurus* Alston | 39.82 [1999] | Leaf | From unpolluted soil. | Selaginellaceae | P | H |
| *Selaginella myosurus* Alston | 64.96 [2000] | Leaf | From polluted (crude-oil contaminated) soil. | Selaginellaceae | P | H |
| *Selaginella delicatula* (Des. ex Poir.) Alston | 720 [2001] | Leaf | Reproductive stage | Selaginellaceae | P | H |
| *Senna auriculata* (L.) Roxb. | 224.5 [2002] | Flower | Reported as *Cassia  Auriculata* L. | Fabaceae | D | T |
| *Senna occidentalis* (L.) Link | 1518 [2003] | Whole plant | Reported as *Cassia occidentalis.* | Fabaceae | D | S |
| *Senna* *tora* (L.) Roxb. | 565 [2004] | Leaf | Reportes as *Cassia tora* L. | Fabaceae | D | H |
| *Senna wislizeni* (A.Gray) H.S.Irwin & Barneby | 239 [2005] | Leaf |  | Fabaceae | D | S |
| *Sericocomopsis pallida* Schinz | 200 [2006] | Aerial parts | These are approximations, as the authors expressed the values in grams/kg (including the iron in the group of “macro elements”) instead of the usual mg/kg or ppm. | Amaranthaceae | D | S |
| *Sesamothamnus busseanus* Engl. | 100 [2007] | Aerial parts | These are approximations, as the authors expressed the values in grams/kg (including the iron in the group of “macro elements”) instead of the usual mg/kg or ppm. | Pedaliaceae | D | S |
| *Sesamum indicum* L. | 53.53 [2008] | Seed |  | Pedaliaceae | D | H |
| *Sesleria* *caerulea* (L.) Ard. | 280 [2009] | Aerial parts |  | Poaceae | M | H |
| *Sesuvium portulacastrum* (L.) L. | 503.21 [2010] | Leaf | Reported as *Sesuvium portulacastrum* L. (Semi)-aquatic. Succulent. | Aizoaceae | D | H |
| *Sesuvium portulacastrum* (L.) L. | 986.1 [2011] | Root | Reported as *Sesuvium portulacastrum* L. (Semi)-aquatic. | Aizoaceae | D | H |
| *Sesuvium portulacastrum* (L.) L. | 10922.2 [2012] | Stem | Reported as *Sesuvium portulacastrum* L. (Semi)-aquatic. Succulent. | Aizoaceae | D | H |
| *Setaria viridis* (L.) P.Beauv. | 2210 [2013] | Leaf |  | Poaceae | M | H |
| *Setaria viridis* (L.)P.Beauv. | 323.0 [2014] | Leaf | From Beijing Botanica Garden (control). | Poaceae | M | H |
| *Setaria viridis* (L.)P.Beauv. | 560.5 [2015] | Leaf | From hills near the Beijing Steel Factory. | Poaceae | M | H |
| *Shorea negrosensis* Foxw*.* | 0 [2016] | Wood | Under LOQ. | Dipterocarpaceae | D | T |
| *Sideroxylon betsimisarakum* Lecomte | 54.1 [2017] | Fruit | Reported as *Sideroxylum betsimisarakum.* | Sapotaceae | D | T |
| *Silene latifolia* Poir*.* | 399.8 [2018] | Shoot | From an area around an abandoned mine. | Caryophyllaceae | D | H |
| *Silene latifolia* Poir*.* | 52.6 [2019] | Shoot | From an area around an abandoned mine. | Caryophyllaceae | D | H |
| *Silene vulgaris* (Moench) Garcke | 90 [2020] | Seed |  | Caryophyllaceae | D | H |
| *Silybum marianum* (L.) Gaertner | 60 [2021] | Seed |  | Asteraceae | D | H |
| *Sinapis alba* L. | 16.0 [2022] | Seed |  | Brassicaceae | D | H |
| *Sinapis alba* L. | 72.00 [2023] | Seed | Reported as *Brassica alba.* | Brassicaceae | D | H |
| *Sinapis alba* L. | 99.8 [2024] | Seed |  | Brassicaceae | D | H |
| *Sinapis arvensis* L. | 70 [2025] | Seed |  | Brassicaceae | D | H |
| *Sinojackia xylocarpa* Hu | 334.8 [2026] | Leaf |  | Styracaceae | D | T |
| *Sinowilsonia henryi* Hemsl. | 330.6 [2027] | Leaf |  | Hamamelidaceae | D | T |
| *Smallanthus sonchifolius* (Poepp.) H.Rob. | 10.8 [2028] | Leaf | It seems that the values for leaf and stem (iron concentrations) are calculated on a dry basis, but this is not very clear from the source paper. | Asteraceae | D | H |
| *Smallanthus sonchifolius* (Poepp.) H.Rob. | 7.3 [2029] | Stem | It seems that the numbers for leaf and stem are calculated on a dry basis, but this is not very clear from the source paper. | Asteraceae | D | H |
| *Smallanthus sonchifolius* (Poepp.) H.Rob. | 9.7 [2030] | Tuber |  | Asteraceae | D | H |
| *Solanum americanum* Mill. | 307 [2031] | Leaf | From a paper mill contaminated area. | Solanaceae | D | Su |
| *Solanum americanum* Mill. | 36.76 [2032] | Leaf | Reported *Solanum nigrum* L. | Solanaceae | D | H |
| *Solanum americanum* Mill. | 427.6 [2033] | Root | From a paper mill contaminated area. | Solanaceae | D | Su |
| *Solanum americanum* Mill. | 180.9 [2034] | Stem | From a paper mill contaminated area. | Solanaceae | D | Su |
| *Solanum lycopersicum* L.*/Lycopersicon esculentum* Mill. | 196.1 [2035] | Fruit | See comment above for this species name. | Solanaceae | D | H |
| *Solanum lycopersicum* L.*/Lycopersicon esculentum* Mill. | 37.0 [2036] | Fruit | Described as “tomato”. *Solanum lycopersicum* and *Lycopersicum esculentum* are most often considered synonyms (e.g., http://www.uniprot.org/taxonomy/4081), but The Plant List treats them as distinct species (http://www.theplantlist.org/tpl1.1/record/tro-29602513 and http://www.theplantlist.org/tpl1.1/record/tro-29605838). | Solanaceae | D | H |
| Solanum melongena L. | 785.4 [2037] | Aerial parts | Destalked. | Solanaceae | D | H |
| *Solanum tuberosum* L. | 69.2 [2038] | Stem | Described as “potato” | Solanaceae | D | H |
| *Solanum tuberosum* L. | 1006 [2039] | Tuber |  | Solanaceae | D | H |
| *Solanum tuberosum* L. | 39.0 [2040] | Tuber | Described as “Irish potato tubers” | Solanaceae | D | H |
| *Solanum tuberosum* L. | 43 [2041] | Tubers |  | Solanaceae | D | H |
| *Solidago virgaurea* L. | 23 [2042] | Leaf |  | Asteraceae | D | H |
| *Solidago virgaurea* L. | 9 [2043] | Leaf |  | Asteraceae | D | H |
| *Sonchus arvensis* L. | 35.6 [2044] | Leaf | From a paper mill contaminated area. | Asteraceae | D | H |
| *Sonchus arvensis* L. | 64.5 [2045] | Root | From a paper mill contaminated area. | Asteraceae | D | H |
| *Sonchus arvensis* L. | 56.7 [2046] | Stem | From a paper mill contaminated area. | Asteraceae | D | H |
| *Sonchus asper* (L.) Hill | 883 [2047] | Leaf |  | Asteraceae | D | H |
| *Sonchus oleraceus* (L.) L. | 193 [2048] | Leaf |  | Asteraceae | D | H |
| *Sonchus oleraceus* (L.) L. | 45.6 [2049] | Leaf |  | Asteraceae | D | H |
| *Sophora japonica* L. | 357.7 [2050] | Leaf | From Beijing Botanica Garden (control). | Fabaceae | D | T |
| *Sophora japonica* L. | 630.7 [2051] | Leaf | From hills near the Beijing Steel Factory. | Fabaceae | D | T |
| *Sorbus* *aucuparia* (L.) | 400 [2052] | Leaf | Median = 100. | Rosaceae | D | T |
| *Sorbus* *aucuparia* (L.) | 60 [2053] | Leaf | Median = 100. | Rosaceae | D | T |
| *Sorbus aucuparia* L. | 110 [2054] | Leaf |  | Rosaceae | D | T |
| *Sorbus aucuparia* L. | 130 [2055] | Leaf |  | Rosaceae | D | T |
| *Sorbus commixta* Hedl. | 162 [2056] | Leaf | Reported as *Sorbus japonica*. | Rosaceae | D | T |
| *Sorghum bicolor* (L.)Moench. | 3.8 [2057] | Seed |  | Poaceae | M | H |
| *Sorghum halepense* (L.) Pers | 2190 [2058] | Leaf |  | Poaceae | M | H |
| *Sparganium* *erectum* L. | 240 [2059] | Leaf | Aquatic | Typhaceae | M | H |
| *Sparganium eurycarpum* Engelm. | 2056 [2060] | Aerial parts | Aquatic | Typhaceae | M | H |
| *Sparganium fluctuans* (Engelm.) B.L.Rob. | 342 [2061] | Aerial parts | Aquatic | Typhaceae | M | H |
| *Sparganium* *natans* L. | 5620 [2062] | Leaf | Acquatic Reported as *Sparganium minimum* Wallr. | Typhaceae | M | H |
| Spartina densiflora Brong. | 768 [2063] | Aerial parts |  | Poaceae | M | H |
| Spartina maritima (Curtis) Fernald | 568 [2064] | Aerial parts |  | Poaceae | M | H |
| *Spathodea campanulata* P. Beauv. | 51.6 [2065] | Fruit |  | Bignoniaceae | D | T |
| *Spathodea campanulata* P.Beauv. | 115.9 [2066] | Leaf | Young leaf. | Bignoniaceae | D | T |
| *Spermacoce articularis* L.f. | 766.6 [2067] | Aerial parts |  | Rubiaceae | D | H |
| *Sphagnum fimbriatum* Warnst. var. *laxifolium* | 660 [2068] | Aerial parts | Var. *laxifolium* is not included in The Plant List. | Sphagnaceae | B | H |
| *Sphagnum subsecundum* var. *auriculatum* (Schimp.) Schlieph. | 2600 [2069] | Aerial parts | Reported as *Sphagnum auriculatum* Schimp. var. *ovatum.* | Sphagnaceae | B | H |
| *Sphagnum* *plumulosum* Roll | 260 [2070] | Aerial parts | Moss from semiaquatic soil | Sphagnaceae | B | H |
| *Sphagnum* *plumulosum* Roll | 340 [2071] | Aerial parts | Moss from semiaquatic soil | Sphagnaceae | B | H |
| *Sphagnum* *teres* (Schimp.) Ångström | 880 [2072] | Aerial parts |  | Sphagnaceae | B | H |
| *Spinacia oleracea* L. | 156.2 | Leaf |  | Amaranthaceae | D | H |
| *Spinacia oleracea* L. | 235.7 [2074] | Leaf |  | Amaranthaceae | D | H |
| *Spinacia oleracea* L. | 416.5 [2075] | Leaf |  | Amaranthaceae | D | H |
| *Spinacia oleracea* L. | 73.2 [2076] | Leaf |  | Amaranthaceae | D | H |
| *Spinacia oleracea* L. | 80 [2077] | Leaf |  | Amaranthaceae | D | H |
| *Spinacia oleracea* L. | 227.4 [2078] | Leaf |  | Amaranthaceae | D | H |
| *Spondias pinnata* (L. f.) Kurz | 42.5 [2079] | Fruit |  | Anacardiaceae | D | T |
| *Spondias purpurea* L. | 3.21 [2080] | Fruit |  | Anacardiaceae | D | T |
| *Sporobolus helvolus* (Trin.) T.Durand & Schinz | 90 [2081] | Aerial parts | These are approximations, as the authors expressed the values in grams/kg (including the iron in the group of “macro elements”) instead of the usual mg/kg or ppm. | Poaceae | M | H |
| *Stachys cretica* L. | 4.2 [2082] | Aerial parts | Reported as *Stachys cretica* L. subsp. *smyrnaea* Rech ﬁl. | Lamiaceae | D | H |
| Stangea rhizantha (A. Gray) Killip | 1480 [2083] | Root |  | Caprifoliaceae | D | H |
| Stenochlaena palustris (Burm. f.) Bedd. | 41.5 [2084] | Leaf | Reported as Stenochlaena palutris (Burm.) Bedd. | Blechnaceae | P | H |
| *Stewartia pseudocamellia* Maxim. | 164 [2085] | Leaf |  | Theaceae | D | T |
| *Stewartia sinensis* Rehder & E.H. Wilson | 297.7 [2086] | Leaf |  | Theaceae | D | S |
| *Sticherus nudus* (Moritz ex Reichard) Nakai | 223.0 [2087] | Aerial parts |  | Gleicheniaceae | P | H |
| *Stipa barbata* Desf. | 5910 [2088] | Whole plant | From a copper and iron mine area. | Poaceae | M | H |
| *Strombosia scheffleri* Engl. | 106.4 [2089] | Leaf | Young leaf | Olacaceae | D | T |
| *Strombosia scheffleri* Engl. | 594.4 [2090] | Leaf |  | Olacaceae | D | T |
| *Strombosia scheffleri* Engl. | 111.6 [2091] | Petiole |  | Olacaceae | D | T |
| *Strychnos innocua* Delile | 60 [2092] | Fruit |  | Loganiaceae | D | T |
| *Strychnos mitis* S.Moore | 13.8 [2093] | Fruit |  | Loganiaceae | D | T |
| *Strychnos mitis* S.Moore | 107.9 [2094] | Leaf | Young leaf | Loganiaceae | D | T |
| *Strychnos mitis* S.Moore | 94.8 [2095] | Leaf |  | Loganiaceae | D | T |
| *Strychnos spinosa* Lam. | 136 [2096] | Fruit |  | Loganiaceae | D | T |
| *Strychnos spinosa* Lam. | 43.9 [2097] | Fruit |  | Loganiaceae | D | T |
| *Stuckenia pectinata* (L.) Börner | 1171 [2098] | Aerial parts | Reported as *Potamogeton pectinatus* L.Aquatic. | Potamogetonaceae | M | H |
| *Styrax japonicus* Siebold & Zucc. | 118 [2099] | Leaf |  | Styracaceae | D | T |
| *Styrax shiraianus* Makino | 103 [2100] | Leaf | Reported as *Styrax shiraiana* [*Styrax shiraianus* Makino] | Styracaceae | D | T |
| *Suaeda vermiculata* Forssk. ex J.F.Gmel. | 722 [2101] | Aerial parts | Reported as *Suaeda fruticosa.* Succulent. | Amaranthaceae | D | S |
| *Symphonia globulifera* L.f. | 77.7 [2102] | Flower |  | Clusiaceae | D | T |
| *Symphonia louvelii* Jum. & H. Perrier | 28.6 [2103] | Flower |  | Clusiaceae | D | T |
| *Symphonia louvelii* Jum. & H. Perrier | 34.6 [2104] | Leaf |  | Clusiaceae | D | T |
| *Symplocos coreana* (H. Lév.) Ohwi | 202 [2105] | Leaf |  | Symplocaceae | D | S |
| *Syntrichia ruralis* (Hedw.) F. Weber & D. Mohr | 2120.4 [2106] | Leaf | Reported as *Tortula ruralis* (Hedw.) P. Gaertn., B. Mey. & Scherb.. Reported in mmol/kg, converted by us in mg/kg. | Pottiaceae | B | H |
| *Syzygium aromaticum* (L.) Merr. & L.M.Perry | 235 [2107] | Bud | Flower buds. | Myrtaceae | D | T |
| *Syzygium aromaticum* (L.) Merr. & L.M.Perry | 64.75 [2108] | Bud | Flower buds. | Myrtaceae | D | T |
| *Syzygium aromaticum* (L.) Merr. & L.M.Perry | 80 [2109] | Bud | Flower buds. | Myrtaceae | D | T |
| *Syzygium caryophyllatum* (L.) Alston | 99.275 [2110] | Leaf |  | Myrtaceae | D | T |
| *Syzygium cumini* (L.) Skeels | 2165 [2111] | Fruit |  | Myrtaceae | D | T |
| *Syzygium cumini* (L.) Skeels | 206 [2112] | Seed | Reported as *Eugenia jambolana* Lam. | Myrtaceae | D | T |
| Syzygium densiflorum Wall. ex Wight & Arn. | 93.829 [2113] | Leaf |  | Myrtaceae | D | T |
| *Syzygium emirnense* (Baker) Labat & Schatz | 39.0 [2114] | Fruit |  | Myrtaceae | D | T |
| *Syzygium emirnense* (Baker) Labat & Schatz | 42.1 [2115] | Leaf |  | Myrtaceae | D | T |
| *Syzygium guineense* (Willd.) DC. | 758 [2116] | Fruit |  | Myrtaceae | D | T |
| *Syzygium* *zeylanicum* (L.) DC. | 14 [2117] | Leaf | Reported as “leaves (or shoots)”. Sandy podzolic soil (pH 5.0). Reported as *Eugenia spicata* Lam. | Myrtaceae | D | T |
| Tabernaemontana siphilitica (L.f.) Leeuwenb. | 167.4 [2118] | Leaf | Old leaf. FeSO_4_ (50 microM) as iron source. Aerobic conditions in hydroponic culture. Reported as Tabernaemontana juruana (Markgr.) K.Schum. ex J.F.Macbr. | Apocynaceae | D | T |
| Tabernaemontana siphilitica (L.f.) Leeuwenb. | 195.3 [2119] | Leaf | Old leaf. FeSO_4_ (250 microM) as iron source. Hypoxic conditions in hydroponic culture. Reported as Tabernaemontana juruana (Markgr.) K.Schum. ex J.F.Macbr. | Apocynaceae | D | T |
| Tabernaemontana siphilitica (L.f.) Leeuwenb. | 228.8 [2120] | Leaf | Young leaf. FeSO_4_ (50 microM) as iron source. Aerobic conditions in hydroponic culture. Reported as Tabernaemontana juruana (Markgr.) K.Schum. ex J.F.Macbr. | Apocynaceae | D | T |
| Tabernaemontana siphilitica (L.f.) Leeuwenb. | 295.7 [2121] | Leaf | Young leaf. FeSO_4_ (250 microM) as iron source. Hypoxic conditions in hydroponic culture. Reported as Tabernaemontana juruana (Markgr.) K.Schum. ex J.F.Macbr. | Apocynaceae | D | T |
| Tabernaemontana siphilitica (L.f.) Leeuwenb. | 329.2 [2122] | Leaf | Old leaf. FeSO_4_ (500 microM) as iron source. Hypoxic conditions in hydroponic culture. Reported as Tabernaemontana juruana (Markgr.) K.Schum. ex J.F.Macbr. | Apocynaceae | D | T |
| Tabernaemontana siphilitica (L.f.) Leeuwenb. | 385.0 [2123] | Leaf | Young leaf. FeSO_4_ (500 microM) as iron source. Hypoxic conditions in hydroponic culture. Reported as Tabernaemontana juruana (Markgr.) K.Schum. ex J.F.Macbr. | Apocynaceae | D | T |
| Tabernaemontana siphilitica (L.f.) Leeuwenb. | 546.8 [2124] | Leaf | Old leaf. FeEDTA (40 microM) as iron source. Aerobic conditions in hydroponic culture. Reported as Tabernaemontana juruana (Markgr.) K.Schum. ex J.F.Macbr. | Apocynaceae | D | T |
| Tabernaemontana siphilitica (L.f.) Leeuwenb. | 569.2 [2125] | Leaf | Young leaf. FeEDTA (40 microM) as iron source. Aerobic conditions in hydroponic culture. Reported as Tabernaemontana juruana (Markgr.) K.Schum. ex J.F.Macbr. | Apocynaceae | D | T |
| Tabernaemontana siphilitica (L.f.) Leeuwenb. | 128652.5 [2126] | Root | FeSO_4_ (500 microM) as iron source. Hypoxic conditions in hydroponic culture. Reported as Tabernaemontana juruana (Markgr.) K.Schum. ex J.F.Macbr. | Apocynaceae | D | T |
| Tabernaemontana siphilitica (L.f.) Leeuwenb. | 22303.3 [2127] | Root | FeSO_4_ (50 microM) as iron source. Aerobic conditions in hydroponic culture. Reported as Tabernaemontana juruana (Markgr.) K.Schum. ex J.F.Macbr. | Apocynaceae | D | T |
| Tabernaemontana siphilitica (L.f.) Leeuwenb. | 67328.3 [2128] | Root | FeSO_4_ (250 microM) as iron source. Hypoxic conditions in hydroponic culture. Reported as Tabernaemontana juruana (Markgr.) K.Schum. ex J.F.Macbr. | Apocynaceae | D | T |
| Tabernaemontana siphilitica (L.f.) Leeuwenb. | 993.2 [2129] | Root | FeEDTA (40 microM) as iron source. Aerobic conditions in hydroponic culture. Reported as Tabernaemontana juruana (Markgr.) K.Schum. ex J.F.Macbr. | Apocynaceae | D | T |
| Tabernaemontana siphilitica (L.f.) Leeuwenb. | 1495.4 [2130] | Shoots | FeSO_4_ (50 microM) as iron source. Aerobic conditions in hydroponic culture. Reported as Tabernaemontana juruana (Markgr.) K.Schum. ex J.F.Macbr. | Apocynaceae | D | T |
| Tabernaemontana siphilitica (L.f.) Leeuwenb. | 228.8 [2131] | Shoots | FeEDTA (40 microM) as iron source. Aerobic conditions in hydroponic culture. Reported as Tabernaemontana juruana (Markgr.) K.Schum. ex J.F.Macbr. | Apocynaceae | D | T |
| Tabernaemontana siphilitica (L.f.) Leeuwenb. | 2572.4 [2132] | Shoots | FeSO_4_ (250 microM) as iron source. Hypoxic conditions in hydroponic culture. Reported as Tabernaemontana juruana (Markgr.) K.Schum. ex J.F.Macbr. | Apocynaceae | D | T |
| Tabernaemontana siphilitica (L.f.) Leeuwenb. | 7873.4 [2133] | Shoots | FeSO_4_ (500 microM) as iron source. Hypoxic conditions in hydroponic culture. Reported as Tabernaemontana juruana (Markgr.) K.Schum. ex J.F.Macbr. | Apocynaceae | D | T |
| *Taiwania cryptomerioides* Hayata | 339.1 [2134] | Leaf | Reported as *Taiwania ﬂousiana* Gaussen. | Cupressaceae | G | T |
| Talinum fruticosum (L.) Juss. | 0 [2135] | Aerial parts | Destalked. We used “0” for non-detected amounts. Reported as Talinum triangulare (Jacq.) Willd. | Talinaceae | D | H |
| *Talinum fruticosum* (L.) Juss. | 0.1 [2136] | Leaf | Reported on a fresh matter basis. Converted by us on dry matter basis. Reported as Talinum triangulare (Jacq.) Willd. | Talinaceae | D | H |
| *Tamarindus indica* L. | 759.0 [2137] | Seed |  | Fabaceae | D | T |
| *Tamarindus indica* L. | 68 [2138] | Fruit |  | Fabaceae | D | T |
| *Tamarindus indica* L. | 90.9 [2139] | Seed |  | Fabaceae | D | T |
| Tamarix gallica L. | 157 [2140] | Aerial parts |  | Tamaricaceae | D | S |
| *Tamarix kotschyi* Bunge | 250 [2141] | Aerial parts |  | Tamaricaceae | D | S |
| *Tapiscia sinensis* Oliv. | 337.2 [2142] | Leaf |  | Tapisciaceae | D | T |
| *Taraxacum campylodes* G.E.Haglund | 209 [2143] | Aerial parts | Reported as *Taraxacum officinale* (L.) Weber ex F.H.Wigg. | Asteraceae | D | H |
| *Taraxacum campylodes* G.E.Haglund | 6376 [2144] | Leaf | Reported as *Taraxacum officinale* (L.) Weber ex F.H.Wigg. 245- herbal part not stated | Asteraceae | D | H |
| *Taxus baccata* L. | 70 [2146] | Leaf |  | Taxaceae | G | T |
| *Taxus wallichiana* Zucc. | 250.8 [2147] | Leaf | Reported as *Taxus yunnanensis* W.C.Cheng & L.K.Fu. | Taxaceae | G | T |
| *Tecoma stans* (L.) Juss. ex Kunth | 24.5 [2148] | Whole plant | Reported as *Tecoma stans* (L.) Juss. ex Kunth | Bignoniaceae | D | T |
| *Telfairia occidentalis* Hook. f. | 10.1 [2149] | Leaf | Upper leaves. Control group. | Cucurbitaceae | D | H |
| *Telfairia occidentalis* Hook. f. | 10.7 [2150] | Leaf | Middle leaves. Control group. | Cucurbitaceae | D | H |
| *Telfairia occidentalis* Hook. f. | 11.0 [2151] | Leaf | Upper leaves. Nitrogen supplied. | Cucurbitaceae | D | H |
| *Telfairia occidentalis* Hook. f. | 12.7 [2152] | Leaf | Middle leaves. Nitrogen supplied. | Cucurbitaceae | D | H |
| *Telfairia occidentalis* Hook. f. | 15.50 [2153] | Leaf | Basal leaves. Control group. | Cucurbitaceae | D | H |
| *Telfairia occidentalis* Hook. f. | 17.2 [2154] | Leaf | Basal leaves. Nitrogen supplied. | Cucurbitaceae | D | H |
| *Telfairia occidentalis* Hook.f. | 4.0 [2155] | Leaf | Reported on a fresh matter basis. Converted by us on dry matter basis. Reported as *Teiferia occidentalis.* | Cucurbitaceae | D | H |
| *Terminalia chebula* Retz. | 0310 [2156] | Fruit |  | Combretaceae | D | T |
| *Terminalia ombrophila* H. Perrier | 126.6 [2157] | Fruit |  | Combretaceae | D | T |
| *Tetrapleura tetraptera* (Schum. & Thonn.) Taub. | 11.5 [2158] | Fruit |  | Fabaceae | D | T |
| *Thapsia garganica* L. | 100 [2159] | Seed |  | Apiaceae | D | H |
| *Thelypteris phegopteris* (L.) Sloss. ex Rydb. | 530 [2160] | Leaf |  | Thelypteridaceae | P | H |
| *Thuidium* *tamariscinum* (Hedw.) Schimp. | 1300 [2161] | Aerial parts |  | Thuidiaceae | B | H |
| *Thujopsis dolabrata* (L.f.) Siebold & Zucc. | 100 [2162] | Bark |  | Cupressaceae | G | T |
| *Thujopsis dolabrata* (L.f.) Siebold & Zucc. | 12 [2163] | Bark |  | Cupressaceae | G | T |
| *Thujopsis dolabrata* (L.f.) Siebold & Zucc. | 3.2 [2164] | Wood | . | Cupressaceae | G | T |
| *Thymbra spicata* L. | 111.86 [2165] | Aerial parts | Flower + leaf. | Lamiaceae | D | Su |
| Thymelaea villosa (L.) Endl. | 258 [2166] | Aerial parts |  | Thymeleaceae | D | S |
| *Thymus dahuricus* Serg. | 196 [2167] | Aerial parts |  | Lamiaceae | D | Su |
| Thymus mastichina (L.) L. | 323 [2168] | Aerial parts |  | Lamiaceae | D | S |
| *Thymus mastichina* (L.) L. | 229.2 [2169] | Shoot | From an area around an abandoned mine. | Lamiaceae | D | H |
| *Thymus mastichina* (L.) L. | 430.0 [2170] | Shoot | From an area around an abandoned mine. | Lamiaceae | D | H |
| *Thymus serpyllum* L. | 84.4 [2171] | Flower |  | Lamiaceae | D | Su |
| *Thymus vulgaris* L. | 11684 [2172] | Aerial parts |  | Lamiaceae | D | Su |
| *Thymus vulgaris* L. | 27095 [2173] | Aerial parts |  | Lamiaceae | D | Su |
| *Thymus vulgaris* L. | 123 [2174] | Leaf |  | Lamiaceae | D | H |
| *Tilia × europaea* L. | 55.8 [2175] | Leaf | Reported as *Tilia vulgaris.* | Malvaceae | D | T |
| *Tilia × europaea* L. | 8.4 [2176] | Leaf | Reported as *Tilia vulgaris.* | Malvaceae | D | T |
| *Tilia cordata* Mill. | 399.47 [2177] | Aerial parts |  | Malvaceae | D | T |
| *Tilia japonica* (Miq.) Simonk. | 16 [2178] | Bark |  | Malvaceae | D | T |
| *Tilia japonica* (Miq.) Simonk. | 27 [2179] | Bark |  | Malvaceae | D | T |
| *Tilia japonica* (Miq.) Simonk. | 0 [2180] | Wood | Under LOQ. | Malvaceae | D | T |
| *Tina urschii* (Capuron) Callm. & Buerki | 130.4 [2181] | Leaf | Reported as *Tinopsis urschii* Capuron*.* | Sapindaceae | D | T |
| *Tinospora sinensis* (Lour.) Merr. | 1300 [2182] | Stem | Reported as *Tinospora cordifolia* (Willd.) Miers | Menispermaceae | D | V |
| *Tinospora sinensis* (Lour.) Merr. | 471.2 [2183] | Stems | Reported as *Tinospora cordifolia* (Willd.) Miers*.* | Menispermaceae | D | V |
| Tolpis barbata (L.) Gaertn. | 1258 [2184] | Aerial parts |  | Asteraceae | D | H |
| Tolpis umbellata Bertol. | 298 [2185] | Aerial parts |  | Asteraceae | D | H |
| *Toona ciliata* M.Roem. | 253.4 [2186] | Leaf |  | Meliaceae | D | T |
| Torilis arvensis (Huds.) Link | 153 [2187] | Aerial parts | Reported as Torilis arvensis Link. | Apiaceae | D | H |
| *Toxicodendron trichocarpum* (Miq.) Kuntze | 125 [2188] | Leaf | Reported as *Rhus trichocarpa* Miq. | Anacardiaceae | D | T |
| *Trachyspermum ammi* (L.) Sprague | 194.92 [2189] | Fruit | Reported as *Carum copticum.* | Apiaceae | D | H |
| *Trachyspermum ammi* (L.) Sprague | 2792 [2190] | Seed |  | Apiaceae | D | H |
| *Tragopogon hybridus* R.Lesson. | 30 [2191] | Seed |  | Asteraceae | D | H |
| *Trapa natans* var. *bispinosa* (Roxb.) Makino | 3300 [2192] | Fruit | Reported as *Trapa bispinosa* Roxb*.* Aquatic. | Lythraceae | D | H |
| *Trema orientalis* (L.) Blume | 310.5 [2193] | Fruit |  | Cannabaceae | D | T |
| *Tribulus terrestris* L. | 44.09 [2194] | Leaf |  | Zygophyllaceae | D | H |
| *Trichilia emetica* Vahl. | 43 [2195] | Fruit |  | Meliaceae | D | T |
| *Trichophorum* *cespitosum* (L.) Hartm. | 210 [2196] | Leaf | From raised-bog peats. Reported as *Scirpus cespitosus* L. | Cyperaceae | M | H |
| *Trichostomum tortuosum* (Hedw.) Dixon | 4000 [2197] | Aerial parts |  |  | B | H |
| *Trifolium alexandrinum* L. | 184 [2198] | Aerial parts |  | Fabaceae | D | H |
| *Trifolium brachycalycinum* (Katzn. & Morley) Katzn. | 146.3 [2199] | Aerial parts | Reported as *Trifolium subterraneum* L. *brachycalycinum* | Fabaceae | D | H |
| Trifolium campestre Schreb. | 108 [2200] | Aerial parts |  | Fabaceae | D | H |
| *Trifolium glomeratum* L. | 136.7 [2201] | Aerial parts |  | Fabaceae | D | H |
| *Trifolium glomeratum* L. | 340.0 [2202] | Aerial parts |  | Fabaceae | D | H |
| *Trifolium pratense* L. | 134 [2203] | Aerial parts |  | Fabaceae | D | H |
| *Trifolium pratense* L. | 535 [2204] | Aerial parts |  | Fabaceae | D | H |
| *Trifolium pratense* L. | 1196 [2205] | Leaf |  | Fabaceae | D | H |
| *Trifolium repens* L. | 201 [2206] | Aerial parts |  | Fabaceae | D | H |
| *Trifolium subterraneum* L. | 171.1 [2207] | Aerial parts | Reported as *Trifolium subterraneum* L. *subterraneum* | Fabaceae | D | H |
| *Trifolium tomentosum* L. | 156.7 [2208] | Aerial parts |  | Fabaceae | D | H |
| *Trifolium tomentosum* L. | 256.7 [2209] | Aerial parts |  | Fabaceae | D | H |
| *Trigonella foenum-graecum L.* | 215.4 [2210] | Leaf |  | Fabaceae | D | H |
| *Trilepisium madagascariense* DC | 74.2 [2211] | Leaf | Reported as *Bosqueia phoberos* Baill. | Moraceae | D | T |
| *Trilepisium madagascariense* DC. | 133.1 [2212] | Leaf | Reported as *Bosqueia phoberos* Baill. Young leaf. | Moraceae | D | T |
| *Triticum aestivum* L. | 4.0 [2213] | Fruit | Caryopses (Fruit+seed) | Poaceae | M | H |
| *Triticum aestivum* L. | 812.0 [2214] | Stem |  | Poaceae | M | H |
| *Trophis montana* (Leandri) C.C. Berg | 64.1 [2215] | Fruit | Reported as *Maillardia orientalis* Leandri*.* | Moraceae | D | T |
| *Trophis montana* (Leandri) C.C. Berg | 73.2 [2216] | Fruit | Reported as *Maillardia orientalis* Leandri*.* | Moraceae | D | T |
| *Tsuga sieboldii* Carrière | 120 [2217] | Bark |  | Pinaceae | G | T |
| *Tsuga sieboldii* Carrière | 25 [2218] | Bark |  | Pinaceae | G | T |
| *Tsuga sieboldii* Carrière | 185 [2219] | Leaf |  | Pinaceae | G | T |
| *Tsuga sieboldii* Carrière | 8.2 [2220] | Wood |  | Pinaceae | G | T |
| *Tsuga heterophylla* (Raf.) Sarg. | 0 [2221] | Wood | Under LOQ. Reported as *Tsuga heterophylla* Sarg | Pinaceae | G | T |
| *Typha latifolia* L. | 320 [2222] | Leaf | Aquatic | Typhaceae | M | H |
| *Typha angustifolia* L. | 1647 [2223] | Aerial parts | Aquatic | Typhaceae | M | H |
| *Typha angustifolia* L. | 190 [2224] | Leaf | Aquatic | Typhaceae | M | H |
| Typha domingensis Pers. | 220 [2225] | Aerial parts | Reported as Typha domingensis (Pers.) Steud. | Typhaceae | M | H |
| *Typha latifolia* L. | 110 [2226] | Leaf | Values are only approximations, as the authors provided graphs but not labels with the accurate values. From a site intensely polluted with heavy metals, including iron. Aquatic. | Typhaceae | M | H |
| *Typha latifolia* L. | 390 [2227] | Leaf | Values are only approximations, as the authors provided graphs but not labels with the accurate values. From a site intensely polluted with heavy metals, including iron. Aquatic. | Typhaceae | M | H |
| *Typha latifolia* L. | 330 [2228] | Root | Values are only approximations, as the authors provided graphs but not labels with the accurate values. From a site intensely polluted with heavy metals, including iron. Aquatic. | Typhaceae | M | H |
| *Typha latifolia* L. | 520 [2229] | Root | Values are only approximations, as the authors provided graphs but not labels with the accurate values. From a site intensely polluted with heavy metals, including iron. Aquatic. | Typhaceae | M | H |
| *Typha latifolia* L. | 160 [2230] | Stem | Values are only approximations, as the authors provided graphs but not labels with the accurate values. From a site intensely polluted with heavy metals, including iron. Aquatic. | Typhaceae | M | H |
| *Typha latifolia* L. | 350 [2231] | Stem | Values are only approximations, as the authors provided graphs but not labels with the accurate values. From a site intensely polluted with heavy metals, including iron. Aquatic. | Typhaceae | M | H |
| *Uapaca kirkiana* Müll.Arg. | 431 [2232] | Fruit |  | Phyllanthaceae | D | T |
| *Uapaca louvelii* Denis | 676.9 [2233] | Fruit |  | Phyllanthaceae | D | T |
| Ulex parviflorus subsp. eriocladus (C.Vicioso) D.A.Webb | 251 [2234] | Aerial parts | Reported as Ulex eriocladus C. Vicioso | Fabaceae | D | S |
| *Ulmus chenmoui* W.C. Cheng | 358.7 [2235] | Leaf |  | Ulmaceae | D | T |
| *Ulmus elongata* L.K. Fu & C.S. Ding | 274.5 [2236] | Leaf |  | Ulmaceae | D | T |
| *Ulmus gaussenii* W.C. Cheng | 284.9 [2237] | Leaf |  | Ulmaceae | D | T |
| *Ulmus pumila* L. | 262.4 [2238] | Leaf | From Beijing Botanica Garden (control). | Ulmaceae | D | T |
| *Ulmus pumila* L. | 530.5 [2239] | Leaf | From hills near the Beijing Steel Factory. | Ulmaceae | D | T |
| *Urena lobata* L. | 48.54 [2240] | Leaf | From unpolluted soil. | Malvaceae | D | S |
| *Urena lobata* L. | 73.00 [2241] | Leaf | From polluted (crude-oil contaminated) soil. | Malvaceae | D | S |
| *Urtica dioica* L. | 13100 [2242] | Aerial parts | Actually „new shoot/leaves”. | Urticaceae | D | H |
| *Urtica dioica* L. | 14.4 | Leaf | 133mg/kg reported by Stef DS *et al.* 2010 and 3456 mg/kg reported by Tokalioglu S, 2012, but herbal parts not stated. | Urticaceae | D | H |
| *Urtica dioica* L. | 22.1 | Leaf |  | Urticaceae | D | H |
| *Urtica dioica* L. | 353.4 [2245] | Leaf |  | Urticaceae | D | H |
| *Urtica dioica* L. | 13.0 [2246] | Whole plant |  | Urticaceae | D | H |
| *Uvaria acuminata* Oliv. | 28.8 [2247] | Fruit |  | Annonaceae | Mag | T |
| *Uvariopsis congensis* Robyns & Ghesq. | 79.8 [2248] | Fruit |  | Annonaceae | Mag | T |
| *Uvariopsis congensis* Robyns & Ghesq*.* | 24.0 [2249] | Leaf | Young leaf. | Annonaceae | Mag | T |
| *Uvariopsis congensis* Robyns & Ghesq. | 90.2 [2250] | Leaf |  | Annonaceae | Mag | T |
| *Vaccaria hispanica* (Mill.) Rauschert | 90 [2251] | Seed |  | Caryophyllaceae | D | H |
| *Vaccinium myrtillus* L. | 100 [2252] | Leaf |  | Ericaceae | D | S |
| *Vaccinium myrtillus* L. | 47 [2253] | Leaf | Mean = 74. Pollution-free area. | Ericaceae | D | S |
| *Vaccinium myrtillus* L. | 97 [2254] | Leaf | Mean = 74. Pollution-free area. | Ericaceae | D | S |
| *Vaccinium myrtillus* L. | 37 [2255] | Stem | Mean = 62. Pollution-free area. | Ericaceae | D | S |
| *Vaccinium myrtillus* L. | 73 [2256] | Stem | Mean = 62. Pollution-free area. | Ericaceae | D | S |
| *Vaccinium myrtillus* L. | 55 [2257] | Whole plant | Mean = 74. Pollution-free area. | Ericaceae | D | S |
| *Vaccinium myrtillus* L. | 93 [2258] | Whole plant | Mean = 74. Pollution-free area. | Ericaceae | D | S |
| *Vaccinium oldhamii* Miq. | 109 [2259] | Leaf |  | Ericaceae | D | S |
| *Vaccinium ovatum* Pursh. | 20 [2260] | Fruit |  | Ericaceae | D | S |
| *Vaccinium parvifolium* Sm. | 40 [2261] | Fruit |  | Ericaceae | D | S |
| *Vaccinium smallii* A.Gray | 150 [2262] | Leaf | Reported as *Vaccinium smallii* var. *glabrum*. | Ericaceae | D | S |
| *Vaccinium vitis-idaea* L. | 210.7 [2263] | Leaf |  | Ericaceae | D | S |
| *Vallisneria spiralis* L. | 141 [2264] | Leaf | Aquatic. | Hydrocharitaceae | M | H |
| *Vallissneria americana* Michx. | 323 [2265] | Aerial parts | Aquatic | Hydrocharitaceae | M | H |
| *Vangueria apiculata* K.Schum. | 97.9 [2266] | Fruit |  | Rubiaceae | D | T |
| *Vangueria infausta* Burch. | 283 [2267] | Fruit |  | Rubiaceae | D | T |
| *Vepris nobilis* (Delile)Mziray | 76.9 [2268] | Leaf | Reported as *Teclea nobilis* Delile | Rutaceae | D | T |
| *Vepris nobilis* (Delile) Mziray | 79.3 [2269] | Leaf | Reported as *Teclea nobilis* Delile. Young leaf. | Rutaceae | D | T |
| *Verbascum speciosum* Schrad | 15390 [2270] | Whole plant | From a copper and iron mine area. | Scrophulariaceae | D | H |
| *Vernonia amygdalina* Delile | 2.3 [2271] | Leaf | Reported on a fresh matter basis. Converted by us on dry matter basis. Reported as *Vernomia anydalira* (which is most likely an erroneous rendition for *Vernonia amygdalina* Delile)*.* | Asteraceae | D | S |
| *Vernonia amygdalina* Delile | 31.5 [2272] | Leaf |  | Asteraceae | D | T |
| *Veronica arvensis* L. | 262.3 [2273] | Leaf | Reported in mmol/kg, converted by us in mg/kg. | Plantaginaceae | D | H |
| *Viburnum dilatatum* Thunb. | 228 [2274] | Leaf |  | Adoxaceae | D | S |
| *Viburnum erosum* Thunb. | 205 [2275] | Leaf |  | Adoxaceae | D | S |
| *Viburnum phlebotrichum* Siebold & Zucc. | 112 [2276] | Leaf |  | Adoxaceae | D | S |
| *Viburnum tinus* L. | 333 [2277] | Aerial parts |  | Adoxaceae | D | S |
| *Viburnum wrightii* Miq. | 128 [2278] | Leaf |  | Adoxaceae | D | S |
| *Vicia benghalesis* L. | 50 [2279] | Seed |  | Fabaceae | D | H |
| *Vicia cracca* L. | 130 [2280] | Aerial parts |  | Fabaceae | D | H |
| *Vicia cracca* L. | 175 [2281] | Aerial parts |  | Fabaceae | D | H |
| *Vicia cracca* L. | 2.6 [2282] | Aerial parts |  | Fabaceae | D | H |
| *Vicia lutea* L. | 30 [2283] | Seed |  | Fabaceae | D | H |
| *Vicia sativa* L. | 60 [2284] | Seed |  | Fabaceae | D | H |
| *Vigna radiata* (L.) R.Wilczek | 34.5 [2285] | Seed |  | Fabaceae | D | H |
| *Vigna radiata* (L.) R.Wilczek | 97 [2286] | Seed | Reported as *Phaseolus aureus.* | Fabaceae | D | H |
| *Vigna radiata* (L.) R.Wilczek | 10.4 [2287] | Stem | Reported as *Phaseolus aureus.* | Fabaceae | D | H |
| *Vigna unguiculata* (L.) Walp. | 300 [2288] | Aerial parts | Reported as *Vigna sinensis.* | Fabaceae | D | H |
| Vigna unguiculata (L.) Walp. | 458.0 [2289] | Aerial parts | Destalked. | Fabaceae | D | H |
| *Vigna unguiculata* L. | 3.9 [2290] | Leaf |  | Fabaceae | D | H |
| *Viola tricolor* subsp. *curtisii* (E.Forst.) Syme | 613.8 [2291] | Leaf | Reported as *Viola curtisii* E. Forst*.* Reported in mmol/kg, converted by us in mg/kg. | Violaceae | D | H |
| *Vitellaria paradoxa* C.F.Gaertn. | 52.0 [2292] | Seed |  | Sapotaceae | D | T |
| *Vitex agnus-castus* L. | 93.7 [2293] | Seed |  | Lamiaceae | D | S |
| *Vitex doniana* Sweet | 93 [2294] | Fruit |  | Lamiaceae | D | T |
| *Vitex negundo* L. | 203.1 [2295] | Leaf | From Beijing Botanica Garden (control). | Lamiaceae | D | T |
| *Vitex negundo* L. | 353.0 [2296] | Leaf | From hills near the Beijing Steel Factory. | Lamiaceae | D | T |
| *Vitis vinifera* L. | 134 [2297] | Fruit |  | Vitaceae | D | V |
| *Vitis vinifera* L. | 77.5 [2298] | Fruit |  | Vitaceae | D | V |
| *Wagneriopteris japonica* (Baker) Á. Löve & D. Löve | 229 [2299] | Leaf | Reportes as *Lastrea japonica* (Baker) Copel. | Tectariaceae | P | H |
| *Wikstroemia chamaedaphne* (Bunge) Meisn. | 346.3 [2300]- | Leaf | From Beijing Botanica Garden (control). | Thymelaeaceae | D | S |
| *Wikstroemia chamaedaphne* (Bunge) Meisn. | 391.8 [2301] | Leaf | From hills near the Beijing Steel Factory. | Thymelaeaceae | D | S |
| *Withania somnifera* (L.) Dunal | 3750.2 [2302] | Leaf |  | Solanaceae | D | Su |
| *Withania somnifera* (L.) Dunal | 15777 [2303] | Roots |  | Solanaceae | D | Su |
| *Withania somnifera* (L.) Dunal | 2380 [2304] | Seed |  | Solanaceae | D | Su |
| *Withania somnifera* (L.) Dunal | 9417.7 [2305] | Shoot |  | Solanaceae | D | Su |
| *Withania coagulans* (Stocks) Dunal | 9293 [2306] | Seed | Reported as *Wathania coagulans.* | Solanaceae | D | S |
| *Xanthosoma* *sagittifolium* (L.) Schott | 291.9 [2307] | Spathe | Reported as *Xanthosoma mafaffa* Schott | Araceae | M | H |
| *Ximenia americana* L. | 33.6 [2308] | Fruit |  | Olacaceae | D | T |
| *Ximenia caffra* Sond. | 366 [2309] | Fruit |  | Olacaceae | D | T |
| *Xylopia aethiopica* (Dunal) A.Rich. | 180 [2310] | Fruit |  | Annonaceae | Mag | T |
| *Xyris complanata* R. Br. | 91 [2311] | Leaf | Reported as “leaves (or shoots)”. Sandy podzolic soil (pH 5.0) | Xyridaceae | M | H |
| Yushania alpina (K.Schum.) W.C.Lin | 147.0 [2312] | Shoot | Reported as Arundinaria alpina K.Schum. | Poaceae | M | H |
| Yushania alpina (K.Schum.) W.C.Lin | 41.67 [2313] | Shoot | Reported as Arundinaria alpina K.Schum. | Poaceae | M | H |
| Yushania alpina (K.Schum.) W.C.Lin | 72.7 [2314] | Shoot | Reported as Arundinaria alpina K.Schum. | Poaceae | M | H |
| *Zanthoxylum rhetsa* DC. | 540 [2315] | Fruit | Reported as *Zanthoxylum rhetusa* Wall. | Rutaceae | D | T |
| *Zea mays* L. | 616.6 [2316] | Aerial parts |  | Poaceae | M | H |
| *Zea mays* L. | 2.8 [2317] | Seed | Probably caryopse | Poaceae | M | H |
| *Zea mays* L. | 59 [2318] | Seed | Probably caryopse | Poaceae | M | H |
| *Zea mays* L. | 71 [2319] | Shoot | Young plants—10 days. Grown in solutions with Cd added (0.10 mg/L). Cultivar BR 473 | Poaceae | M | H |
| *Zea mays* L. | 81 [2320] | Shoot | Young plants—10 days. Grown in solutions with no Cd added. Cultivar BR 473 | Poaceae | M | H |
| *Zea mays* L. | 86 [2321] | Shoot | Young plants—10 days. Grown in solutions with added Cd (0.05 mg/L). Cultivar BR 473 | Poaceae | M | H |
| *Zea mays* L. | 86 [2322] | Shoot | Young plants—10 days. Grown in solutions with Cd added (1.0 mg/L). Cultivar CMS 54 | Poaceae | M | H |
| *Zea mays* L. | 89 [2323] | Shoot | Young plants—10 days. Grown in solutions with Cd added (0.50 mg/L). Cultivar BR 473 | Poaceae | M | H |
| *Zea mays* L. | 91 [2324] | Shoot | Young plants—10 days. Grown in solutions with Cd added (1.0 mg/L). Cultivar BR 473 | Poaceae | M | H |
| *Zea mays* L. | 91 [2325] | Shoot | Young plants—10 days. Grown in solutions with Cd added (1.0 mg/L). Cultivar BR 473 | Poaceae | M | H |
| *Zea mays* L. | 91 [2326] | Shoot | Young plants—10 days. Grown in solutions with Cd added (0.1 mg/L). Cultivar CMS 54 | Poaceae | M | H |
| *Zea mays* L. | 92 [2327] | Shoot | Young plants—10 days. Grown in solutions with Cd added (0.05 mg/L). Cultivar CMS 54 | Poaceae | M | H |
| *Zea mays* L. | 92 [2328] | Shoot | Young plants—10 days. Grown in solutions with Cd added (0.5 mg/L). Cultivar CMS 54 | Poaceae | M | H |
| *Zea mays* L. | 93 [2329] | Shoot | Young plants—10 days. Grown in solutions with no Cd added. Cultivar CMS 54. | Poaceae | M | H |
| *Zelkova schneideriana* Hand.-Mazz. | 273.1 [2330] | Leaf |  | Ulmaceae | D | T |
| *Zelkova serrata* (Thunb.) Makino | 29 [2331] | Bark |  | Ulmaceae | D | T |
| *Zelkova serrata* (Thunb.) Makino | 9 [2332] | Bark |  | Ulmaceae | D | T |
| *Zelkova serrata* (Thunb.) Makino | 0 [2333] | Wood | Under LOQ. | Ulmaceae | D | T |
| *Zingiber officinale* Roscoe | 345 [2334] | Root |  | Zingiberaceae | M | H |
| *Zingiber officinale* Roscoe | 92.5 | Root |  | Zingiberaceae | M | H |
| *Zingiber officinale* Roscoe | 2475 [2336] | Roots |  | Zingiberaceae | M | H |
| *Zizania aquatica* | 2112 [2337] | Aerial parts | Aquatic | Poaceae | M | H |
| *Ziziphora clinopodioides* Lam. | 1965 [2338] | Whole plant | From a copper and iron mine area. | Lamiaceae | D | H |
| *Ziziphus jujuba* Mill. | 21 [2339] | Fruit |  | Rhamnaceae | D | T |
| *Ziziphus jujuba* Mill. | 3.5 [2340] | Fruit |  | Rhamnaceae | D | T |
| *Ziziphus jujuba* Mill. | 115 [2341] | Leaf |  | Rhamnaceae | D | T |
| *Ziziphus jujuba* Mill. | 768 [2342] | Leaf |  | Rhamnaceae | D | T |
| *Ziziphus mauritiana* Lam. | 178.5 [2343] | Fruit |  | Rhamnaceae | D | T |
| *Ziziphus mauritiana* Lam. | 21 [2344] | Fruit |  | Rhamnaceae | D | T |
| *Ziziphus mauritiana* Lam. | 6 [2345] | Fruit |  | Rhamnaceae | D | T |
| *Ziziphus spina-christi* (L.) Desf. | 72 [2346] | Leaf | Reported as *Ziziphus spina-christi* (L.) Willd. | Rhamnaceae | D | T |
| *Ziziphus vulgaris* Mill. | 384 [2347] | Fruit |  | Rhamnaceae | D | T |
| *Ziziphus jujube var. spinosa* (Bunge) Hu ex H.F.Chow | 219.6 [2348] | Leaf | From Beijing Botanica Garden (control). | Rhamnaceae | D | S |
| *Ziziphus jujuba var. spinosa* (Bunge) Hu ex H.F.Chow | 250.7 [2349] | Leaf | From hills near the Beijing Steel Factory. | Rhamnaceae | D | S |

Plant names used by various primary sources but treated as synonyms by The Plant List have been replaced by the currently accepted name in The Plant List and the name used in the original publication has been mentioned in a comment; in many publications the binary name has been limited to the genus and species, with no mentioning of the author name, but we have generally included the authors for the name where a single author is known. For the few cases where more than one author variant is known for a certain name (e.g., for *Alchemilla vulgaris*: *A. vulgaris* auct., *A. vulgaris* Willd., *A. vulgaris* Wight, *A. vulgaris* S.E. Fröhner, *A. vulgaris* Buser), no author name has been added by us in the comment to the binary name reported in the source publication for iron contents. Relatively often the names used in publications contained small errors (one letter lacking or a vowel erroneously replaced by a different vowel etc); most such errors have been silently corrected when included in the table. Where the change seemed more substantial, for the accuracy of reporting we mentioned the form under which the name was published (e.g., *Ceiba patendra* for what is most likely *Ceiba pentandra* (L.) Gaertn. or *Dereium microcarpum* for what should have been *Detarium microcarpum* Guill. & Perr.). Pseudofruits/composed fruits (e.g., *Rosa canina* L., where the receptacle becomes part of the “fruit”) are treated simply as fruits for analysis purposes. The number of digits for iron contents have varied (between 0 and 3) in the various source publications; we limited their number to one. If only point estimates were reported, we included the point estimate. If more than two iron values were available in a paper for a specific organ/part of a defined species (e.g., from different accessions), only minimal and maximal values were included. When different variables were considered by the authors of a specific study (e.g., species from two different habitats, a typical and an atypical one, needle age classes in case of *Abies alba* *etc.*), values for both such variables were included in the table (see e.g., *Dicranum majus* Turner). In certain papers (e.g., [2350] only graphical representations have been provided for the iron contents, but accurate figures were not reported.; inferring values from the graphics would be affected by errors and we only used such graphics for two species for which otherwise no comparative values for three different herbal parts (organs) would have been available (*Schoenoplectus americanus* (Pers.) Volkart. and *Typha latifolia* L. [2351]). Herbaceous vines were treated as herbaceous species (H) and not as (woody) vines (V).

References

1. Zoro, A.F.; Zoue, L.T.; Kra, S.A.K.; Yepie, A.E.; Niamke, S.L. An Overview of Nutritive Potential of Leafy Vegetables Consumed in Western Côte d’Ivoire. *Pak. J. Nutr.* **2013**, *12*, 949–956.
2. Osaki, M.; Watanabe, T.; Ishizawa, T.; Nilnond, C.; Nuyim, T.; Sittibush, C.; Tadano, T. Nutritional characteristics in leaves of native plants grown in acid sulfate, peat, sandy podzolic, and saline soils distributed in Peninsular Thailand. *Plant. Soil.* **1998**, *201*, 175–182.
3. Szymura, T.H. Concentration of elements in silver ﬁr (*Abies alba* Mill) needles as a function of needles’ age. *Trees* **2009**, *23*, 211–217.
4. Szymura, T.H. Concentration of elements in silver ﬁr (*Abies alba* Mill) needles as a function of needles’ age. *Trees* **2009**, *23*, 211–217.
5. Szymura, T.H. Concentration of elements in silver ﬁr (*Abies alba* Mill) needles as a function of needles’ age. *Trees* **2009**, *23*, 211–217.
6. Szymura, T.H. Concentration of elements in silver ﬁr (*Abies alba* Mill) needles as a function of needles’ age. *Trees* **2009**, *23*, 211–217.
7. Szymura, T.H. Concentration of elements in silver ﬁr (*Abies alba* Mill) needles as a function of needles’ age. *Trees* **2009**, *23*, 211–217.
8. Szymura, T.H. Concentration of elements in silver ﬁr (*Abies alba* Mill) needles as a function of needles’ age. *Trees* **2009**, *23*, 211–217.
9. Szymura, T.H. Concentration of elements in silver ﬁr (*Abies alba* Mill) needles as a function of needles’ age. *Trees* **2009**, *23*, 211–217.
10. Szymura, T.H. Concentration of elements in silver ﬁr (*Abies alba* Mill) needles as a function of needles’ age. *Trees* **2009**, *23*, 211–217.
11. Szymura, T.H. Concentration of elements in silver ﬁr (*Abies alba* Mill) needles as a function of needles’ age. *Trees* **2009**, *23*, 211–217.
12. Szymura, T.H. Concentration of elements in silver ﬁr (*Abies alba* Mill) needles as a function of needles’ age. *Trees* **2009**, *23*, 211–217.
13. Szymura, T.H. Concentration of elements in silver ﬁr (*Abies alba* Mill) needles as a function of needles’ age. *Trees* **2009**, *23*, 211–217.
14. Szymura, T.H. Concentration of elements in silver ﬁr (*Abies alba* Mill) needles as a function of needles’ age. *Trees* **2009**, *23*, 211–217.
15. Tsuchiya, Y.; Shimogaki, H.; Abe, H.; Kagawa, A. Inorganic elements in typical Japanese trees for woody biomass fuel. *Wood Sci.* **2010**, *56*, 53–63.
16. Tsuchiya, Y.; Shimogaki, H.; Abe, H.; Kagawa, A. Inorganic elements in typical Japanese trees for woody biomass fuel. *Wood Sci.* **2010**, *56*, 53–63.
17. Memon, A.R.; Itô, S.; Yatazawa, M. Absorption and accumulation of iron, manganese and copper in plants in the temperate forest of central Japan. Soil Sci. *Plant. Nutr.* **1979**, *15*, 611–620.
18. Tsuchiya, Y.; Shimogaki, H.; Abe, H.; Kagawa, A. Inorganic elements in typical Japanese trees for woody biomass fuel. *Wood Sci.* **2010**, *56*, 53–63.
19. Hafızoglu, H.; Sivrikaya, H.; Bacak, L. Inorganic constituents in barks of Abies bornmulleriana and Castanea sativa. *Holz. Werkstoff*. **2006**, *64*, 247–249.
20. Tsuchiya, Y.; Shimogaki, H.; Abe, H.; Kagawa, A. Inorganic elements in typical Japanese trees for woody biomass fuel. *Wood Sci.* **2010**, *56*, 53–63.
21. Tsuchiya, Y.; Shimogaki, H.; Abe, H.; Kagawa, A. Inorganic elements in typical Japanese trees for woody biomass fuel. *Wood Sci.* **2010**, *56*, 53–63.
22. Tsuchiya, Y.; Shimogaki, H.; Abe, H.; Kagawa, A. Inorganic elements in typical Japanese trees for woody biomass fuel. *Wood Sci.* **2010**, *56*, 53–63.
23. Kuria, S.G.; Tura, I.A.; Amboga, S.; Walaga, H.K. Assessment of status of minerals in forages preferred by camels (Camelus dromedarius) in the Arid North Eastern Kenya. *Res. Opin. Anim. Vet. Sci.* **2012**, *2*, 173–180.
24. Guerrero–Cervantes, M.; Ramírez, R.G.; González–Rodríguez, H.; Cerrillo–Soto, A.; Juárez–Réyes, A. Mineral content in range forages from north Mexico. *J. Appl. Anim. Res.* **2012**, *40*, 102–107.
25. Guerrero–Cervantes, M.; Ramírez, R.G.; González–Rodríguez, H.; Cerrillo-Soto, A.; Juárez–Réyes, A. Mineral content in range forages from north Mexico. *J. Appl. Anim. Res.* **2012**, *40*, 102–107.
26. Guerrero–Cervantes, M.; Ramírez, R.G.; González-Rodríguez, H.; Cerrillo-Soto, A.; Juárez–Réyes, A. Mineral content in range forages from north Mexico. *J. Appl. Anim. Res.* **2012**, *40*, 102–107.
27. Kuria, S.G.; Tura, I.A.; Amboga, S.; Walaga, H.K. Assessment of status of minerals in forages preferred by camels (*Camelus dromedarius*) in the Arid North Eastern Kenya. *Res. Opin. Anim. Vet. Sci.* **2012**, *2*, 173–180.
28. Malayeri, B.E.; Chehregani, A.; Yousefi, N.; Lorestani, B. Identification of the Hyper Acumulator Plants in Copper and Iron Mine in Iran. Pakistan *J. Biol. Sci.* **2008**, *11*, 490–492.
29. Rode, K.D.; Chapman, C.A.; Chapman, L.J.; McDowell, L.R. Mineral Resource Availability and Consumption by Colobus in Kibale National Park, Uganda. *Int. J. Primatol.*. **2003**, *24*, 541–573.
30. Rode, K.D.; Chapman, C.A.; Chapman, L.J.; McDowell, L.R. Mineral Resource Availability and Consumption by Colobus in Kibale National Park, Uganda. *Int. J. Primatol.* **2003**, *24*, 541–573.
31. Memon, A.R.; Itô, S.; Yatazawa, M. Absorption and accumulation of iron, manganese and copper in plants in the temperate forest of central Japan. *Soil Sci. Plant. Nutr.* **1979**, *15*, 611–620.
32. Wan, K.; Chen, F.; Tao, Y.; Chen, S. Nutrient Elements in Leaves of Rare and Endangered Species in Wuhan Botanical Garden, China. *J. Plant. Nutr.* **2009**, *32*, 1914–1940.
33. Memon, A.R.; Itô, S.; Yatazawa, M. Absorption and accumulation of iron, manganese and copper in plants in the temperate forest of central Japan. *Soil Sci. Plant. Nutr.* **1979**, *15*, 611–620.
34. Wan, K.; Chen, F.; Tao, Y.; Chen, S. Nutrient Elements in Leaves of Rare and Endangered Species in Wuhan Botanical Garden, China. *J. Plant. Nutr.* **2009**, *32*, 1914–1940.
35. Tsuchiya, Y.; Shimogaki, H.; Abe, H.; Kagawa, A. Inorganic elements in typical Japanese trees for woody biomass fuel. *Wood Sci.* **2010**, *56*, 53–63.
36. Tsuchiya, Y.; Shimogaki, H.; Abe, H.; Kagawa, A. Inorganic elements in typical Japanese trees for woody biomass fuel. *Wood Sci.* **2010**, *56*, 53–63.
37. Memon, A.R.; Itô, S.; Yatazawa, M. Absorption and accumulation of iron, manganese and copper in plants in the temperate forest of central Japan. *Soil Sci. Plant. Nutr.* **1979**, *15*, 611–620.
38. Memon AR, Itô S, Yatazawa M. Absorption and accumulation of iron, manganese and copper in plants in the temperate forest of central Japan. *Soil Sci. Plant. Nutr.* **1979**, *15*, 611–620.
39. Tsuchiya, Y.; Shimogaki, H.; Abe, H.; Kagawa, A. Inorganic elements in typical Japanese trees for woody biomass fuel. *Wood Sci*. **2010**, *56*, 53–63.
40. Memon, A.R.; Itô, S.; Yatazawa, M. Absorption and accumulation of iron, manganese and copper in plants in the temperate forest of central Japan. *Soil Sci. Plant. Nutr.* **1979**, *15*, 611–620.
41. Memon, A.R.; Itô, S.; Yatazawa, M. Absorption and accumulation of iron, manganese and copper in plants in the temperate forest of central Japan. *Soil Sci. Plant. Nutr.* **1979**, *15*, 611–620
42. Grzegorczyk, S.; Olszewska, M.; Alberski, J. Accumulation of copper, zinc, manganese and iron by selected species of grassland legumes and herbs. *J. Elem.* **2014**, *19*, 109–118.
43. Grzegorczyk, S.; Olszewska, M.; Alberski, J.Accumulation of copper, zinc, manganese and iron by selected species of grassland legumes and herbs. *J. Elem.* **2014**, *19*, 109–118.
44. Chizzola, R.; Michitsch, H.; Franz, C. Monitoring of metallic micronutrients and heavy metals in herbs, spices and medicinal plants from Austria. *Eur. Food Res. Technol*. **2003**, *216*, 407–411.
45. Kashin, V.K.Vitally Important Microelements in Transbaikalian Herbs. *Chem. Sustain. Dev.* **2009**, *17*, 371–381
46. Chizzola, R.; Michitsch, H.; Franz, C. Monitoring of metallic micronutrients and heavy metals in herbs, spices and medicinal plants from Austria. *Eur. Food Res. Technol.* **2003**, *216*, 407–411.
47. Razić, S.; Dogo, S.; Slavković, L. Investigation on bioavailability of some essential and toxic elements in medicinal herbs. *J. Nat. Med.* **2008**, *62*, 340–4.
48. Razić, S.; Dogo, S.; Slavković, L. Investigation on bioavailability of some essential and toxic elements in medicinal herbs. *J. Nat. Med.* **2008**, *62*, 340–344.
49. Divrikli, U.; Horzum, N.; Soylak, M.; Elci, L.Trace heavy metal contents of some spices and herbal plants from western Anatolia, Turkey. *Int. J. Food Sci. Technol.* **2006**, *41*, 712–716.
50. Divrikli, U.; Horzum, N.; Soylak, M.; Elci, L.Trace heavy metal contents of some spices and herbal plants from western Anatolia, Turkey. *Int. J. Food Sci. Technol.* **2006**, *41*, 712–716.
51. Ugulu, I.; Dogan, Y.; Baslar, S.; Varol, O. Biomonitoring of trace element accumulation in plants growing at Murat Mountain. *Int. J. Environ. Sci. Technol.* **2012**, *9*, 527–534.
52. Ugulu, I.; Dogan, Y.; Baslar, S.; Varol, O. Biomonitoring of trace element accumulation in plants growing at Murat Mountain. *Int. J. Environ. Sci. Technol.* **2012**, *9*, 527–534.
53. Saraf, A.; Samant, A. Evaluation of some minerals and trace elements in *Achyranthes aspera* Linn. *Int. J.* *Pharma Sci.* **2013**, *3*, 229–233
54. Vishwakarma, K.L.; Dubey, V. Nutritional analysis of indigenous wild edible herbs used in eastern Chhattisgarh, India. *Emir. J. Food Agric.* **2011**, *23*, 554–560.
55. Saraf, A.; Samant, A. Evaluation of some minerals and trace elements in *Achyranthes aspera* Linn. *Int. J.* *Pharma Sci.* **2013**, *3*, 229–233.
56. Saraf, A.; Samant, A. Evaluation of some minerals and trace elements in *Achyranthes aspera* Linn. *Int. J.* *Pharma Sci.* **2013**, *3*, 229–233.
57. Ozcan MM, Akbulut M. Estimation of minerals, nitrate and nitrite contents of medicinal and aromatic plants used as spices, condiments and herbal tea. *Food Chem.* **2008**, *106*, 852–858
58. Kumar, A.; Singh, R.P.; Singh, N.P. Analysis of macro and micro nutrients in some Indian medicinal herbs grown in Jaunpur (u.p.) soil. *Nat. Sci.* **2011**, *3*, 551–555.
59. Hussain, I.; Khattak, M.U.R.; Khan, F.A.; Rehman, I.U.; Khan, F.U.; Khan, F.U. Analysis of Heavy Metals in Selected Medicinal Plants from Dir, Swat and Peshawar Districts of Khyber Pakhtunkhwa. *J. Chem.
    Soc. Pak.* **2001**, *33*, 495–498.
60. Osaki, M.; Watanabe, T.; Ishizawa, T.; Nilnond, C.; Nuyim, T.; Sittibush, C.; Tadano, T. Nutritional characteristics in leaves of native plants grown in acid sulfate, peat, sandy podzolic, and saline soils distributed in Peninsular Thailand. *Plant. Soil* **1998**, *201*, 175–182.
61. Wan, K.; Chen, F.; Tao, Y.; Chen, S. Nutrient Elements in Leaves of Rare and Endangered Species in Wuhan Botanical Garden, China. *J. Plant. Nutr.* **2009**, *32*, 1914–1940.
62. Lockett, C.T.; Calvert, C.C.; Grivetti, L.E. Energy and micronutrient composition of dietary and medicinal wild plants consumed during drought. Study of rural Fulani, northeastern Nigeria. *Int. J. Food Sci. Nutr.* **2000**, *51*, 195–208.
63. Vinceti, B.; Eyzaguirre, P.; Johns, T. The Nutritional Role of Forest Plant Foods for Rural Communities.
    In *Human Health and Forests: A Global Overview of Issues, Practice, and Policy*; Colfer, C.J.P., Ed., Center for International Forestry Research/People and Plant International: Sterling, VA, USA, 2008, p. 66.
64. Patricia, O.; Zoue, L.; Megnanou, R.M.; Doue, R.; Niamke, S. Proximate Composition And Nutritive Value Of Leafy Vegetables Consumed In Northern Côte d’Ivoire. *Eur. Sci. J.* **2014**, *10*, 212–227.
65. Nyanga, L.K.; Gadaga, T.H.; Nout, M.J.; Smid, E.J.; Boekhout, T.; Zwietering, M.H. Nutritive value of masau (*Ziziphus mauritiana*) fruits from Zambezi Valley in Zimbabwe. *Food Chem.* **2013**, *138*, 168–172.
66. Lockett, C.T.; Calvert, C.C.; Grivetti, L.E. Energy and micronutrient composition of dietary and medicinal wild plants consumed during drought. Study of rural Fulani, northeastern Nigeria. *Int. J. Food Sci. Nutr.* **2000**, *51*, 195–208.
67. Moreno–Jiménez, E.; Peñalosa, J.M.; Manzano, R.; Carpena–Ruiz, R.O.; Gamarra, R.; Esteban, E. Heavy metals distribution in soils surrounding an abandoned mine in NW Madrid (Spain) and their transference to wild flora. *J. Hazard. Mater.* **2009**, *162*, 854–859.
68. Moreno–Jiménez, E.; Peñalosa, J.M.; Manzano, R.; Carpena-Ruiz, R.O.; Gamarra, R.; Esteban, E. Heavy metals distribution in soils surrounding an abandoned mine in NW Madrid (Spain) and their transference to wild flora. *J. Hazard. Mater.* **2009**, *162*, 854–859.
69. De la Fuente, V.; Rufo, L.; Rodríguez, N.; Amils, R.; Zuluaga, J. Metal accumulation screening of the Río Tinto flora (Huelva, Spain). *Biol. Trace Elem. Res*. **2010**, *134*, 318–341.
70. Wan, K.; Chen, F.; Tao, Y.; Chen, S. Nutrient Elements in Leaves of Rare and Endangered Species in Wuhan Botanical Garden, China*. J. Plant. Nutr.* **2009**, *32*, 1914–1940.
71. Vishwakarma, K.L.; Dubey, V. Nutritional analysis of indigenous wild edible herbs used in eastern Chhattisgarh, India. Emir. *J. Food Agric.* **2011**, *23*, 554–560.
72. Wan, K.; Chen, F.; Tao, Y.; Chen, S. Nutrient Elements in Leaves of Rare and Endangered Species in Wuhan Botanical Garden, China. *J. Plant. Nutr.* **2009**, *32*, 1914–1940.
73. Tsuchiya, Y.; Shimogaki, H.; Abe, H.; Kagawa, A. Inorganic elements in typical Japanese trees for woody biomass fuel. *Wood Sci.* **2010**, *56*, 53–63.
74. Tsuchiya, Y.; Shimogaki, H.; Abe, H.; Kagawa, A. Inorganic elements in typical Japanese trees for woody biomass fuel. *Wood Sci.* **2010**, *56*, 53–63.
75. Memon, A.R.; Itô, S.; Yatazawa, M. Absorption and accumulation of iron, manganese and copper in plants in the temperate forest of central Japan. *Soil Sci. Plant. Nutr.* **1979**, *15*, 611–620.
76. Tsuchiya, Y.; Shimogaki, H.; Abe, H.; Kagawa, A. Inorganic elements in typical Japanese trees for woody biomass fuel. *Wood Sci.* **2010**, *56*, 53–63.
77. Wan, K.; Chen, F.; Tao, Y.; Chen, S. Nutrient Elements in Leaves of Rare and Endangered Species in Wuhan Botanical Garden, China. *J. Plant. Nutr.* **2009**, *32*, 1914–1940.
78. Lockett, C.T.; Calvert, C.C.; Grivetti, L.E. Energy and micronutrient composition of dietary and medicinal wild plants consumed during drought. Study of rural Fulani, northeastern Nigeria. *Int. J. Food Sci. Nutr.* **2000**, *51*, 195–208.
79. Agunbiade, O.S.; Ojezele, O.M.; Ojezele, J.O.; Ajayi, A.Y. Hypoglycaemic activity of *Commelina africana* and *Ageratum conyzoides* in relation to their mineral composition. *Afr. Health Sci.* **2012**, *12*, 198–203.
80. Franco, M.J.; Caetano, I.C.S.; Caetano, J.; Dragunski, D.C. Determinação de Metais em Plantas Medicinais Comercializadas na Região de Umuarama–PR. *Arq. Ciênc. Saúde. UNIPAR, Umuarama.* **2011**, *15*, 121–127.
81. Franco, M.J.; Caetano, I.C.S.; Caetano, J.; Dragunski, D.C. Determinação de Metais em Plantas Medicinais Comercializadas na Região de Umuarama–PR. *Arq. Ciênc. Saúde. UNIPAR, Umuarama.,* **2011**, *15*, 121–127.
82. Dim LA, Funtua II, Oyewale, A.O., *et al.* Determination of some elements in Ageratum conyziodes, a tropical medicinal plant, using instrumental neutron activation analysis. *J. Radioanalytical and Nuclear Chem.* **2004**, *261*, 225–228.
83. De la Fuente, V.; Rufo, L.; Rodríguez, N.; Amils, R.; Zuluaga, J. Metal accumulation screening of the Río Tinto flora (Huelva, Spain). *Biol. Trace Elem. Res.* **2010**, *134*, 318–341.
84. Liu, Y.; Ding, H.; Zhu, Y. Metal bioaccumulation in plant leaves from an industrious area and the Botanical Garden in Beijing*. J. Environ. Sci.* **2005**, *17*, 294–300.
85. Liu, Y.; Ding, H.; Zhu, Y. Metal bioaccumulation in plant leaves from an industrious area and the Botanical Garden in Beijing*. J. Environ. Sci.* **2005**, *17*, 294–300.
86. Moreno-Jiménez, E.; Peñalosa, J.M.; Manzano, R.; Carpena-Ruiz, R.O.; Gamarra, R.; Esteban, E. Heavy metals distribution in soils surrounding an abandoned mine in NW Madrid (Spain) and their transference to wild flora. *J. Hazard. Mater.* **2009**, *162*, 854–859.
87. Moreno-Jiménez, E.; Peñalosa, J.M.; Manzano, R.; Carpena-Ruiz, R.O.; Gamarra, R.; Esteban, E. Heavy metals distribution in soils surrounding an abandoned mine in NW Madrid (Spain) and their transference to wild flora. *J. Hazard. Mater.* **2009**, *162*, 854–859.
88. Rode, K.D.; Chapman, C.A.; Chapman, L.J.; McDowell, L.R. Mineral Resource Availability and Consumption by Colobus in Kibale National Park, Uganda. *Int. J. Primatol.* **2003**, *24*, 541–573.
89. Rode, K.D.; Chapman, C.A.; Chapman, L.J.; McDowell, L.R. Mineral Resource Availability and Consumption by Colobus in Kibale National Park, Uganda. *Int. J. Primatol.* **2003**, *24*, 541–573.
90. Rode, K.D.; Chapman, C.A.; Chapman, L.J.; McDowell, L.R. Mineral Resource Availability and Consumption by Colobus in Kibale National Park, Uganda. *Int. J. Primatol.* **2003**, *24*, 541–573.
91. Rode, K.D.; Chapman, C.A.; Chapman, L.J.; McDowell, L.R. Mineral Resource Availability and Consumption by Colobus in Kibale National Park, Uganda. *Int. J. Primatol.*. **2003**, *24*, 541–573.
92. Rode, K.D.; Chapman, C.A.; Chapman, L.J.; McDowell, L.R. Mineral Resource Availability and Consumption by Colobus in Kibale National Park, Uganda. *Int. J. Primatol.* **2003**, *24*, 541–573.
93. Rode, K.D.; Chapman, C.A.; Chapman, L.J.; McDowell, L.R. Mineral Resource Availability and Consumption by Colobus in Kibale National Park, Uganda. *Int. J. Primatol.*. **2003**, *24*, 541–573.
94. Grzegorczyk, S.; Olszewska, M.; Alberski, J. Accumulation of copper, zinc, manganese and iron by selected species of grassland legumes and herbs*. J. Elem.* **2014**, *19*, 109–118.
95. Grzegorczyk, S.; Olszewska, M.; Alberski, J. Accumulation of copper, zinc, manganese and iron by selected species of grassland legumes and herbs*. J. Elem*. **2014**, *19*, 109–118.
96. Grzegorczyk, S.; Alberski, J. Contents of some micronutrients in selected species of meadow–pasture herbs. *Zeszyty Problemowe Postepow Nauk Rolniczych.* **2000**, *471*, 705–710.
97. Towhidi, A.; Saberifar, T.; Dirandeh, E. Nutritive value of some herbage for dromedary camels in the central arid zone of Iran. *Trop. Anim. Health Prod.* **2011**, *43*, 617–622.
98. Seal, T.; Pillai, B.; Chaudhuri, K. Nutritive Value and Mineral Composition of Some Wild Edible Plants from Meghalaya State in India. *Adv. Biol. Res*. **2014**, *8*, 116–122.
99. Golubev, F.V.; Golubkina, N.A.; Gorbunov, Y.N. Mineral Composition of Wild Onions and Their Nutritional Value. *Appl. Biochem. Microbiol.* **2003**, *39*, 532–535.
100. Karmakar, K.; Muslim, T.; Rahman, M.A. Chemical Composition of Some Leafy Vegetables of Bangladesh. *Dhaka Univ. J. Sci.* **2013**, *61*, 199–201.
101. Dini, I.; Tenore, G.C.; Dini, A. Chemical composition, nutritional value and antioxidant properties of *Allium caepa* L. Var. tropeana (red onion) seeds. *Food Chem.* **2008**, *107*, 613–621.
102. Dini, I.; Tenore, G.C.; Dini, A. Chemical composition, nutritional value and antioxidant properties of *Allium caepa* L. Var. tropeana (red onion) seeds. *Food Chem.* **2008**, *107*, 613–621.
103. Ali, M.H.H.; Al–Quahtani, K. Assessment of some heavy metals in vegetables, cereals and fruits in Saudi Arabian markets. *Egypt. J. Aquatic Res.* **2012**, *38*, 31–37.
104. Golubev, F.V.; Golubkina, N.A.; Gorbunov, Y.N. Mineral Composition of Wild Onions and Their Nutritional Value. *Appl. Biochem. Microbiol.* **2003**, *39*, 532–535.
105. Golubev, F.V.; Golubkina, N.A.; Gorbunov, Y.N. Mineral Composition of Wild Onions and Their Nutritional Value. *Appl. Biochem. Microbiol.* **2003**, *39*, 532–535.
106. Golubev, F.V.; Golubkina, N.A.; Gorbunov, Y.N. Mineral Composition of Wild Onions and Their Nutritional Value. *Appl. Biochem. Microbiol.*. **2003**, *39*, 532–535.
107. Golubev, F.V.; Golubkina, N.A.; Gorbunov, Y.N. Mineral Composition of Wild Onions and Their Nutritional Value. *Appl. Biochem. Microbiol.* **2003**, *39*, 532–535.
108. Golubev, F.V.; Golubkina, N.A.; Gorbunov, Y.N. Mineral Composition of Wild Onions and Their Nutritional Value. *Appl. Biochem. Microbiol.* **2003**, *39*, 532–535.
109. Golubev, F.V.; Golubkina, N.A.; Gorbunov, Y.N. Mineral Composition of Wild Onions and Their Nutritional Value. *Appl. Biochem. Microbiol.* **2003**, *39*, 532–535.
110. Golubev, F.V.; Golubkina, N.A.; Gorbunov, Y.N. Mineral Composition of Wild Onions and Their Nutritional Value. *Appl. Biochem. Microbiol.* **2003**, *39*, 532–535.
111. Duke, J.A.; Ayensu, E.S. Medicinal Plants of China. Cited by http://www.pfaf.org/user/
     Plant.aspx?LatinName=Allium+schoenoprasum (accessed on 23 July 2014).
112. Golubev, F.V.; Golubkina, N.A.; Gorbunov, Y.N. Mineral Composition of Wild Onions and Their Nutritional Value. *Appl. Biochem. Microbiol.* **2003**, *39*, 532–535.
113. Golubev, F.V.; Golubkina, N.A.; Gorbunov, Y.N. Mineral Composition of Wild Onions and Their Nutritional Value. *Appl. Biochem. Microbiol.* **2003**, *39*, 532–535.
114. Tsuchiya, Y.; Shimogaki, H.; Abe, H.; Kagawa, A. Inorganic elements in typical Japanese trees for woody biomass fuel. *Wood Sci.* **2010**, *56*, 53–63.
115. Mayer, A.M.; Gorham, E. The Iron and Manganese Content of Plants present in the Natural Vegetation of the English Lake District. *Ann. Bot.* **1951**, *15*, 247–263.
116. Malik, J.; Frankova, A.; Drabek, O.; Szakova, J.; Ash, C.; Kokoska, L. Aluminium and other elements in selected herbal tea plant species and their infusions. *Food Chem*. **2013**, *139*, 728–34
117. Ansari, T.M.; Ikram, N.; Najam-ul-Haq, M.; Fayyaz, Y.; Fayyaz, Q.; Ghafoor, I.; Khalid, N. Essential Trace Metal (Zinc, Manganese, Copper and Iron) Levels in Plants of Medicinal Importance. *J. Biol. Sci.* **2004**, *4*, 95–99.
118. Gbadamosi IT, Obogo SF. Chemical Constituents and In Vitro Antimicrobial Activities of Five botanicals Used Traditionally for the Treatment of Neonatal Jaundice in Ibadan, Nigeria. *Nat. Sci.* **2013**, *11*, 130–135.
119. Borah S, Baruah AM, Das AK, Borah J. Determination of Mineral Content in Commonly Consumed Leafy Vegetables. *Food Anal. Methods.* 2009; 2:226–230.
120. Mazumdar, K.; Das, S. Phytoremediation of Pb, Zn, Fe, and Mg with 25 wetland plant species from a paper mill contaminated site in North East India. *Environ. Sci. Pollut. Res.* **2014**, doi:10.1007/s11356-014-3377-7.
121. Mazumdar, K.; Das, S. Phytoremediation of Pb, Zn, Fe, and Mg with 25 wetland plant species from a paper mill contaminated site in North East India. *Environ. Sci. Pollut. Res.* **2014**, doi:10.1007/s11356-014-3377-7.
122. Chizzola, R.; Michitsch, H.; Franz, C. Monitoring of metallic micronutrients and heavy metals in herbs, spices and medicinal plants from Austria. *Eur Food Res Technol.* **2003**, *216*, 407–411.
123. Divrikli, U.; Horzum, N.; Soylak, M.; Elci, L.Trace heavy metal contents of some spices and herbal plants from western Anatolia, Turkey. *Int. J. Food Sci. Technol.* **2006**, *41*, 712–716.
124. Ugulu, I.; Dogan, Y.; Baslar, S.; Varol, O. Biomonitoring of trace element accumulation in plants growing at Murat Mountain. *Int. J. Environ. Sci. Technol.* **2012**, *9*, 527–534.
125. Ugulu, I.; Dogan, Y.; Baslar, S.; Varol, O. Biomonitoring of trace element accumulation in plants growing at Murat Mountain. *Int. J. Environ. Sci. Technol.* **2012**, *9*, 527–534.
126. Rangarajan, A.; Kelly, J.F. Iron Bioavailability from Amaranthus Species: 1—*In Vitro* Dialysable Iron for Estimation of Genetic Variation. *J. Sci. Food Agric.* **1998**, *78*, 267–273.
127. Rangarajan, A.; Kelly, J.F. Iron Bioavailability from Amaranthus Species: 1—*In Vitro* Dialysable Iron for Estimation of Genetic Variation. *J. Sci. Food Agric.* **1998**, *78*, 267–273.
128. Rangarajan, A.; Kelly, J.F. Iron Bioavailability from Amaranthus Species: 1—*In Vitro* Dialysable Iron for Estimation of Genetic Variation. *J. Sci. Food Agric.* **1998**, *78*, 267–273.
129. Rangarajan, A.; Kelly, J.F. Iron Bioavailability from Amaranthus Species: 1—*In Vitro* Dialysable Iron for Estimation of Genetic Variation. *J. Sci. Food Agric.* **1998**, *78*, 267–273.
130. Rangarajan A, Kelly JF. Iron Bioavailability from Amaranthus Species: 1—*In Vitro* Dialysable Iron for Estimation of Genetic Variation. *J. Sci. Food Agric.* **1998**, *78*, 267–273.
131. Rangarajan, A.; Kelly, J.F. Iron Bioavailability from Amaranthus Species: 1—*In Vitro* Dialysable Iron for Estimation of Genetic Variation. *J. Sci. Food Agric.* **1998**, *78*, 267–273.
132. Rangarajan, A.; Kelly, J.F. Iron Bioavailability from Amaranthus Species: 1—*In Vitro* Dialysable Iron for Estimation of Genetic Variation. *J. Sci. Food Agric.* **1998**, *78*, 267–273.
133. Rangarajan, A.; Kelly, J.F. Iron Bioavailability from Amaranthus Species: 1—*In Vitro* Dialysable Iron for Estimation of Genetic Variation. *J. Sci. Food Agric.* **1998**, *78*, 267–273.
134. Rangarajan, A.; Kelly, J.F.Iron Bioavailability from Amaranthus Species: 1—*In Vitro* Dialysable Iron for Estimation of Genetic Variation. *J. Sci. Food Agric.* **1998**, *78*, 267–273.
135. Vishwakarma, K.L.; Dubey, V.Nutritional analysis of indigenous wild edible herbs used in eastern Chhattisgarh, India. *Emir. J. Food Agric.* **2011**, *23*, 554–560.
136. Rangarajan, A.; Kelly, J.F. Iron Bioavailability from Amaranthus Species: 1—In Vitro Dialysable Iron for Estimation of Genetic Variation. *J. Sci. Food Agric.* **1998**, *78*, 267–273.
137. Rangarajan, A.; Kelly, J.F. Iron Bioavailability from Amaranthus Species: 1—In Vitro Dialysable Iron for Estimation of Genetic Variation. *J. Sci. Food Agric.* **1998**, *78*, 267–273.
138. Rangarajan, A.; Kelly, J.F. Iron Bioavailability from Amaranthus Species: 1—In Vitro Dialysable Iron for Estimation of Genetic Variation. *J. Sci. Food Agric.* **1998**, *78*, 267–273.
139. Rangarajan, A.; Kelly, J.F. Iron Bioavailability from Amaranthus Species: 1—In Vitro Dialysable Iron for Estimation of Genetic Variation. *J. Sci. Food Agric.* **1998**, *78*, 267–273.
140. Mazumdar, K.; Das, S. Phytoremediation of Pb, Zn, Fe, and Mg with 25 wetland plant species from a paper mill contaminated site in North East India. *Environ. Sci. Pollut. Res.* **2014**, doi:10.1007/s11356-014-3377-7.
141. Rangarajan, A.; Kelly, J.F.Iron Bioavailability from Amaranthus Species: 1—*In Vitro* Dialysable Iron for Estimation of Genetic Variation. *J. Sci. Food Agric.* **1998**, *78*, 267–273.
142. Rangarajan, A.; Kelly, J.F. Iron Bioavailability from Amaranthus Species: 1—*In Vitro* Dialysable Iron for Estimation of Genetic Variation. *J. Sci. Food Agric.* **1998**, *78*, 267–273.
143. Mazumdar, K.; Das, S. Phytoremediation of Pb, Zn, Fe, and Mg with 25 wetland plant species from a paper mill contaminated site in North East India. *Environ. Sci. Pollut. Res.* **2014**, doi:10.1007/s11356-014-3377-7.
144. Pillay, V.; Jonnalagadda, S.B. Elemental uptake by edible herbs and lettuce (*Latuca sativa*). *J.* *Environ. Sci. Health Part B* **2007**, *42*, 423–428.
145. Rangarajan, A.; Kelly, J.F. Iron Bioavailability from Amaranthus Species: 1—*In Vitro* Dialysable Iron for Estimation of Genetic Variation. *J. Sci. Food Agric.* **1998**, *78*, 267–273.
146. Rangarajan A, Kelly JF. Iron Bioavailability from Amaranthus Species: 1—*In Vitro* Dialysable Iron for Estimation of Genetic Variation. *J. Sci. Food Agric.* **1998**, *78*, 267–273.
147. Rangarajan, A.; Kelly, J.F. Iron Bioavailability from Amaranthus Species: 1—*In Vitro* Dialysable Iron for Estimation of Genetic Variation. *J. Sci. Food Agric.* **1998**, *78*, 267–273.
148. Rangarajan, A.; Kelly, J.F. Iron Bioavailability from Amaranthus Species: 1—*In Vitro* Dialysable Iron for Estimation of Genetic Variation. *J. Sci. Food Agric.* **1998**, *78*, 267–273
149. Rangarajan, A.; Kelly, J.F. Iron Bioavailability from Amaranthus Species: 1—*In Vitro* Dialysable Iron for Estimation of Genetic Variation. *J. Sci. Food Agric.* **1998**, *78*, 267–273.
150. Rangarajan, A.; Kelly, J.F. Iron Bioavailability from Amaranthus Species: 1—*In Vitro* Dialysable Iron for Estimation of Genetic Variation. *J. Sci. Food Agric.* **1998**, *78*, 267–273.
151. Rangarajan, A.; Kelly, J.F. Iron Bioavailability from Amaranthus Species: 1—*In Vitro* Dialysable Iron for Estimation of Genetic Variation. *J. Sci. Food Agric.* **1998**, *78*, 267–273.
152. Rangarajan, A.; Kelly, J.F. Iron Bioavailability from Amaranthus Species: 1—*In Vitro* Dialysable Iron for Estimation of Genetic Variation. *J. Sci. Food Agric.* **1998**, *78*, 267–273.
153. Rangarajan, A.; Kelly, J.F. Iron Bioavailability from Amaranthus Species: 1—*In Vitro* Dialysable Iron for Estimation of Genetic Variation. *J. Sci. Food Agric.* **1998**, *78*, 267–273.
154. Rangarajan A, Kelly JF. Iron Bioavailability from Amaranthus Species: 1—*In Vitro* Dialysable Iron for Estimation of Genetic Variation. *J. Sci. Food Agric.* **1998**, *78*, 267–273.
155. Akubugwo, I.E.; Obasi, N.A.; Chinyere, G.C.; Ugbogu, A.E. Nutritional and chemical value of *Amaranthus hybridus* L. leaves from Afikpo, Nigeria. *Afr. J. Biotechnol.* **2007**, *6*, 2833–2839.
156. Rangarajan, A.; Kelly, J.F. Iron Bioavailability from Amaranthus Species: 1—*In Vitro* Dialysable Iron for Estimation of Genetic Variation. *J. Sci. Food Agric.* **1998**, *78*, 267–273.
157. Rangarajan, A.; Kelly, J.F. Iron Bioavailability from Amaranthus Species: 1—*In Vitro* Dialysable Iron for Estimation of Genetic Variation. *J. Sci. Food Agric.* **1998**, *78*, 267–273.
158. Rangarajan A, Kelly JF. Iron Bioavailability from Amaranthus Species: 1—*In Vitro* Dialysable Iron for Estimation of Genetic Variation. *J. Sci. Food Agric.* **1998**, *78*, 267–273.
159. Iheanako, K.; Udebuani, A. Nutritional Composition of Some Leafy Vegetables Consumed in Imo State, Nigeria. *J. Appl. Sci. Environ. Manag.* **2009**, *13*, 35–38.
160. Rangarajan, A.; Kelly, J.F. Iron Bioavailability from Amaranthus Species: 1—*In Vitro* Dialysable Iron for Estimation of Genetic Variation. *J. Sci. Food Agric.* **1998**, *78*, 267–273.
161. Rangarajan, A.; Kelly, J.F. Iron Bioavailability from Amaranthus Species: 1—*In Vitro* Dialysable Iron for Estimation of Genetic Variation. *J. Sci. Food Agric.* **1998**, *78*, 267–273.
162. Rangarajan, A.; Kelly, J.F. Iron Bioavailability from Amaranthus Species: 1—*In Vitro* Dialysable Iron for Estimation of Genetic Variation. *J. Sci. Food Agric.* **1998**, *78*, 267–273.
163. Rangarajan, A.; Kelly, J.F. Iron Bioavailability from Amaranthus Species: 1—*In Vitro* Dialysable Iron for Estimation of Genetic Variation. *J. Sci. Food Agric.* **1998**, *78*, 267–273.
164. Rangarajan, A.; Kelly, J.F. Iron Bioavailability from Amaranthus Species: 1—In Vitro Dialysable Iron for Estimation of Genetic Variation. *J. Sci. Food Agric.* **1998**, *78*, 267–273.
165. Rangarajan, A.; Kelly, J.F. Iron Bioavailability from Amaranthus Species: 1—*In Vitro* Dialysable Iron for Estimation of Genetic Variation. *J. Sci. Food Agric.* **1998**, *78*, 267–273.
166. Rangarajan, A.; Kelly, J.F. Iron Bioavailability from Amaranthus Species: 1—*In Vitro* Dialysable Iron for Estimation of Genetic Variation. *J. Sci. Food Agric.* **1998**, *78*, 267–273.
167. Rangarajan, A.; Kelly, J.F. Iron Bioavailability from Amaranthus Species: 1—*In Vitro* Dialysable Iron for Estimation of Genetic Variation. *J. Sci. Food Agric.* **1998**, *78*, 267–273.
168. Rangarajan, A.; Kelly, J.F. Iron Bioavailability from Amaranthus Species: 1—*In Vitro* Dialysable Iron for Estimation of Genetic Variation. *J. Sci. Food Agric.* **1998**, *78*, 267–273.
169. Agbaire, P.O. Nutritional and Anti–nutritional Levels of Some Local Vegetables (*Vernomia anydalira*, *Manihot esculenta*, *Teiferia occidentalis*, *Talinum triangulare*, *Amaranthus spinosus*) from Delta State, Nigeria. *J. Appl. Sci. Environ. Manag.* **2011**, *15*, 625–628
170. Duke, J.A.; Ayensu, E.S. Medicinal Plants of China. Available online: http://www.pfaf.org/user/
     Plant.aspx?LatinName=Amaranthus+spinosus (accessed on 23 July 2014).
171. Duke, J.A.; Ayensu, E.S. Medicinal Plants of China. Available online: http://www.pfaf.org/user/
     Plant.aspx?LatinName=Amaranthus+spinosus (accessed on 23 July 2014).
172. Borah, S.; Baruah, A.M.; Das, A.K.; Borah, J. Determination of Mineral Content in Commonly Consumed Leafy Vegetables. *Food Anal. Methods* **2009**, *2*, 226–230.
173. Mazumdar K, Das S. Phytoremediation of Pb, Zn, Fe, and Mg with 25 wetland plant species from a paper mill contaminated site in North East India. *Environ. Sci. Pollut. Res.* **2014**, doi:10.1007/s11356-014-3377-7.
174. Rangarajan, A.; Kelly, J.F. Iron Bioavailability from Amaranthus Species: 1—*In Vitro* Dialysable Iron for Estimation of Genetic Variation*. J. Sci. Food Agric.* **1998**, *78*, 267–273.
175. Olowoyo, J.O.; Okedeyi, O.O.; Mkolo, M.M.; Lion, G.N.; Mdakane, S.T.R. Uptake and translocation
     of heavy metals by medicinal plants growing around a waste dump site in Pretoria, South Africa. *S. Afr.
     J. Bot*. **2012**, *78*, 116–121.
176. Rangarajan, A.; Kelly, J.F. Iron Bioavailability from Amaranthus Species: 1—*In Vitro* Dialysable Iron for Estimation of Genetic Variation*. J. Sci. Food Agric.* **1998**, *78*, 267–273.
177. Mazumdar K, Das S. Phytoremediation of Pb, Zn, Fe, and Mg with 25 wetland plant species from a paper mill contaminated site in North East India. *Environ. Sci. Pollut. Res.* **2014**, doi:10.1007/s11356-014-3377-7.
178. Olowoyo, J.O.; Okedeyi, O.O.; Mkolo, M.M.; Lion, G.N.; Mdakane, S.T.R. Uptake and translocation
     of heavy metals by medicinal plants growing around a waste dump site in Pretoria, South Africa. *S. Afr.
     J. Bot.* **2012**, *78*, 116–121.
179. Olowoyo, J.O.; Okedeyi, O.O.; Mkolo, M.M.; Lion, G.N.; Mdakane, S.T.R. Uptake and translocation of heavy metals by medicinal plants growing around a waste dump site in Pretoria, South Africa. *S. Afr. J. Bot.* **2012**, *78*, 116–121.
180. Mazumdar, K.; Das, S. Phytoremediation of Pb, Zn, Fe, and Mg with 25 wetland plant species from a paper mill contaminated site in North East India. *Environ. Sci. Pollut. Res.* **2014**, doi:10.1007/s11356-014-3377-7.
181. Rangarajan, A.; Kelly, J.F. Iron Bioavailability from Amaranthus Species: 1—*In Vitro* Dialysable Iron for Estimation of Genetic Variation. *J. Sci. Food Agric.* **1998**, *78*, 267–273.
182. Schönfeldt, H.C.; Pretorius, B. The nutrient content of five traditional South African dark green leafy vegetables–A preliminary study. *J. Food Compos. Anal.* **2011**, *24*, 1141–1146.
183. Karmakar, K.; Muslim, T.; Rahman, M.A. Chemical Composition of Some Leafy Vegetables of Bangladesh. *Dhaka Univ. J. Sci.* **2013**, *61*, 199–201.
184. ***. Plants for a future. Amaranthus tricolor L. Available online: http://www.pfaf.org/user/
     Plant.aspx?LatinName=Amaranthus+tricolor (accessed on 18 July 2014).
185. Karmakar, K.; Muslim, T.; Rahman, M.A. Chemical Composition of Some Leafy Vegetables of Bangladesh. *Dhaka Univ. J. Sci.* **2013**, *61*, 199–201.
186. Rangarajan, A.; Kelly, J.F. Iron Bioavailability from Amaranthus Species: 1—*In Vitro* Dialysable Iron for Estimation of Genetic Variation. *J. Sci. Food Agric.* **1998**, *78*, 267–273.
187. Rangarajan, A.; Kelly, J.F. Iron Bioavailability from Amaranthus Species: 1—*In Vitro* Dialysable Iron for Estimation of Genetic Variation. *J. Sci. Food Agric.* **1998**, *78*, 267–273.
188. Rangarajan, A.; Kelly, J.F. Iron Bioavailability from Amaranthus Species: 1—*In Vitro* Dialysable Iron for Estimation of Genetic Variation. *J. Sci. Food Agric.* **1998**, *78*, 267–273.
189. Rangarajan, A.; Kelly, J.F. Iron Bioavailability from Amaranthus Species: 1—*In Vitro* Dialysable Iron for Estimation of Genetic Variation. *J. Sci. Food Agric.* **1998**, *78*, 267–273.
190. Rangarajan, A.; Kelly, J.F. Iron Bioavailability from Amaranthus Species: 1—*In Vitro* Dialysable Iron for Estimation of Genetic Variation. *J. Sci. Food Agric.* **1998**, *78*, 267–273.
191. Rangarajan, A.; Kelly, J.F. Iron Bioavailability from Amaranthus Species: 1—*In Vitro* Dialysable Iron for Estimation of Genetic Variation. *J. Sci. Food Agric.* **1998**, *78*, 267–273.
192. Rangarajan, A.; Kelly, J.F. Iron Bioavailability from Amaranthus Species: 1—*In Vitro* Dialysable Iron for Estimation of Genetic Variation. *J. Sci. Food Agric.* **1998**, *78*, 267–273.
193. Rangarajan, A.; Kelly, J.F. Iron Bioavailability from Amaranthus Species: 1—*In Vitro* Dialysable Iron for Estimation of Genetic Variation. *J. Sci. Food Agric.* **1998**, *78*, 267–273.
194. Rangarajan, A.; Kelly, J.F. Iron Bioavailability from Amaranthus Species: 1—In Vitro Dialysable Iron for Estimation of Genetic Variation. *J. Sci. Food Agric.* **1998**, *78*, 267–273.
195. Rangarajan, A.; Kelly, J.F. Iron Bioavailability from Amaranthus Species: 1—*In Vitro* Dialysable Iron for Estimation of Genetic Variation. *J. Sci. Food Agric.* **1998**, *78*, 267–273.
196. Rangarajan, A.; Kelly, J.F. Iron Bioavailability from Amaranthus Species: 1—*In Vitro* Dialysable Iron for Estimation of Genetic Variation. *J. Sci. Food Agric.* **1998**, *78*, 267–273.
197. Rangarajan, A.; Kelly, J.F. Iron Bioavailability from Amaranthus Species: 1—*In Vitro* Dialysable Iron for Estimation of Genetic Variation. *J. Sci. Food Agric.* **1998**, *78*, 267–273.
198. Rangarajan, A.; Kelly, J.F. Iron Bioavailability from Amaranthus Species: 1—*In Vitro* Dialysable Iron for Estimation of Genetic Variation. *J. Sci. Food Agric.* **1998**, *78*, 267–273.
199. Rangarajan, A.; Kelly, J.F. Iron Bioavailability from Amaranthus Species: 1—*In Vitro* Dialysable Iron for Estimation of Genetic Variation. *J. Sci. Food Agric.* **1998**, *78*, 267–273.
200. Rangarajan, A.; Kelly, J.F. Iron Bioavailability from Amaranthus Species: 1—*In Vitro* Dialysable Iron for Estimation of Genetic Variation. *J. Sci. Food Agric.* **1998**, *78*, 267–273.
201. Rangarajan, A.; Kelly, J.F. Iron Bioavailability from Amaranthus Species: 1—*In Vitro* Dialysable Iron for Estimation of Genetic Variation. *J. Sci. Food Agric.* **1998**, *78*, 267–273.
202. Borah, S.; Baruah, A.M.; Das, A.K.; Borah, J. Determination of Mineral Content in Commonly Consumed Leafy Vegetables. *Food Anal. Methods* **2009**, *2*, 226–230.
203. Duke, J.A.; Ayensu, E.S. Medicinal Plants of China. Available online: http://www.pfaf.org/user/
     plant.aspx?LatinName=Amaranthus+viridis (accessed on 23 July 2014).
204. Vishwakarma, K.L.; Dubey, V. Nutritional analysis of indigenous wild edible herbs used in eastern Chhattisgarh, India. *Emir. J. Food Agric*. **2011**, *23*, 554–560.
205. Pillay, V.; Jonnalagadda, S.B. Elemental uptake by edible herbs and lettuce (Latuca sativa). *J. Environ. Sci. Health Part B* **2007**, *42*, 423–428.
206. Olivares, E.; Pena, E.; Marcano, E.; Mostacero, J.; Aguiar, G.; Benitez, M.; Rengifo, E. Aluminum accumulation and its relationship with mineral plant nutrients in 12 pteridophytes from Venezuela. *Environ. Exp. Bot.* **2009**, *65*, 132–141.
207. Rangarajan, A.; Kelly, J.F. Iron Bioavailability from Amaranthus Species: 1—*In Vitro* Dialysable Iron for Estimation of Genetic Variation. *J. Sci. Food Agric.* **1998**, *78*, 267–273.
208. Ansari, T.M.; Ikram, N.; Najam-ul-Haq, M.; Fayyaz, Y.; Fayyaz, Q.; Ghafoor, I.; Khalid, N. Essential Trace Metal (Zinc, Manganese, Copper and Iron) Levels in Plants of Medicinal Importance. *J. Biol. Sci.* **2004**, *4*, 95–99.
209. Plants for a future. Available online: http://www.pfaf.org/user/Plant.aspx?LatinName=
     Amorphophallus+rivieri (accessed on 23 July 2014).
210. Brahma, J.; Singh, B.; Rethy, P.; Gajurel, P. Nutritional Analysis Of Some Selected Wild Edible Species Consumed By The Bodos Tribes of Kokrajhar District, BTC, Assam. *Asian J. Pharm. Clin. Res.* **2014**, *7*, 34–37.
211. Liu, Y.; Ding, H.; Zhu, Y. Metal bioaccumulation in plant leaves from an industrious area and the Botanical Garden in Beijing. *J. Environ. Sci.* **2005**, *17*, 294–300.
212. Liu, Y.; Ding, H.; Zhu, Y. Metal bioaccumulation in plant leaves from an industrious area and the Botanical Garden in Beijing. *J. Environ. Sci.* **2005**, *17*, 294–300.
213. Osaki, M.; Watanabe, T.; Ishizawa, T.; Nilnond, C.; Nuyim, T.; Sittibush, C.; Tadano, T. Nutritional characteristics in leaves of native plants grown in acid sulfate, peat, sandy podzolic, and saline soils distributed in Peninsular Thailand. *Plant Soil.* **1998**, *201*, 175–182.
214. Tanji, A.; Elgharous, M. A survey of mineral composition of weed seeds. *Weed Res.* **1998**, *38*, 79–86.
215. Tanji, A.; Elgharous, M. A survey of mineral composition of weed seeds. *Weed Res.* **1998**, *38*, 79–86.
216. Olivares E, Pena E, Marcano E, Mostacero J, Aguiar G, Benitez M, Rengifo E. Aluminum accumulation and its relationship with mineral plant nutrients in 12 pteridophytes from Venezuela. *Environ. Exp. Bot*. **2009**, *65*, 132–141.
217. Plants for a future. Anethum graveolens L. Available online: http://www.pfaf.org/user/
     Plant.aspx?LatinName=Anethum+graveolens (accessed on 23 July 2014).
218. Tokalıoğlu, Ş. Determination of trace elements in commonly consumed medicinal herbs by ICP-MS and multivariate analysis. *Food Chem.* **2012**, *134*, 2504–2508.
219. Dini, I.; Tenore, G.C.; Dini, A. Chemical composition, nutritional value and antioxidant properties of Allium caepa L. Var. tropeana (red onion) seeds. *Food Chem.* **2008**, *107*, 613–621.
220. Olivares, E.; Pena, E.; Marcano, E.; Mostacero, J.; Aguiar, G.; Benitez, M.; Rengifo, E. Aluminum accumulation and its relationship with mineral plant nutrients in 12 pteridophytes from Venezuela. *Environ. Exp. Bot.* **2009**, *65*, 132–141.
221. Vinceti, B.; Eyzaguirre, P.; Johns, T. The Nutritional Role of Forest Plant Foods for Rural Communities.
     In *Human Health and Forests: A Global Overview of Issues, Practice, and Policy*, Colfer, C.J.P., Ed.; Center for International Forestry Research/People and Plant International, Sterling, VA, USA, 2008; p. 66.
222. Lockett, C.T.; Calvert, C.C.; Grivetti, L.E. Energy and micronutrient composition of dietary and medicinal wild plants consumed during drought. Study of rural Fulani, northeastern Nigeria. *Int. J. Food Sci. Nutr.* **2000**, *51*, 195–208.
223. De la Fuente, V.; Rufo, L.; Rodríguez, N.; Amils, R.; Zuluaga, J. Metal accumulation screening of the Río Tinto flora (Huelva, Spain). *Biol. Trace Elem. Res.* **2010**, *134*, 318–341.
224. Schmidt, D.A.; Iambana, R.B.; Britt, A.; Junge, R.E.; Welch, C.R.; Porton, I.J.; Kerley, M.S. Nutrient composition of plants consumed by black and white ruffed lemurs, Varecia variegata, in the Betampona Natural Reserve, Madagascar. *Zoo Biol.* **2010**, *29*, 375–96.
225. Schmidt, D.A.; Iambana, R.B.; Britt, A.; Junge, R.E.; Welch, C.R.; Porton, I.J.; Kerley, M.S. Nutrient composition of plants consumed by black and white ruffed lemurs, Varecia variegata, in the Betampona Natural Reserve, Madagascar. *Zoo Biol.* **2010**, *29*, 375–396.
226. Pillay, V.; Jonnalagadda, S.B. Elemental uptake by edible herbs and lettuce (*Latuca sativa*). *J. Environ. Sci. Health Part B* **2007**, *42*, 423–428.
227. Hengweia, L.; MengXianga, G.; GuiZhenb, R. A Comparison of Nutritive Components in Apium graveolens and Wild Oenanthe javanica Plants. Available online: http://en.cnki.com.cn/Article_en/
     CJFDTOTAL-ZYSZ200701010.htm (accessed on 23 July 2014).
228. Xie, W.; Zhang, X.; Wang, T.; Hu, J. Botany, traditional uses, phytochemistry and pharmacology of *Apocynum venetum* L. (Luobuma): A review. *J. Ethnopharmacol.* **2012**, *141*, 1–8.
229. Animal Nutrition Group, National Dairy Development Board. Nutritive value of commonly available
     feeds and fodders in India, 2012. Available online: http://www.nddb.coop/English/Services/AN/
     Documents/Animal-Nutrition-booklet.pdf (accessed on 09 February 2014).
230. Sundriyal, M.; Sundriyal, R.C. Wild edible plants of the sikkim himalaya: nutritive values of selected species. *Econ. Bot.* **2001**, *55*, 377–390.
231. Cordenunsi, B.R.; de Menezes, E.W.; Genovese, M.I.; Colli, C.; de Souza, A.G.; Lajolo, F.M. Chemical Composition and Glycemic Index of Brazilian Pine (*Araucaria angustifolia*) Seeds. *J. Agric. Food Chem.* **2004**, *52*, 3412–3416.
232. De la Fuente V, Rufo L, Rodríguez N, Amils R, Zuluaga J. Metal accumulation screening of the Río Tinto flora (Huelva, Spain). *Biol. Trace Elem Res.* **2010**, *134*, 318–341.
233. Sundriyal, M.; Sundriyal, R.C. Wild edible plants of the sikkim himalaya: Nutritive values of selected species. *Econ. Bot.* **2001**, *55*, 377–390.
234. Sundriyal, M.; Sundriyal, R.C. Wild edible plants of the sikkim himalaya: Nutritive values of selected species. *Econ. Bot.* **2001**, *55*, 377–390.
235. Rajendran D, Balakrishnan V. Diet Composition, Biomass Yield and Mineral Contents of Vegetation in Native Tract of Mecheri Sheep. *Anim. Nutr. Feed Technol.* **2012**, *12*, 63–71.
236. Rajendran D, Balakrishnan V. Diet Composition, Biomass Yield and Mineral Contents of Vegetation in Native Tract of Mecheri Sheep. *Anim. Nutr. Feed Technol.* **2012**, *12*, 63–71.
237. Butnariu, M.; Bostan, C.; Samfira, I. Determination of mineral contents and antioxidant activity in some plants that contain allelochemicals of Banat region. *Studia Universitatis “Vasile Goldiş”, Seria Ştiinţele Vieţii* **2012**, *22*, 95–100.
238. De la Fuente, V.; Rufo, L.; Rodríguez, N.; Amils, R.; Zuluaga, J. Metal accumulation screening of the Río Tinto flora (Huelva, Spain). *Biol. Trace Elem. Res.* **2010**, *134*, 318–341.
239. Razić, S.; Dogo, S.; Slavković, L. Investigation on bioavailability of some essential and toxic elements in medicinal herbs. *J. Nat. Med.* **2008**, *62*, 340–344.
240. Razić, S.; Dogo, S.; Slavković, L. Investigation on bioavailability of some essential and toxic elements in medicinal herbs. *J. Nat. Med.* **2008**, *62*, 340–344.
241. Hussain, I.; Khattak, M.U.R.; Khan, F.A.; Rehman, I.U.; Khan, F.U.; Khan, F.U. Analysis of Heavy Metals
     in Selected Medicinal Plants from Dir, Swat and Peshawar Districts of Khyber Pakhtunkhwa*. J. Chem.
     Soc. Pak.* **2001**, *33*, 495–498.
242. Kashin, V.K. Vitally Important Microelements in Transbaikalian Herbs. *Chem. Sustain. Dev.* **2009**, *17*,
     371–381.
243. Kashin, V.K. Vitally Important Microelements in Transbaikalian Herbs. *Chem. Sustain. Dev.* **2009**, *17*,
     371–381.
244. Kashin VK. Vitally Important Microelements in Transbaikalian Herbs. *Chem. Sustain. Dev.* **2009**, *17*, 371–381.
245. Kashin, V.K. Vitally Important Microelements in Transbaikalian Herbs. *Chem. Sustain. Dev.* **2009**, *17*, 371–381.
246. Imelouane, B.; Tahri, M.; Elbastrioui, M.; Aouinti, F.; Elbachiri, A. Mineral Contents of Some Medicinal and Aromatic Plants Growing in Eastern Morocco. *J. Mater. Environ. Sci.* **2001**, *2*, 104–111.
247. Khuder, A.; Sawan, M.K.h.; Karjou, J.; Razouk, A.K. Determination of trace elements in Syrian medicinal plants and their infusions by energy dispersive X-ray fluorescence and total reflection X-ray fluorescence spectrometry. *Spectrochim Acta Part B* **2009**, *64*, 721–725.
248. Imelouane, B.; Tahri, M.; Elbastrioui, M.; Aouinti, F.; Elbachiri, A. Mineral Contents of Some Medicinal and Aromatic Plants Growing in Eastern Morocco. *J. Mater. Environ. Sci.* **2001**, *2*, 104–111.
249. Khuder, A.; Sawan, M.K.h.; Karjou, J.; Razouk, A.K. Determination of trace elements in Syrian medicinal plants and their infusions by energy dispersive X–ray fluorescence and total reflection X–ray fluorescence spectrometry. *Spectrochim Acta Part B* **2009**, *64*, 721–725.
250. Imelouane, B.; Tahri, M.; Elbastrioui, M.; Aouinti, F.; Elbachiri, A. Mineral Contents of Some Medicinal and Aromatic Plants Growing in Eastern Morocco. *J. Mater. Environ. Sci.* **2001**, *2*, 104–111.
251. Towhidi, A.; Saberifar, T.; Dirandeh, E. Nutritive value of some herbage for dromedary camels in the central arid zone of Iran. *Trop. Anim. Health Prod.* **2011**, *43*, 617–622.
252. Liu Y, Ding H，Zhu Y. Metal bioaccumulation in plant leaves from an industrious area and the Botanical Garden in Beijing. Journal of Environmental Sciences. **2005**, *17*, 294–300.
253. de la Fuente V, Rufo L, Rodríguez N, Amils R, Zuluaga J. Metal accumulation screening of the Río Tinto flora (Huelva, Spain). Biol Trace Elem Res. **2010**, *134*, 318–341.
254. Karunaratne AM, Amerasinghe PH, Sadagopa Ramanujam VM, Sandstead HH, Perera PAJ. Zinc, iron and phytic acid levels of some popular foods consumed by rural children in Sri Lanka. Journal of Food Composition and Analysis. **2008**, *21*, 481–488.
255. Karunaratne AM, Amerasinghe PH, Sadagopa Ramanujam VM, Sandstead HH, Perera PAJ. Zinc, iron and phytic acid levels of some popular foods consumed by rural children in Sri Lanka. Journal of Food Composition and Analysis. **2008**, *21*, 481–488.
256. Duke JA, Ayensu ES. Medicinal Plants of China. Available online: http://www.pfaf.org/user/
     Plant.aspx?LatinName=Asparagus+officinalis (accessed on 23 July 2014).
257. Cornara L, Roccotiello E, Minganti V, Drava G, De Pellegrini R, Mariotti MG. Level of trace elements in Pteridophytes growing on serpentine and metalliferous soils. *J. Plant Nutr. Soil Sci.* **2007**, *170*, 781–787.
258. Cornara L, Roccotiello E, Minganti V, Drava G, De Pellegrini R, Mariotti MG. Level of trace elements in Pteridophytes growing on serpentine and metalliferous soils. *J. Plant Nutr. Soil Sci.* **2007**, *170*, 781–787.
259. Cornara L, Roccotiello E, Minganti V, Drava G, De Pellegrini R, Mariotti MG. Level of trace elements in Pteridophytes growing on serpentine and metalliferous soils. *J. Plant Nutr. Soil Sci.* **2007**, *170*, 781–787.
260. Cornara L, Roccotiello E, Minganti V, Drava G, De Pellegrini R, Mariotti MG. Level of trace elements in Pteridophytes growing on serpentine and metalliferous soils. *J. Plant Nutr. Soil Sci.* **2007**, *170*, 781–787.
261. Wan K, Chen F, Tao Y, Chen S. Nutrient Elements in Leaves of Rare and Endangered Species in Wuhan Botanical Garden, China. *J. Plant. Nutr.* **2009**, *32*, 1914–1940.
262. Cornara L, Roccotiello E, Minganti V, Drava G, De Pellegrini R, Mariotti MG. Level of trace elements in Pteridophytes growing on serpentine and metalliferous soils. *J. Plant Nutr. Soil Sci.* **2007**, *170*, 781–787.
263. Cornara L, Roccotiello E, Minganti V, Drava G, De Pellegrini R, Mariotti MG. Level of trace elements in Pteridophytes growing on serpentine and metalliferous soils. *J. Plant Nutr. Soil Sci.* **2007**, *170*, 781–787.
264. Mayer AM, Gorham E. The Iron and Manganese Content of Plants present in the Natural Vegetation of the English Lake District. *Ann. Bot.* **1951**, *15*, 247–263.
265. Tanji A, Elgharous M. A survey of mineral composition of weed seeds. Weed Research. **1998**, *38*, 79–86.
266. Malayeri BE, Chehregani A, Yousefi N, Lorestani B. Identification of the Hyper Acumulator Plants in Copper and Iron Mine in Iran. *Pakistan J. Biol.Sci.* **2008**, *11*, 490–492.
267. Cornara L, Roccotiello E, Minganti V, Drava G, De Pellegrini R, Mariotti MG. Level of trace elements in Pteridophytes growing on serpentine and metalliferous soils. *J. Plant Nutr. Soil Sci.* **2007**, *170*, 781–787.
268. Cornara L, Roccotiello E, Minganti V, Drava G, De Pellegrini R, Mariotti MG. Level of trace elements in Pteridophytes growing on serpentine and metalliferous soils. *J. Plant Nutr. Soil Sci.* **2007**, *170*, 781–787.
269. Moreno–Jiménez E, Peñalosa JM, Manzano R, Carpena–Ruiz RO, Gamarra R, Esteban E. Heavy metals distribution in soils surrounding an abandoned mine in NW Madrid (Spain) and their transference to wild flora. *J. Hazard. Mater.* **2009**, *162*, 854–859.
270. Moreno–Jiménez E, Peñalosa JM, Manzano R, Carpena–Ruiz RO, Gamarra R, Esteban E. Heavy metals distribution in soils surrounding an abandoned mine in NW Madrid (Spain) and their transference to wild flora. *J. Hazard. Mater.* **2009**, *162*, 854–859.
271. Mayer AM, Gorham E. The Iron and Manganese Content of Plants present in the Natural Vegetation of the English Lake District. *Ann. Bot.* **1951**, *15*, 247–263.
272. Guerrero–Cervantes M, Ramírez RG, González–Rodríguez H, Cerrillo–Soto A, Juárez–Réyes A. Mineral content in range forages from north Mexico. *J. Appl. Anim. Res.* **2012**, *40*, 102–107.
273. Guerrero–Cervantes M, Ramírez RG, González–Rodríguez H, Cerrillo–Soto A, Juárez–Réyes A. Mineral content in range forages from north Mexico. *J. Appl. Anim. Res.* **2012**, *40*, 102–107.
274. Towhidi A, Saberifar T, Dirandeh E. Nutritive value of some herbage for dromedary camels in the central arid zone of Iran. Trop Anim Health Prod. **2011**, *43*, 617–622.
275. de la Fuente V, Rufo L, Rodríguez N, Amils R, Zuluaga J. Metal accumulation screening of the Río Tinto flora (Huelva, Spain). Biol Trace Elem Res. **2010**, *134*, 318–41.
276. Echeverri JA, Roman–Jitdutjaño OE. Witoto Ash Salts from the Amazon. Journal of Ethnopharmacology. **2011**, *138*, 492–502.
277. Animal Nutrition Group, National Dairy Development Board. Nutritive value of commonly available feeds and fodders in India, 2012. Available online:: http://www.nddb.coop/English/Services/AN/
     Documents/Animal–Nutrition–booklet.pdf (2 September 2014).
278. Animal Nutrition Group, National Dairy Development Board. Nutritive value of commonly available feeds and fodders in India, 2012. Available online:: http://www.nddb.coop/English/Services/AN/
     Documents/Animal–Nutrition–booklet.pdf (2 September 2014).
279. Tanji A, Elgharous M. A survey of mineral composition of weed seeds. Weed Research. **1998**, *38*, 79–86.
280. Naga Raju GJ, Sarita P, Ramana Murty GAV, Ravi Kumar M, Seetharami Reddy B, John Charles M, Lakshminarayana S, Seshi Reddy T, Bhuloka Reddy S, Vijayan V. stimation of trace elements in some anti–diabetic medicinal plants using PIXE technique. *Appl. Radiation Isotopes.* **2006**, *64*, 893–900.
281. Ansari TM, Ikram N, Najam–ul–Haq M, Fayyaz Y, Fayyaz Q, Ghafoor I, Khalid N. Essential Trace Metal (Zinc, Manganese, Copper and Iron) Levels in Plants of Medicinal Importance. *J. Biol. Sci.* **2004**, *4*, 95–99.
282. Vinceti B, Eyzaguirre P, Johns T. The Nutritional Role of Forest Plant Foods for Rural Communities. *Human Health and Forests: A Global Overview of Issues, Practice, and Policy*; Colfer, C.J.P., Ed. Center for International Forestry Research/People and Plant International: Sterling, VA, USA, 2008; p. 66.
283. Sundriyal M, Sundriyal RC. Wild edible plants of the sikkim himalaya: nutritive values of selected species. Economic Botany. **2001**, *55*, 377–390.
284. Franco, M.J.; Caetano ICS, Caetano J, Dragunski DC. Determinação de Metais em Plantas Medicinais Comercializadas na Região de Umuarama–PR. Arq. Ciênc. Saúde UNIPAR, Umuarama, **2011**, *15*, 121–127.
285. Franco MJ, Caetano ICS, Caetano J, Dragunski DC. Determinação de Metais em Plantas Medicinais Comercializadas na Região de Umuarama–PR. Arq. Ciênc. Saúde UNIPAR, Umuarama, **2011**, *15*, 121–127.
286. Igile GO, Iwara IA, Mgbeje BIA, Uboh FE, Ebong PE. Phytochemical, Proximate and Nutrient Composition of Vernonia calvaona Hook (Asterecea): A Green–Leafy Vegetable in Nigeria. Journal of Food Research. 2013; 2. doi:10.5539/jfr.v2n6p1.
287. Osaki M, Watanabe T, Ishizawa T, Nilnond C, Nuyim T, Sittibush C, Tadano T. Nutritional characteristics in leaves of native plants grown in acid sulfate, peat, sandy podzolic, and saline soils distributed in Peninsular Thailand. Plant and Soil. **1998**, *201*, 175–182.
288. Lockett CT, Calvert CC, Grivetti LE. Energy and micronutrient composition of dietary and medicinal wild plants consumed during drought. Study of rural Fulani, northeastern Nigeria. International Journal of Food Sciences and Nutrition; May **2000**, *51*, 195–208.
289. Lockett CT, Calvert CC, Grivetti LE. Energy and micronutrient composition of dietary and medicinal wild plants consumed during drought. Study of rural Fulani, northeastern Nigeria. International Journal of Food Sciences and Nutrition; May **2000**, *51*, 195–208.
290. Karmakar K, Muslim T, Rahman MA. Chemical Composition of Some Leafy Vegetables of Bangladesh. Dhaka Univ. J. Sci. **2013**, *61*, 199–201.
291. Vishwakarma KL, Dubey V. Nutritional analysis of indigenous wild edible herbs used in eastern Chhattisgarh, India. *Emir. J. Food Agric*. **2011**, *23*, 554–560.
292. Franco MJ, Caetano ICS, Caetano J, Dragunski DC. Determinação de Metais em Plantas Medicinais Comercializadas na Região de Umuarama–PR. Arq. Ciênc. Saúde UNIPAR, Umuarama, **2011**, *15*, 121–127.
293. Franco MJ, Caetano ICS, Caetano J, Dragunski DC. Determinação de Metais em Plantas Medicinais Comercializadas na Região de Umuarama–PR. Arq. Ciênc. Saúde UNIPAR, Umuarama, **2011**, *15*, 121–127.
294. Vishwakarma KL, Dubey V. Nutritional analysis of indigenous wild edible herbs used in eastern Chhattisgarh, India. *Emir. J. Food Agric*. **2011**, *23*, 554–560.
295. Seal T. Evaluation of Nutritional Potential of Wild Edible Plants Traditionally Used by The Tribal People of Meghalaya State in India. American Journal of Plant Nutrition and Fertilization Technology. **2012**, *2*, 19–26.
296. dos Santos UM, de Carvalho Gonçalvesa JF, Feldpausch TR. Growth, leaf nutrient concentration and photosynthetic nutrient use efficiency in tropical tree species planted in degraded areas in central Amazonia. Forest Ecology and Management. **2006**, *226*, 299–309.
297. Duke JA, Ayensu ES. Medicinal Plants of China. Available online: http://www.pfaf.org/user/Plant.aspx?LatinName=Benincasa+hispida (accessed on 23 July 2014).
298. Karmakar K, Muslim T, Rahman MA. Chemical Composition of Some Leafy Vegetables of Bangladesh. Dhaka Univ. J. Sci. **2013**, *61*, 199–201.
299. Rajendran D, Balakrishnan V. Diet Composition, Biomass Yield and Mineral Contents of Vegetation in Native Tract of Mecheri Sheep. Animal Nutrition and Feed Technology. **2012**, *12*, 63–71.
300. Keely P, Martinsen CS, Hunn ES, Norton HH. Composition of Native American Fruits in the Pacific Nortwest. Journal of the American Dietetic Association. **1982**, *81*, 568–572.
301. Tanji A, Elgharous M. A survey of mineral composition of weed seeds. Weed Research. **1998**, *38*, 79–86.
302. Marbaniang DG, Baruah P, Decruse R, Dkhar ER, Diengdoh DF, Nongpiur CL. Study of the Trace Metal Concentration in Some Local Vegetables Available in Shillong City, Meghlaya, India. International Journal of Environmental Protection. **2012**, *2*, 24–28.
303. Vishwakarma KL, Dubey V. Nutritional analysis of indigenous wild edible herbs used in eastern Chhattisgarh, India. *Emir. J. Food Agric.* **2011**, *23*, 554–560.
304. Divrikli U, Horzum N, Soylak M, Elci L. Trace heavy metal contents of some spices and herbal plants from western Anatolia, Turkey. *Int. J. Food Sci. Technol.* **2006**, *41*, 712–716.
305. Divrikli U, Horzum N, Soylak M, Elci L. Trace heavy metal contents of some spices and herbal plants from western Anatolia, Turkey. *Int. J. Food Sci. Technol.* **2006**, *41*, 712–716.
306. Marbaniang DG, Baruah P, Decruse R, Dkhar ER, Diengdoh DF, Nongpiur CL. Study of the Trace Metal Concentration in Some Local Vegetables Available in Shillong City, Meghlaya, India. International Journal of Environmental Protection. **2012**, *2*, 24–28.
307. Moreno–Jiménez E, Peñalosa JM, Manzano R, Carpena–Ruiz RO, Gamarra R, Esteban E. Heavy metals distribution in soils surrounding an abandoned mine in NW Madrid (Spain) and their transference to wild flora. *J. Hazard. Mater.* **2009**, *162*, 854–859.
308. Moreno–Jiménez E, Peñalosa JM, Manzano R, Carpena–Ruiz RO, Gamarra R, Esteban E. Heavy metals distribution in soils surrounding an abandoned mine in NW Madrid (Spain) and their transference to wild flora. *J. Hazard. Mater.* **2009**, *162*, 854–859.
309. Tsuchiya Y, Shimogaki H, Abe H, Kagawa A. Inorganic elements in typical Japanese trees for woody biomass fuel. *Wood Sci.* **2010**, *56*, 53–63.
310. Tsuchiya Y, Shimogaki H, Abe H, Kagawa A. Inorganic elements in typical Japanese trees for woody biomass fuel. *Wood Sci.* **2010**, *56*, 53–63.
311. Tsuchiya Y, Shimogaki H, Abe H, Kagawa A. Inorganic elements in typical Japanese trees for woody biomass fuel. *Wood Sci.* **2010**, *56*, 53–63
312. Gülser F, Çığ A, Sönmez F. The Determination of Phytoremediation Levels of Ornamental Plants Used in Landscape. J. Int. Environmental Application & Science. **2011**, *6*, 661–667.
313. Mayer AM, Gorham E. The Iron and Manganese Content of Plants present in the Natural Vegetation of the English Lake District. *Ann. Bot.* **1951**, *15*, 247–263.
314. Mayer AM, Gorham E. The Iron and Manganese Content of Plants present in the Natural Vegetation of the English Lake District. *Ann. Bot.* **1951**, *15*, 247–263.
315. Reimann C, Arnoldussen A, Boyd R, Finne TE, Koller F, Nordgulen O, Englmaier P. Element contents in leaves of four plant species (birch, mountain ash, fern and spruce) along anthropogenic and geogenic concentration gradients. Science of the Total Environment. **2007**, *377*, 416–433.
316. Reimann C, Arnoldussen A, Boyd R, Finne TE, Koller F, Nordgulen O, Englmaier P. Element contents in leaves of four plant species (birch, mountain ash, fern and spruce) along anthropogenic and geogenic concentration gradients. Science of the Total Environment. **2007**, *377*, 416–433.
317. de la Fuente V, Rufo L, Rodríguez N, Amils R, Zuluaga J. Metal accumulation screening of the Río Tinto flora (Huelva, Spain). Biol Trace Elem Res. **2010**, *134*, 318–41.
318. Memon AR, Itô S, Yatazawa M. Absorption and accumulation of iron, manganese and copper in plants in the temperate forest of central Japan. Soil Sci. Plant Nutr. **1979**, *15*, 611–620.
319. Olivares E, Pena E, Marcano E, Mostacero J, Aguiar G, Benitez M, Rengifo E. Aluminum accumulation and its relationship with mineral plant nutrients in 12 pteridophytes from Venezuela. *Environ. Exp. Bot.* **2009**, *65*, 132–141.
320. Osaki M, Watanabe T, Ishizawa T, Nilnond C, Nuyim T, Sittibush C, Tadano T. Nutritional characteristics in leaves of native plants grown in acid sulfate, peat, sandy podzolic, and saline soils distributed in Peninsular Thailand. Plant and Soil. **1998**, *201*, 175–182.
321. Mayer AM, Gorham E. The Iron and Manganese Content of Plants present in the Natural Vegetation of the English Lake District. *Ann. Bot.* **1951**, *15*, 247–263.
322. Kuria SG, IA Tura, S Amboga, HK Walaga, 2012. Assessment of status of minerals in forages preferred by camels (Camelus dromedarius) in the Arid North Eastern Kenya. *Res. Opin. Anim. Vet. Sci.* **2012**, *2*, 173–180.
323. Abolaji OA, Adebayo AH, Odesanmi OS. Nutritional Qualities of Three Medicinal Plant Parts (Xylopia aethiopica, Blighia sapida and Parinari polyandra) commonly used by Pregnant Women in the Western Part of Nigeria. *Pak. J. Nutr.* **2007**, *6*, 665–668.
324. Rode KD, Chapman CA, Chapman LJ, McDowell LR. Mineral Resource Availability and Consumption by Colobus in Kibale National Park, Uganda. *Int. J. Primatol*. **2003**, *24*, 541–573.
325. Brahma J, Singh B, Rethy P, Gajurel P. Nutritional Analysis Of Some Selected Wild Edible Species Consumed By The Bodos Tribes Of Kokrajhar District, BTC, Assam. Asian J Pharm Clin Res. **2014**, *7*, 34–37.
326. Olobanji SO, Adebajo AC, Omobuwajo OR, Ceccato D, Buoso MC, Moschini G. PIXE analysis of some Nigerian anti–diabetic medicinal plants (II). Nuclear Instruments and Methods in Physics Research B. **2014**, *318*, 187–190.
327. Madejón P, Murillo JM, Marañón T, Espinar JL, Cabrera F. Accumulation of As, Cd and selected trace elements in tubers of Scirpus maritimus L. from Doñana marshes (South Spain). Chemosphere. **2006**, *64*, 742–748.
328. dos Santos UM, de Carvalho Gonçalvesa JF, Feldpausch TR. Growth, leaf nutrient concentration and photosynthetic nutrient use efficiency in tropical tree species planted in degraded areas in central Amazonia. Forest Ecology and Management. **2006**, *226*, 299–309.
329. Chizzola R, Michitsch H, Franz C. Monitoring of metallic micronutrients and heavy metals in herbs, spices and medicinal plants from Austria. Eur Food Res Technol. **2003**, *216*, 407–411.
330. Lockett CT, Calvert CC, Grivetti LE. Energy and micronutrient composition of dietary and medicinal wild plants consumed during drought. Study of rural Fulani, northeastern Nigeria. International Journal of Food Sciences and Nutrition; May **2000**, *51*, 195–208.
331. Kuria SG, IA Tura, S Amboga, HK Walaga, 2012. Assessment of status of minerals in forages preferred by camels (Camelus dromedarius) in the Arid North Eastern Kenya. Res. Opin. Anim. Vet. Sci. **2012**, *2*, 173–180.
332. Kuria SG, IA Tura, S Amboga, HK Walaga, 2012. Assessment of status of minerals in forages preferred by camels (Camelus dromedarius) in the Arid North Eastern Kenya. Res. Opin. Anim. Vet. Sci. **2012**, *2*, 173–180.
333. Kuria SG, IA Tura, S Amboga, HK Walaga, 2012. Assessment of status of minerals in forages preferred by camels (Camelus dromedarius) in the Arid North Eastern Kenya. Res. Opin. Anim. Vet. Sci. **2012**, *2*, 173–180.
334. Kuria SG, IA Tura, S Amboga, HK Walaga, 2012. Assessment of status of minerals in forages preferred by camels (Camelus dromedarius) in the Arid North Eastern Kenya. Res. Opin. Anim. Vet. Sci. **2012**, *2*, 173–180.
335. Rajendran D, Balakrishnan V. Diet Composition, Biomass Yield and Mineral Contents of Vegetation in Native Tract of Mecheri Sheep. Animal Nutrition and Feed Technology. **2012**, *12*, 63–71.
336. Rajendran D, Balakrishnan V. Diet Composition, Biomass Yield and Mineral Contents of Vegetation in Native Tract of Mecheri Sheep. Animal Nutrition and Feed Technology. **2012**, *12*, 63–71.
337. Rajendran D, Balakrishnan V. Diet Composition, Biomass Yield and Mineral Contents of Vegetation in Native Tract of Mecheri Sheep. Animal Nutrition and Feed Technology. **2012**, *12*, 63–71.
338. Rajendran D, Balakrishnan V. Diet Composition, Biomass Yield and Mineral Contents of Vegetation in Native Tract of Mecheri Sheep. Animal Nutrition and Feed Technology. **2012**, *12*, 63–71.
339. Mayer AM, Gorham E. The Iron and Manganese Content of Plants present in the Natural Vegetation of the English Lake District. *Ann. Bot.* **1951**, *15*, 247–263.
340. Mayer AM, Gorham E. The Iron and Manganese Content of Plants present in the Natural Vegetation of the English Lake District. *Ann. Bot.* **1951**, *15*, 247–263.
341. Mayer AM, Gorham E. The Iron and Manganese Content of Plants present in the Natural Vegetation of the English Lake District. *Ann. Bot.* **1951**, *15*, 247–263.
342. Bolanle AO, Funmilola AS, Adedayo A. Proximate Analysis, Mineral Contents, Amino Acid Composition, Anti–Nutrients and Phytochemical Screening of Brachystegia Eurycoma Harms and Pipper Guineense Schum and Thonn. American Journal of Food and Nutrition. **2014**, *2*, 11–17.
343. Duke JA, Ayensu ES. Medicinal Plants of China. Available online: http://www.pfaf.org/user/Plant.aspx?LatinName=Brasenia+schreberi [23.07.2014].
344. Karmakar K, Muslim T, Rahman MA. Chemical Composition of Some Leafy Vegetables of Bangladesh. Dhaka Univ. J. Sci. **2013**, *61*, 199–201.
345. Karmakar K, Muslim T, Rahman MA. Chemical Composition of Some Leafy Vegetables of Bangladesh. Dhaka Univ. J. Sci. **2013**, *61*, 199–201.
346. Seal T, Pillai B, Chaudhuri K. Nutritive Value and Mineral Composition of Some Wild Edible Plants from Meghalaya State in India. *Adv. Biol. Res.* **2014**, *8*, 116–122.
347. Duke JA, Ayensu ES. Medicinal Plants of China. Available online: http://www.pfaf.org/user/
     Plant.aspx?LatinName=Brassica+oleracea+gongylodes [23.07.2014].
348. Duke JA, Ayensu ES. Medicinal Plants of China. Available online: http://www.pfaf.org/user/
     plant.aspx?LatinName=Brassica+oleracea [23.07.2014].
349. Ali MHH, Al–Quahtani K. Assessment of some heavy metals in vegetables, cereals and fruits in Saudi Arabian markets. The Egyptian Journal of Aquatic Research. **2012**, *38*, 31–37.
350. Pillay V, Jonnalagadda SB. Elemental uptake by edible herbs and lettuce (Latuca sativa). Journal of Environmental Science and Health Part B. **2007**, *42*, 423–428.
351. Emebu PK, Anyika JU. Proximate and Mineral Composition of Kale (Brassica oleracea) Grown in Delta State, Nigeria. Pakistan Journal of Nutrition. **2011**, *10*, 190–194.
352. Wu J, Schat H, Sun R, Koornneef M, Wang X, Aarts MGM. Characterization of natural variation for zinc, iron and manganese accumulation and zinc exposure response in Brassica rapa L. Plant Soil. **2007**, *291*, 167–180.
353. Karmakar K, Muslim T, Rahman MA. Chemical Composition of Some Leafy Vegetables of Bangladesh. Dhaka Univ. J. Sci. **2013**, *61*, 199–201.
354. Duke JA, Ayensu ES. Medicinal Plants of China. Available online: http://www.pfaf.org/user/
     plant.aspx?LatinName=Brassica+rapa [23.07.2014].
355. Wu J, Schat H, Sun R, Koornneef M, Wang X, Aarts MGM. Characterization of natural variation for zinc, iron and manganese accumulation and zinc exposure response in Brassica rapa L. Plant Soil. **2007**, *291*, 167–180.
356. Ali MHH, Al–Quahtani K. Assessment of some heavy metals in vegetables, cereals and fruits in Saudi Arabian markets. The Egyptian Journal of Aquatic Research. **2012**, *38*, 31–37.
357. Animal Nutrition Group, National Dairy Development Board. Nutritive value of commonly available feeds and fodders in India, 2012. Available online:: http://www.nddb.coop/English/Services/AN/
     Documents/Animal–Nutrition–booklet.pdf [09.02.2014].
358. Lockett CT, Calvert CC, Grivetti LE. Energy and micronutrient composition of dietary and medicinal wild plants consumed during drought. Study of rural Fulani, northeastern Nigeria. International Journal of Food Sciences and Nutrition; May **2000**, *51*, 195–208.
359. Wan K, Chen F, Tao Y, Chen S. Nutrient Elements in Leaves of Rare and Endangered Species in Wuhan Botanical Garden, China. *J. Plant. Nutr.* **2009**, *32*, 1914–1940.
360. Lockett CT, Calvert CC, Grivetti LE. Energy and micronutrient composition of dietary and medicinal wild plants consumed during drought. Study of rural Fulani, northeastern Nigeria. International Journal of Food Sciences and Nutrition; May **2000**, *51*, 195–208.
361. Olobanji SO, Adebajo AC, Omobuwajo OR, Ceccato D, Buoso MC, Moschini G. PIXE analysis of some Nigerian anti–diabetic medicinal plants (II). Nuclear Instruments and Methods in Physics Research B. **2014**, *318*, 187–190.
362. Rode KD, Chapman CA, Chapman LJ, McDowell LR. Mineral Resource Availability and Consumption by Colobus in Kibale National Park, Uganda. *Int. J. Primatol*. **2003**, *24*, 541–573.
363. Rode KD, Chapman CA, Chapman LJ, McDowell LR. Mineral Resource Availability and Consumption by Colobus in Kibale National Park, Uganda. *Int. J. Primatol*. **2003**, *24*, 541–573.
364. de la Fuente V, Rufo L, Rodríguez N, Amils R, Zuluaga J. Metal accumulation screening of the Río Tinto flora (Huelva, Spain). Biol Trace Elem Res. **2010**, *134*, 318–41.
365. Adarve MJ, Hernández AJ, Gil A, Pasto J. Boron, Zinc, Iron, and Manganese Content in Four Grassland Species. Journal of Environmental Quality. 1998; 27 : 1286–1293.
366. Adarve MJ, Hernández AJ, Gil A, Pasto J. Boron, Zinc, Iron, and Manganese Content in Four Grassland Species. Journal of Environmental Quality. 1998; 27 : 1286–1293.
367. Tanji A, Elgharous M. A survey of mineral composition of weed seeds. Weed Research. 1998; 38 : 79–86
368. Liu Y, Ding H，Zhu Y. Metal bioaccumulation in plant leaves from an industrious area and the Botanical Garden in Beijing. Journal of Environmental Sciences. **2005**, *17*, 294–300.
369. Liu Y, Ding H，Zhu Y. Metal bioaccumulation in plant leaves from an industrious area and the Botanical Garden in Beijing. Journal of Environmental Sciences. **2005**, *17*, 294–300.
370. Tsuchiya Y, Shimogaki H, Abe H, Kagawa A. Inorganic elements in typical Japanese trees for woody biomass fuel. *Wood Sci.* **2010**, *56*, 53–63.
371. Tsuchiya Y, Shimogaki H, Abe H, Kagawa A. Inorganic elements in typical Japanese trees for woody biomass fuel. *Wood Sci.* **2010**, *56*, 53–63.
372. Tsuchiya Y, Shimogaki H, Abe H, Kagawa A. Inorganic elements in typical Japanese trees for woody biomass fuel. *Wood Sci.* **2010**, *56*, 53–63.
373. Olobanji SO, Adebajo AC, Omobuwajo OR, Ceccato D, Buoso MC, Moschini G. PIXE analysis of some Nigerian anti–diabetic medicinal plants (II). Nuclear Instruments and Methods in Physics Research B. **2014**, *318*, 187–190.
374. Ugulu I, Dogan Y, Baslar S, Varol O. Biomonitoring of trace element accumulation in plants growing at Murat Mountain. Int. J. Environ. Sci. Technol. **2012**, *9*, 527–534.
375. Kashin VK. Vitally Important Microelements in Transbaikalian Herbs. *Chem. Sustain. Dev.* **2009**, *17*, 371–381.
376. Kashin VK. Vitally Important Microelements in Transbaikalian Herbs. *Chem. Sustain. Dev.* **2009**, *17*, 371–381.
377. Ugulu I, Dogan Y, Baslar S, Varol O. Biomonitoring of trace element accumulation in plants growing at Murat Mountain. Int. J. Environ. Sci. Technol. **2012**, *9*, 527–534.
378. Kuria SG, IA Tura, S Amboga, HK Walaga, 2012. Assessment of status of minerals in forages preferred by camels (Camelus dromedarius) in the Arid North Eastern Kenya. Res. Opin. Anim. Vet. Sci. **2012**, *2*, 173–180.
379. Kuria SG, IA Tura, S Amboga, HK Walaga, 2012. Assessment of status of minerals in forages preferred by camels (Camelus dromedarius) in the Arid North Eastern Kenya. Res. Opin. Anim. Vet. Sci. **2012**, *2*, 173–180.
380. Yusuf AA, Mofio BM, Ahmed AB. Nutrient Contents of Pride of Barbados (Caesalpinia pulcherima Linn.) Seeds. Pakistan Journal of Nutrition. **2007**, *6*, 117–121.
381. Mayer AM, Gorham E. The Iron and Manganese Content of Plants present in the Natural Vegetation of the English Lake District. *Ann. Bot.* **1951**, *15*, 247–263.
382. Tanji A, Elgharous M. A survey of mineral composition of weed seeds. Weed Research. **1998**, *38*, 79–86.
383. Chizzola R, Michitsch H, Franz C. Monitoring of metallic micronutrients and heavy metals in herbs, spices and medicinal plants from Austria. Eur Food Res Technol. **2003**, *216*, 407–411.
384. Chizzola R, Michitsch H, Franz C. Monitoring of metallic micronutrients and heavy metals in herbs, spices and medicinal plants from Austria. Eur Food Res Technol. **2003**, *216*, 407–411.
385. Franco MJ, Caetano ICS, Caetano J, Dragunski DC. Determinação de Metais em Plantas Medicinais Comercializadas na Região de Umuarama–PR. Arq. Ciênc. Saúde UNIPAR, Umuarama, **2011**, *15*, 121–127.
386. Franco MJ, Caetano ICS, Caetano J, Dragunski DC. Determinação de Metais em Plantas Medicinais Comercializadas na Região de Umuarama–PR. Arq. Ciênc. Saúde UNIPAR, Umuarama, **2011**, *15*, 121–127.
387. Linn JG, Staba EJ, Goodrich RD, Meiske JC, Otterby DE. Nutritive Value of Dried or Ensiled Aquatic Plants. I. Chemical Composition. *J. Anim. Sci.* **1975**, *41*, 601–609.
388. Guerrero–Cervantes M, Ramírez RG, González–Rodríguez H, Cerrillo–Soto A, Juárez–Réyes A. Mineral content in range forages from north Mexico. J. Appl. Anim. Res. **2012**, *40*, 102–107.
389. Memon AR, Itô S, Yatazawa M. Absorption and accumulation of iron, manganese and copper in plants in the temperate forest of central Japan. Soil Sci. Plant Nutr. **1979**, *15*, 611–620.
390. Mayer AM, Gorham E. The Iron and Manganese Content of Plants present in the Natural Vegetation of the English Lake District. *Ann. Bot.* **1951**, *15*, 247–263.
391. Mayer AM, Gorham E. The Iron and Manganese Content of Plants present in the Natural Vegetation of the English Lake District. *Ann. Bot.* **1951**, *15*, 247–263.
392. Mayer AM, Gorham E. The Iron and Manganese Content of Plants present in the Natural Vegetation of the English Lake District. *Ann. Bot.* **1951**, *15*, 247–263.
393. Wan K, Chen F, Tao Y, Chen S. Nutrient Elements in Leaves of Rare and Endangered Species in Wuhan Botanical Garden, China. *J. Plant. Nutr.* **2009**, *32*, 1914–1940.
394. Frame DD, Palmer M, Peterson B. Use of Camelina sativa in the Diets of Young Turkeys. J. Appl. Poult. Res. **2007**, *16*, 381–386
395. Zubr J. Carbohydrates, vitamins and minerals of Camelina sativa seed. Nutrition & Food Science. **2010**, *40*, 523–531.
396. Toncea I, Necseriu D, Prisecaru T, Balint LN, Ghilvacs MI, Popa M. The seed’s and oil composition of Camelia–first romanian cultivar of camelina (Camelina sativa, L. Crantz). Romanian Biotechnological Letters. **2013**, *18*, 8594–8602.
397. Wan K, Chen F, Tao Y, Chen S. Nutrient Elements in Leaves of Rare and Endangered Species in Wuhan Botanical Garden, China. *J. Plant. Nutr.* 2009; 32 : 1914–1940.
398. Wan K, Chen F, Tao Y, Chen S. Nutrient Elements in Leaves of Rare and Endangered Species in Wuhan Botanical Garden, China. *J. Plant. Nutr.* 2009; 32 : 1914–1940.
399. Gallaher RN, Gallaher K, Marshall AJ, Marshall AC. Mineral analysis of ten types of commercially available tea. Journal of Food Composition and Analysis. 2006; 19: S53–S57.
400. Gallaher RN, Gallaher K, Marshall AJ, Marshall AC. Mineral analysis of ten types of commercially available tea. Journal of Food Composition and Analysis. 2006; 19: S53–S57.
401. Wan K, Chen F, Tao Y, Chen S. Nutrient Elements in Leaves of Rare and Endangered Species in Wuhan Botanical Garden, China. *J. Plant. Nutr.* **2009**, *32*, 1914–1940.
402. Schmidt DA, Iambana RB, Britt A, Junge RE, Welch CR, Porton IJ, Kerley MS. Nutrient composition of plants consumed by black and white ruffed lemurs, Varecia variegata, in the Betampona Natural Reserve, Madagascar. *Zoo Biol.* **2010**, *29*, 375–396.
403. Chew LY, Nagendra Prasad K, Amin I, Azrinaa A, Lauc CY. Nutritional composition and antioxidant properties of Canarium odontophyllum Miq. (dabai) fruits. Journal of Food Composition and Analysis. **2011**, *24*, 670–677.
404. Chew LY, Nagendra Prasad K, Amin I, Azrinaa A, Lauc CY. Nutritional composition and antioxidant properties of Canarium odontophyllum Miq. (dabai) fruits. Journal of Food Composition and Analysis. **2011**, *24*, 670–677.
405. Thangadurai D, Viswanathan MB, Ramesh N. The chemical composition and nutritional evaluation of Canavalia virosa: a wild perennial bean from Eastern Ghats of Peninsular India. Eur Food Res Technol. **2001**, *213*, 456–459.
406. Ozcan MM, Akbulut M. Estimation of minerals, nitrate and nitrite contents of medicinal and aromatic plants used as spices, condiments and herbal tea. Food Chem. 2008; 106 : 852–858.
407. Duke JA, Ayensu ES. Medicinal Plants of China. Available online: http://www.pfaf.org/user/
     Plant.aspx?LatinName=Capsella+bursa–pastoris [18.07.2014].
408. Soylak M, Cihan Z, Yilmaz E. Evaluation of trace element contents of some herbal plants and spices retailed in Kayseri, Turkey. Environ Monit Assess. **2012**, *184*, 3455–3461.
409. Ansari TM, Ikram N, Najam–ul–Haq M, Fayyaz Y, Fayyaz Q, Ghafoor I, Khalid N. Essential Trace Metal (Zinc, Manganese, Copper and Iron) Levels in Plants of Medicinal Importance. Journal of Biological Sciences. **2004**, *4*, 95–99.
410. Ozcan MM, Akbulut M. Estimation of minerals, nitrate and nitrite contents of medicinal and aromatic plants used as spices, condiments and herbal tea. Food Chem. **2008**, *106*, 852–858.
411. Carvajal M, Martinez–Sanchez F, Pastor JJ, Alcaraz CF. Leaf spray with Ti(IV) ascorbate improves the iron uptake and iron activity in Capsicum annuum L. plants. In : Iron nutrition in soil and plants. J. Abadia (Ed.). Springer Science + Business Media, Dordrecht, 1995, 1–5.
412. Carvajal M, Martinez–Sanchez F, Pastor JJ, Alcaraz CF. Leaf spray with Ti(IV) ascorbate improves the iron uptake and iron activity in Capsicum annuum L. plants. In : Iron nutrition in soil and plants. J. Abadia (Ed.). Springer Science + Business Media, Dordrecht, 1995, 1–5.
413. Rode KD, Chapman CA, Chapman LJ, McDowell LR. Mineral Resource Availability and Consumption by Colobus in Kibale National Park, Uganda. *Int. J. Primatol*. **2003**, *24*, 541–573.
414. Ernst WHO. Element Nutrition of Two Contrasted Dune Annuals. Journal of Ecology. **1983**, *71*, 197–209.
415. Mayer AM, Gorham E. The Iron and Manganese Content of Plants present in the Natural Vegetation of the English Lake District. *Ann. Bot.* **1951**, *15*, 247–263.
416. Mayer AM, Gorham E. The Iron and Manganese Content of Plants present in the Natural Vegetation of the English Lake District. *Ann. Bot.* **1951**, *15*, 247–263.
417. de la Fuente V, Rufo L, Rodríguez N, Amils R, Zuluaga J. Metal accumulation screening of the Río Tinto flora (Huelva, Spain). Biol Trace Elem Res. **2010**, *134*, 318–41.
418. Linn JG, Staba EJ, Goodrich RD, Meiske JC, Otterby DE. Nutritive Value of Dried or Ensiled Aquatic Plants. I. Chemical Composition. *J. Anim. Sci.* **1975**, *41*, 601–609.
419. Mayer AM, Gorham E. The Iron and Manganese Content of Plants present in the Natural Vegetation of the English Lake District. *Ann. Bot.* **1951**, *15*, 247–263.
420. Linn JG, Staba EJ, Goodrich RD, Meiske JC, Otterby DE. Nutritive Value of Dried or Ensiled Aquatic Plants. I. Chemical Composition. *J. Anim. Sci.* **1975**, *41*, 601–609.
421. Mayer AM, Gorham E. The Iron and Manganese Content of Plants present in the Natural Vegetation of the English Lake District. *Ann. Bot.* **1951**, *15*, 247–263.
422. Mayer AM, Gorham E. The Iron and Manganese Content of Plants present in the Natural Vegetation of the English Lake District. *Ann. Bot.* **1951**, *15*, 247–263.
423. Seal T, Pillai B, Chaudhuri K. Nutritive Value and Mineral Composition of Some Wild Edible Plants from Meghalaya State in India. *Adv. Biol. Res.* **2014**, *8*, 116–122.
424. Olobanji SO, Adebajo AC, Omobuwajo OR, Ceccato D, Buoso MC, Moschini G. PIXE analysis of some Nigerian anti–diabetic medicinal plants (II). Nuclear Instruments and Methods in Physics Research B. **2014**, *318*, 187–190.
425. Memon AR, Itô S, Yatazawa M. Absorption and accumulation of iron, manganese and copper in plants in the temperate forest of central Japan. Soil Sci. Plant Nutr. **1979**, *15*, 611–620.
426. Memon AR, Itô S, Yatazawa M. Absorption and accumulation of iron, manganese and copper in plants in the temperate forest of central Japan. Soil Sci. Plant Nutr. **1979**, *15*, 611–620.
427. Memon AR, Itô S, Yatazawa M. Absorption and accumulation of iron, manganese and copper in plants in the temperate forest of central Japan. Soil Sci. Plant Nutr. **1979**, *15*, 611–620.
428. Wan K, Chen F, Tao Y, Chen S. Nutrient Elements in Leaves of Rare and Endangered Species in Wuhan Botanical Garden, China. *J. Plant. Nutr.* **2009**, *32*, 1914–1940.
429. Memon AR, Itô S, Yatazawa M. Absorption and accumulation of iron, manganese and copper in plants in the temperate forest of central Japan. Soil Sci. Plant Nutr. **1979**, *15*, 611–620
430. Vishwakarma KL, Dubey V. Nutritional analysis of indigenous wild edible herbs used in eastern Chhattisgarh, India. *Emir. J. Food Agric.* **2011**, *23*, 554–560.
431. Kashin VK. Vitally Important Microelements in Transbaikalian Herbs. *Chem. Sustain. Dev.* **2009**, *17*, 371–381.
432. Chizzola R, Michitsch H, Franz C. Monitoring of metallic micronutrients and heavy metals in herbs, spices and medicinal plants from Austria. Eur Food Res Technol. **2003**, *216*, 407–411.
433. Chizzola R, Michitsch H, Franz C. Monitoring of metallic micronutrients and heavy metals in herbs, spices and medicinal plants from Austria. Eur Food Res Technol. **2003**, *216*, 407–411.
434. ***. Plants for a future. Available online: http://www.pfaf.org/user/Plant.aspx?LatinName=Carum+carvi [23.07.2014].
435. Dini I, Tenore GC, Dini A. Chemical composition, nutritional value and antioxidant properties of Allium caepa L. Var. tropeana (red onion) seeds. Food Chemistry. **2008**, *107*, 613–621.
436. Schmidt DA, Iambana RB, Britt A, Junge RE, Welch CR, Porton IJ, Kerley MS. Nutrient composition of plants consumed by black and white ruffed lemurs, Varecia variegata, in the Betampona Natural Reserve, Madagascar. *Zoo Biol.* **2010**, *29*, 375–96
437. Ansari TM, Ikram N, Najam–ul–Haq M, Fayyaz Y, Fayyaz Q, Ghafoor I, Khalid N. Essential Trace Metal (Zinc, Manganese, Copper and Iron) Levels in Plants of Medicinal Importance. Journal of Biological Sciences. **2004**, *4*, 95–99
438. Borah S, Baruah AM, Das AK, Borah J. Determination of Mineral Content in Commonly Consumed Leafy Vegetables. Food Anal. Methods. 2009; 2:226–230
439. Nkafamiya II, Manji AJ, Modibbo UU, Umaru HA. Biochemical evaluation of Cassipourea congoensis (Tunti) and Nuclea latifolia (Luzzi) fruits. African Journal of Biotechnology. **2006**, *6*, 2461–2463
440. Rode KD, Chapman CA, Chapman LJ, McDowell LR. Mineral Resource Availability and Consumption by Colobus in Kibale National Park, Uganda. *Int. J. Primatol*. **2003**, *24*, 541–573.
441. Rode KD, Chapman CA, Chapman LJ, McDowell LR. Mineral Resource Availability and Consumption by Colobus in Kibale National Park, Uganda. *Int. J. Primatol*. **2003**, *24*, 541–573.
442. Tsuchiya Y, Shimogaki H, Abe H, Kagawa A. Inorganic elements in typical Japanese trees for woody biomass fuel. *Wood Sci.* **2010**, *56*, 53–63
443. Tsuchiya Y, Shimogaki H, Abe H, Kagawa A. Inorganic elements in typical Japanese trees for woody biomass fuel. *Wood Sci.* **2010**, *56*, 53–63
444. Memon AR, Itô S, Yatazawa M. Absorption and accumulation of iron, manganese and copper in plants in the temperate forest of central Japan. Soil Sci. Plant Nutr. **1979**, *15*, 611–620
445. Tsuchiya Y, Shimogaki H, Abe H, Kagawa A. Inorganic elements in typical Japanese trees for woody biomass fuel. *Wood Sci.* **2010**, *56*, 53–63
446. Hafızoglu H, Sivrikaya H, Bacak L. Inorganic constituents in barks of Abies bornmulleriana and Castanea sativa. Holz als Roh– und Werkstoff. **2006**, *64*, 247–249
447. Mayer AM, Gorham E. The Iron and Manganese Content of Plants present in the Natural Vegetation of the English Lake District. *Ann. Bot.* **1951**, *15*, 247–263
448. Mayer AM, Gorham E. The Iron and Manganese Content of Plants present in the Natural Vegetation of the English Lake District. *Ann. Bot.* **1951**, *15*, 247–263
449. Singh N. Wild edible plants: a potential source of nutraceuticals. International Journal of Pharma Sciences and Research (IJPSR). **2011**, *2*, 216–225
450. Osaki M, Watanabe T, Ishizawa T, Nilnond C, Nuyim T, Sittibush C, Tadano T. Nutritional characteristics in leaves of native plants grown in acid sulfate, peat, sandy podzolic, and saline soils distributed in Peninsular Thailand. Plant and Soil. **1998**, *201*, 175–182.
451. Brahma J, Singh B, Rethy P, Gajurel P. Nutritional Analysis of Some Selected Wild Edible Species Consumed By The Bodos Tribes Of Kokrajhar District, BTC, Assam. *Asian J. Pharm. Clin. Res.* **2014**, *7*,
     34–37.
452. dos Santos UM, de Carvalho Gonçalvesa JF, Feldpausch TR. Growth, leaf nutrient concentration and photosynthetic nutrient use efficiency in tropical tree species planted in degraded areas in central Amazonia. *For. Ecol. Manag*. **2006**, *226*, 299–309
453. dos Santos UM, de Carvalho Gonçalvesa JF, Feldpausch TR. Growth, leaf nutrient concentration and photosynthetic nutrient use efficiency in tropical tree species planted in degraded areas in central Amazonia. Forest Ecology and Management. **2006**, *226*, 299–309
454. Rucandio MI, Petit–Domínguez MD, Fidalgo–Hijano C, García–Giménez R. Biomonitoring of chemical elements in an urban environment using arboreal and bush plant species. *Environ. Sci. Pollut. Res*. **2011**, *18*, 51–63
455. Ugulu I, Dogan Y, Baslar S, Varol O. Biomonitoring of trace element accumulation in plants growing at Murat Mountain. *Int. J. Environ. Sci. Technol*. **2012**, *9*, 527–534
456. Gülser F, Çığ A, Sönmez F. The Determination of Phytoremediation Levels of Ornamental Plants Used in Landscape. *J. Int. Environ. Appl. Sci.* **2011**, *6*, 661–667
457. Patricia O, Zoue L, Megnanou RM, Doue R, Niamke S. Proximate Composition And Nutritive Value Of Leafy Vegetables Consumed In Northern Côte d’Ivoire. European Scientific Journal. **2014**, *10*, 212–227
458. Kumar A, Singh RP, Singh NP. Analysis of macro and micro nutrients in some Indian medicinal herbs grown in Jaunpur (u.p.) soil. *Nat. Sci.* **2011**, *3*, 551–555
459. Zoro, A.F.; Zoue, L.T.; Kra, S.A.K.; Yepie, A.E.; Niamke, S.L. An Overview of Nutritive Potential of Leafy Vegetables Consumed in Western Côte d’Ivoire. *Pak. J. Nutr.* **2013**, *12*, 949–956
460. Olivares E, Pena E, Marcano E, Mostacero J, Aguiar G, Benitez M, Rengifo E. Aluminum accumulation and its relationship with mineral plant nutrients in 12 pteridophytes from Venezuela. *Environ. Exp. Bot*. **2009**, *65*, 132–141
461. Rode KD, Chapman CA, Chapman LJ, McDowell LR. Mineral Resource Availability and Consumption by Colobus in Kibale National Park, Uganda. *Int. J. Primatol*. **2003**, *24*, 541–573.
462. Rode KD, Chapman CA, Chapman LJ, McDowell LR. Mineral Resource Availability and Consumption by Colobus in Kibale National Park, Uganda. *Int. J. Primatol*. **2003**, *24*, 541–573.
463. Ugulu I, Dogan Y, Baslar S, Varol O. Biomonitoring of trace element accumulation in plants growing at Murat Mountain. Int. J. Environ. Sci. Technol. **2012**, *9*, 527–534
464. Rode KD, Chapman CA, Chapman LJ, McDowell LR. Mineral Resource Availability and Consumption by Colobus in Kibale National Park, Uganda. *Int. J. Primatol*. **2003**, *24*, 541–573.
465. Rode KD, Chapman CA, Chapman LJ, McDowell LR. Mineral Resource Availability and Consumption by Colobus in Kibale National Park, Uganda. *Int. J. Primatol*. **2003**, *24*, 541–573.
466. Rode KD, Chapman CA, Chapman LJ, McDowell LR. Mineral Resource Availability and Consumption by Colobus in Kibale National Park, Uganda. *Int. J. Primatol*. **2003**, *24*, 541–573.
467. Rode KD, Chapman CA, Chapman LJ, McDowell LR. Mineral Resource Availability and Consumption by Colobus in Kibale National Park, Uganda. *Int. J. Primatol*. **2003**, *24*, 541–573.
468. Rode KD, Chapman CA, Chapman LJ, McDowell LR. Mineral Resource Availability and Consumption by Colobus in Kibale National Park, Uganda. *Int. J. Primatol*. **2003**, *24*, 541–573.
469. Rode KD, Chapman CA, Chapman LJ, McDowell LR. Mineral Resource Availability and Consumption by Colobus in Kibale National Park, Uganda. *Int. J. Primatol*. **2003**, *24*, 541–573.
470. Rode KD, Chapman CA, Chapman LJ, McDowell LR. Mineral Resource Availability and Consumption by Colobus in Kibale National Park, Uganda. *Int. J. Primatol*. **2003**, *24*, 541–573.
471. Guerrero–Cervantes M, Ramírez RG, González–Rodríguez H, Cerrillo–Soto A, Juárez–Réyes A. Mineral content in range forages from north Mexico. *J. Appl. Anim. Res.* **2012**, *40*, 102–107.
472. Tanji A, Elgharous M. A survey of mineral composition of weed seeds. Weed Research. **1998**, *38*, 79–86.
473. de la Fuente V, Rufo L, Rodríguez N, Amils R, Zuluaga J. Metal accumulation screening of the Río Tinto flora (Huelva, Spain). Biol Trace Elem Res. **2010**, *134*, 318–341.
474. Moreno–Jiménez E, Peñalosa JM, Manzano R, Carpena–Ruiz RO, Gamarra R, Esteban E. Heavy metals distribution in soils surrounding an abandoned mine in NW Madrid (Spain) and their transference to wild flora. *J. Hazard. Mater.* **2009**, *162*, 854–859.
475. Moreno–Jiménez E, Peñalosa JM, Manzano R, Carpena–Ruiz RO, Gamarra R, Esteban E. Heavy metals distribution in soils surrounding an abandoned mine in NW Madrid (Spain) and their transference to wild flora *J. Hazard. Mater.* **2009**, *162*, 854–859.
476. Malayeri BE, Chehregani A, Yousefi N, Lorestani B. Identification of the Hyper Acumulator Plants in Copper and Iron Mine in Iran. Pakistan Journal of Biological Sciences. **2008**, *11*, 490–492.
477. Dodson HG. Total iron in selected genotypes of spinach and cowpeas and the in vitro iron bioavailability of selected spinach genotypes. Thesis, University of Arkansas, 2008. Available online: http://books.google.ro/books?id=a11YrZXUJEYC&pg=PA65&lpg=PA65&dq=Total+Iron+in+Selected+Genotypes+of+Spinach+and+Cowpeas+and+the+in+Vitro+dissertation&source=bl&ots=cf4tjiZxke&sig=jn4pjk78t6mfXY2vR3lzOWDVpGU&hl=ro&sa=X&ei=YgB2U4elOqqu7Aa8ioCoCg&ved=0CDYQ6AEwAQ#v=onepage&q=Total%20Iron%20in%20Selected%20Genotypes%20of%20Spinach%20and%20Cowpeas%20and%20the%20in%20Vitro%20dissertation&f=false [16.05.2014].
478. Dodson HG. Total iron in selected genotypes of spinach and cowpeas and the in vitro iron bioavailability of selected spinach genotypes. Thesis, University of Arkansas, 2008. Available online: http://books.google.ro/books?id=a11YrZXUJEYC&pg=PA65&lpg=PA65&dq=Total+Iron+in+Selected+Genotypes+of+Spinach+and+Cowpeas+and+the+in+Vitro+dissertation&source=bl&ots=cf4tjiZxke&sig=jn4pjk78t6mfXY2vR3lzOWDVpGU&hl=ro&sa=X&ei=YgB2U4elOqqu7Aa8ioCoCg&ved=0CDYQ6AEwAQ#v=onepage&q=Total%20Iron%20in%20Selected%20Genotypes%20of%20Spinach%20and%20Cowpeas%20and%20the%20in%20Vitro%20dissertation&f=false [16.05.2014].
479. Divrikli U, Horzum N, Soylak M, Elci L. Trace heavy metal contents of some spices and herbal plants from western Anatolia, Turkey. *Int. J. Food Sci. Technol.* **2006**, *41*, 712–716.
480. Divrikli U, Horzum N, Soylak M, Elci L. Trace heavy metal contents of some spices and herbal plants from western Anatolia, Turkey. *Int. J. Food Sci. Technol.* **2006**, *41*, 712–716.
481. Borah, S.; Baruah, A.M.; Das, A.K.; Borah, J. Determination of Mineral Content in Commonly Consumed Leafy Vegetables. *Food Anal. Methods* **2009**, *2*, 226–230.
482. Vishwakarma, K.L.; Dubey, V. Nutritional analysis of indigenous wild edible herbs used in eastern Chhattisgarh, India. *Emir. J. Food Agric.* **2011**, *23*, 554–560.
483. Mazumdar, K.; Das, S.Phytoremediation of Pb, Zn, Fe, and Mg with 25 wetland plant species from a paper mill contaminated site in North East India. *Environ. Sci. Pollut. Res.* **2014**, doi:10.1007/s11356-014-3377-7.
484. Mazumdar, K.; Das, S.Phytoremediation of Pb, Zn, Fe, and Mg with 25 wetland plant species from a paper mill contaminated site in North East India. *Environ. Sci. Pollut. Res.* **2014**, doi:10.1007/s11356-014-3377-7.
485. Mazumdar, K.; Das, S. Phytoremediation of Pb, Zn, Fe, and Mg with 25 wetland plant species from a paper mill contaminated site in North East India. *Environ. Sci. Pollut. Res.* **2014**, doi:10.1007/s11356-014-3377-7.
486. Karmakar, K.; Muslim, T.; Rahman, M.A. Chemical Composition of Some Leafy Vegetables of Bangladesh. *Dhaka Univ. J. Sci.* **2013**, *61*, 199–201.
487. Memon, A.R.; Itô, S.; Yatazawa, M. Absorption and accumulation of iron, manganese and copper in plants in the temperate forest of central Japan. *Soil Sci. Plant Nutr.* **1979**, *15*, 611–620.
488. Wan, K.; Chen, F.; Tao, Y.; Chen, S. Nutrient Elements in Leaves of Rare and Endangered Species in Wuhan Botanical Garden, China. *J. Plant. Nutr.* **2009**, *32*, 1914–1940.
489. Adlassnig, W.; Steinhauser, G.; Peroutka, M.; Musilek, A.; Sterba, J.H.; Lichtscheidl, I.K.; Bichler, M. Expanding the menu for carnivorous plants: uptake of potassium, iron and manganese by carnivorous pitcher plants. Appl Radiat Isot. **2009**, *67*, 2117–22.
490. Ernst, W.H.O. Element Nutrition of Two Contrasted Dune Annuals. Journal of Ecology. **1983**, *71*, 197–209.
491. Memon, A.R.; Itô, S.; Yatazawa, M. Absorption and accumulation of iron, manganese and copper in plants in the temperate forest of central Japan. Soil Sci. Plant Nutr. **1979**, *15*, 611–620.
492. De la Fuente, V.; Rufo, L.; Rodríguez, N.; Amils, R.; Zuluaga, J. Metal accumulation screening of the Río Tinto flora (Huelva, Spain). Biol Trace Elem Res. **2010**, *134*, 318–41.
493. Linn JG, Staba EJ, Goodrich RD, Meiske JC, Otterby DE. Nutritive Value of Dried or Ensiled Aquatic Plants. I. Chemical Composition. *J. Anim. Sci.* **1975**, *41*, 601–609.
494. Wan, K.; Chen, F.; Tao, Y.; Chen, S. Nutrient Elements in Leaves of Rare and Endangered Species in Wuhan Botanical Garden, China. *J. Plant. Nutr.* **2009**, *32*, 1914–1940.
495. Rode, K.D.; Chapman, C.A.; Chapman, L.J.; McDowell, L.R. Mineral Resource Availability and Consumption by Colobus in Kibale National Park, Uganda. *Int. J. Primatol*. **2003**, *24*, 541–573.
496. Rode, K.D.; Chapman, C.A.; Chapman, L.J.; McDowell, L.R. Mineral Resource Availability and Consumption by Colobus in Kibale National Park, Uganda. *Int. J. Primatol*. **2003**, *24*, 541–573.
497. Rode KD, Chapman CA, Chapman LJ, McDowell LR. Mineral Resource Availability and Consumption by Colobus in Kibale National Park, Uganda. *Int. J. Primatol*. **2003**, *24*, 541–573.
498. De la Fuente, V.; Rufo, L.; Rodríguez, N.; Amils, R.; Zuluaga, J. Metal accumulation screening of the Río Tinto flora (Huelva, Spain). *Biol. Trace Elem. Res.* **2010**, *134*, 318–341.
499. Ansari, T.M.; Ikram, N.; Najam–ul–Haq, M.; Fayyaz, Y.; Fayyaz, Q.; Ghafoor, I.; Khalid, N. Essential Trace Metal (Zinc, Manganese, Copper and Iron) Levels in Plants of Medicinal Importance. Journal of Biological Sciences. **2004**, *4*, 95–99.
500. Wan, K.; Chen, F.; Tao, Y.; Chen, S. Nutrient Elements in Leaves of Rare and Endangered Species in Wuhan Botanical Garden, China. *J. Plant. Nutr.* **2009**, *32*, 1914–1940.
501. Tsuchiya, Y.; Shimogaki, H.; Abe, H.; Kagawa, A. Inorganic elements in typical Japanese trees for woody biomass fuel. *Wood Sci.* **2010**, *56*, 53–63.
502. Tsuchiya, Y.; Shimogaki, H.; Abe, H.; Kagawa, A. Inorganic elements in typical Japanese trees for woody biomass fuel. *Wood Sci.* **2010**, *56*, 53–63.
503. Tsuchiya, Y.; Shimogaki, H.; Abe, H.; Kagawa, A. Inorganic elements in typical Japanese trees for woody biomass fuel. *Wood Sci.* **2010**, *56*, 53–63.
504. Chizzola, R.; Michitsch, H.; Franz, C. Monitoring of metallic micronutrients and heavy metals in herbs, spices and medicinal plants from Austria. *Eur. Food Res. Technol.* **2003**, *216*, 407–411.
505. De la Fuente, V.; Rufo, L.; Rodríguez, N.; Amils, R.; Zuluaga, J. Metal accumulation screening of the Río Tinto flora (Huelva, Spain). *Biol. Trace Elem. Res.* **2010**, *134*, 318–41.
506. Wan K, Chen F, Tao Y, Chen S. Nutrient Elements in Leaves of Rare and Endangered Species in Wuhan Botanical Garden, China. *J. Plant. Nutr.* **2009**, *32*, 1914–1940.
507. Memon AR, Itô S, Yatazawa M. Absorption and accumulation of iron, manganese and copper in plants in the temperate forest of central Japan. *Soil Sci. Plant Nutr.* **1979**, *15*, 611–620.
508. Karmakar K, Muslim T, Rahman MA. Chemical Composition of Some Leafy Vegetables of Bangladesh. *Dhaka Univ. J. Sci.* **2013**, *61*, 199–201.
509. Borah, S.; Baruah, A.M.; Das, A.K.; Borah, J. Determination of Mineral Content in Commonly Consumed Leafy Vegetables. *Food Anal. Methods* **2009**, *2*, 226–230.
510. Duke JA, Ayensu ES. Medicinal Plants of China. Available online: http://pfaf.org/user/
     plant.aspx?LatinName=Chenopodium+album (accessed on 18 July 2014).
511. Vishwakarma, K.L.; Dubey, V. Nutritional analysis of indigenous wild edible herbs used in eastern Chhattisgarh, India. *Emir. J. Food Agric*. **2011**, *23*, 554–560.
512. Mazumdar, K.; Das, S. Phytoremediation of Pb, Zn, Fe, and Mg with 25 wetland plant species from a paper mill contaminated site in North East India. *Environ. Sci. Pollut. Res.* **2014**, doi:10.1007/s11356-014-3377-7.
513. Bhargava, A.; Shukla, S.; Srivastava, J.; Singh, N.; Ohri, D. Genetic diversity for mineral accumulation in the foliage of Chenopodium spp. *Sci. Hortic.* **2008**, *118*, 338–346.
514. Bhargava, A.; Shukla, S.; Srivastava, J.; Singh, N.; Ohri, D. Chenopodium: a prospective plant for phytoextraction. *Acta Physiol. Plant.* **2008**, *30*, 111–120.
515. Bhargava, A.; Shukla, S.; Srivastava, J.; Singh, N.; Ohri, D. Genetic diversity for mineral accumulation in the foliage of Chenopodium spp. *Sci. Hortic.* **2008**, *118*, 338–346.
516. Bhargava, A.; Shukla, S.; Srivastava, J.; Singh, N.; Ohri, D. Chenopodium: a prospective plant for phytoextraction. *Acta Physiol. Plant.* **2008**, *30*, 111–120.
517. Mazumdar, K.; Das, S. Phytoremediation of Pb, Zn, Fe, and Mg with 25 wetland plant species from a paper mill contaminated site in North East India. *Environ. Sci. Pollut. Res.* **2014**, doi:10.1007/s11356-014-3377-7.
518. Mazumdar, K.; Das, S. Phytoremediation of Pb, Zn, Fe, and Mg with 25 wetland plant species from a paper mill contaminated site in North East India. *Environ. Sci. Pollut. Res.* **2014**, doi:10.1007/s11356-014-3377-7.
519. Bhargava, A.; Shukla, S.; Srivastava, J.; Singh, N.; Ohri, D. Genetic diversity for mineral accumulation in the foliage of Chenopodium spp. *Sci. Hortic.* **2008**, *118*, 338–346.
520. Bhargava, A.; Shukla, S.; Srivastava, J.; Singh, N.; Ohri, D. Chenopodium: A prospective plant for phytoextraction. *Acta Physiol. Plant.* **2008**, *30*, 111–120.
521. Bhargava, A.; Shukla, S.; Srivastava, J.; Singh, N.; Ohri, D. Genetic diversity for mineral accumulation in the foliage of Chenopodium spp. *Sci. Hortic.* **2008**, *118*, 338–346.
522. Bhargava, A.; Shukla, S.; Srivastava, J.; Singh, N.; Ohri, D. Chenopodium: A prospective plant for phytoextraction. *Acta Physiol. Plant.* **2008**, *30*, 111–120.
523. Bhargava, A.; Shukla, S.; Srivastava, J.; Singh, N.; Ohri, D. Genetic diversity for mineral accumulation in the foliage of Chenopodium spp. *Sci. Hortic.* **2008**, *118*, 338–346.
524. Bhargava, A.; Shukla, S.; Srivastava, J.; Singh, N.; Ohri, D. Chenopodium: A prospective plant for phytoextraction. *Acta Physiol. Plant.* **2008**, *30*, 111–120.
525. Hussain, I.; Khattak, M.U.R.; Khan, F.A.; Rehman, I.U.; Khan, F.U.; Khan, F.U. Analysis of Heavy Metals in Selected Medicinal Plants from Dir, Swat and Peshawar Districts of Khyber Pakhtunkhwa. [*J. Chem. Soc. Pak.*](http://www.researchgate.net/journal/0253-5106_Journal-Chemical_Society_of_Pakistan)**2001**, *33*, 495–498.
526. Bhargava A, Shukla S, Srivastava J, Singh N, Ohri D. Genetic diversity for mineral accumulation in the foliage of Chenopodium spp. *Sci. Hortic.* **2008**, *118*, 338–346.
527. Bhargava A, Shukla S, Srivastava J, Singh N, Ohri D. Chenopodium: A prospective plant for phytoextraction. *Acta Physiol. Plant.* **2008**, *30*, 111–120.
528. Bhargava, A.; Shukla, S.; Srivastava, J.; Singh, N.; Ohri, D. Genetic diversity for mineral accumulation in the foliage of Chenopodium spp. Scientia Horticulturae. **2008**, *118*, 338–346.
529. Bhargava, A.; Shukla, S.; Srivastava, J.; Singh, N.; Ohri, D. Chenopodium: a prospective plant for phytoextraction. *Acta Physiol. Plant.* **2008**, *30*, 111–120.
530. Bhargava, A.; Shukla, S.; Srivastava, J.; Singh, N.; Ohri, D. Genetic diversity for mineral accumulation in the foliage of Chenopodium spp. Scientia Horticulturae. **2008**, *118*, 338–346.
531. Bhargava A, Shukla S, Srivastava J, Singh N, Ohri D. Chenopodium: a prospective plant for phytoextraction. *Acta Physiol. Plant.* **2008**, *30*, 111–120.
532. Bhargava A, Shukla S, Srivastava J, Singh N, Ohri D. Genetic diversity for mineral accumulation in the foliage of Chenopodium spp. Scientia Horticulturae. **2008**, *118*, 338–346.
533. Tanji A, Elgharous M. A survey of mineral composition of weed seeds. Weed Research. **1998**, *38*, 79–86.
534. Bhargava A, Shukla S, Srivastava J, Singh N, Ohri D. Chenopodium: a prospective plant for phytoextraction. Acta Physiol Plant. **2008**, *30*, 111–120.
535. Bhargava A, Shukla S, Srivastava J, Singh N, Ohri D. Chenopodium: a prospective plant for phytoextraction. Acta Physiol Plant. **2008**, *30*, 111–120.
536. Bhargava A, Shukla S, Srivastava J, Singh N, Ohri D. Chenopodium: a prospective plant for phytoextraction. Acta Physiol Plant. **2008**, *30*, 111–120.
537. Bhargava A, Shukla S, Srivastava J, Singh N, Ohri D. Genetic diversity for mineral accumulation in the foliage of Chenopodium spp. Scientia Horticulturae. **2008**, *118*, 338–346.
538. Bhargava A, Shukla S, Srivastava J, Singh N, Ohri D. Genetic diversity for mineral accumulation in the foliage of Chenopodium spp. Scientia Horticulturae. **2008**, *118*, 338–346.
539. Bhargava A, Shukla S, Ohri D. Chenopodium quinoa—An Indian perspective. Industrial Crops and Products. **2006**, *23*, 73–87.
540. Bhargava A, Shukla S, Ohri D. Chenopodium quinoa—An Indian perspective. Industrial Crops and Products. **2006**, *23*, 73–87.
541. Rode KD, Chapman CA, Chapman LJ, McDowell LR. Mineral Resource Availability and Consumption by Colobus in Kibale National Park, Uganda. *Int. J. Primatol*. **2003**, *24*, 541–573.
542. Sundriyal M, Sundriyal RC. Wild edible plants of the sikkim himalaya: nutritive values of selected species. Economic Botany. **2001**, *55*, 377–390.
543. Malayeri BE, Chehregani A, Yousefi N, Lorestani B. Identification of the Hyper Acumulator Plants in Copper and Iron Mine in Iran. Pakistan Journal of Biological Sciences. **2008**, *11*, 490–492.
544. Masal VP, Meena M. Screening of Pteridophytes for Inorganic Constituents In Some Species From Ratnagiri District of Maharashtra (India). *Bionano Front*. **2010**, *3*, 273–275
545. Masal VP, Meena M. Screening of Pteridophytes For Inorganic Constituents In Some Species From Ratnagiri District of Maharashtra (India). *Bionano Front*. **2010**, *3*, 273–275
546. Olivares E, Pena E, Marcano E, Mostacero J, Aguiar G, Benitez M, Rengifo E. Aluminum accumulation and its relationship with mineral plant nutrients in 12 pteridophytes from Venezuela. *Environ. Exp. Bot.* **2009**, *65*, 132–141.
547. Bassey ME, Johnny II, Okoro BI. Lesser known spices of Akwa Ibom State; their nutritional, antinutritional, mineral and phytochemical analyses. Arch. Appl. Sci. Res. **2011**, *3*, 553–559.
548. Schmidt DA, Iambana RB, Britt A, Junge RE, Welch CR, Porton IJ, Kerley MS. Nutrient composition of plants consumed by black and white ruffed lemurs, Varecia variegata, in the Betampona Natural Reserve, Madagascar. *Zoo Biol.* **2010**, *29*, 375–96.
549. Schmidt DA, Iambana RB, Britt A, Junge RE, Welch CR, Porton IJ, Kerley MS. Nutrient composition of plants consumed by black and white ruffed lemurs, Varecia variegata, in the Betampona Natural Reserve, Madagascar. *Zoo Biol.* **2010**, *29*, 375–96.
550. dos Santos UM, de Carvalho Gonçalvesa JF, Feldpausch TR. Growth, leaf nutrient concentration and photosynthetic nutrient use efficiency in tropical tree species planted in degraded areas in central Amazonia. Forest Ecology and Management. **2006**, *226*, 299–309.
551. Osaki M, Watanabe T, Ishizawa T, Nilnond C, Nuyim T, Sittibush C, Tadano T. Nutritional characteristics in leaves of native plants grown in acid sulfate, peat, sandy podzolic, and saline soils distributed in Peninsular Thailand. Plant and Soil. **1998**, *201*, 175–182.
552. Karunaratne AM, Amerasinghe PH, Sadagopa Ramanujam VM, Sandstead HH, Perera PAJ. Zinc, iron and phytic acid levels of some popular foods consumed by rural children in Sri Lanka. Journal of Food Composition and Analysis. **2008**, *21*, 481–488
553. ***. Plants for a future. Available online: http://www.pfaf.org/user/plant.aspx?LatinName=
     Cichorium+intybus [23.07.2014].
554. Ansari TM, Ikram N, Najam–ul–Haq M, Fayyaz Y, Fayyaz Q, Ghafoor I, Khalid N. Essential Trace Metal (Zinc, Manganese, Copper and Iron) Levels in Plants of Medicinal Importance. Journal of Biological Sciences. **2004**, *4*, 95–99.
555. Tsuchiya Y, Shimogaki H, Abe H, Kagawa A. Inorganic elements in typical Japanese trees for woody biomass fuel. *Wood Sci.* **2010**, *56*, 53–63.
556. Tsuchiya Y, Shimogaki H, Abe H, Kagawa A. Inorganic elements in typical Japanese trees for woody biomass fuel. *Wood Sci.* **2010**, *56*, 53–63.
557. Tsuchiya Y, Shimogaki H, Abe H, Kagawa A. Inorganic elements in typical Japanese trees for woody biomass fuel. *Wood Sci.* **2010**, *56*, 53–63.
558. Wan K, Chen F, Tao Y, Chen S. Nutrient Elements in Leaves of Rare and Endangered Species in Wuhan Botanical Garden, China. *J. Plant. Nutr.* **2009**, *32*, 1914–1940.
559. Wan K, Chen F, Tao Y, Chen S. Nutrient Elements in Leaves of Rare and Endangered Species in Wuhan Botanical Garden, China. *J. Plant. Nutr.* **2009**, *32*, 1914–1940.
560. Ozcan MM, Akbulut M. Estimation of minerals, nitrate and nitrite contents of medicinal and aromatic plants used as spices, condiments and herbal tea. Food Chem. **2008**, *106*, 852–858
561. Ansari TM, Ikram N, Najam–ul–Haq M, Fayyaz Y, Fayyaz Q, Ghafoor I, Khalid N. Essential Trace Metal (Zinc, Manganese, Copper and Iron) Levels in Plants of Medicinal Importance. Journal of Biological Sciences. **2004**, *4*, 95–99
562. Malayeri BE, Chehregani A, Yousefi N, Lorestani B. Identification of the Hyper Acumulator Plants in Copper and Iron Mine in Iran. Pakistan Journal of Biological Sciences. 2008; 11 :490–492
563. Grzegorczyk S, Olszewska M, Alberski J. Accumulation of copper, zinc, manganese and iron by selected species of grassland legumes and herbs. J. Elem. s. **2014**, *19*, 109–118
564. Grzegorczyk S, Olszewska M, Alberski J. Accumulation of copper, zinc, manganese and iron by selected species of grassland legumes and herbs. J. Elem. s. **2014**, *19*, 109–118
565. Malayeri BE, Chehregani A, Yousefi N, Lorestani B. Identification of the Hyper Acumulator Plants in Copper and Iron Mine in Iran. Pakistan Journal of Biological Sciences. 2008; 11 :490–492
566. Lockett CT, Calvert CC, Grivetti LE. Energy and micronutrient composition of dietary and medicinal wild plants consumed during drought. Study of rural Fulani, northeastern Nigeria. International Journal of Food Sciences and Nutrition; May **2000**, *51*, 195–208
567. Adebowale KO, Nwokocha LM, Agbaje WB. Composition of Cissus populnea stem. Journal of Food Composition and Analysis. **2013**, *30*, 41–46
568. Adebowale KO, Nwokocha LM, Agbaje WB. Composition of Cissus populnea stem. Journal of Food Composition and Analysis. **2013**, *30*, 41–46
569. Vishwakarma KL, Dubey V. Nutritional analysis of indigenous wild edible herbs used in eastern Chhattisgarh, India. *Emir. J. Food Agric*. **2011**, *23*, 554–560.
570. Yeotkar SD, Malode SN, Jadhao KD. Investigations of minerals from some ethno–vegetable and medicinal plants of Melghat region. *Bionano Front*. **2010**, *3*, 268–272.
571. de la Fuente V, Rufo L, Rodríguez N, Amils R, Zuluaga J. Metal accumulation screening of the Río Tinto flora (Huelva, Spain). *Biol Trace Elem Res.* **2010**, *134*, 318–41
572. Ugulu I, Dogan Y, Baslar S, Varol O. Biomonitoring of trace element accumulation in plants growing at Murat Mountain. Int. J. Environ. Sci. Technol. **2012**, *9*, 527–534
573. de la Fuente V, Rufo L, Rodríguez N, Amils R, Zuluaga J. Metal accumulation screening of the Río Tinto flora (Huelva, Spain). Biol Trace Elem Res. **2010**, *134*, 318–41
574. de la Fuente V, Rufo L, Rodríguez N, Amils R, Zuluaga J. Metal accumulation screening of the Río Tinto flora (Huelva, Spain). Biol Trace Elem Res. **2010**, *134*, 318–41
575. de la Fuente V, Rufo L, Rodríguez N, Amils R, Zuluaga J. Metal accumulation screening of the Río Tinto flora (Huelva, Spain). Biol Trace Elem Res. **2010**, *134*, 318–41
576. de la Fuente V, Rufo L, Rodríguez N, Amils R, Zuluaga J. Metal accumulation screening of the Río Tinto flora (Huelva, Spain). Biol Trace Elem Res. **2010**, *134*, 318–41
577. de la Fuente V, Rufo L, Rodríguez N, Amils R, Zuluaga J. Metal accumulation screening of the Río Tinto flora (Huelva, Spain). Biol Trace Elem Res. **2010**, *134*, 318–41
578. Ansari TM, Ikram N, Najam–ul–Haq M, Fayyaz Y, Fayyaz Q, Ghafoor I, Khalid N. Essential Trace Metal (Zinc, Manganese, Copper and Iron) Levels in Plants of Medicinal Importance. Journal of Biological Sciences. **2004**, *4*, 95–99
579. Umar KJ, Hassan LG, Usman H, Wasagu RSU. Nutritional Composition of the Seeds of Wild Melon (Citrullus ecirrhosus). Pak. J. Biol. Sci. **2013**, *16*, 536–40.
580. Duke JA, Ayensu ES. Medicinal Plants of China. Available online: http://www.pfaf.org/user/
     Plant.aspx?LatinName=Citrullus+lanatus [23.07.2014]
581. Carpena Artes O, Moreno JJ, Lucena JJ, Carpena Ruiz RO. Response to iron chlorosis of different hydroponically grown Citrus varieties. Iron nutrition in soil and plants. In: J. Abadia (Ed.). Springer Science + Business Media, Dordrecht, 1995, 147–151
582. Carpena Artes O, Moreno JJ, Lucena JJ, Carpena Ruiz RO. Response to iron chlorosis of different hydroponically grown Citrus varieties. Iron nutrition in soil and plants. In: J. Abadia (Ed.). Springer Science + Business Media, Dordrecht, 1995, 147–151
583. Duke JA, Ayensu ES. Medicinal Plants of China. Available online: http://www.pfaf.org/user/
     Plant.aspx?LatinName=Fortunella+margarita [23.07.2014].
584. Duke JA, Ayensu ES. Medicinal Plants of China. Available online: http://www.pfaf.org/user/
     Plant.aspx?LatinName=Citrus+limon [18.07.2014].
585. Carpena Artes O, Moreno JJ, Lucena JJ, Carpena Ruiz RO. Response to iron chlorosis of different hydroponically grown Citrus varieties. In: Iron nutrition in soil and plants. J. Abadia (Ed.). Springer Science + Business Media, Dordrecht, 1995, 147–151.
586. Mayer AM, Gorham E. The Iron and Manganese Content of Plants present in the Natural Vegetation of the English Lake District. *Ann. Bot.* **1951**, *15*, 247–263.
587. Rode KD, Chapman CA, Chapman LJ, McDowell LR. Mineral Resource Availability and Consumption by Colobus in Kibale National Park, Uganda. *Int. J. Primatol*. **2003**, *24*, 541–573.
588. Rode KD, Chapman CA, Chapman LJ, McDowell LR. Mineral Resource Availability and Consumption by Colobus in Kibale National Park, Uganda. *Int. J. Primatol*. **2003**, *24*, 541–573.
589. Liu Y, Ding H，Zhu Y. Metal bioaccumulation in plant leaves from an industrious area and the Botanical Garden in Beijing. Journal of Environmental Sciences. 2005; 17 : 294–300
590. Schönfeldt HC, Pretorius B. The nutrient content of five traditional South African dark green leafy vegetables–A preliminary study. Journal of Food Composition and Analysis. 2011; 24 : 1141–1146
591. Yeotkar SD, Malode SN, Jadhao KD. Investigations of minerals from some ethno–vegetable and medicinal plants of Melghat region. *Bionano Front*. **2010**, *3*, 268–272.
592. Memon AR, Itô S, Yatazawa M. Absorption and accumulation of iron, manganese and copper in plants in the temperate forest of central Japan. Soil Sci. Plant Nutr. 1979; 15 : 611–620
593. Gbadamosi IT, Obogo SF. Chemical Constituents and In Vitro Antimicrobial Activities of Five botanicals Used Traditionally for the Treatment of Neonatal Jaundice in Ibadan, Nigeria. Nature and Science 2013;11 : 130–135
594. Duke JA, Ayensu ES. Medicinal Plants of China. Available online: http://www.pfaf.org/user/
     Plant.aspx?LatinName=Coix+lacryma–jobi [23.07.2014]
595. Guerrero–Cervantes M, Ramírez RG, González–Rodríguez H, Cerrillo–Soto A, Juárez–Réyes A. Mineral content in range forages from north Mexico. *J. Appl. Anim. Res.* **2012**, *40*, 102–107.
596. Borah S, Baruah AM, Das AK, Borah J. Determination of Mineral Content in Commonly Consumed Leafy Vegetables. Food Anal. Methods. **2009**, *2*, 226–230.
597. Mazumdar K, Das S. Phytoremediation of Pb, Zn, Fe, and Mg with 25 wetland plant species from a paper mill contaminated site in North East India. *Environ. Sci. Pollut. Res.* **2014**, doi:10.1007/s11356-014-3377-7.
598. Yeotkar SD, Malode SN, Jadhao KD. Investigations of minerals from some ethno–vegetable and medicinal plants of Melghat region. *Bionano Front*. **2010**, *3*, 268–272.
599. Vishwakarma KL, Dubey V. Nutritional analysis of indigenous wild edible herbs used in eastern Chhattisgarh, India. *Emir. J. Food Agric*. **2011**, *23*, 554–560.
600. Acho CF, Zoue LT, Akpa EE, Yapo VG, Niamke SL. Leafy vegetables consumed in Southern Côte d’Ivoire: a source of high value nutrients. Journal of Animal &Plant Sciences. **2014**, *20*, 3159–3170
601. Mazumdar K, Das S. Phytoremediation of Pb, Zn, Fe, and Mg with 25 wetland plant species from a paper mill contaminated site in North East India. *Environ. Sci. Pollut. Res.* **2014**, doi:10.1007/s11356-014-3377-7.
602. Irawan D, Wijaya CH, Limin SH, Hashidoko Y, Osaki M, Kulu IP. Ethnobotanical study and nutrient potency of local traditional vegetables in Central Kalimantan. Tropics. **2006**, *15*, 441–448.
603. Mazumdar K, Das S. Phytoremediation of Pb, Zn, Fe, and Mg with 25 wetland plant species from a paper mill contaminated site in North East India. *Environ. Sci. Pollut. Res.* **2014**, doi:10.1007/s11356-014-3377-7.
604. Karmakar K, Muslim T, Rahman MA. Chemical Composition of Some Leafy Vegetables of Bangladesh. Dhaka Univ. J. Sci. **2013**, *61*, 199–201.
605. Kashin VK. Vitally Important Microelements in Transbaikalian Herbs. *Chem. Sustain. Dev.* **2009**, *17*, 371–381.
606. Agunbiade OS, Ojezele OM, Ojezele JO, Ajayi AY. Hypoglycaemic activity of Commelina africana and Ageratum conyzoides in relation to their mineral composition. Afr Health Sci. **2012**, *12*, 198–203
607. Vishwakarma KL, Dubey V. Nutritional analysis of indigenous wild edible herbs used in eastern Chhattisgarh, India. *Emir. J. Food Agric*. **2011**, *23*, 554–560.
608. Kuria SG, IA Tura, S Amboga, HK Walaga, 2012. Assessment of status of minerals in forages preferred by camels (Camelus dromedarius) in the Arid North Eastern Kenya. Res. Opin. Anim. Vet. Sci. **2012**, *2*, 173–180.
609. Guerrero–Cervantes M, Ramírez RG, González–Rodríguez H, Cerrillo–Soto A, Juárez–Réyes A. Mineral content in range forages from north Mexico. *J. Appl. Anim. Res.* **2012**, *40*, 102–107.
610. Kozanecka T, Chojnicki J, Kwasowski W. Content of Heavy Metals in Plant from Pollution–Free Regions. *Pol. J. Environ. Stud.* **2002**, *11*, 395–399
611. Kozanecka T, Chojnicki J, Kwasowski W. Content of Heavy Metals in Plant from Pollution–Free Regions. *Pol. J. Environ. Stud.* **2002**, *11*, 395–399
612. Karmakar K, Muslim T, Rahman MA. Chemical Composition of Some Leafy Vegetables of Bangladesh. Dhaka Univ. J. Sci. **2013**, *61*, 199–201.
613. Acho CF, Zoue LT, Akpa EE, Yapo VG, Niamke SL. Leafy vegetables consumed in Southern Côte d’Ivoire: a source of high value nutrients. Journal of Animal &Plant Sciences. **2014**, *20*, 3159–3170.
614. Schönfeldt HC, Pretorius B. The nutrient content of five traditional South African dark green leafy vegetables–A preliminary study. Journal of Food Composition and Analysis. **2011**, *24*, 1141–1146.
615. Vishwakarma KL, Dubey V. Nutritional analysis of indigenous wild edible herbs used in eastern Chhattisgarh, India. *Emir. J. Food Agric*. **2011**, *23*, 554–560.
616. Rode KD, Chapman CA, Chapman LJ, McDowell LR. Mineral Resource Availability and Consumption by Colobus in Kibale National Park, Uganda. *Int. J. Primatol*. **2003**, *24*, 541–573.
617. Rode KD, Chapman CA, Chapman LJ, McDowell LR. Mineral Resource Availability and Consumption by Colobus in Kibale National Park, Uganda. *Int. J. Primatol*. **2003**, *24*, 541–573.
618. Rode KD, Chapman CA, Chapman LJ, McDowell LR. Mineral Resource Availability and Consumption by Colobus in Kibale National Park, Uganda. *Int. J. Primatol*. **2003**, *24*, 541–573.
619. Rode KD, Chapman CA, Chapman LJ, McDowell LR. Mineral Resource Availability and Consumption by Colobus in Kibale National Park, Uganda. *Int. J. Primatol*. **2003**, *24*, 541–573.
620. Guerrero–Cervantes M, Ramírez RG, González–Rodríguez H, Cerrillo–Soto A, Juárez–Réyes A. Mineral content in range forages from north Mexico. *J. Appl. Anim. Res.* **2012**, *40*, 102–107
621. Kuria SG, IA Tura, S Amboga, HK Walaga, 2012. Assessment of status of minerals in forages preferred by camels (Camelus dromedarius) in the Arid North Eastern Kenya. Res. Opin. Anim. Vet. Sci. **2012**, *2*, 173–180.
622. Kuria SG, IA Tura, S Amboga, HK Walaga, 2012. Assessment of status of minerals in forages preferred by camels (Camelus dromedarius) in the Arid North Eastern Kenya. Res. Opin. Anim. Vet. Sci. **2012**, *2*, 173–180.
623. Kuria SG, IA Tura, S Amboga, HK Walaga, 2012. Assessment of status of minerals in forages preferred by camels (Camelus dromedarius) in the Arid North Eastern Kenya. Res. Opin. Anim. Vet. Sci. **2012**, *2*, 173–180.
624. Chizzola R, Michitsch H, Franz C. Monitoring of metallic micronutrients and heavy metals in herbs, spices and medicinal plants from Austria. Eur Food Res Technol. **2003**, *216*, 407–411.
625. Soylak M, Cihan Z, Yilmaz E. Evaluation of trace element contents of some herbal plants and spices retailed in Kayseri, Turkey. Environ Monit Assess. **2012**, *184*, 3455–3461.
626. Ansari TM, Ikram N, Najam–ul–Haq M, Fayyaz Y, Fayyaz Q, Ghafoor I, Khalid N. Essential Trace Metal (Zinc, Manganese, Copper and Iron) Levels in Plants of Medicinal Importance. Journal of Biological Sciences. **2004**, *4*, 95–99
627. Karmakar K, Muslim T, Rahman MA. Chemical Composition of Some Leafy Vegetables of Bangladesh. Dhaka Univ. J. Sci. **2013**, *61*, 199–201
628. Wan K, Chen F, Tao Y, Chen S. Nutrient Elements in Leaves of Rare and Endangered Species in Wuhan Botanical Garden, China. *J. Plant. Nutr.* **2009**, *32*, 1914–1940
629. de la Fuente V, Rufo L, Rodríguez N, Amils R, Zuluaga J. Metal accumulation screening of the Río Tinto flora (Huelva, Spain). Biol Trace Elem Res. **2010**, *134*, 318–41
630. Mayer AM, Gorham E. The Iron and Manganese Content of Plants present in the Natural Vegetation of the English Lake District. *Ann. Bot.* **1951**, *15*, 247–263
631. Duke JA, Ayensu ES. Medicinal Plants of China. Available online: http://www.pfaf.org/user/
     Plant.aspx?LatinName=Corylus+avellana [23.07.2014]
632. Wan K, Chen F, Tao Y, Chen S. Nutrient Elements in Leaves of Rare and Endangered Species in Wuhan Botanical Garden, China. *J. Plant. Nutr.* **2009**, *32*, 1914–1940
633. Ansari TM, Ikram N, Najam–ul–Haq M, Fayyaz Y, Fayyaz Q, Ghafoor I, Khalid N. Essential Trace Metal (Zinc, Manganese, Copper and Iron) Levels in Plants of Medicinal Importance. Journal of Biological Sciences. **2004**, *4*, 95–99
634. Liu Y, Ding H，Zhu Y. Metal bioaccumulation in plant leaves from an industrious area and the Botanical Garden in Beijing. Journal of Environmental Sciences. **2005**, *17*, 294–300
635. Liu Y, Ding H，Zhu Y. Metal bioaccumulation in plant leaves from an industrious area and the Botanical Garden in Beijing. Journal of Environmental Sciences. **2005**, *17*, 294–300
636. Malayeri BE, Chehregani A, Yousefi N, Lorestani B. Identification of the Hyper Acumulator Plants in Copper and Iron Mine in Iran. Pakistan Journal of Biological Sciences. **2008**, *11*, 490–492
637. Arawande JO, Komolafe EA, Imokhuede B. Nutritional and phytochemical compositions of fireweed (Crassocephalum crepidioides). Journal of Agricultural Technology. **2013**, *9*, 439–449
638. Sowa I, Wojciak–Kosior M, Kocjan R. The Content Of Some Trace Elements In Selected Medicinal Plants Collected In The Province Of Lublin. Acta Scientiarum Polonorum–Hortorum Cultus. **2012**, *11*, 15–22
639. Stef DS, Gergen I, Trasca TI *et al.* Screening of 33 Medicinal Plants for the Microelements Content. Animal Science and Biotechnologies, **2010**, *43*, 127–132
640. Duke JA, Ayensu ES. Medicinal Plants of China. Cited by http://www.pfaf.org/user/
     Plant.aspx?LatinName=Crataegus+pinnatifida [23.07.2014]
641. Schmidt DA, Iambana RB, Britt A, Junge RE, Welch CR, Porton IJ, Kerley MS. Nutrient composition of plants consumed by black and white ruffed lemurs, Varecia variegata, in the Betampona Natural Reserve, Madagascar. *Zoo Biol.* **2010**, *29*, 375–96
642. Tsuchiya Y, Shimogaki H, Abe H, Kagawa A. Inorganic elements in typical Japanese trees for woody biomass fuel. *Wood Sci.* **2010**, *56*, 53–63
643. Tsuchiya Y, Shimogaki H, Abe H, Kagawa A. Inorganic elements in typical Japanese trees for woody biomass fuel. *Wood Sci.* **2010**, *56*, 53–63
644. Memon AR, Itô S, Yatazawa M. Absorption and accumulation of iron, manganese and copper in plants in the temperate forest of central Japan. Soil Sci. Plant Nutr. **1979**, *15*, 611–620
645. Tsuchiya Y, Shimogaki H, Abe H, Kagawa A. Inorganic elements in typical Japanese trees for woody biomass fuel. *Wood Sci.* **2010**, *56*, 53–63
646. Tsuchiya Y, Shimogaki H, Abe H, Kagawa A. Inorganic elements in typical Japanese trees for woody biomass fuel. *Wood Sci.* **2010**, *56*, 53–63
647. Kubo T, Ataka S. Blackening of sugi (Cryptomeria japonica D. Don) heartwood in relation to metal content and moisture content. *J. Wood Sci.* **1998**, *44*, 137–141.
648. Kubo T, Ataka S. Blackening of sugi (Cryptomeria japonica D. Don) heartwood in relation to metal content and moisture content. *J. Wood Sci.* **1998**, *44*, 137–141.
649. Kubo T, Ataka S. Blackening of sugi (Cryptomeria japonica D. Don) heartwood in relation to metal content and moisture content. *J. Wood Sci.* **1998**, *44*, 137–141.
650. Kubo T, Ataka S. Blackening of sugi (Cryptomeria japonica D. Don) heartwood in relation to metal content and moisture content. *J. Wood Sci.* **1998**, *44*, 137–141.
651. Sundriyal M, Sundriyal RC. Wild edible plants of the sikkim himalaya: Nutritive values of selected species. Economic Botany. **2001**, *55*, 377–390.
652. Ali MHH, Al–Quahtani K. Assessment of some heavy metals in vegetables, cereals and fruits in Saudi Arabian markets. *Egypt. J. Aquatic Res.* **2012**, *38*, 31–37
653. Karmakar K, Muslim T, Rahman MA. Chemical Composition of Some Leafy Vegetables of Bangladesh. *Dhaka Univ. J. Sci.* **2013**, *61*, 199–201
654. Schönfeldt HC, Pretorius B. The nutrient content of five traditional South African dark green leafy vegetables–A preliminary study. Journal of Food Composition and Analysis. **2011**, *24*, 1141–1146
655. Amde M, Megersa N, Taddesse AM, Bedassa T. Determination of the levels of selected metals in seeds, flowers and fruits of medicinal plants used for tapeworm treatment in Ethiopia. Toxicological & Environmental Chemistry. **2013**, *95*, 82–100
656. Amde M, Megersa N, Taddesse AM, Bedassa T. Determination of the levels of selected metals in seeds, flowers and fruits of medicinal plants used for tapeworm treatment in Ethiopia. Toxicological & Environmental Chemistry. **2013**, *95*, 82–100
657. Duke JA, Ayensu ES. Medicinal Plants of China. Available online: http://www.pfaf.org/user/plant.aspx?LatinName=Cucurbita+pepo [23.07.2014]
658. Iheanako K, Udebuani A. Nutritional Composition of Some Leafy Vegetables Consumed in Imo State, Nigeria. J. Appl. Sci. Environ. Manage. 2009; 13: 35–38
659. Ansari TM, Ikram N, Najam–ul–Haq M, Fayyaz Y, Fayyaz Q, Ghafoor I, Khalid N. Essential Trace Metal (Zinc, Manganese, Copper and Iron) Levels in Plants of Medicinal Importance. Journal of Biological Sciences. **2004**, *4*, 95–99
660. Ozcan MM, Akbulut M. Estimation of minerals, nitrate and nitrite contents of medicinal and aromatic plants used as spices, condiments and herbal tea. Food Chem. 2008; 106 : 852–858
661. Ozkutlu F. Determination of cadmium and trace elements in some spices cultivated in Turkey. Asian Journal of Chemistry. 2008; 20:1081–1088
662. Gupta KK, Bhattacharjee S, Kar S *et al.* Mineral Compositions of Eight Common Spices, Communications in Soil Science and Plant Analysis. 2003; 34(5–6):681–693
663. Gupta KK, Bhattacharjee S, Kar S *et al.* Mineral Compositions of Eight Common Spices, Communications in Soil Science and Plant Analysis. 2003; 34(5–6):681–693
664. Rihawy MS, Bakraji EH, Odeh A. PIXE and GC–MS investigation for the determination of the chemical composition of Syrian Cuminum cyminum L. Applied Radiation and Isotopes. **2014**, *86*, 118–125
665. Alamin MB, Mhapes AA, Bejey AM, Sadek A, Atweer RH, Dubali K, Saad DM. Determination of essential and toxic elements in Libyan foodstuff using instrumental neutron activation analysis (INAA). Journal of Radioanalytical and Nuclear Chemistry. 2007; 271 , 247–250
666. Gupta KK, Bhattacharjee S, Kar S *et al.* Mineral Compositions of Eight Common Spices, Communications in Soil Science and Plant Analysis. **2003**, *34*, 681–693
667. Ansari TM, Ikram N, Najam–ul–Haq M, Fayyaz Y, Fayyaz Q, Ghafoor I, Khalid N. Essential Trace Metal (Zinc, Manganese, Copper and Iron) Levels in Plants of Medicinal Importance. Journal of Biological Sciences. **2004**, *4*, 95–99
668. Wan K, Chen F, Tao Y, Chen S. Nutrient Elements in Leaves of Rare and Endangered Species in Wuhan Botanical Garden, China. *J. Plant. Nutr.* **2009**, *32*, 1914–1940
669. Hussain I, Khattak MUR, Khan FA, Rehman IU, Khan FU, Khan FU. Analysis of Heavy Metals in Selected Medicinal Plants from Dir, Swat and Peshawar Districts of Khyber Pakhtunkhwa. 2001; 33 : 495–498
670. Wan K, Chen F, Tao Y, Chen S. Nutrient Elements in Leaves of Rare and Endangered Species in Wuhan Botanical Garden, China. *J. Plant. Nutr.* **2009**, *32*, 1914–1940
671. Morton–Bermea O, Gomez–Bernal JM, Armienta MA, Lozano R, Hernandez–Alvarez E, Romero F, Castro–Larragoitia J. Metal accumulation by plant species growing on a mine contaminated site in Mexico. Environ Earth Sci. **2014**, *71*, 5207–5213
672. Morton–Bermea O, Gomez–Bernal JM, Armienta MA, Lozano R, Hernandez–Alvarez E, Romero F, Castro–Larragoitia J. Metal accumulation by plant species growing on a mine contaminated site in Mexico. Environ Earth Sci. **2014**, *71*, 5207–5213
673. El–Hasan T, Al–Omari H, Jiries A, Al–Nasir F. Cypress tree (Cupressus semervirens L.) bark as an indicator for heavy metal pollution in the atmosphere of Amman City, Jordan. Environment International. **2002**, *28*, 513–519
674. El–Hasan T, Al–Omari H, Jiries A, Al–Nasir F. Cypress tree (Cupressus semervirens L.) bark as an indicator for heavy metal pollution in the atmosphere of Amman City, Jordan. Environment International. **2002**, *28*, 513–519
675. El–Hasan T, Al–Omari H, Jiries A, Al–Nasir F. Cypress tree (Cupressus semervirens L.) bark as an indicator for heavy metal pollution in the atmosphere of Amman City, Jordan. Environment International. **2002**, *28*, 513–519
676. El–Hasan T, Al–Omari H, Jiries A, Al–Nasir F. Cypress tree (Cupressus semervirens L.) bark as an indicator for heavy metal pollution in the atmosphere of Amman City, Jordan. Environment International. **2002**, *28*, 513–519
677. El–Quesni FEM, Taha LS, Ibrahim SMM, Farahat MM. Growth and chemical constituents of Cupresus sempervirens L. plant as influence by kinetin and iron treatments at Nubaria. American–Eurasian J. Agric. & Environ. Sci. **2007**, *2*, 282–288
678. El–Quesni FEM, Taha LS, Ibrahim SMM, Farahat MM. Growth and chemical constituents of Cupresus sempervirens L. plant as influence by kinetin and iron treatments at Nubaria. American–Eurasian J. Agric. & Environ. Sci. **2007**, *2*, 282–288
679. El–Quesni FEM, Taha LS, Ibrahim SMM, Farahat MM. Growth and chemical constituents of Cupresus sempervirens L. plant as influence by kinetin and iron treatments at Nubaria. American–Eurasian J. Agric. & Environ. Sci. **2007**, *2*, 282–288
680. El–Quesni FEM, Taha LS, Ibrahim SMM, Farahat MM. Growth and chemical constituents of Cupresus sempervirens L. plant as influence by kinetin and iron treatments at Nubaria. American–Eurasian J. Agric. & Environ. Sci. **2007**, *2*, 282–288
681. El–Quesni FEM, Taha LS, Ibrahim SMM, Farahat MM. Growth and chemical constituents of Cupresus sempervirens L. plant as influence by kinetin and iron treatments at Nubaria. American–Eurasian J. Agric. & Environ. Sci. **2007**, *2*, 282–288
682. El–Quesni FEM, Taha LS, Ibrahim SMM, Farahat MM. Growth and chemical constituents of Cupresus sempervirens L. plant as influence by kinetin and iron treatments at Nubaria. American–Eurasian J. Agric. & Environ. Sci. **2007**, *2*, 282–288
683. Rucandio MI, Petit–Domínguez MD, Fidalgo–Hijano C, García–Giménez R. Biomonitoring of chemical elements in an urban environment using arboreal and bush plant species. Environ Sci Pollut Res. 2011; 18:51–63
684. El–Quesni FEM, Taha LS, Ibrahim SMM, Farahat MM. Growth and chemical constituents of Cupresus sempervirens L. plant as influence by kinetin and iron treatments at Nubaria. American–Eurasian J. Agric. & Environ. Sci. **2007**, *2*, 282–288
685. El–Quesni FEM, Taha LS, Ibrahim SMM, Farahat MM. Growth and chemical constituents of Cupresus sempervirens L. plant as influence by kinetin and iron treatments at Nubaria. American–Eurasian J. Agric. & Environ. Sci. **2007**, *2*, 282–288
686. El–Quesni FEM, Taha LS, Ibrahim SMM, Farahat MM. Growth and chemical constituents of Cupresus sempervirens L. plant as influence by kinetin and iron treatments at Nubaria. American–Eurasian J. Agric. & Environ. Sci. **2007**, *2*, 282–288
687. Ansari TM, Ikram N, Najam–ul–Haq M, Fayyaz Y, Fayyaz Q, Ghafoor I, Khalid N. Essential Trace Metal (Zinc, Manganese, Copper and Iron) Levels in Plants of Medicinal Importance. Journal of Biological Sciences. **2004**, *4*, 95–99
688. Mazumdar K, Das S. Phytoremediation of Pb, Zn, Fe, and Mg with 25 wetland plant
689. species from a paper mill contaminated site in North East India. E *Environ. Sci. Pollut. Res.* **2014**, doi:10.1007/s11356-014-3377-7.
690. Mazumdar K, Das S. Phytoremediation of Pb, Zn, Fe, and Mg with 25 wetland plant species from a paper mill contaminated site in North East India. *Environ. Sci. Pollut. Res.* **2014**, doi:10.1007/s11356-014-3377-7.
691. Chizzola R, Michitsch H, Franz C. Monitoring of metallic micronutrients and heavy metals in herbs, spices and medicinal plants from Austria. Eur Food Res Technol. **2003**, *216*, 407–411
692. Olivares E, Pena E, Marcano E, Mostacero J, Aguiar G, Benitez M, Rengifo E. Aluminum accumulation and its relationship with mineral plant nutrients in 12 pteridophytes from Venezuela. *Environ. Exp. Bot.* **2009**, *65*, 132–141
693. Olivares E, Pena E, Marcano E, Mostacero J, Aguiar G, Benitez M, Rengifo E. Aluminum accumulation and its relationship with mineral plant nutrients in 12 pteridophytes from Venezuela. *Environ. Exp. Bot.* **2009**, *65*, 132–141
694. Duke JA, Ayensu ES. Medicinal Plants of China. Available online: http://www.pfaf.org/user/
     plant.aspx?LatinName=Cydonia+oblonga [23.07.2014]
695. Guerrero–Cervantes M, Ramírez RG, González–Rodríguez H, Cerrillo–Soto A, Juárez–Réyes A. Mineral content in range forages from north Mexico. *J. Appl. Anim. Res.* **2012**, *40*, 102–107
696. Guerrero–Cervantes M, Ramírez RG, González–Rodríguez H, Cerrillo–Soto A, Juárez–Réyes A. Mineral content in range forages from north Mexico. *J. Appl. Anim. Res.* **2012**, *40*, 102–107
697. Guerrero–Cervantes M, Ramírez RG, González–Rodríguez H, Cerrillo–Soto A, Juárez–Réyes A. Mineral content in range forages from north Mexico. *J. Appl. Anim. Res.* **2012**, *40*, 102–107
698. Guerrero–Cervantes M, Ramírez RG, González–Rodríguez H, Cerrillo–Soto A, Juárez–Réyes A. Mineral content in range forages from north Mexico. *J. Appl. Anim. Res.* **2012**, *40*, 102–107
699. Malik J, Frankova A, Drabek O, Szakova J, Ash C, Kokoska L. Aluminium and other elements in selected herbal tea plant species and their infusions. Food Chem. 2013; 139(1–4):728–34
700. Malik J, Frankova A, Drabek O, Szakova J, Ash C, Kokoska L. Aluminium and other elements in selected herbal tea plant species and their infusions. Food Chem. **2013**, *139*, 728–34
701. Malik J, Frankova A, Drabek O, Szakova J, Ash C, Kokoska L. Aluminium and other elements in selected herbal tea plant species and their infusions. Food Chem. **2013**, *139*, 728–34
702. Colla G, Rouphael Y, Cardarelli M, Svecova E, Rea E, Lucini L. Effects of saline stress on mineral composition, phenolic acids and flavonoids in leaves of artichoke and cardoon genotypes grown in floating system. J Sci Food Agric. **2013**, *93*, 1119–27.
703. Colla G, Rouphael Y, Cardarelli M, Svecova E, Rea E, Lucini L. Effects of saline stress on mineral composition, phenolic acids and flavonoids in leaves of artichoke and cardoon genotypes grown in floating system. J Sci Food Agric. **2013**, *93*, 1119–27.
704. Colla G, Rouphael Y, Cardarelli M, Svecova E, Rea E, Lucini L. Effects of saline stress on mineral composition, phenolic acids and flavonoids in leaves of artichoke and cardoon genotypes grown in floating system. J Sci Food Agric. **2013**, *93*, 1119–27.
705. Colla G, Rouphael Y, Cardarelli M, Svecova E, Rea E, Lucini L. Effects of saline stress on mineral composition, phenolic acids and flavonoids in leaves of artichoke and cardoon genotypes grown in floating system. J Sci Food Agric. **2013**, *93*, 1119–27.
706. Colla G, Rouphael Y, Cardarelli M, Svecova E, Rea E, Lucini L. Effects of saline stress on mineral composition, phenolic acids and flavonoids in leaves of artichoke and cardoon genotypes grown in floating system. J Sci Food Agric. **2013**, *93*, 1119–27.
707. Colla G, Rouphael Y, Cardarelli M, Svecova E, Rea E, Lucini L. Effects of saline stress on mineral composition, phenolic acids and flavonoids in leaves of artichoke and cardoon genotypes grown in floating system. J Sci Food Agric. **2013**, *93*, 1119–27.
708. Colla G, Rouphael Y, Cardarelli M, Svecova E, Rea E, Lucini L. Effects of saline stress on mineral composition, phenolic acids and flavonoids in leaves of artichoke and cardoon genotypes grown in floating system. J Sci Food Agric. **2013**, *93*, 1119–27.
709. Colla G, Rouphael Y, Cardarelli M, Svecova E, Rea E, Lucini L. Effects of saline stress on mineral composition, phenolic acids and flavonoids in leaves of artichoke and cardoon genotypes grown in floating system. J Sci Food Agric. **2013**, *93*, 1119–27.
710. Colla G, Rouphael Y, Cardarelli M, Svecova E, Rea E, Lucini L. Effects of saline stress on mineral composition, phenolic acids and flavonoids in leaves of artichoke and cardoon genotypes grown in floating system. J Sci Food Agric. **2013**, *93*, 1119–27.
711. Colla G, Rouphael Y, Cardarelli M, Svecova E, Rea E, Lucini L. Effects of saline stress on mineral composition, phenolic acids and flavonoids in leaves of artichoke and cardoon genotypes grown in floating system. J Sci Food Agric. **2013**, *93*, 1119–27.
712. Colla G, Rouphael Y, Cardarelli M, Svecova E, Rea E, Lucini L. Effects of saline stress on mineral composition, phenolic acids and flavonoids in leaves of artichoke and cardoon genotypes grown in floating system. J Sci Food Agric. **2013**, *93*, 1119–27
713. Colla G, Rouphael Y, Cardarelli M, Svecova E, Rea E, Lucini L. Effects of saline stress on mineral composition, phenolic acids and flavonoids in leaves of artichoke and cardoon genotypes grown in floating system. J Sci Food Agric. **2013**, *93*, 1119–27.
714. Razić S, Dogo S, Slavković L. Investigation on bioavailability of some essential and toxic elements in medicinal herbs. J Nat Med. **2008**, *62*, 340–4.
715. Mazumdar K, Das S. Phytoremediation of Pb, Zn, Fe, and Mg with 25 wetland plant species from a paper mill contaminated site in North East India. *Environ. Sci. Pollut. Res.* **2014**, doi:10.1007/s11356-014-3377-7.
716. Mazumdar K, Das S. Phytoremediation of Pb, Zn, Fe, and Mg with 25 wetland plant species from a paper mill contaminated site in North East India. *Environ. Sci. Pollut. Res.* **2014**, doi:10.1007/s11356-014-3377-7.
717. Mazumdar K, Das S. Phytoremediation of Pb, Zn, Fe, and Mg with 25 wetland plant species from a paper mill contaminated site in North East India. *Environ. Sci. Pollut. Res.* **2014**, doi:10.1007/s11356-014-3377-7.
718. Rajendran D, Balakrishnan V. Diet Composition, Biomass Yield and Mineral Contents of Vegetation in Native Tract of Mecheri Sheep. Animal Nutrition and Feed Technology. **2012**, *12*, 63–71.
719. Animal Nutrition Group, National Dairy Development Board. Nutritive value of commonly available feeds and fodders in India, 2012. Available online:: http://www.nddb.coop/English/Services/AN/
     Documents/Animal–Nutrition–booklet.pdf [09.02.2014].
720. de la Fuente V, Rufo L, Rodríguez N, Amils R, Zuluaga J. Metal accumulation screening of the Río Tinto flora (Huelva, Spain). Biol Trace Elem Res. **2010**, *134*, 318–41.
721. Schmidt DA, Iambana RB, Britt A, Junge RE, Welch CR, Porton IJ, Kerley MS. Nutrient composition of plants consumed by black and white ruffed lemurs, Varecia variegata, in the Betampona Natural Reserve, Madagascar. *Zoo Biol.* **2010**, *29*, 375–96.
722. Rajendran D, Balakrishnan V. Diet Composition, Biomass Yield and Mineral Contents of Vegetation in Native Tract of Mecheri Sheep. Animal Nutrition and Feed Technology. **2012**, *12*, 63–71.
723. Rajendran D, Balakrishnan V. Diet Composition, Biomass Yield and Mineral Contents of Vegetation in Native Tract of Mecheri Sheep. Animal Nutrition and Feed Technology. **2012**, *12*, 63–71.
724. Kribek B, Mihaljevic M, Sracek O, Knesl I, Ettler V, Nyambe I. The Extent of Arsenic and of Metal Uptake by Aboveground Tissues of Pteris vittata and Cyperus involucratus Growing in Copper– and Cobalt–Rich Tailings of the Zambian Copperbelt. Arch Environ Contam Toxicol. **2011**, *61*, 228–242.
725. Kribek B, Mihaljevic M, Sracek O, Knesl I, Ettler V, Nyambe I. The Extent of Arsenic and of Metal Uptake by Aboveground Tissues of Pteris vittata and Cyperus involucratus Growing in Copper– and Cobalt–Rich Tailings of the Zambian Copperbelt. Arch Environ Contam Toxicol. **2011**, *61*, 228–242.
726. Osaki M, Watanabe T, Ishizawa T, Nilnond C, Nuyim T, Sittibush C, Tadano T. Nutritional characteristics in leaves of native plants grown in acid sulfate, peat, sandy podzolic, and saline soils distributed in Peninsular Thailand. Plant and Soil. **1998**, *201*, 175–182.
[truncated: 327,553 more chars]
